# Total Synthesis of Kibdelomycin

**DOI:** 10.1002/anie.202206183

**Published:** 2022-07-06

**Authors:** Chi He, Yu Wang, Cheng Bi, David S. Peters, Timothy J. Gallagher, Johannes Teske, Jason S. Chen, Rachel Corsetti, Anthony D'Onofrio, Kim Lewis, Phil S. Baran

**Affiliations:** ^1^ Department of Chemistry Scripps Research 10550N. Torrey Pines Road La Jolla CA 92122 USA; ^2^ Department of Biology Northeastern University 360 Huntington Avenue Boston Ma 02115 USA

**Keywords:** Antibiotics, Glycosidation, Modular Synthesis, Total Synthesis

## Abstract

A modular total synthesis of kibdelomycin is disclosed that should enable structure–activity relationship (SAR) studies of this interesting class of antibiotics. The route uses simple building blocks and addresses lingering questions about its structural assignment and relationship to amycolamicin, a recently described natural product reported to have a similar structure. Initial antibacterial assays reveal that both C‐22 epimers (the *N*‐glycosidic linkage) of the natural product have similar activity while structurally truncated analogs lose activity.

## Graphical abstract

A convergent synthesis of kibdelomycin, which leveraged readily available starting materials, enabled the efficient preparation of the potent antimicrobial and its C‐22 epimer in 16 steps (LLS). The modularity of the strategy provided rapid access to 8 analogs, allowing investigation of structural activity relation.

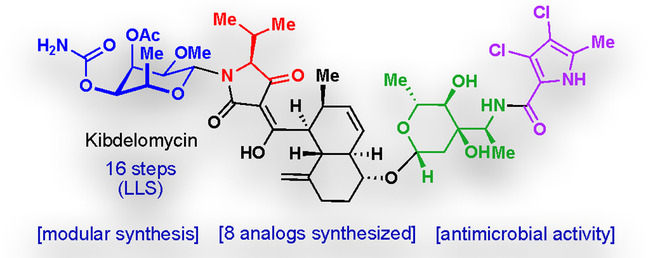

It has become apparent and generally accepted that there is a pressing need for the identification of new antibiotics, yet their discovery remains at historic lows. While important work is focused on the identification of new biochemical targets, studying structurally unique inhibitors of clinically validated ones provides the benefit of tools, data, and a path forward that, in part, has already been blazed. The inhibition of DNA synthesis/replication represents one of the major validated strategies for antibiotic therapeutics. A natural product antibiotic that inhibits this process is kibdelomycin (**1 b**, Figure 1). Isolated by chemists at Merck in 2012, it was found to be a strong inhibitor of type II topoisomerases (DNA gyrase and topoisomerase IV), though has a structure unique to that of other topoisomerase inhibitors.^[1]^ Its antibacterial activity is also notable. Particularly, its activity against important human pathogens *Acinetobacter baumannii* (MIC_50_=≤0.015 μg mL^−1^, MIC_90_=0.125 μg mL^−1^; 19 isolates) and *Clostridium difficile* warrant further investigation.^[2]^ Despite appearing in numerous reviews highlighting its potential,^[3]^ little work has been published since the initial reports until very recently, the Li and Kuwahara groups disclosed the synthesis of kibdelomycin and amycolamicin, respectively.[^4^, ^5^] Further studies would benefit from synthetic access to **1 b** and analogs thereof to interrogate its biological effects and SAR (enabled by preexisting structural data). However, due to its complex and chemically diverse structure, semi‐synthetic efforts beyond simple manipulations would likely prove challenging.

**Figure 1:**
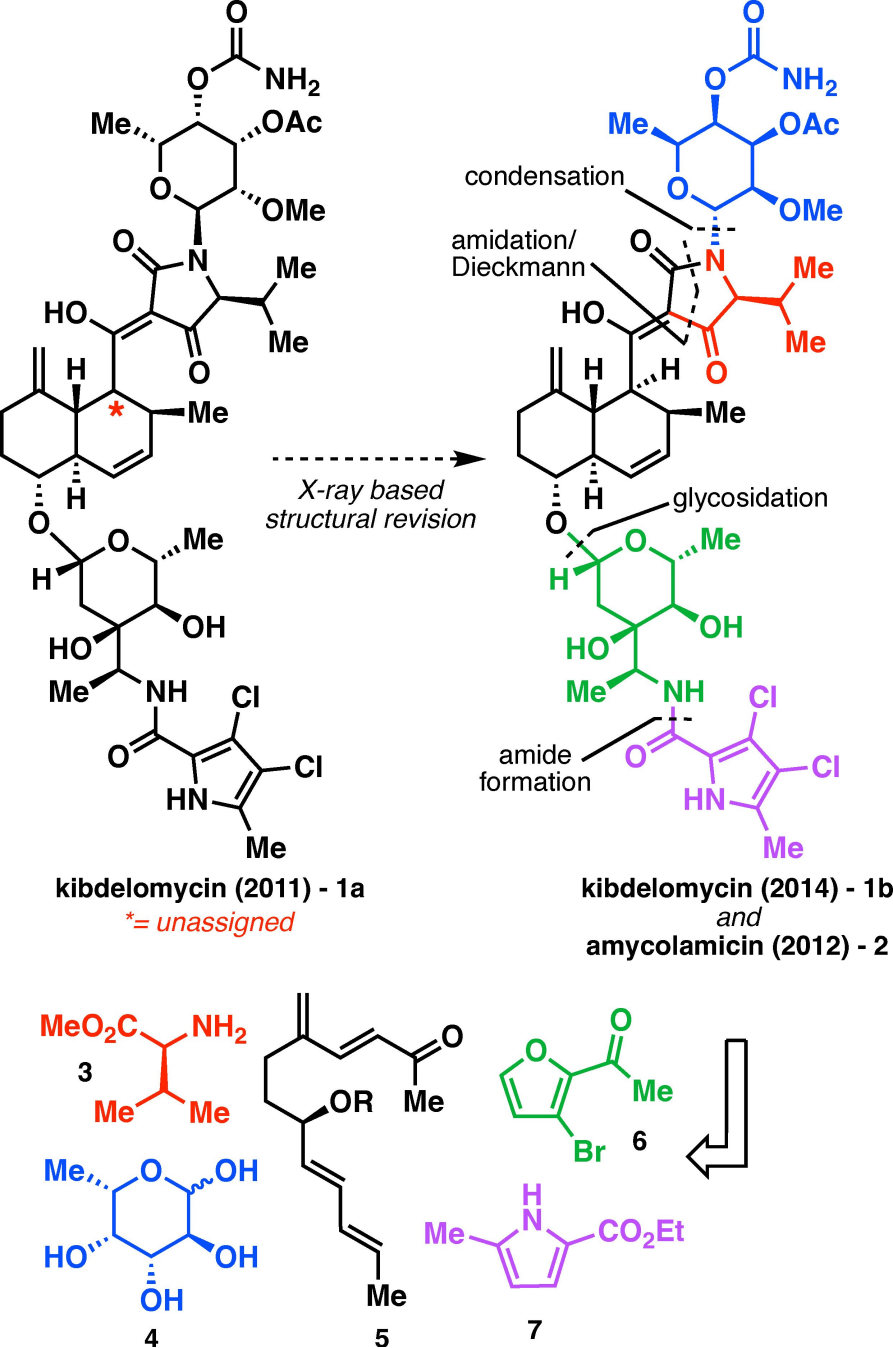
Structure and retrosynthetic analysis of kibdelomycin.

Adding interest to the kibdelomycin story was the question about its identity with respect to another seemingly related natural product, amycolamicin (**2**).^[6]^ Kibdelomycin was first isolated in 2011 and assigned as shown in Figure 1 (**1 a**). Upon full characterization of **2** in 2012, the molecules were thought to be diastereomers of each other due to differing stereochemical assignments and spectroscopic data. However, a structural reassignment of kibdelomycin to **1 b** based on X‐ray crystal data suggested that its structure was identical to that of **2**.^[7]^ With the structural assignment of each molecule seemingly verified, the cause of the spectroscopic discrepancies remained a mystery. Taken together, these features make **1 b** a compelling candidate for total synthesis. In this Communication, a total synthesis is presented that settles lingering structural questions surrounding this family via a highly convergent route that is amenable to rapid analog synthesis.

The structure of kibdelomycin appears rather daunting at first glance, however the multi‐component nature of the molecule points to conventional polar disconnections of the subunits that largely emulate those utilized by Nature (Figure 1).^[8]^ Performing these disconnections breaks the molecule into five distinct fragments (**3**–**7**), which each can ultimately be traced back to their highly simplified starting materials shown in Figure 1 (except for the decalin core whose linear intramolecular Diels–Alder (IMDA) precursor **5** is shown). This approach is not only attractive due to its convergent nature, but should also be directly translatable to any future analog syntheses.

Beginning with the northern two fragments, the focus was first placed on the 6‐deoxytallose derivative (Figure 1, blue). l‐Fucose (**4**) was chosen as the starting material as it contained nearly all desired stereocenters requiring only one inversion and three selective functionalizations to arrive at the target compound. As such, **4** was first protected as the acetal with benzyl alcohol (Scheme 1A). The 3,4‐diol of the resulting intermediate was then selectively protected as the acetonide to provide **8**. The remaining alcohol was then inverted through an oxidation/reduction sequence providing **9** as a single diastereomer, aided by the steric environment provided by the acetonide. Unfortunately, only the α‐anomer was competent for use in the oxidation/reduction sequence. Having all the permanent stereocenters set, the next task was to selectively functionalize each of the alcohols with their corresponding appendages. The previously inverted alcohol was methylated to provide **10**. The acetonide was then liberated and the resulting diol selectively acylated under standard conditions, yielding **11**, due to the axial nature of the C4‐OH. Next, the carbamate was installed through the use of trichloroacetyl isocyanate (**12**),^[9]^ whose enhanced reactivity and relative ease of deprotection at a late stage proved crucial for clean installation of the desired functional group. Finally, the benzyl group at the anomeric position was removed by hydrogenolysis (along with an inconsequential mono‐dechlorination) followed by condensation of the crude product with l‐valine methyl ester to provide aminal **14** as the β‐anomer depicted (single diastereomer). Although this is the opposite stereochemistry as that found in the natural product, it was of no consequence due to the lability of this stereocenter (see below).

**Scheme 1:**
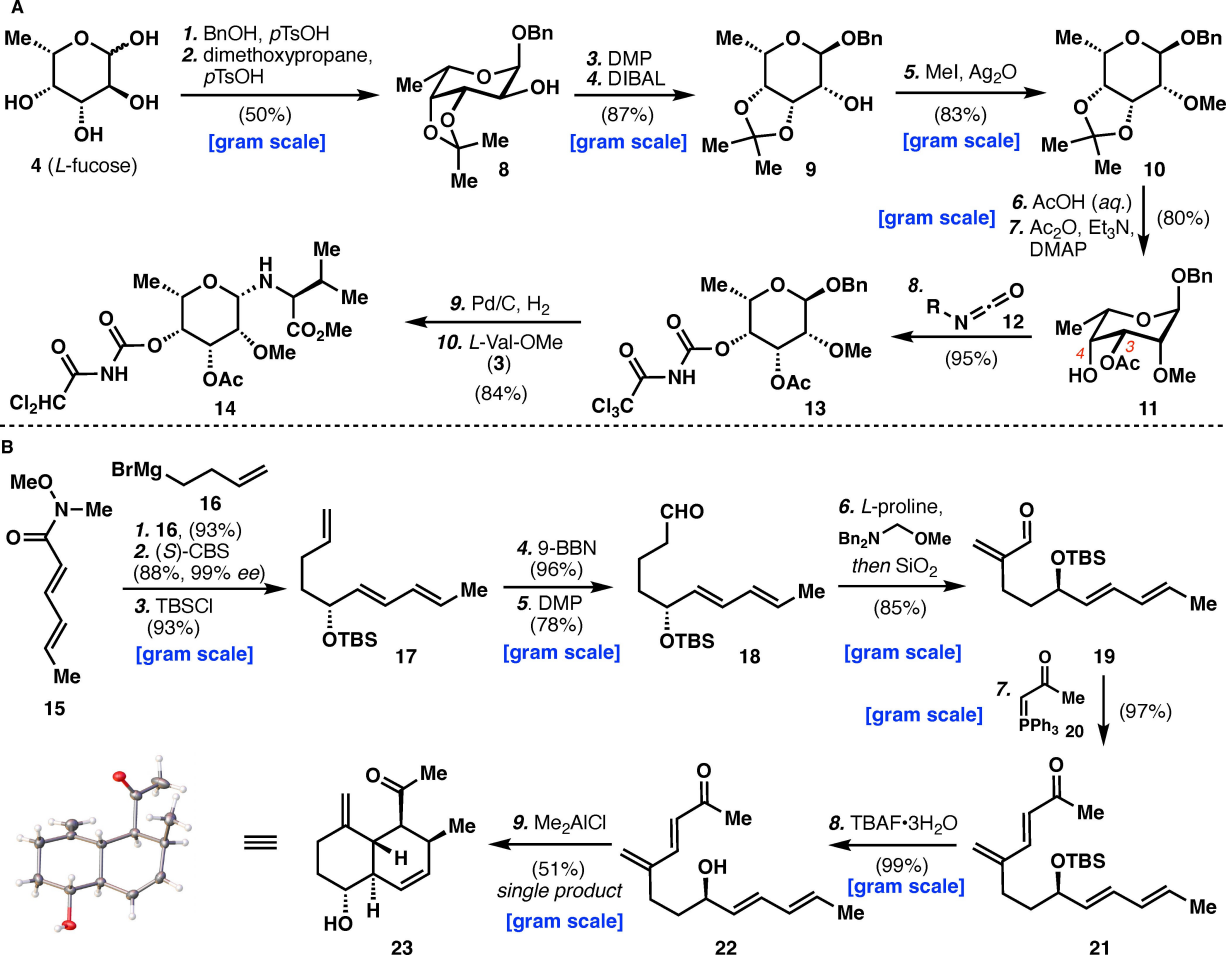
Synthesis of intermediates **14** and **23**. Reagents and conditions: A) 1) BnOH (10.0 equiv), *p*TsOH (0.1 equiv), 80 °C, overnight; 2) 2,2‐dimethoxypropane (3.0 equiv), *p*TsOH (0.2 equiv), DMF, rt, overnight, 50 % (2 steps); 3) DMP (1.75 equiv), DCM, rt, 2 h; 4) DIBAL (2.5 equiv), THF, −78 °C to rt, overnight, 87 % (2 steps); 5) MeI (6.0 equiv), Ag_2_O (3.0 equiv), CH_3_CN, 75 °C, overnight, 83 %; 6) 80 % *aq*. AcOH, 80 °C, 1 h; 7) Ac_2_O (1.05 equiv), Et_3_N (1.1 equiv), DMAP (0.05 equiv), DCM, rt, overnight, 80 % (2 steps); 8) **12** (2.0 equiv), DCM, 0 °C to rt, 1 h, 95 %; 9) Pd/C (10 % w/w), EtOAc, rt, 3 h; 10) H_2_N‐Val‐OMe (1.8 equiv), PPTS (0.2 equiv), DCM, rt, 6 h, 84 % (2 steps). B) 1) **16** (1.4 equiv), THF, 0 °C, 4 h, 93 %; 2) (*S*)‐CBS (2.0 equiv), BH_3_⋅THF (2.2 equiv), THF, −78 °C, 5 h, 88 % yield, 99 % *ee*; 3) TBSCl (1.5 equiv), imidazole (3.0 equiv), DMF, 50 °C, overnight, 93 %; 4) 9‐BBN (1.4 equiv), THF, 0 °C to rt, 5 h, then NaBO_3_⋅4H_2_O (4.0 equiv), H_2_O, 0 °C to rt, overnight, 96 %; 5) DMP (1.4 equiv), DCM, rt, 2.5 h, 78 %; 6) Bn_2_NCH_2_OMe (1.1 equiv), *L*‐proline (0.2 equiv), DMF, 0 °C to rt, 2 h, then SiO_2_, DCM, rt, 5 h, 85 %; 7) **20** (1.8 equiv), DCM, 45 °C, 24 h, 97 %; 8) TBAF⋅3H_2_O (2.0 equiv), THF, 0 °C to rt, 2 h, 99 %; 9) Me_2_AlCl (1.0 equiv), DCM, −20 °C to rt, 18 h, 51 %. Bn=benzyl, *p*TsOH=*p*‐toluenesulfonic acid, DMF=*N*,*N*‐dimethylformamide, DMP=Dess–Martin periodinane, DCM=dichloromethane, DIBAL=diisobutylaluminum hydride, THF=tetrahydrofuran, Ac=acetyl, DMAP=*N*,*N*‐4‐dimethylaminopyridine, PPTS=pyridinium *p*‐toluenesulfonate, CBS=Corey–Bakshi–Shibata reagent, TBSCl=*tert*‐butyldimethylsilyl chloride, 9‐BBN=9‐borabicyclo[3.3.1]nonane, TBAF=*tetra*‐*n*‐butylammonium fluoride.

Moving to the decalin core (Scheme 1B), its synthesis proved to be rather challenging. Numerous routes were evaluated with some failing at very late stages (see Supporting Information). While a variety of IMDA substrates were evaluated, most suffered from issues with relative stereochemistry, diastereoselectivity, and reactivity of the products.^[10]^ Ultimately the use of the IMDA shown above proved successful with respect to these aspects. The successful synthesis of the decalin core began with the 1,2‐addition of alkyl Grignard reagent **16**, into Weinreb amide (**15**). In order to proceed with only one enantiomer, CBS reduction (99 % *ee*) was utilized followed by TBS protection of the desired allylic alcohol to provide **17**. The terminal olefin of **17** was transformed to the primary aldehyde via selective hydroboration/oxidation with 9‐BBN followed by DMP oxidation of the resulting alcohol. With aldehyde **18** in hand, a simple Mannich addition using substoichiometric l‐proline was performed followed by silica‐promoted elimination of the resultant tertiary amine.^[11]^ The resulting enal **19**, was then subjected to Wittig olefination with stabilized ylide **20** to provide tetraene **21**. When this compound was treated with Me_2_AlCl it exhibited no stereocontrol in the Diels–Alder (DA) reaction (see Supporting Information for a summary of conditions). In stark contrast, when the TBS group was removed to deliver free allylic alcohol **22**, the same intramolecular DA reaction provided the desired product (**23**) as a single diastereomer in 51 % isolated yield (as confirmed through X‐ray crystallography^[12]^). The chelation between the free hydroxyl group and Lewis acid is essential for the selectivity.^[5b]^

Lastly, the 2,6‐dideoxyhexopyranose moiety (Scheme 2), previously dubbed amycolose, offered an interesting challenge in that unlike a common sugar pyranose, not all the stereocenters of the fragment reside on the ring. With this in mind, it was clear that the pyranose would need to be constructed rather than adapted from the chiral pool. It was hoped that an enone resulting from an Achmatowicz reaction^[13]^ would provide a congener of the desired compound only varying by oxidation state. This approach would allow the exocyclic stereocenter to be installed, as well as another before the pyranose is formed, divorcing the formation of those chiral centers from the possible control or interference of the inconsequential anomeric stereocenter. Beginning from 2‐acetyl‐3‐bromofuran, the first stereocenter was set through a Noyori reduction, which after TBS‐protection provided furan **24** in 92 % *ee* (see Supporting Information). The α‐amino stereocenter was then installed via a lithium halogen exchange followed by addition to a chiral sulfinimine (**25**) bearing an Ellman's auxiliary, providing **26** as the major diastereomer (5.5 : 1). The silyl protecting group was next removed, setting the stage for the Achmatowicz reaction. Only singlet oxygen proved effective in providing the desired product **27**.^[14]^ Treating **27** with HCl in trichloroethanol installed a mixed acetal and simultaneously revealed the primary amine, which was subsequently acylated with pyrrole **28** to give **29**. Attempts to convert **29**, and related enones to the desired diol via a reduction/hydration sequence were unsuccessful due to stereochemical considerations (see Supporting Information), thus the following lengthier approach was utilized. Reduction of the enone under Luche conditions provided the allylic alcohol as a single diastereomer.^[15]^ The complete stereocontrol of this reaction can be rationalized by considering that the top face of the carbonyl is disfavored for hydride attack by the axial methyl group next to the nitrogen atom. Directed allylic epoxidation with peroxytrifluoroacetic acid was successful in providing the desired epoxy‐alcohol **30** with complete stereocontrol. The opening of the epoxide was found to be challenging due to steric hinderance. A series of reductants were screened, such as, DIBAL, Red‐Al, LiBHEt_3_, leaded to either decomposition or no reaction. Fortunately, Lithium borohydride was found to be singularly useful in this step. The resulting diol was subsequently protected as the silyl ether (**31**) setting the stage for union with the decalin fragment **23**.

**Scheme 2:**
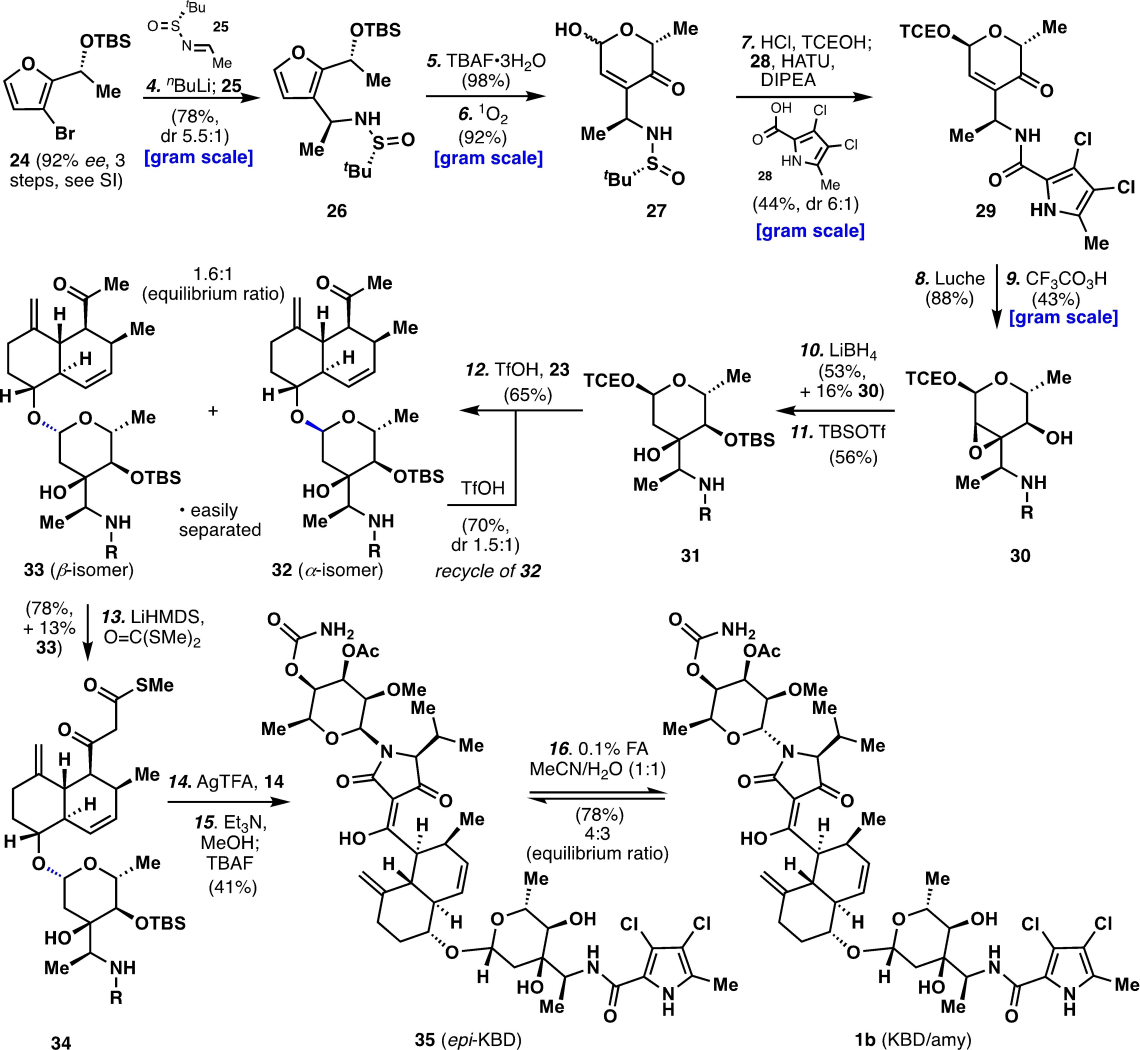
Total Synthesis of kibdelomycin (**1 b**). Reagents and conditions: For the synthesis of compound **24**, see step 1–3 in Supporting Information; 4) *n*‐BuLi (1.1 equiv), Et_2_O, −40 °C, 1 h, then **25** (1.2 equiv), −78 °C to rt, 2 h, 78 % (*d.r*. 5.5 : 1); 5) TBAF⋅3H_2_O (2.0 equiv), THF, rt, 0.5 h, 98 %; 6) MB (0.0014 equiv), O_2_, DCM, −78 °C, 2.5 h, then Me_2_S (5.0 equiv), −78 °C to rt, 2 h, 92 %; 7) *p*TsOH (0.2 equiv), TCEOH, rt, 1.5 h, then HCl (2.0 equiv), rt, 1.5 h, then HATU (2.0 equiv), **28** (2.0 equiv), DIPEA (5.5 equiv), DMF, rt, 8 h, 44 % (*d.r*. 6 : 1); 8) NaBH_4_ (4.0 equiv), CeCl_3_⋅7H_2_O (0.4 equiv), MeOH, 0 °C, 20 min, 88 %; 9) CF_3_CO_3_H (1.36 equiv), DCM, −40 °C to rt, 2 h, 43 %; 10) LiBH_4_ (6.0 equiv), toluene, 60 °C, 3 h, 53 % **S9**+16 % **30**; 11) TBSOTf (6.0 equiv), Et_3_N (10.0 equiv), DCE, 7 h, 56 %; 12) **23** (2.0 equiv), 4 A MS, TfOH (1.0 equiv), DCM, rt, 2.5 h, 65 % (1.6 : 1, β:α); 12′) **23** (2.0 equiv), TCEOH (1.0 equiv), 4 A MS, TfOH (2.0 equiv), DCM, rt, 2.5 h, 70 % (1.5 : 1, β:α); 13) LiHMDS (20.0 equiv), CO(SMe)_2_ (12.0 equiv), THF, −78 to 30 °C, 6.5 h, 78 % **34**+13 % **33**; 14) **14** (3.0 equiv), 4 A MS, AgTFA (5.0 equiv), THF, rt, 2 h; 15) Et_3_N (5.0 equiv), MeOH, rt, 10 min, then TBAF (8.0 equiv), THF, rt, 0.5 h, 41 % (2 steps); 16) 0.1 % HCO_2_H (4.4 equiv) in MeCN/H_2_O, rt, 24 h, 78 % (4 : 3, **35**/**1 b**). Bu=butyl, MB=methylene blue, TCE=trichloroethyl, HATU=*O*‐(7‐azabenzotriazol‐1‐yl)‐*N*,*N*,*N′*,*N′*‐tetramethyluronium hexafluorophosphate, DIPEA=diisopropylethylamine, AgTFA=silver trifluoroacetate, Tf=trifluoromethanesulfonyl, DCE=dichloroethane, LiHMDS=lithium bis(trimethylsilyl)amide.

Glycosidation of the decalin core **23** proved to be rather difficult for a variety of reasons. First, with the diol of amycolose protected in any form, the introduced steric environment makes the formation of glycosyl donors difficult. Second, β‐selectivity was difficult to achieve under most conditions, particularly when the diol was left unprotected. It was eventually discovered that TBS protection of the secondary‐OH still allowed for the formation of glycosyl donor. These studies are summarized in the Supporting Information which eventually inspired a more simple and direct approach. Thus, simply treating **31** and **23** with TfOH (1.0 equiv) at room temperature in the presence of molecular sieves led to an easily separable 1.6 : 1 mixture of glycosylated decalins **32** and **33** in 65 % yield. The undesired α‐isomer (**32**) could be easily recycled by resubjecting to the same conditions to obtain the same 1.5 : 1 ratio of products. Subsequent homologation was achieved by deprotonation with LiHMDS followed by treatment with *S*,*S*‐dimethyl dithiocarbonate.^[16]^ It was found that the enolate formed is particularly unreactive. As such, an excess of base and the electrophile, as well as gentle heating, were necessary to achieve good yields. The resulting β‐ketothioester (**34**) was treated with silver trifluoroacetate in the presence of aminal**14** (3.0 equiv) to generate a β‐ketoamide intermediate.^[17]^ The crude material from this reaction was immediately treated with Et_3_N/MeOH to remove the dichloroacetamide group on the top sugar part, followed by TBAF to both remove the silyl group and effect the desired Dieckmann cyclization providing *epi*‐kibdelomycin (**35**).^[18]^

Upon isolation of **35**, it was found that the β‐anomer of the northern sugar had formed rather than the natural, α‐anomer. The original isolation publication of **2** suggested that there existed an equilibrium between the α‐ and β‐isomers.^[6]^ Inference from data shown below suggested that this might be acid‐catalyzed. As such treatment **35** with 0.1 % formic acid in 1 : 1 (CH_3_CN/H_2_O) at room temperature for 24 h provided a 4 : 3 ratio of the two anomers. This ultimately provides a potential way to convert all material synthesized to the desired final product and completed the total synthesis of **1 b**.

The following observations are supportive of **1 b** and **2** being a conjugate base and acid pair, respectively. First evidence for this hypothesis can be obtained from the isolation literature. The *λ*_max_ for **1 b** (in MeOH) was reported as 248 and 276 nm.^[1]^ The *λ*_max_ for **2** was reported as 280 nm in acidic MeOH but shifts to 248 and 277 nm in basic MeOH.^[6]^ Noting that differences in isolation procedures for the respective natural products could account for samples of **2** being slightly acidic, we set out to investigate the spectroscopic dependence on pH of the compound(s). The ^1^H NMR of a natural sample of **2** (provided graciously by Prof. Adachi) in CD_3_OD matches the reported NMR of **2**. Treatment of this solution with NaHCO_3_ results in a shift of the signals and the resulting ^1^H NMR spectrum matches that of **1 b** (Figure 2). Finally, co‐injection of synthetic **1 b**, whose stereochemistry matches that of the reported co‐crystal structure, with natural **2** on an HPLC‐MS (eluent containing 0.1 % formic acid) demonstrates that the natural and synthetic samples have the same identity (t_R and_ MS fragmentation, see Supporting Information). This is consistent with the independent discoveries of the other two groups that completed the synthesis of these natural products.^[5]^

**Figure 2:**
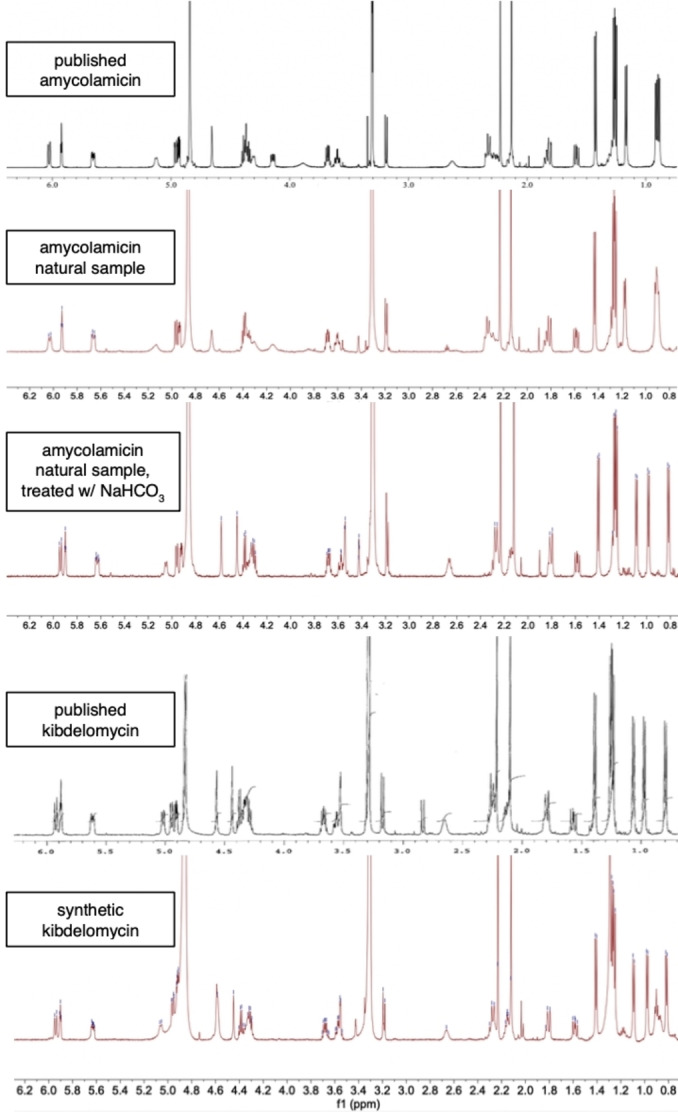
^1^H NMR comparison of kibdelomycin and amycolamicin at varying pH (spectra taken in CD_3_OD).

Synthetic kibdelomycin (**1 b**) was tested for antibacterial activity (Table 1) and had nearly identical activity against the same *S. aureus* FDA209P and analogous *E. coli* strains (wild type MG1655^[20]^ and permeabilized W0153^[21]^) compared to the literature data.[^1^, ^6^, ^19^] Interestingly, *epi*‐kibdelomycin (**35**) had nearly identical activity against *S. aureus* and the permeabilized *E. coli* strain. This may be indicative of either epimerization in the assay, the ease of which is demonstrated above, or promiscuity at the site of binding of this motif (crystal data suggests the 6‐deoxytallose is in a solvent‐exposed region^[6]^). The wild type *E. coli* was insensitive to **35**, possibly due to poor penetration. The modular nature of the synthesis was also leveraged to provide seven truncated analogs (see Supporting Information). Unfortunately, these compounds had little to no antibacterial activity, demonstrating the necessity of each substructure for the natural product's antibacterial activity.

**Table 1:**
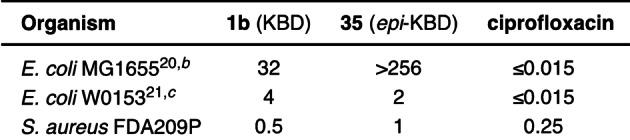
MIC assay of **1 b** and **35** ^[a]^ (μg mL^−1^).

[a] MIC was determined by microdilution assay using Meuller Hinton II Broth. [b] Wild‐type strain. [c] Permeabilized. MIC: Minimum inhibitory concentration.

In conclusion, a total synthesis of kibdelomycin/amycolamicin has been completed providing another route to access to this promising antibiotic class. This investigation also demonstrated the connection between two seemingly different natural products as being a simple acid/conjugate base pair. Any distinction between the two becomes irrelevant under the physiological conditions in which the compounds are tested/used as is demonstrated by their nearly identical activities.[^1^, ^6^] This important information along with the modular synthetic route presented here will aid in the synthesis of analogs of **1 b**.

## Conflict of interest

The authors declare no conflict of interest.

## Supporting information

As a service to our authors and readers, this journal provides supporting information supplied by the authors. Such materials are peer reviewed and may be re‐organized for online delivery, but are not copy‐edited or typeset. Technical support issues arising from supporting information (other than missing files) should be addressed to the authors.

Supporting Information

## Data Availability

The data that support the findings of this study are available in the Supporting Information of this article.
