# Supplementary material for: Total Synthesis of Kibdelomycin
Source: Angew Chem Int Ed Engl. 2022 Jul 6;61(32):e202206183. doi: 10.1002/anie.202206183 (PMC9357209; doi:10.1002/anie.202206183)
Supplement: Supplementary file 1 — Supporting Information [file ANIE-61-0-s001.pdf]

Angewandte Chemie
Eine Zeitschrift der Gesellschaft Deutscher Chemiker

Supporting Information

Total Synthesis of Kibdelomycin

*C. He, Y. Wang, C. Bi, D. S. Peters, T. J. Gallagher, J. Teske, J. S. Chen, R. Corsetti, A. D'Onofrio, K. Lewis, P. S. Baran**

Supplementary Materials

I) Experimental Section

II) Chiral SFC Results

III) X-Ray Crystallography Data

IV) References

V) NMR Spectra

I) General Experimental

All reactions were carried out under an inert argon atmosphere with dry solvents under anhydrous conditions unless otherwise stated. Dry acetonitrile (MeCN), dichloromethane (DCM), diethyl ether (Et_2O), tetrahydrofuran (THF), toluene (PhMe), dimethylformamide (DMF), benzene, and triethylamine (Et_3N) were obtained by passing the previously degassed solvents through activated alumina columns. Reagents were purchased at the highest commercial quality and used without further purification, unless otherwise stated. Yields refer to chromatographically and spectroscopically (^1H NMR) homogeneous material, unless otherwise stated. Reactions were monitored by thin layer chromatography (TLC) carried out on 0.25 mm E. Merck silica plates (60F-254), using UV light as the visualizing agent and/or phosphomolybdic acid and heat as a developing agent. Flash silica gel chromatography was performed using E. Merck silica gel (60, particle size 0.043 – 0.063 mm). NMR spectra were recorded on Bruker DRX-600 and AMX-400 instruments and were calibrated using residual undeuterated solvent as an internal reference ($CDCl_3$: ^1H NMR δ = 7.26 ppm, ^{13}C NMR δ = 77.16 ppm; acetone-*d6*: ^1H NMR δ = 2.05 ppm, ^{13}C NMR δ = 49.84 ppm; CD_3OD: ^1H NMR δ = 3.31 ppm, ^{13}C NMR δ = 49.00 ppm). The following abbreviations were used to explain NMR peak multiplicities: s = singlet, d = doublet, t = triplet, q = quartet, p = quint, m = multiplet, br = broad. High-resolution mass spectra (HRMS) were recorded on an Agilent LC/MSD TOF mass spectrometer by electrospray ionization time-of-flight (ESI-TOF) reflectron experiments.

The detailed route and conditions:

Scheme S1. The synthesis of intermediates **14** and **23**[a]

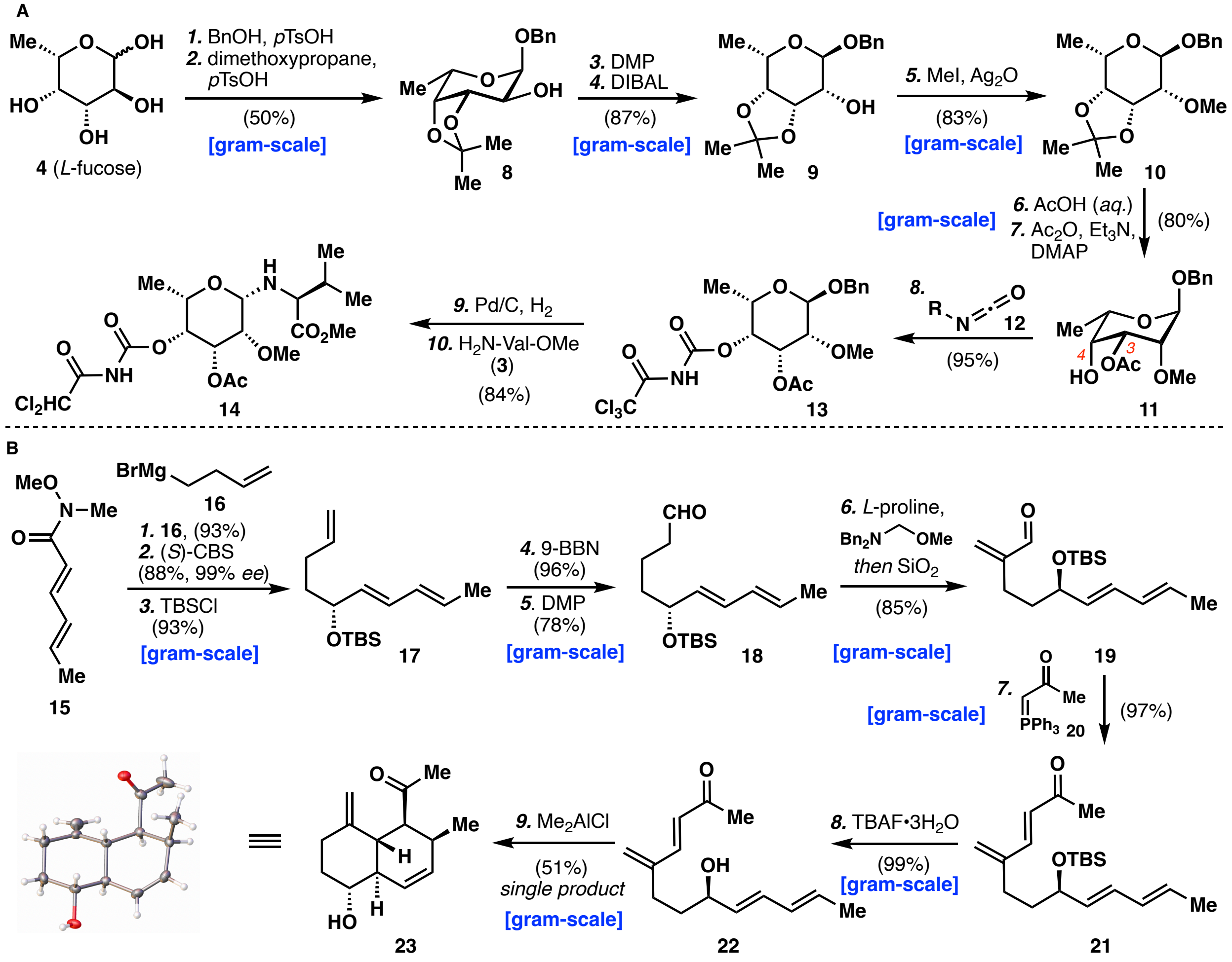

[a]Reagents and conditions:

A (for **14**)**:** (1) BnOH (10.0 eq.), *p*TsOH (0.1 eq.), 80 °C, overnight; (2) 2,2-dimethoxypropane (3.0 eq.), *p*TsOH (0.2 eq.), DMF, rt, overnight, 50% (2 steps); (3) DMP (1.75 eq.), DCM, rt, 2 h; (4) DIBAL (2.5 eq.), THF, –78 °C to rt, overnight, 87% (2 steps); (5) MeI (6.0 eq.), Ag_2O (3.0 eq.), CH_3CN, 75 °C, overnight, 83%; (6) 80% *aq.* AcOH, 80 °C, 1 h; (7) Ac_2O (1.05 eq.), Et_3N (1.1 eq.), DMAP (0.05 eq.), DCM, rt, overnight, 80% (2 steps); (8) **12** (2.0 eq.), DCM, 0 °C to rt, 1 h, 95%; (9) Pd/C (10% w/w), EtOAc, rt, 3 h; (10) H_2N-Val-OMe (1.8 eq.), PPTS (0.2 eq.), DCM, rt, 6 h, 84% (2 steps).

B (for **23**)**:** (1) **16** (1.4 eq.), THF, 0 °C, 4 h, 93%; (2) (*S*)-CBS (2.0 eq.), BH_3·THF (2.2 eq.), THF, –78 °C, 5 h, 88% yield, 99% *ee*; (3) TBSCl (1.5 eq.), imidazole (3.0 eq.), DMF, 50 °C, overnight, 93%; (4) 9-BBN (1.4 eq.), THF, 0 °C to rt, 5 h, then $NaBO_3·4H_2O$ (4.0 eq.), H_2O, 0 °C to rt, overnight, 96%; (5) DMP (1.4 eq.), DCM, rt, 2.5 h, 78%; (6) Bn_2NCH_2OMe (1.1 eq.), *L*-proline (0.2 eq.), DMF, 0 °C to rt, 2 h, then SiO_2, DCM, rt, 5 h, 85%; (7) **20** (1.8 eq.), DCM, 45 °C, 24 h, 97%;

(8) TBAF•$3H_2O$ (2.0 eq.), THF, 0 °C to rt, 2 h, 99%; (9) Me_2AlCl (1.0 eq.), DCM, –20 °C to rt, 18 h, 51%.

Abbreviations: Bn, benzyl; *p*TsOH, *p*-toluenesulfonic acid; DMF, *N*,*N*-dimethylformamide; DMP, Dess-Martin periodinane; DCM, dichloromethane; DIBAL, diisobutylaluminum hydride; THF, tetrahydrofuran; Ac, acetyl; DMAP, *N*,*N*-4-dimethylaminopyridine; PPTS, pyridinium *p*-toluenesulfonate; CBS, Corey-Bakshi-Shibata reagent; TBSCl, *tert*-butyldimethylsilyl chloride; 9-BBN, 9-borabicyclo[3.3.1]nonane; TBAF, *tetra*-*n*-butylammonium fluoride.

Scheme S2. The synthesis of intermediates *epi*-KBD (**35**) and KBD (**1b**)[a]

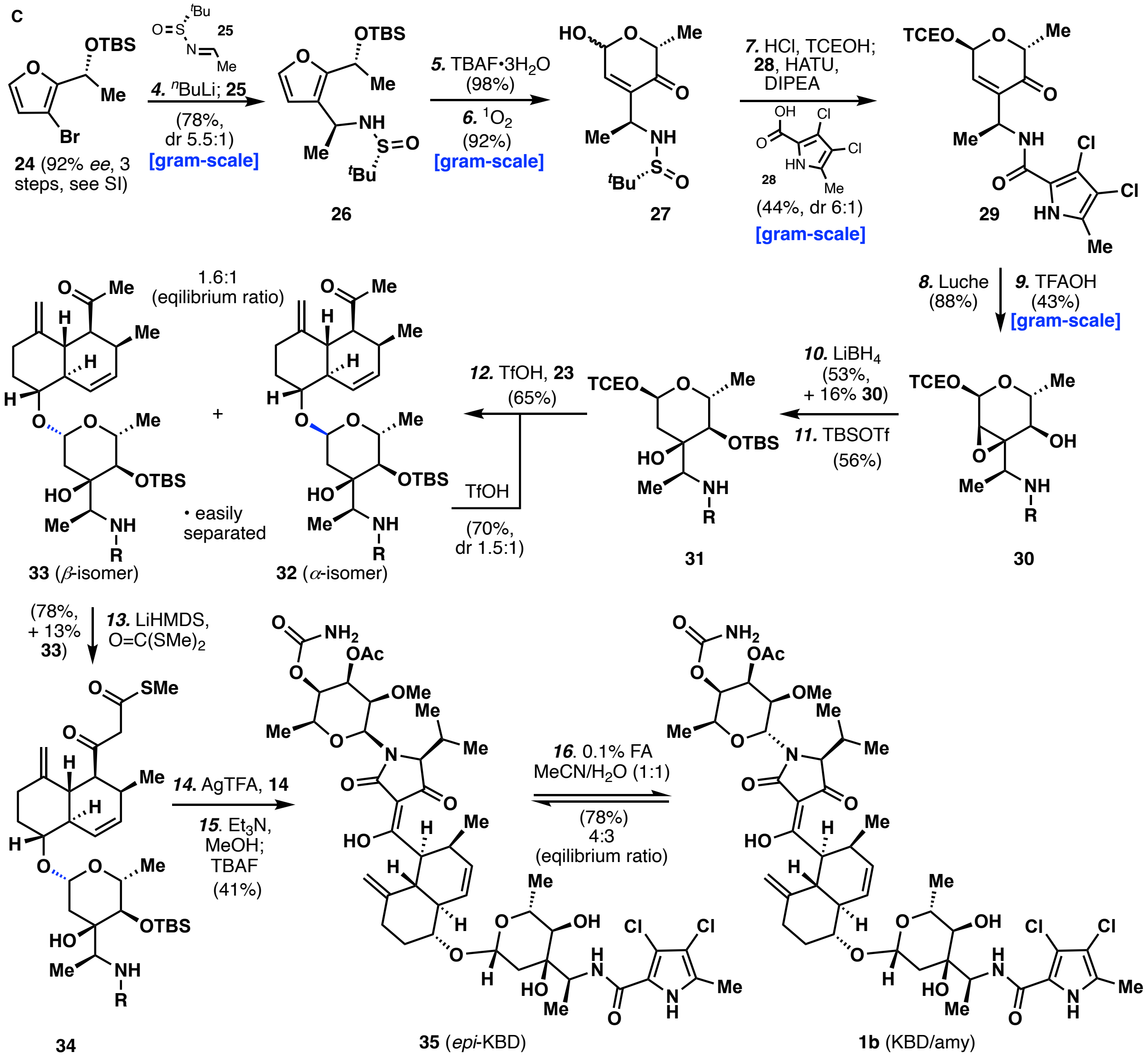

a) Reagents and conditions:

C (for **35** and **1b**)**:** For the synthesis of compound **24**, see steps 1-3: (1) 2-bromofuran (1.0 eq.), AcCl (3.2 eq.), $AlCl_3$ (2.9 eq.), DCM, 0 °C to rt, 75 min, 93%; (2) [$RuCl_2$(*p*Cymene)]$_2$ (0.0075 eq.), *N*-((1*R*,2*R*)-2-amino-1,2-diphenylethyl)-4-methylbenzenesulfonamide (0.018 eq.), HCO_2Na (5.0 eq.), THF/H_2O (1/2, v:v), 40 °C, 48 h, 92% yield, 92% *ee*; (3) TBSCl (1.5 eq.), imidazole (3.0 eq.), DMF, rt, overnight, 95%; (4) *n*-BuLi (1.1 eq.), Et_2O, –40 °C, 1 h, then **25** (1.2 eq.), –78 °C to rt, 2 h, 78% (*d.r.* 5.5:1); (5) TBAF•$3H_2O$ (2.0 eq.), THF, rt, 0.5 h, 98%; (6) MB (0.0014 eq.), O_2, DCM, –78 °C, 2.5 h, then Me_2S (5.0 eq.), –78 °C to rt, 2 h, 92%; (7) *p*TsOH (0.2 eq.), TCEOH, rt, 1.5 h, then HCl (2.0 eq.), rt, 1.5 h, then HATU (2.0 eq.), **28** (2.0 eq.), DIPEA (5.5 eq.), DMF, rt, 8 h, 44% (*d.r.* 6:1); (8) $NaBH_4$ (4.0 eq.), $CeCl_3{\cdot}7H_2O$ (0.4 eq.), MeOH, 0 °C, 20 min, 88%; (9) TFAOH (1.36 eq.), DCM, –40 °C to rt, 2 h, 43%; (10) $LiBH_4$ (6.0 eq.), toluene, 60 °C, 3 h, 53% **S9** + 16% **30**; (11) TBSOTf (6.0 eq.), Et_3N (10.0 eq.), DCE, 7 h, 56%; (12) **23** (2.0 eq.), 4 A MS, TfOH (1.0 eq.), DCM, rt, 2.5 h, 65% (1.6:1, β:α); (12') **23** (2.0 eq.), TCEOH (1.0 eq.), 4 A MS, TfOH (2.0 eq.),

DCM, rt, 2.5 h, 70% (1.5:1, β:α); (13) LiHMDS (20.0 eq.), $CO(SMe)_2$ (12.0 eq.), THF, –78 to 30 °C, 6.5 h, 78% **34** + 13% **33**; (14) **14** (3.0 eq.), 4 A MS, AgTFA (5.0 eq.), THF, rt, 2 h; (15) Et_3N (5.0 eq.), MeOH, rt, 10 min, then TBAF (8.0 eq.), THF, rt, 0.5 h, 41% (2 steps); (16) 0.1% HCO_2H (4.4 eq.) in $MeCN/H_2O$, rt, 24 h, 78% (4:3, **35**/**1b**). Abbreviations: Bu, butyl; MB, methylene blue; TCE, trichloroethyl; HATU, *O*-(7-azabenzotriazol-1-yl)-*N*,*N*,*N'*,*N'*-tetramethyluronium hexafluorophosphate; DIPEA, diisopropylethylamine; TFAOH, trifluoroperacetic acid; AgTFA, silver trifluoroacetate; Tf, trifluoromethanesulfonyl; DCE, dichloroethane; LiHMDS, lithium *bis*(trimethylsilyl)amide.

Scheme S3. The list of compounds for the antimicrobial activity test[a]

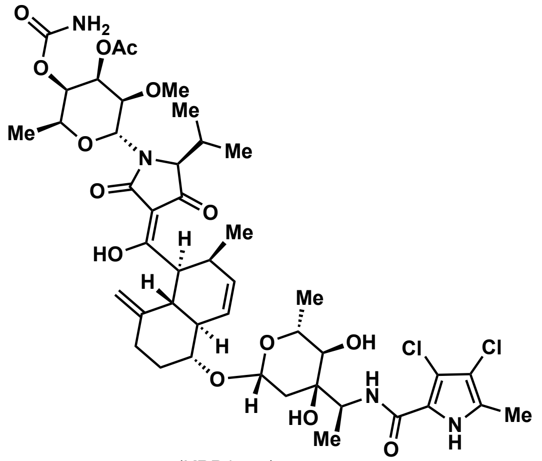

1 (KBD/amy)

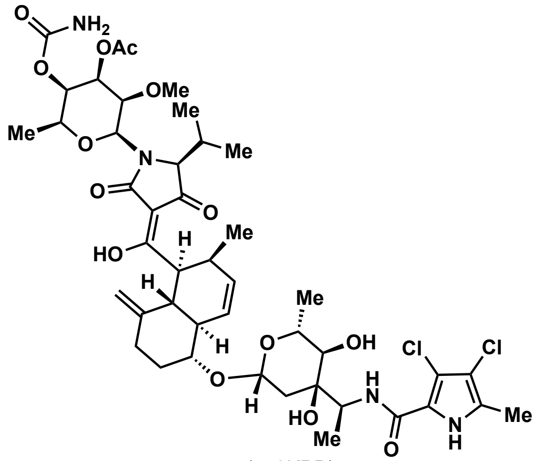

35 (*epi*-KBD)

analogs:

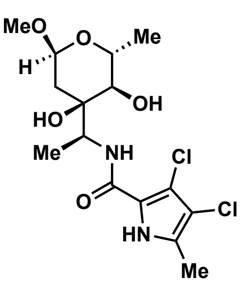

36

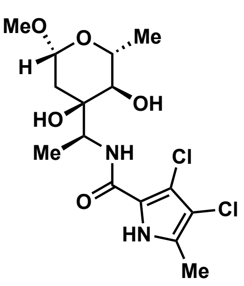

37

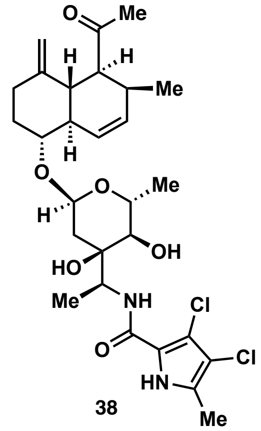

38

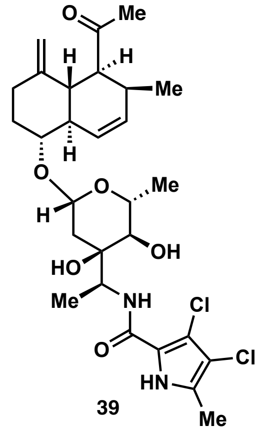

39

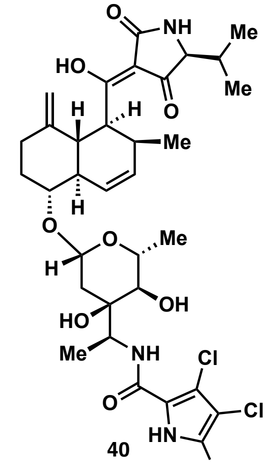

40

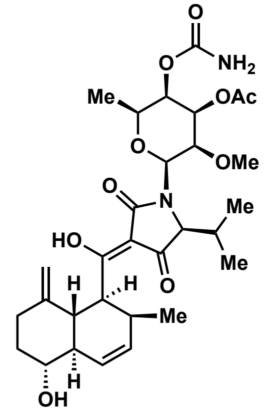

41

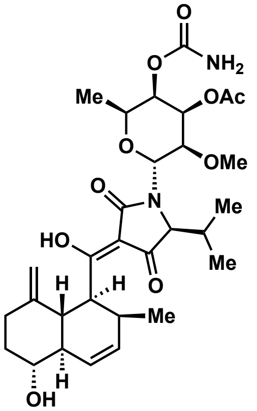

42

a) For the synthesis of analogs, please see the detailed procedures below.

Table S1. Minimum inhibitory concentration (MIC) of kibdelomycin (**1b**) and analogs[a] (μg/mL)

Organism	**1b** (KBD)	**35** (*epi*-KBD)	**36**	**37**	**38**	**39**	**40**	**41**	**42**	**ciprofloxacin**
E. coli MG1655[b]	32	>256	>256	>256	>256	>256	>256	>256	>256	≤0.015
E. coli W0153[c]	4	2	128	>256	>256	64	64	>256	>256	≤0.015
S. aureus FDA209P	0.5	1	-	-	-	-	-	256	>128	0.25
S. aureus HG003	-[d]	-	>64	>64	>64	>64	>64	-	-	0.25
S. aureus SH1000	-	-	>128	>128	>128	>128	>128	-	-	0.25
S. aureus UAMS-1	-	-	>128	>128	>128	>128	>128	-	-	0.25
S. aureus USA300	-	-	>128	>128	>128	>128	>128	-	-	-

[a] MIC was determined by microdilution assay using Meuller Hinton II Broth.
[b] wild-type strain. [c] permeabilized. [d] '-' = not available.

MIC determination

The microbroth dilution Minimum Inhibitory Concentration (MIC) method was used to determine the *in vitro* antibacterial activity of Kibdelomycin and its analogs against bacterial strains. Briefly, assay plates were prepared by two-fold dilution of each compound across the plate, and included a positive and growth control. Overnight liquid cultures of bacterial strains (*E. coli* MG1655, *E. coli* W0153, and *S. aureus* FDA209P) were diluted into the assay plate to achieve 5 x 10^5 CFU/mL and incubated at 37°C for 16-20 hours. The MIC was determined as the lowest concentration of the compound that inhibits growth of the bacteria as determined by the unaided eye. All assays were repeated in triplicate.

The development of the routes for decalin synthesis and related Diels-Alder outcomes:

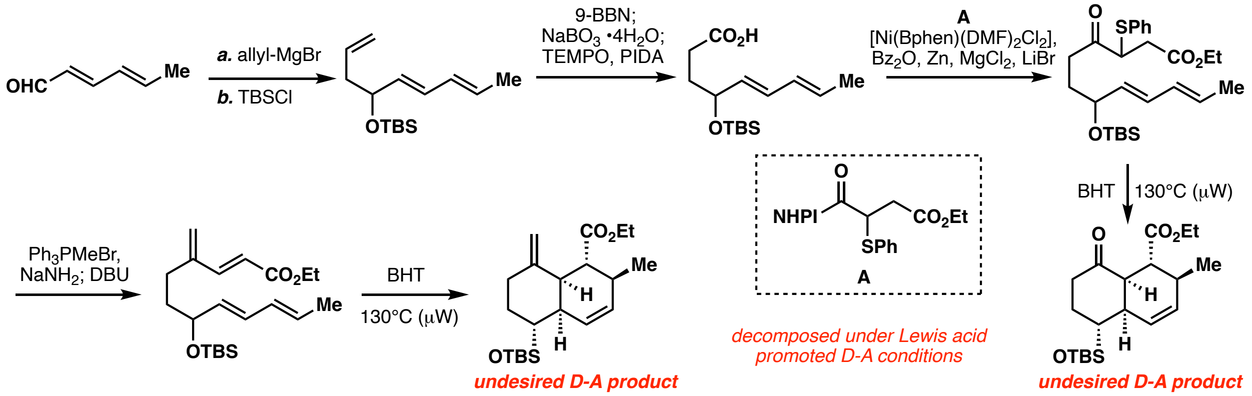

Scheme S4. The failed 1st generation route

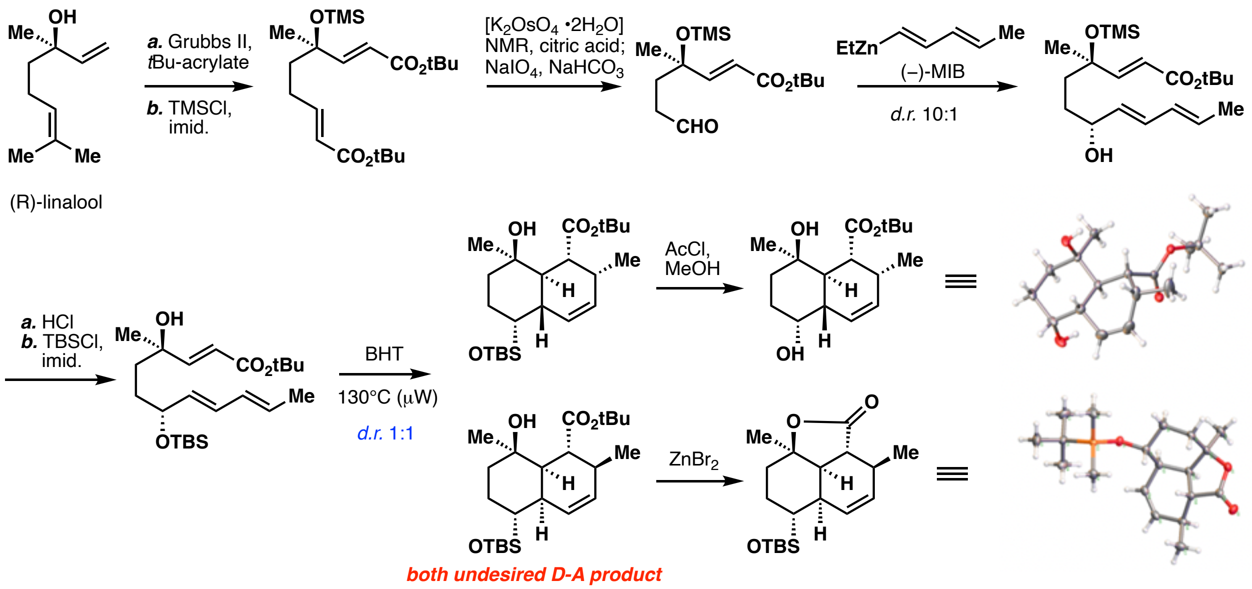

Scheme S5. The failed 2nd generation route starts from (R)-linalool

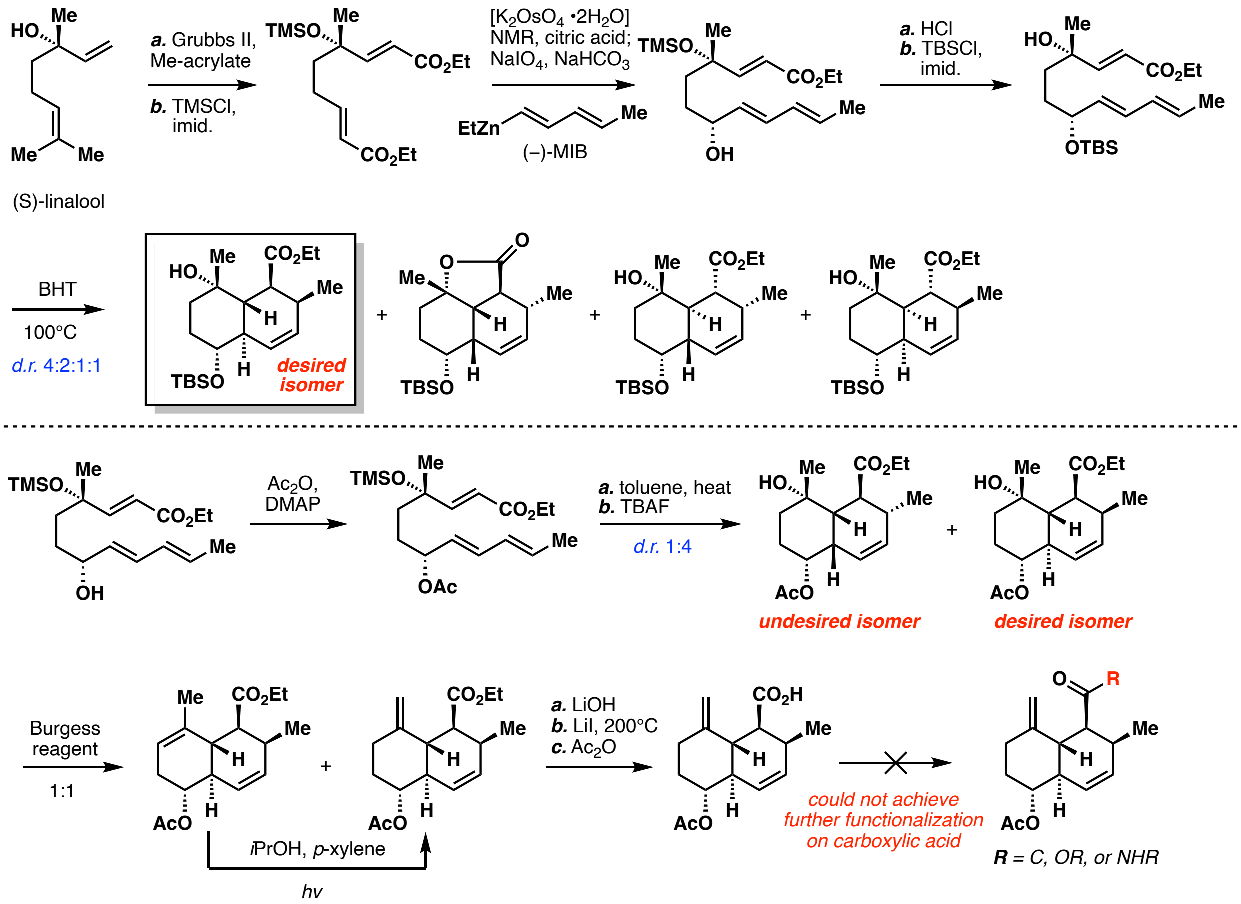

Scheme S6. The failed further functionalization on the 2nd generation D-A product

Other deadend intermediates:

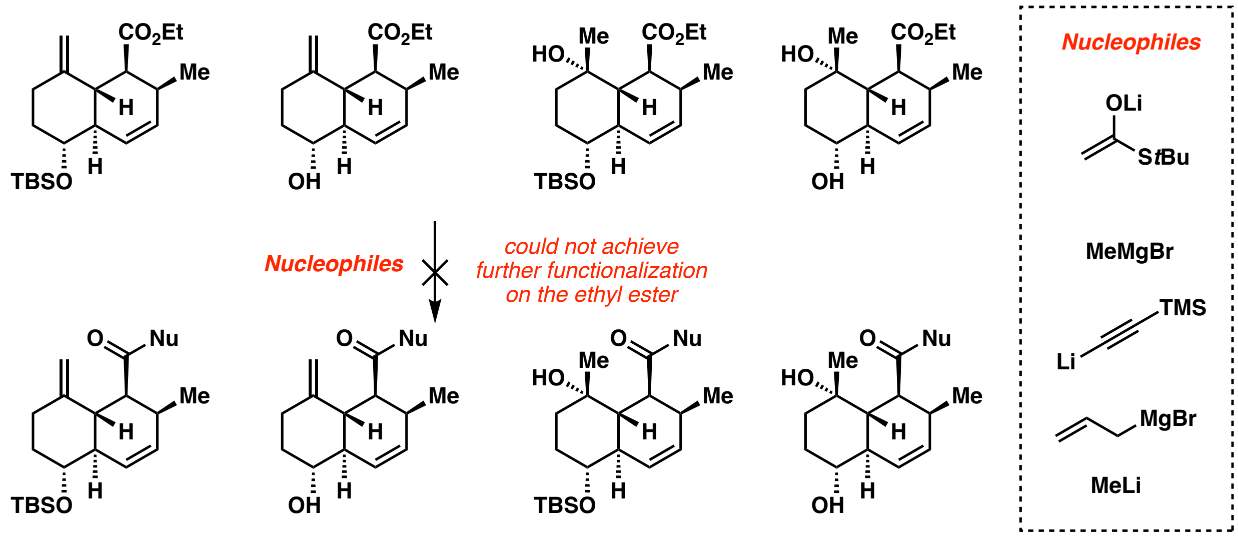

Scheme S7. Other dead-end intermediates in the 2nd generation route

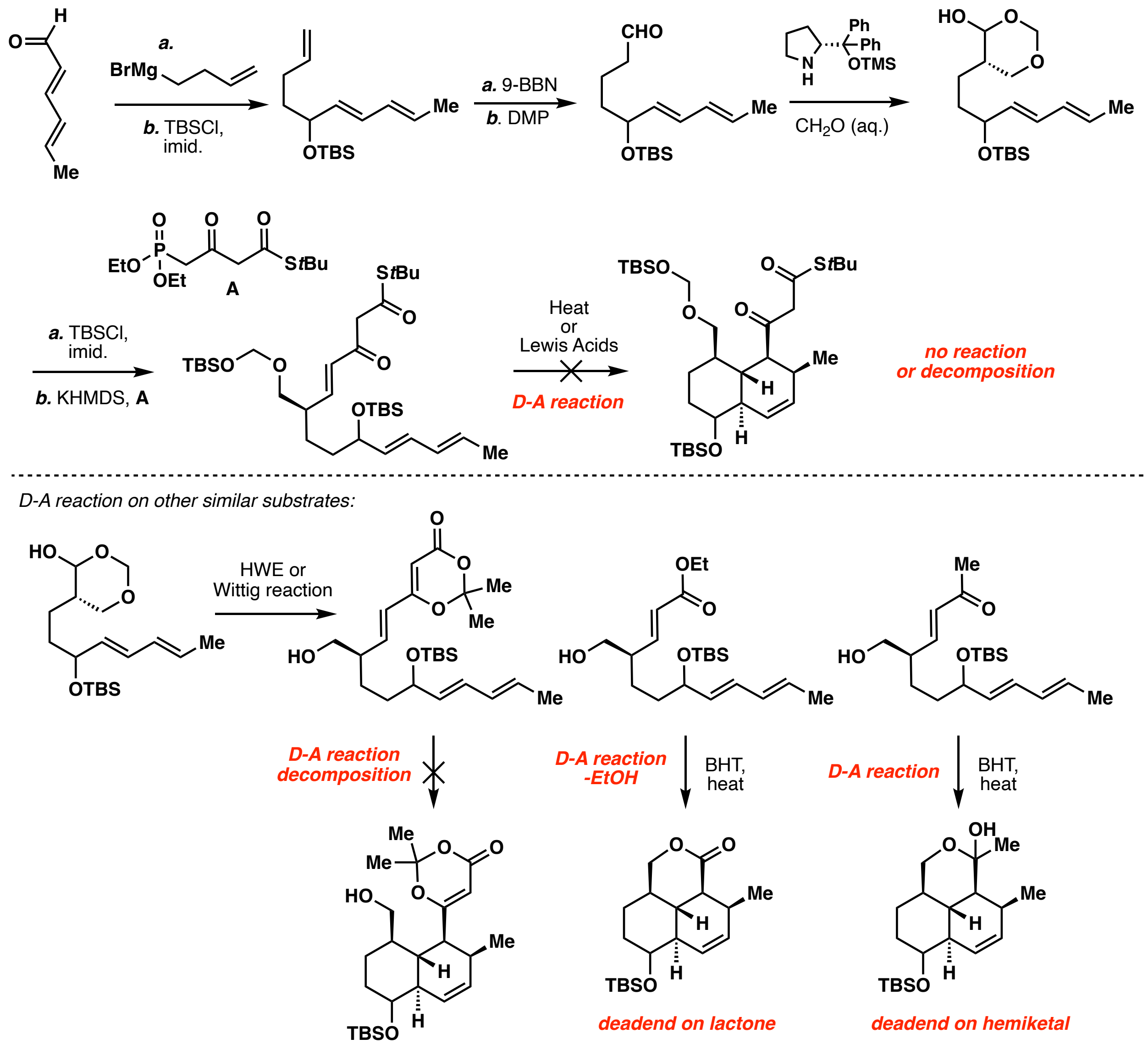

Scheme S8. The 3rd generation route and the related intermediates for D-A reaction

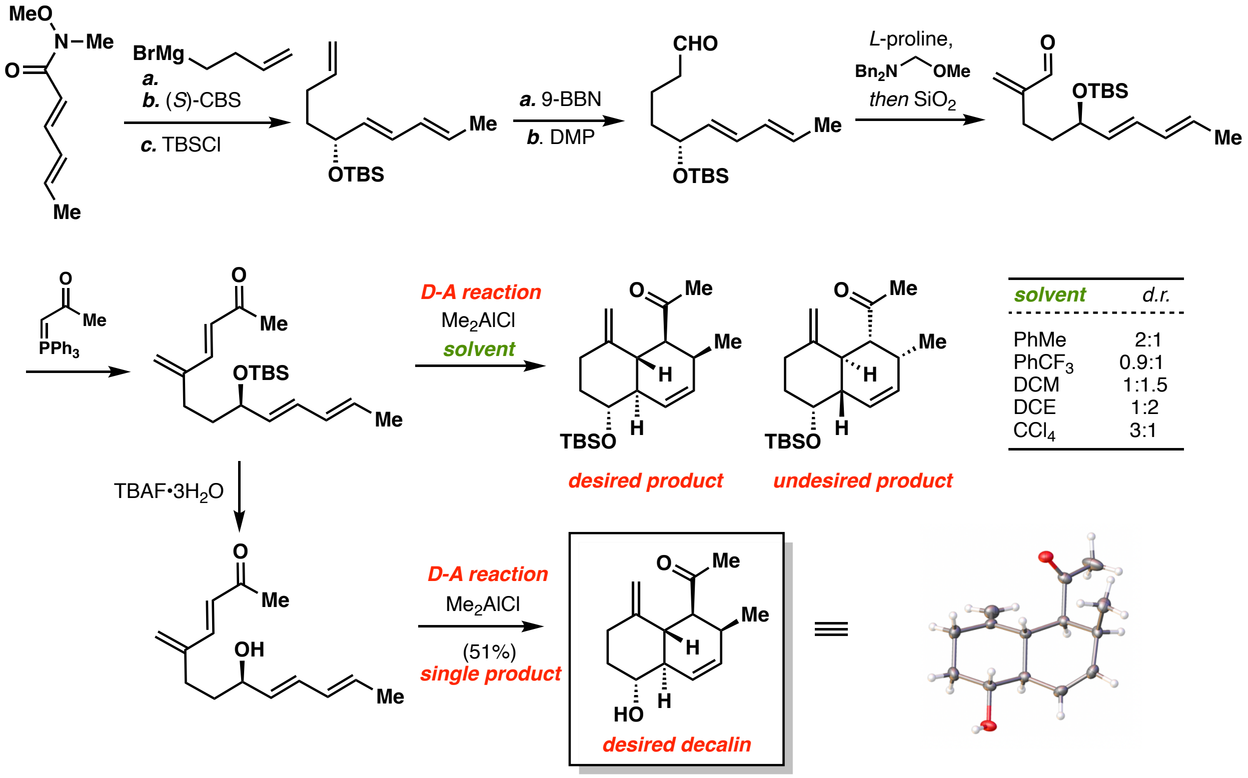

solvent	d.r.
PhMe	2:1
$PhCF_3$	0.9:1
DCM	1:1.5
DCE	1:2
CCl_4	3:1

Scheme S9. The final route for decalin

The *cis*-diol construction through reduction/hydration sequence:

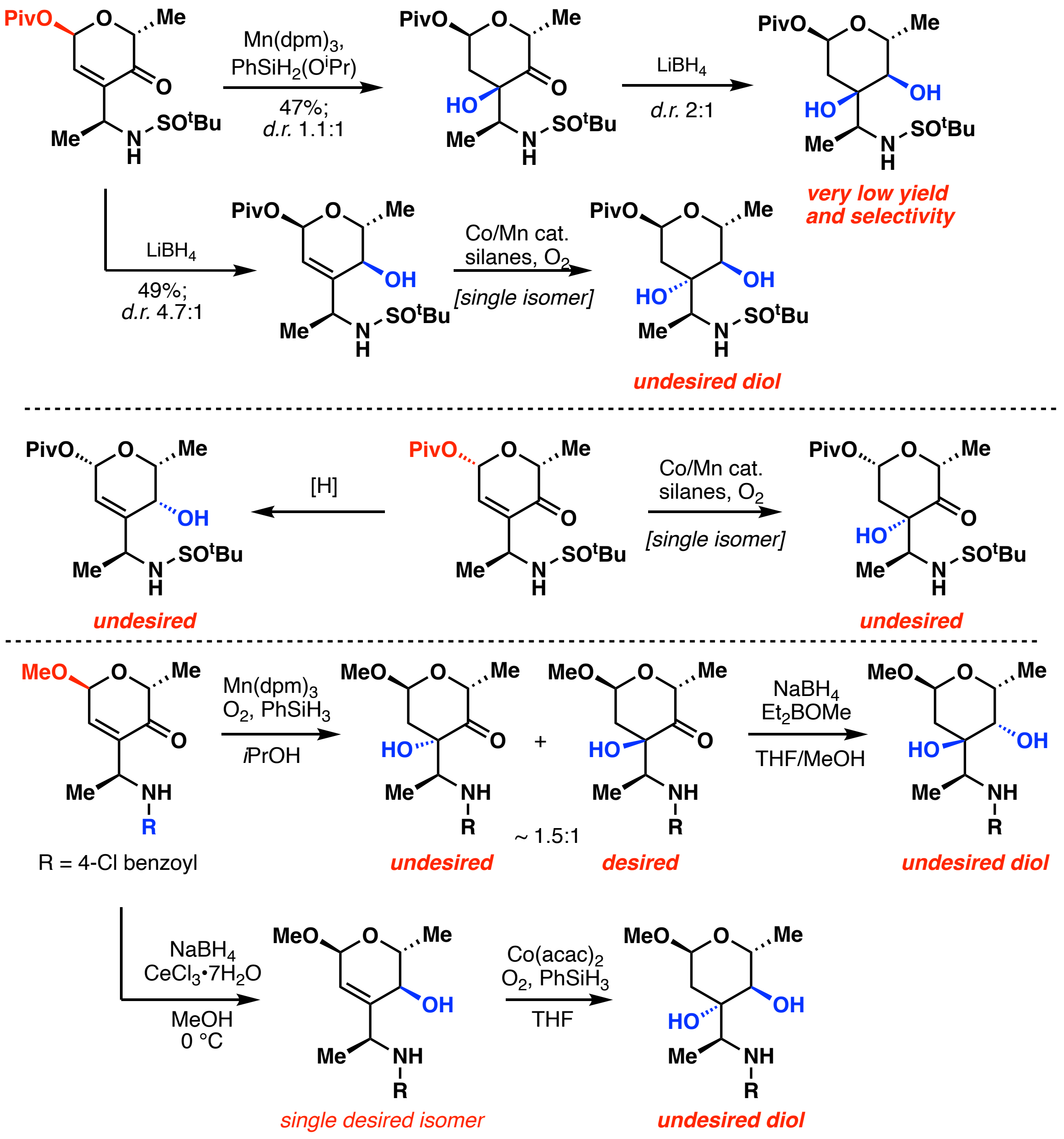

Scheme S10. The poor selectivity of the reduction/hydration strategy

The efforts on the *O*-glycosidation:

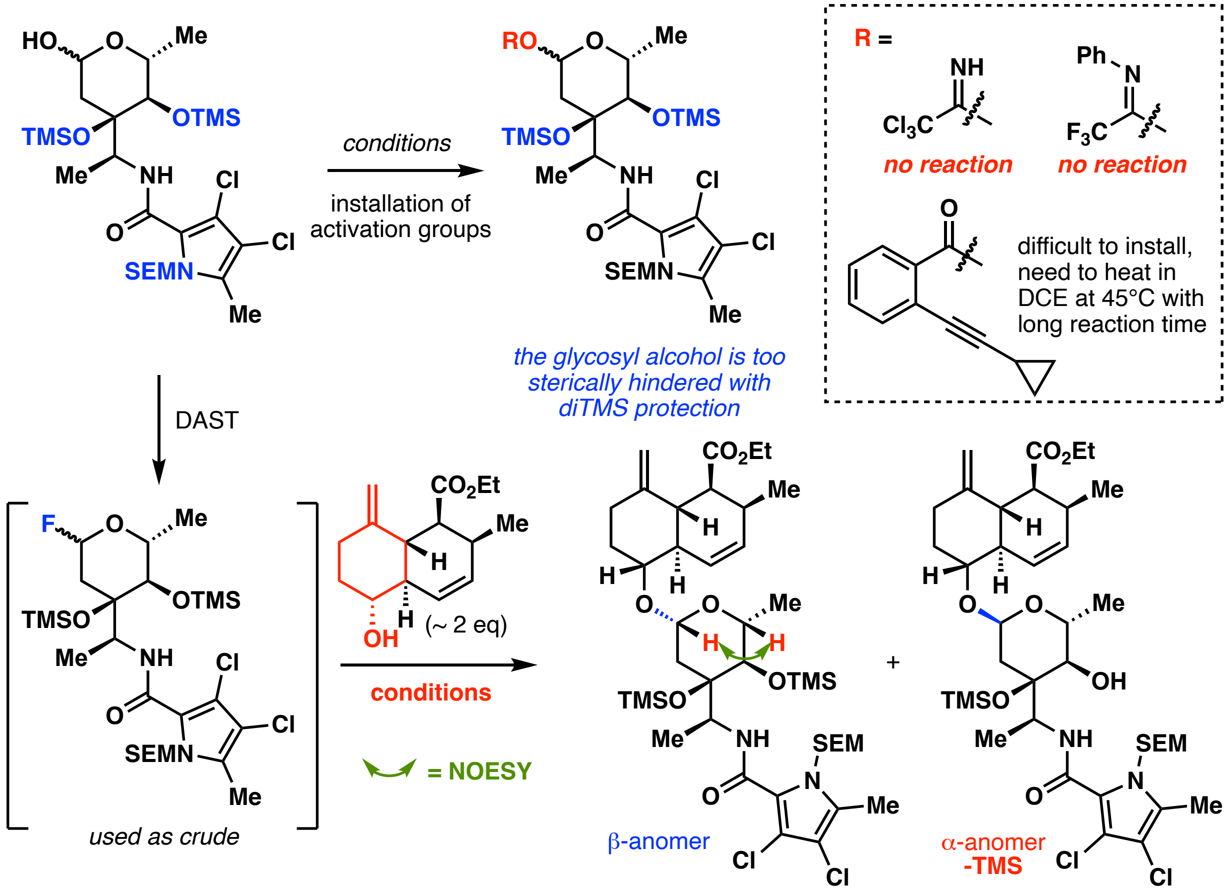

conditions	results
1) BF_3•Et_2O	no reaction, glycosyl fluoride decomposed
2) TMSOTf	decomposed in 10 min
3) $SnCl_2$, $AgClO_4$, Et_2O	50% yield, β/α 1:2
4) Cp_2HfCl_2, $AgClO_4$, DCM	75% yield, β/α 1:1
5) Cp2HfCl2, AgClO4, MeCN	no reaction, glycosyl fluoride slowly decomposed
6) Cp_2ZrCl_2, $AgClO_4$, DCM	unknown product

Scheme S11. The *O*-glycosidation through glycosyl fluoride

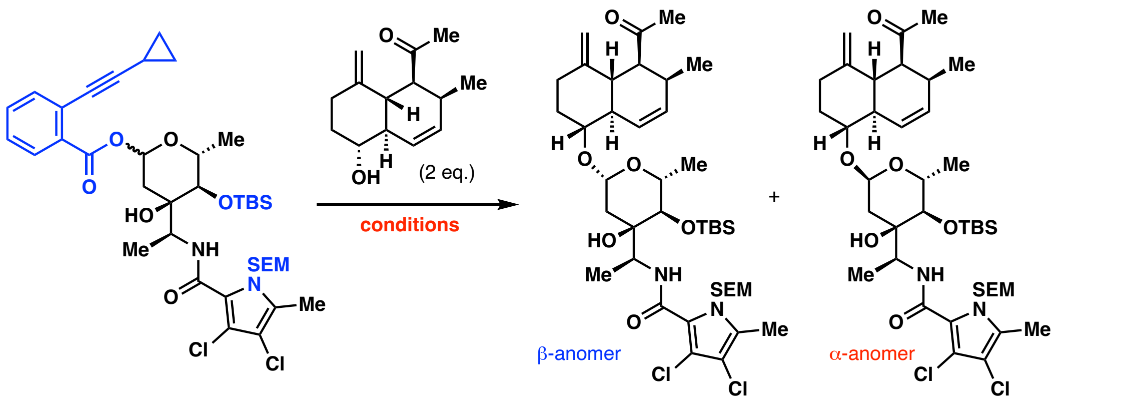

conditions	results (β:α)	conditions	results (β:α)
AgOTf, 4 A MS, DCM, rt	2:1	AgOTf, 4 A MS, $CHCl_3$ or EtOAc or Et_2O, rt	no β anomer
AgOTf, 4 A MS, DCM, rt, c = 0.0025M	1.6:1	AgOTf, 3 A MS, DCM, rt	1.2:1
AgOTf, 4 A MS, DCM/toluene, rt	0.6:1	I_2, 3 A MS, DCM, rt	0.2:1
AgOTf, $Gd(OTf)_3$, 4 A MS, DCM, rt	1.1:1	$Hg(OAc)_2$, 4 A MS, DCM, rt	no pdt
AgOTf, 4 A MS, DCM, 0 °C	1.3:1	$Hg(OAc)_2$, 4 A MS, DCM/CH_3CN, rt	1.5:1
Ag_2O or $AgNO_3$ or Ag_2SO_4, 4 A MS, DCM, rt	NR	$Hg(OTf)_2$, 4 A MS, DCM, rt[a]	2.4:1
$AgClO_4$, 4 A MS, DCM, rt	2:1	$Hg(OTf)_2$, $In(OTf)_3$, 4 A MS, DCM, rt	2:1
$AgClO_4$, 4 A MS, DCM, rt	2:1	$Hg(OTf)_2$, 4 A MS, DCM, -20 °C to rt	low conv.
$AgClO_4$, 4 A MS, DMSO, rt	NR	$Hg(OTf)_2$, 4 A MS, CH_3CN, rt	complex, little β anomer
$AgSbF_6$, 4 A MS, DCM, rt	no pdt	$Ph_3PAuNTf_2$, 4 A MS, DCM, -40 °C to rt	0.12:1
AgOTf, 4 A MS, toluene, rt	0.3:1	$SPhosAuNTf_2$, 4 A MS, DCM, -40 °C	low conv.
AgOTf, 4 A MS, DCE, rt	1.5:1	$Pt(NH_3)_2Cl_2$ or K_2PtCl_4, 4 A MS, DCE, rt	NR

[a] 200 mg scale, β: 53%, α: 20%.

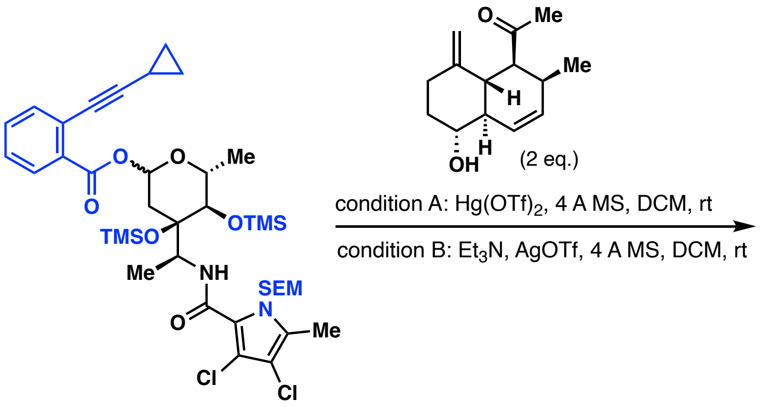

result A: single β isomer,
but TMS was unstable
and the reaction mixture was complex

result B: the material balance was better,
but β/α was only 0.9:1

Scheme S12. The *O*-glycosidation with alkynyl benzoic ester as the activating group

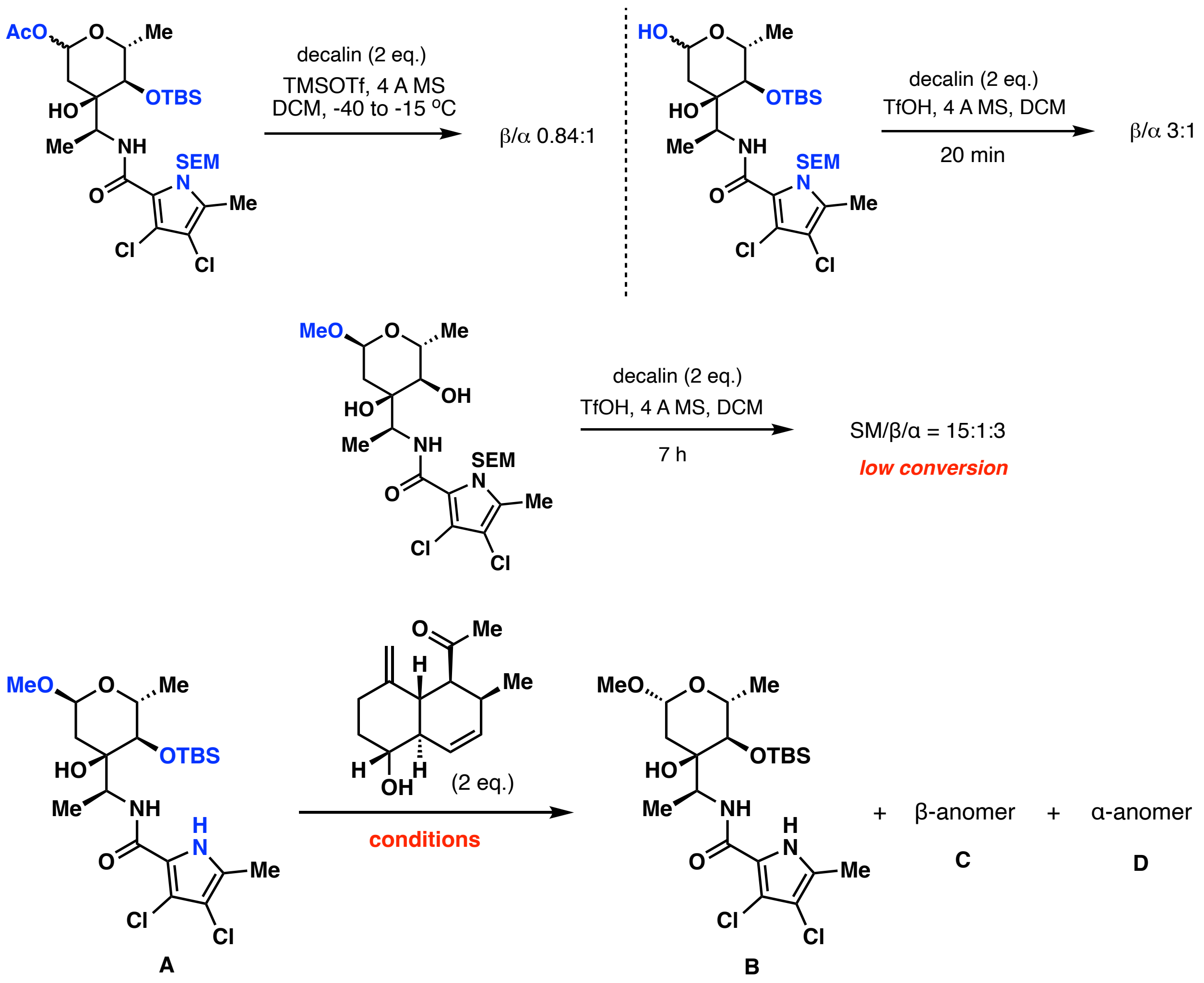

conditions	result
TfOH, DCM	low conversion
TfOH, DCM, TMSCl	low conversion
TfOH, DCM, TBSOTf	low conversion
Tf_2NH, DCM	low conversion
BF_3•Et_2O, DCM	A: 25%, B: 20%, C: 15%, D: 15%

Scheme S13. The poor selectivity of *O*-glycosidation with other activating groups

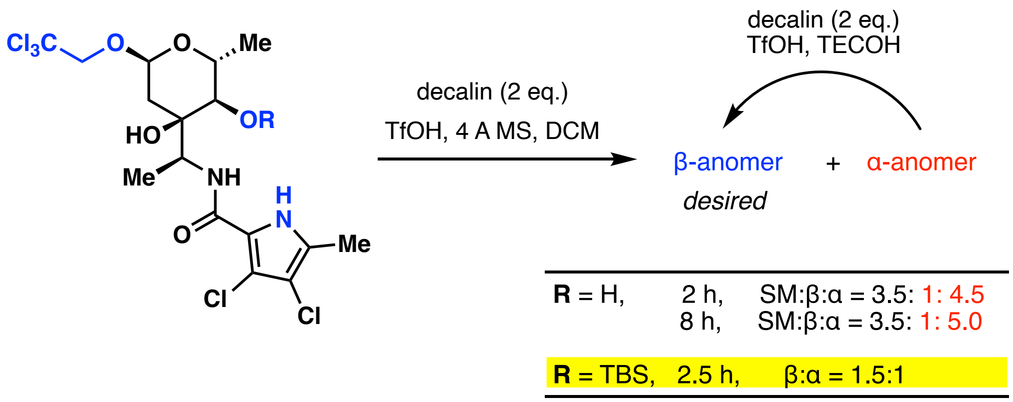

Scheme S14. The final *O*-glycosidation substrate with mono TBS protection and TCE as the activating group achieves the good conversion and equilibrium

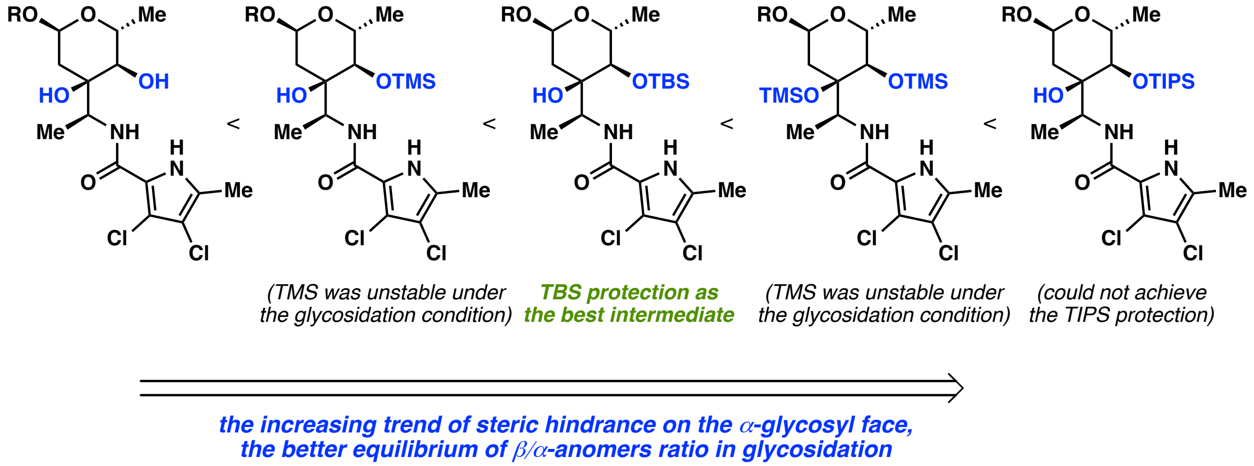

Scheme S15. The control of *O*-glycosidation selectivity based on the steric hindrance of the substrate

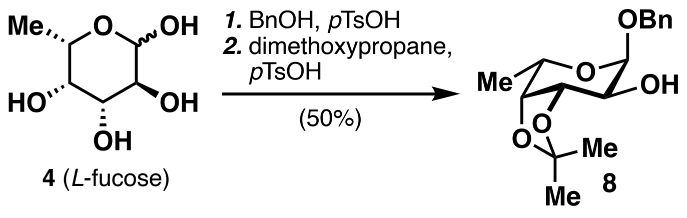

Experimental: To a flame-dried round bottom flask fixed with a stirbar, was added *L*-fucose (4.105 g, 25.00 mmol). The flask was placed under argon and BnOH (10.0 mL, 10.0 eq.) was added via syringe. The mixture was stirred until all solids were dissolved at which point *p*-TsOH (4.305 g, 2.5 mmol, 0.10 eq.) was added, and the flask was quickly resealed. The solution was then placed on an oil bath preheated to 80 °C and was allowed to stir at this temperature overnight. Upon completion, the flask was removed from the oil bath and allowed to cool. The crude reaction mixture was then loaded directly onto a column and purified by flash column chromatography (SiO_2; DCM/MeOH 20:1 to 10:1) and used directly in the next reaction.

The benzylated fucopyranose was placed in a round bottom flask fixed with a stirbar and placed under argon. DMF was added (70 mL, 0.36M) and stirred until the solids were dissolved. *p*-TsOH (0.861 g, 5.00 mmol, 0.20 eq.) was added, and once all matierals were dissolved, 2,2-dimethoxypropane (9.20 mL, 75.00 mmol, 3.0 eq.) was added dropwise. The resulting solution was stirred overnight at room temperature. When complete, the DMF was removed by rotary evaporation. The resulting residue was re-dissolved in chloroform (70.0 mL). This solution was rinsed sequentially with $NaHCO_3$ (*sat. aq.*), 5% LiCl (*aq.*), and brine. The organic layer was dried over $MgSO_4$, filtered, and concentrated under reduced pressure. The desired product was purified by flash column chromatography (SiO_2; 30% EtOAc in hexanes) yielding **8** (3.62 g, 50% yield).

NOTE: *Both α and β anomers result. In the above eluent, the alpha anomer has a higher R_f (α = 0.30; β = 0.25). The beta anomer provided unsatisfactory diastereoselectivity in subsequent reactions and thus was discarded.*

Physical State: colorless oil;

R_f Value: 0.30 (30% EtOAc in hexanes);

^{1}H NMR (600 MHz, C_6D_6) δ 7.21 – 7.18 (m, 2H), 7.16 – 7.13 (m, 2H), 7.11 – 7.07 (m, 1H), 4.88 (d, *J* = 3.9 Hz, 1H), 4.60 (d, *J* = 11.9 Hz, 1H), 4.30 (d, *J* = 12.0 Hz, 1H), 4.25 (t, *J* = 6.2 Hz, 1H), 4.04 (dq, *J* = 2.3, 6.6 Hz, 1H), 3.95 (m, 1H), 3.72 (dd, *J* = 6.3, 2.3 Hz, 1H), 2.24 (d, *J* = 5.5 Hz, 1H), 1.47 (s, 3H), 1.33 (d, *J* = 6.6 Hz, 3H), 1.23 (s, 3H), 0.59 ppm (s, 1H);

^{13}C NMR (151 MHz, C_6D_6) δ 138.2, 128.7 (2C), 238.4, 128.0 (2C), 109.0, 97.2, 76.3, 75.9, 69.6, 69.4, 64.8, 27.8, 25.9, 16.6 ppm;

Optical Rotation: $[\alpha]_D^{20}$ = -126.0° (c = 0.50 g / 100 mL, $CHCl_3$);

HRMS (m/z): calc'd for $C_{16}H_{22}O_5Na^+$ $[M+Na]^+$ 317.1359, found 317.1364.

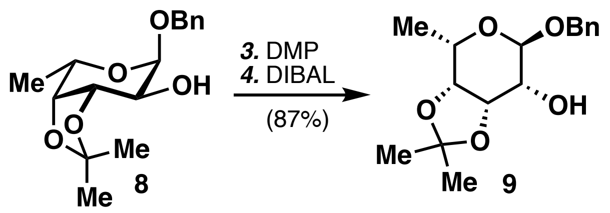

Experimental: To a flame-dried round bottom flask fixed with a stir bar, was added **8** (3.62 g, 12.3 mmol) and CH_2Cl_2 (120 mL, 0.1M). Dess-Martin-Periodinane (9.13 g, 21.5 mmol, 1.75 eq.) was added in one portion and the reaction mixture was stirred for 2 h at room temperature. The reaction mixture was concentrated under reduced pressure. The crude solids were re-dissolved in Et_2O (0.1M) and run through a plug of silica. The silica plug was rinsed with three more volumes of Et_2O, and the resulting eluent was concentrated under reduced pressure. Finally, the resulting residue was azeotroped with toluene twice on a rotary evaporator (in order to remove any residual acetic acid) yielding a white solid that was used directly in the next reaction.

The above ketone (3.62 g, 12.3 mmol) was dissolved in THF (120 mL, 0.1M) under argon, and the solution was cooled to –78 °C. DIBAL-H (1.0 M in hexanes; 30.75 mL, 30.75 mmol, 2.5 eq.) was then added dropwise. After stirring the reaction mixture at –78 °C for 2 h and overnight at room temperature, saturated NH_4Cl (*aq.*) (35 mL) was added. Next, a saturated aqueous solution of Rochelle's salt (35 mL) and EtOAc (35 mL) were added, and the mixture was stirred vigorously for 30 min. The aqueous phase was extracted with EtOAc (3 x 50 mL). The combined organic layers were rinsed with brine, dried over anhydrous Na_2SO_4, and filtered. The solvent was removed under reduced pressure. The crude product was purified *via* flash column chromatography (SiO_2; 30% EtOAc in hexanes) yielding **9** (3.15 g; 87% yield) as a colorless oil.

Physical State: colorless oil;

R_f Value: 0.30 (30% EtOAc in hexanes);

^{1}H NMR (600 MHz, C_6D_6) δ 7.32 – 7.29 (m, 2H), 7.18 – 7.15 (m, 2H), 7.11 – 7.07 (m, 1H), 4.90 (d, *J* = 5.4 Hz, 1H), 4.73 (d, *J* = 12.1 Hz, 1H), 4.44 (d, *J* = 12.1 Hz, 1H), 4.16 (dd, *J*

= 7.3, 3.4 Hz, 1H), 3.63 – 3.60 (m, 1H), 3.48 (m, 2H), 2.16 (s, 1H), 1.51 (s, 3H), 1.22 (d, J = 6.4 Hz, 3H), 1.20 ppm (s, 3H);

^{13}C NMR (151 MHz, C_6D_6) δ 138.9, 128.6 (2C), 128.0 (2C), 127.7, 109.8, 101.0, 76.5, 74.5, 69.5, 69.3, 65.4, 26.4, 25.5, 16.1 ppm;

Optical Rotation: $[\alpha]_D^{20}$ = -94.4° (c = 0.50 g / 100 mL, $CHCl_3$);

HRMS (m/z): calc'd for $C_{16}H_{22}O_5Na^+$ $[M+Na]^+$ 317.1359, found 317.1364.

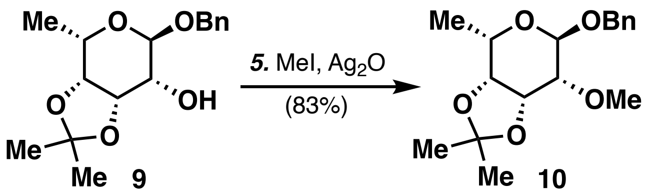

Experimental: To a flame-dried round bottom flask fixed with a stir bar, was added the sugar alcohol **9** (2.22 g, 7.5 mmol) and Ag_2O (3.15 g, 22.5 mmol, 3.0 eq.). The flask was placed under argon and MeCN (75 mL, 0.1M) was added. Next, methyl iodide (2.8 mL, 24.0 mmol, 6.0 eq.) was added via syringe, and the resulting mixture was heated on an oil bath to 75 °C. After stirring overnight at this temperature, the reaction was allowed to cool, then was filtered through celite ($CHCl_3$). The solvent was removed under reduced pressure and the crude product was purified *via* flash column chromatography (SiO_2; 30% Et_2O in hexanes) yielding **10** (1.92 g, 83% yield).

Physical State: colorless oil;

R_f Value: 0.30 (30% Et_2O in hexanes);

^{1}H NMR (600 MHz, C_6D_6) δ 7.32 – 7.29 (m, 2H), 7.17 – 7.15 (m, 2H), 7.10 – 7.06 (m, 1H), 5.14 (d, J = 5.9 Hz, 1H), 4.78 (d, J = 12.0 Hz, 1H), 4.45 (d, J = 12.0 Hz, 1H), 4.32 (dd, J = 7.5, 2.7 Hz, 1H), 3.57 (dd, J = 7.6, 2.0 Hz, 1H), 3.50 (dq, J = 2.0, 6.4 Hz, 1H), 3.33 (s, 3H), 3.15 (dd, J = 5.9, 2.7 Hz, 1H), 1.56 (s, 3H), 1.27 (s, 3H), 1.24 ppm (d, J = 6.4 Hz, 3H);

^{13}C NMR (151 MHz, C_6D_6) δ 139.3, 128.5 (2C), 128.0 (2C), 127.6, 110.1, 100.4, 78.9, 77.1, 72.8, 69.4, 66.0, 58.1, 26.6, 25.7, 15.8 ppm;

Optical Rotation: $[\alpha]_D^{20}$ = -70.6° (c = 0.50 g / 100 mL, $CHCl_3$);

HRMS (m/z): calc'd for $C_{17}H_{24}O_5Na^+$ $[M+Na]^+$ 331.1516, found 331.1520.

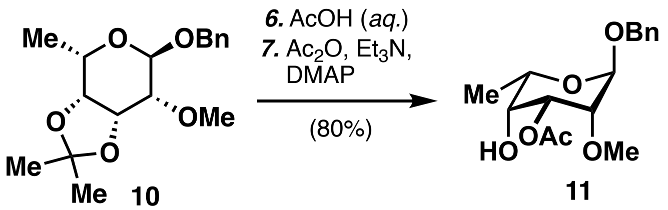

Experimental: The methylated product **10** (1.90 g, 6.17 mmol) was placed in a round bottom flask fixed with a stir bar. To this was added an aqueous solution of acetic acid (80% AcOH (v/v), 62 mL, 0.1M). The resulting solution was placed on an oil bath preheated to 80 °C. The reaction was monitored by TLC and upon completion (about 1 h), the solvent was removed under reduced pressure. Trace amounts of water and acetic acid were removed by azeotrope with toluene (2 x 30 mL). The resulting diol was directly used in the next step.

The crude diol was placed in a round bottom flask fixed with a stir bar and placed under argon. CH_2Cl_2 (62.0 mL, 0.1M) and Et_3N (0.95 mL, 6.80 mmol, 1.1 eq.) were added via syringe. Next, DMAP (0.0378 g, 0.31 mmol, 0.05 eq.) was added to the flask followed by the dropwise addition of Ac_2O (6.16 mL, 6.51 mmol, 1.05 eq.). The reaction was stirred overnight at room temperature. Upon completion, the reaction was quenched with NH_4Cl *(sat., aq.)*. The aqueous phase was extracted with Et_2O (3x). The combined organic layers were rinsed with brine, dried over $MgSO_4$, and filtered. The solvent was removed under reduced pressure. The crude material was purified by flash column chromatography (SiO_2; 30% to 40% EtOAc in hexanes) to yield **11** (1.53 g, 80% yield).

Physical State: colorless oil;

R_f Value: 0.30 (40% EtOAc in hexanes);

^{1}H NMR (600 MHz, $CDCl_3$) δ 7.38 – 7.29 (m, 5H), 5.08 (t, *J* = 3.2 Hz, 1H), 5.01 (d, *J* = 1.7 Hz, 1H), 4.73 (d, *J* = 11.9 Hz, 1H), 4.55 (d, *J* = 11.9 Hz, 1H), 3.95 (q *J* = 6.6 Hz, 1H), 3.70 (s, 1H), 3.57 (m, 1H), 3.49 (s, 3H), 2.16 (s, 3H), 1.28 ppm (d, *J* = 6.5 Hz, 3H);

^{13}C NMR (151 MHz, $CDCl_3$) δ 170.5, 137.1, 128.6 (2C), 128.1 (3C), 96.9, 78.7, 71.0, 69.9, 69.5, 67.7, 59.8, 21.3, 16.4 ppm;

Optical Rotation: $[\alpha]_D^{20}$ = -113.6° (c = 0.50 g / 100 mL, $CHCl_3$);

HRMS (m/z): calc'd for $C_{16}H_{22}O_6Na^+$ $[M+Na]^+$ 333.1309, found 333.1308.

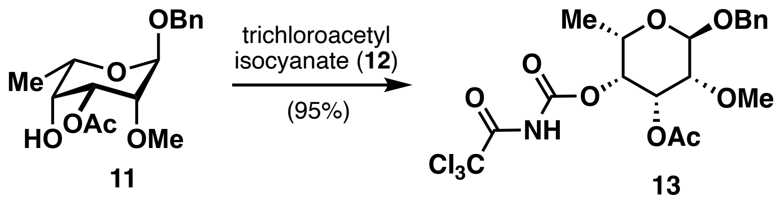

Experimental: To the starting material **11** (205 mg, 0.661 mmol) was added DCM (6.6 mL), and the resulting solution was cooled to 0°C. Trichloroacetyl isocyanate **12** (0.16 mL, 1.33 mmol, 2.0 eq.) was added dropwise *via* syringe and the resulting mixture was stirred at 0°C for 30 min followed by another 30 min at room temperature. Upon completion (as determined by TLC; product R_f = 0.30 in hexanes/EtOAc 2:1), the reaction was quenched by MeOH (0.5 mL) and stirred for 10 minutes. All solvent was removed under reduced pressure, and the resulting material was purified by flash column chromatography (SiO_2; hexanes/EtOAc 3:1 to 2:1) to yield **13** (312 mg, 95% yield).

Physical State: white foam;
R_f Value: 0.30 (hexanes/EtOAc 2:1);
^{1}H NMR (600 MHz, $CDCl_3$) δ 8.95 (s, 1H), 7.40 – 7.29 (m, 5H), 5.27 (t, *J* = 3.5 Hz, 1H), 5.18 (m, 1H), 5.01 (d, *J* = 1.6 Hz, 1H), 4.71 (d, *J* = 11.9 Hz, 1H), 4.56 (d, *J* = 11.9 Hz, 1H), 4.16 (qd, *J* = 6.5, 1.6 Hz, 1H), 3.49 – 3.47 (m, 1H), 3.47 (s, 3H), 2.10 (s, 3H), 1.25 ppm (d, *J* = 6.6 Hz, 3H);
^{13}C NMR (151 MHz, $CDCl_3$) δ 170.1, 158.2, 149.8, 136.8, 128.7 (2C), 128.3, 128.2 (2C), 97.8, 91.9, 76.8, 72.9, 69.8, 68.3, 64.8, 60.2, 21.0, 16.2 ppm;
Optical Rotation: $[\alpha]_D^{25}$ = -82.2° (c = 1.00 g / 100 mL, $CHCl_3$);
HRMS (m/z): calc'd for $C_{19}H_{23}Cl_3NO_8^+$ $[M+H]^+$ 498.0484, found 498.0489.

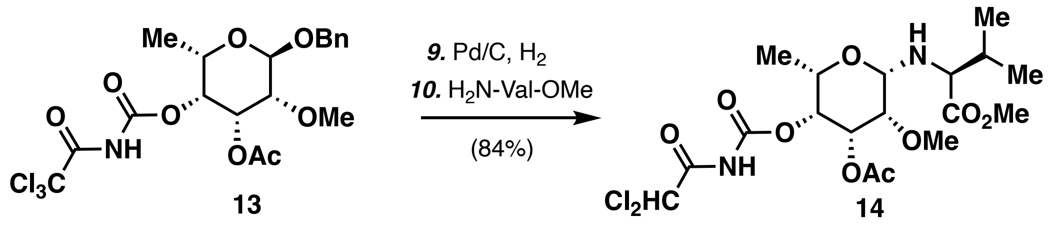

Experimental: To the starting material **13** (325 mg, 0.654 mmol) in EtOAc (10 mL), was added Pd/C (10% Pd/wt; 33 mg, 10 wt/wt %). The flask was evacuated and backfilled with H_2 (in a balloon) three times. Finally, the flask was fitted with a balloon of H_2 and the reaction mixture was vigorously stirred at room temperature for 3 h until complete as determined by TLC. Upon completion, the reaction mixture was filtered through a pad of celite (EtOAc) and the solvent was removed under reduced pressure yielding the product as a white foam, which was used directly in the next step with no further purification.

The PPTS (32.8 mg, 0.13 mmol, 0.2 eq) and *L*-valine methyl ester (154 mg, 1.17 mmol, 1.8 eq) were added to 5 mL of DCM, and the mixture was stirred for 10 min before adding to another flask of the dried crude material above. The reaction was stirred at room temperature for 6 h and quenched with excess solid $NaHCO_3$. The mixture was filtered (washed with EtOAc), and the solvent was removed under reduced pressure. The crude material was purified by flash column chromatography (SiO_2; hexanes/acetone 8:1) to yield **14** (267 mg, 84% yield over 2 steps).

Note: *The product is almost a single β-isomer. The very trace amount of α-isomer was removed by the silica gel chromatography.*

Physical State: white foam;
R_f Value: 0.40 (hexanes/acetone 2:1);
^{1}H NMR (600 MHz, Acetone-*d6*) δ 10.59 (s, 1H), 6.97 (s, 1H), 5.02 (t, *J* = 3.6 Hz, 1H), 4.98 (m, 1H), 4.17 (dd, *J* = 12.7, 1.4 Hz, 1H), 3.76 (dd, *J* = 6.5, 1.5 Hz, 1H), 3.68 (s, 3H), 3.51 (dd, *J* = 3.5, 1.3 Hz, 1H), 3.49 (s, 3H), 3.09 (dd, *J* = 9.8, 6.8 Hz, 1H), 2.92 (dd, *J* = 12.6, 9.7 Hz, 1H), 2.02 (s, 3H), 1.82 – 1.73 (m, 1H), 1.08 (d, *J* = 6.4 Hz, 3H), 0.94 (d, *J* = 6.7 Hz, 4H), 0.89 ppm (d, *J* = 6.8 Hz, 3H);

^{13}C NMR (151 MHz, Acetone-d6) δ 176.4, 170.1, 164.0, 152.2, 89.4, 78.2, 73.0, 72.2, 70.3, 67.1, 65.2, 61.6, 51.6, 33.0, 20.9, 19.3, 18.8, 16.4 ppm;

Optical Rotation: $[\alpha]_D^{25}$ = -23.5° (c = 0.95 g / 100 mL, $CHCl_3$);

HRMS (m/z): calc'd for $C_{18}H_{29}Cl_2N_2O_9{}^+$ [M+H]$^+$ 487.1245, found 487.1241.

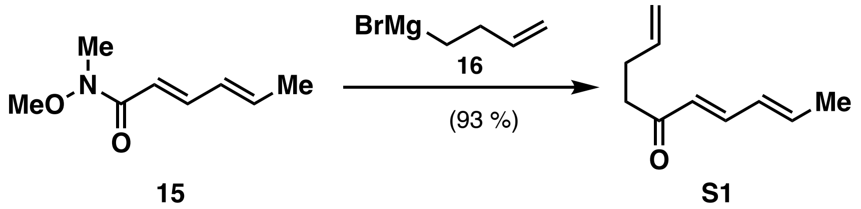

Experimental: An oven-dried round bottom flask was placed under argon and 210 mL of THF was added to it followed by 4-bromobutene (15.0 mL, 150.0 mmol). A separate oven-dried two-neck round bottom flask was fitted with a stir bar. To this flask was added magnesium turnings (5.40 g, 150 mmol, 1.0 eq.) and I_2 (381 mg, 1.50 mmol, 0.01 eq.) under argon. About 9 mL of the 4-bromobutene solution was added to the iodine and magnesium turnings *via* syringe, and the resulting mixture was heated with a heat gun while being stirred until the brown color disappeared completely. At this point, the entirety of the remaining 4-bromobutene solution was cannulated into the 2-neck flask while occasionally heating the solution to near-reflux with a heat gun. Upon complete addition, the resulting mixture was allowed to stir at room temperature for 1 h. The resulting Grignard reagent **16** was then titrated using the method outlined in the supporting information of the former publication.[1]

Afterward, Weinreb amide **15** (9.31 g, 60 mmol) was placed in a round bottom flask fixed with a stir bar. THF (350.0 mL) was added *via* syringe and stirring was started. Cooled the solution down to 0 °C and added the Grignard reagent **16** (1.4 eq.) slowly into the reaction mixture. After the addition of the Grignard reagent, the mixture was stirred for another 4 h. The reaction was quenched with NH_4Cl (*sat., aq.*, 200 mL), and the aqueous layer was extracted with Et_2O (3 x 200 mL). The combined organic layers were washed with brine, dried with $MgSO_4$, and filtered. The solvent was removed under reduced pressure and the crude material was purified by flash column chromatography (SiO_2; 0% to 10% Et_2O in hexanes), yielding the product **S1** (8.38 g, 93% yield).

Physical State: colorless oil;

TLC: R_f = 0.50 (30% Et_2O in hexanes);

^{1}H NMR (400 MHz, $CDCl_3$) δ 7.22 – 6.98 (m, 1H), 6.33 – 6.16 (m, 2H), 6.13 – 6.01 (m, 1H), 5.93 – 5.76 (m, 1H), 5.16 – 4.84 (m, 2H), 2.78 – 2.56 (m, 2H), 2.48 – 2.30 (m, 2H), 1.92 – 1.76 ppm (m, 3H);

^{13}C NMR (151 MHz, $CDCl_3$) δ 200.1, 143.1, 140.5, 137.5, 130.5, 127.8, 115.3, 39.7, 28.4, 19.0 ppm;

HRMS (m/z): calc'd for, $C_{10}H_{15}O^+$ $[M+H]^+$ 151.1117, found 151.1122.

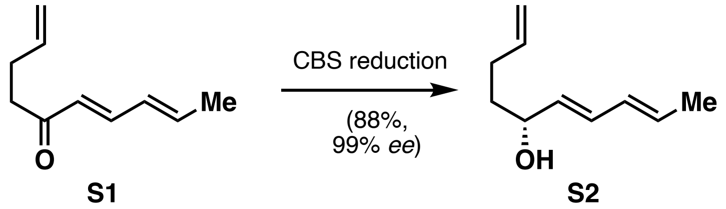

Experimental: The (*S*)-CBS catalyst (27.72 g, 2.0 eq) was placed in a round bottom flask fixed with a stir bar and flushed with argon 3 times. THF (250.0 mL) was added *via* syringe and stirring was started. The resulting solution was cooled down to 0 °C and BH_3/THF complex solution (1.0 M in THF, 80 ml, 1.6 eq.) was added into the mixture slowly. After 20 mins, the solution was cooled down to -78 °C and a THF solution (30 ml) of ketone **S1** (7.51 g, 1.0 eq.) was added *via* a syringe pump over 45 min. 1 h after the addition of ketone **S1**, another 0.6 eq. of BH_3/THF complex solution (1.0 M in THF, 30 ml) was added to the mixture slowly. 4 h later, the reaction was quenched with NH_4Cl (*sat., aq.*, 200 mL), and the aqueous layer was extracted Et_2O (3 x 300 mL). The combined organic layers were washed with brine and dried over $MgSO_4$. The solvent was removed under reduced pressure and the crude material was purified by flash column chromatography (SiO_2; 0% to 50% Et_2O in hexanes), yielding the product **S2** (6.69 g, 88% yield).

Physical State: colorless oil;

TLC: R_f = 0.30 (40% Et_2O in hexanes);

^{1}H NMR (600 MHz, $CDCl_3$) δ 6.19 (dd, *J* = 15.2, 10.4 Hz, 1H), 6.10 – 5.98 (m, 1H), 5.85 (ddt, *J* = 16.9, 10.2, 6.7 Hz, 1H), 5.79 – 5.65 (m, 1H), 5.61 – 5.50 (m, 1H), 5.06 (dq, *J* = 17.1, 1.7 Hz, 1H), 4.99 (ddt, *J* = 10.2, 2.2, 1.3 Hz, 1H), 4.16 (q, *J* = 6.7 Hz, 1H), 2.15 (dtdd, *J* = 15.3, 8.0, 4.7, 1.4 Hz, 2H), 1.83 – 1.74 (m, 3H), 1.74 – 1.56 ppm (m, 2H);

^{13}C NMR (151 MHz, $CDCl_3$) δ 138.4, 133.2, 131.1, 130.9, 130.2, 115.0, 72.4, 36.4, 29.8, 18.2 ppm;

Optical Rotation: $[\alpha]_D^{20}$ = -5.3° (c = 1.00 g / 100 mL, $CHCl_3$);

Enantiomeric excess: 99% *ee* (measured at Diels-Alder product **23**, see part II)

HRMS (m/z): calc'd for, $C_{10}H_{17}O^+$ $[M+H]^+$ 153.1274, found 153.1278.

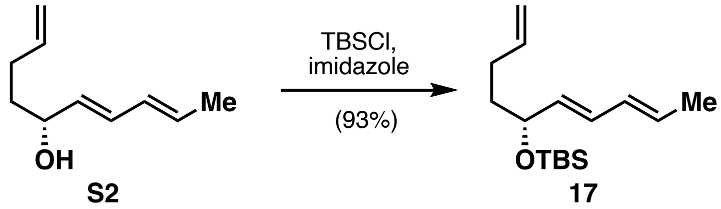

Experimental: The reduction product **S2** (9.52 g) was placed in a round bottom flask fixed with a stir bar. DMF (250.0 mL) was added *via* syringe and stirring was started. Imidazole (12.79 g, 187.8 mmol, 3.0 eq.) was added and stirred until dissolved. Finally, TBSCl (14.15 g, 93.9 mmol, 1.5 eq.) was added then the reaction was heated to 50 °C and stirred until complete (as determined by TLC). The reaction was quenched with $NaHCO_3$ (*sat., aq.,* 500 mL). The aqueous layer was extracted three times with Et_2O. The combined organic layers were rinsed with LiCl (10% *aq.*, 300 mL x 2) and brine (300 mL), dried over $MgSO_4$ and filtered. The solvent was removed under reduced pressure and the crude material was purified by flash column chromatography (SiO_2; 0% to 5% Et_2O in hexanes), yielding the product **17** (15.5 g, 93% yield).

Physical State: colorless oil;

TLC: R_f = 0.50 (1% Et_2O in hexanes);

^{1}H NMR (400 MHz, $CDCl_3$) δ 6.13 – 5.97 (m, 2H), 5.87 – 5.75 (m, 1H), 5.72 – 5.57 (m, 1H), 5.57 – 5.41 (m, 1H), 5.06 – 4.90 (m, 2H), 4.12 (q, *J* = 6.4 Hz, 1H), 2.16 – 2.00 (m, 2H), 1.78 – 1.72 (m, 3H), 1.69 – 1.47 (d, *J* = 6.4, 2H), 0.90 (s, 9H), 0.04 (s, 3H), 0.02 ppm (s, 3H);

^{13}C NMR (151 MHz, $CDCl_3$) δ 138.9, 134.3, 131.2, 129.8, 129.0, 114.5, 73.0, 37.8, 29.7, 26.1 (3C), 18.4, 18.2, -4.1, -4.6 ppm;

Optical Rotation: $[\alpha]_D^{20}$ = -13.9° (c = 1.00 g / 100 mL, $CHCl_3$);

HRMS (m/z): calc'd for, $C_{16}H_{31}OSi^+$ $[M+H]^+$ 267.2139, found 267.2138.

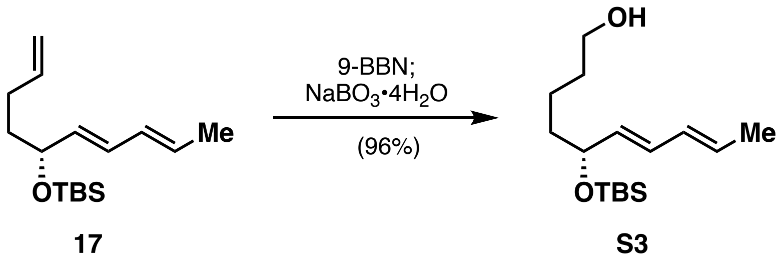

Experimental: The silyl ether **17** (15.5 g, 58.2 mmol) was placed in an oven-dried round bottom flask fixed with a stir bar and placed under nitrogen. THF (500.0 mL) was added to the flask and the contents were cooled to 0 °C. A solution of 9-BBN (0.5 M in THF, 163.0 mL, 81.5 mmol, 1.4 eq.) was then slowly added *via* syringe. The resulting solution was stirred for 30 min at the same temperature before stirring at room temperature for 4.5 h. Upon completion, the reaction was again cooled to 0 °C and sodium perborate tetrahydrate (35.84 g, 232.9 mmol, 4.0 eq.) was added in one portion. Then 200 mL of water was slowly added. Afterward, the reaction was warmed to room temperature and stirred overnight. The mixture was filtered through a pad of celite then transferred to a separatory funnel and extracted with Et_2O (3 x 300 mL). The combined organic layers were rinsed with brine, dried over $MgSO_4$, and filtered. The solvent was removed under reduced pressure and the crude material was purified by flash column chromatography (SiO_2; 30% Et_2O in hexanes), yielding the product **S3** (15.90 g, 96% yield).

Physical State: colorless oil;
TLC: R_f = 0.22 (30% Et_2O in hexanes);
^{1}H NMR (600 MHz, $CDCl_3$) δ 6.10 – 5.97 (m, 2H), 5.69 – 5.61 (m, 1H), 5.49 (dd, *J* = 14.5, 6.7 Hz, 1H), 4.10 (q, *J* = 6.3 Hz, 1H), 3.63 (t, *J* = 6.6 Hz, 2H), 1.75 (dd, *J* = 6.7, 1.6 Hz, 3H), 1.64 – 1.17 (m, 6H), 0.89 (s, 9H), 0.04 (s, 3H), 0.02 ppm (s, 3H);
^{13}C NMR (151 MHz, $CDCl_3$) δ 134.3, 131.2, 129.8, 129.0, 73.4, 63.1, 38.3, 32.9, 26.1 (3C), 21.5, 18.4, 18.2, -4.1, -4.6 ppm;
Optical Rotation: $[\alpha]_D^{20}$ = +2.0° (c = 1.00 g / 100 mL, $CHCl_3$);
HRMS (m/z): calc'd for, $C_{16}H_{33}O_2Si^+$ $[M+H]^+$ 285.2244, found 285.2245.

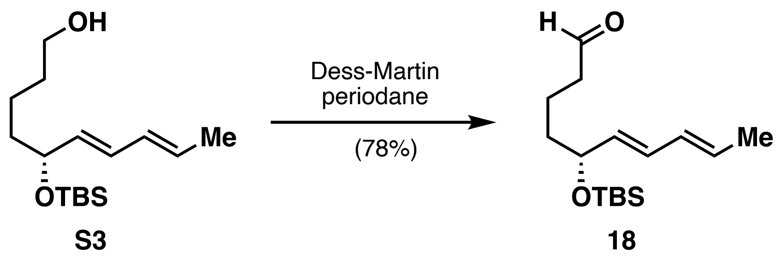

Experimental: The primary alcohol **S3** (15.9 g, 55.94 mmol) was placed in an oven-dried round bottom flask fixed with a stir. CH_2Cl_2 (350 mL) was added to the flask *via* syringe and the solution was stirred. To the flask was added Dess-Martin periodane (33.22 g, 78.32 mmol, 1.4 eq.) The reaction was stirred for 2.5 h at room temperature. Upon completion, the reaction is directly filtered through a pad of celite using CH_2Cl_2 to rinse. The excess DMP was quenched with $Na_2S_2O_3$ (*sat., aq.*, 200 mL), and the mixture was extracted with DCM (3 x 200 mL). The combined organic phase was washed with $NaHCO_3$ (*sat., aq.*) and brine. The solvent was removed under reduced pressure. This material was then suspended in hexanes and again filtered through a pad of celite, using hexanes to rinse. The solvent was removed under reduced pressure and the material was purified by using a short plug of silica gel (SiO_2; CH_2Cl_2) to remove any residual polar impurities, yielding the product **18** (12.3 g, 78% yield).

Physical State: colorless oil;

TLC: R_f = 0.71 (30% Et_2O in hexanes);

^{1}H NMR (600 MHz, $CDCl_3$) δ 9.74 (t, *J* = 1.8 Hz, 1H), 6.11 – 5.95 (m, 2H), 5.66 (dq, *J* = 13.6, 6.7 Hz, 1H), 5.48 (dd, *J* = 14.9, 6.7 Hz, 1H), 4.12 (q, *J* = 6.3 Hz, 1H), 2.42 (td, *J* = 7.5, 1.8 Hz, 2H), 1.75 (d, *J* = 6.9 Hz, 3H), 1.72 – 1.58 (m, 2H), 1.52 (m, 2H), 0.88 (s, 9H), 0.04 (s, 3H), 0.02 ppm (s, 3H);

^{13}C NMR (151 MHz, $CDCl_3$) δ 202.8, 133.8, 131.1, 130.1, 129.3, 73.1, 44.0, 37.9, 26.0 (3C), 18.4, 18.2, 18.0, -4.1, -4.7 ppm;

Optical Rotation: $[\alpha]_D^{20}$ = +2.5° (c = 1.00 g / 100 mL, $CHCl_3$);

HRMS (m/z): calc'd for, $C_{16}H_{31}O_2Si^+$ $[M+H]^+$ 283.2088, found 283.2086.

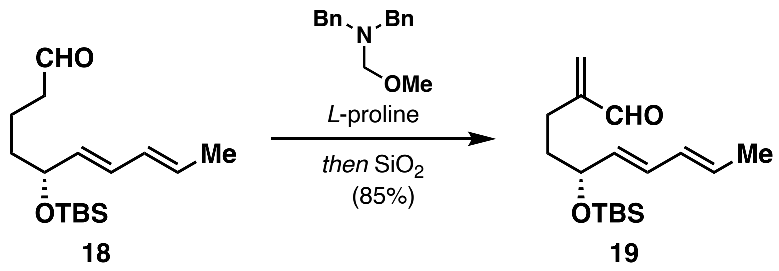

Experimental: The aldehyde (1.36 g, 4.81mmol) was added to a round bottom flask fixed with a stir bar and placed under argon. DMF (6.4 mL) was added via syringe and the solution was cooled to 0 °C. *L*-proline (110.7 mg, 0.962 mmol, 0.20 eq.) was added to the flask and the resulting solution was stirred for 5 minutes. Next, the aminal (1.22 mL, 1.278 g, 5.29 mmol, 1.1 eq.) was added *via* syringe and the reaction was stirred for 2 hours. Upon completion, the reaction was quenched with NH_4Cl (*sat., aq.*, 25 mL) and extracted with Et_2O (3 x 40 mL). The combined organic layers were rinsed with LiCl (10% aq., 50 mL), then brine, dried over $MgSO_4$, and filtered. The solvent was removed under reduced pressure. The crude material was redissolved in DCM (50 mL) and SiO_2 (15.0 g) was added to the flask. The resulting slurry was stirred at room temperature for 5 h. Upon completion, the slurry was filtered, and the solids were rinsed with DCM. The solvent was removed under reduced pressure and the crude material was purified by flash column chromatography (SiO_2; 0% to 5% Et_2O in hexanes), yielding the product **19** (1.20 g, 85% yield).

Physical State: colorless oil;

TLC: R_f = 0.56 (10% Et_2O in hexanes);

^{1}H NMR (600 MHz, $CDCl_3$) δ 9.53 (s, 1H), 6.23 (s, 1H), 6.10 – 5.96 (m, 2H), 6.02 (ddd, *J* = 13.9, 11.3, 1.7 Hz, 1H), 5.97 (s, 1H), 5.66 (dq, *J* = 13.6, 6.7 Hz, 1H), 5.49 (dd, *J* = 15.0, 6.7 Hz, 1H), 4.13 (q, *J* = 6.3 Hz, 1H), 2.36 – 2.19 (m, 2H), 1.74 (dd, *J* = 7.4 Hz, 3H), 1.69 – 1.57 (m, 2H), 0.89 (s, 9H), 0.04 (s, 3H), 0.02 ppm (s, 3H);

^{13}C NMR (151 MHz, $CDCl_3$) δ 194.8, 150.5, 133.9, 133.7, 131.1, 130.2, 129.3, 73.1, 36.5, 26.0 (3C), 23.9, 18.4, 18.2, -4.1, -4.6 ppm;

Optical Rotation: $[\alpha]_D^{20}$ = +6.9° (c = 1.00 g / 100 mL, $CHCl_3$);

HRMS (m/z): calc'd for $C_{17}H_{31}O_2Si^+$ $[M+H]^+$ 295.2088, found 295.2100.

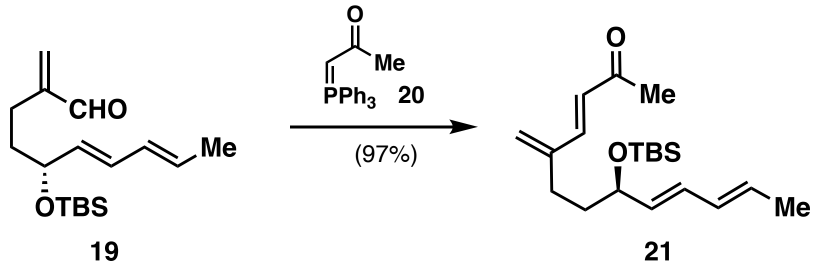

Experimental: To a flame dried vial was added the enal **19** (8.82 g, 30.0 mmol). The vial was placed under argon and DCM (30.0 mL) was added *via* syringe. After becoming homogeneous, acetylmethylene-triphenylphosphorane **20** (17.17 g, 54.0 mmol, 1.8 eq.) was added, and the vial was tightly sealed. The reaction was heated to 45 °C and stirred at this temperature for 24 h. Upon completion, the reaction was diluted with hexanes and stirred rapidly for 5 min. The resulting slurry was filtered over a pad of celite and rinsed with excess hexanes. The solvent was removed under reduced pressure and the crude material was purified by flash column chromatography (SiO_2; 5% to 10% Et_2O in hexanes), yielding the product **21** (9.72 g, 97% yield).

Physical State: colorless oil;

TLC: R_f = 0.35 (10% Et_2O in hexanes);

^{1}H NMR (600 MHz, C_6D_6) δ 7.12 (d, *J* = 16.2, 1H), 6.36 (d, *J* = 16.2 Hz, 1H), 6.27 (dd, *J* = 15.3, 10.4 Hz, 1H), 6.14 – 6.06 (m, 1H), 5.68 – 5.58 (m, 2H), 5.22 (d, *J* = 1.4 Hz, 1H), 5.17 (s, 1H), 4.19 (t, *J* = 6.0 Hz, 1H), 2.42 (dq, *J* = 14.9, 5.3, 1H), 2.32 (dq, *J* = 14.8, 5.3, 1H), 1.98 (s, 3H), 1.86 – 1.71 (m, 2H), 1.67 (dd, *J* = 6.8, 1.7 Hz, 3H), 1.12 (s, 9H), 0.20 (s, 3H), 0.20 ppm (s, 3H);

^{13}C NMR (151 MHz, C_6D_6) δ 196.6, 145.5, 144.2, 134.0, 131.5, 130.6, 129.4, 127.7, 123.2, 73.3, 37.4, 27.6, 27.3, 26.2 (3C), 18.5, 18.1, -3.9, -4.6 ppm;

Optical Rotation: $[\alpha]_D^{20}$ = +3.5° (c = 1.00 g / 100 mL, $CHCl_3$);

HRMS (m/z): calc'd for $C_{20}H_{35}O_2Si^+$ $[M+H]^+$ 335.2401, found 335.2409.

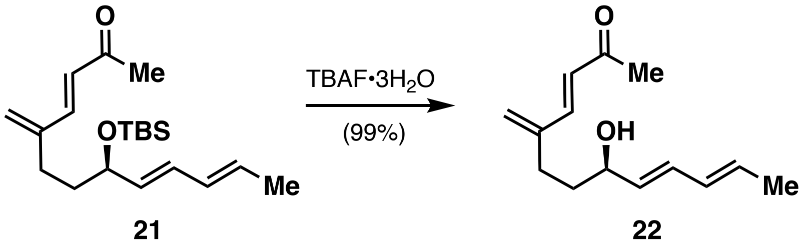

Experimental: The above material **21** (9.72 g, 29.1 mmol) was placed under argon and dissolved in dry THF (160.0 mL). The solution was cooled to 0°C and TBAF•3H_2O (18.3 g, 58.2 mmol, 2.0 eq.) was added in one portion. After complete addition, the reaction was warmed to room temperature and stirred for about 2 h. Upon completion, the reaction was quenched with NH_4Cl (*sat., aq.*, 120 mL) and extracted with Et_2O (3 x 150 mL). The combined organic layers were rinsed with brine, dried over $MgSO_4$, and filtered. The solvent was removed under reduced pressure and the crude material was purified by flash column chromatography (SiO_2; 25% to 35% EtOAc in hexanes), yielding the product **22** (6.34 g, 99% yield).

Physical state: yellow to colorless oil;

R_f Value: 0.40 (35% EtOAc in hexanes);

^{1}H NMR (600 MHz, $CDCl_3$) δ 7.20 – 6.99 (dd, *J* = 16.2, 0.7 Hz, 1H), 6.28 – 6.15 (dd, *J* = 15.8, 10.7 Hz, 2H), 6.10 – 5.93 (m, 1H), 5.85 – 5.69 (m, 1H), 5.60 – 5.51 (ddq, *J* = 15.3, 7.0, 0.7 Hz, 1H), 5.50 – 5.27 (m, 2H), 4.24 – 4.09 (q, *J* = 6.7 Hz, 1H), 2.47 – 2.22 (m, 5H), 1.85 – 1.67 ppm (m, 5H);

^{13}C NMR (151 MHz, $CDCl_3$) δ 199.1, 145.5, 144.8, 132.8, 131.6, 130.8, 130.7, 127.5, 124.3, 72.5, 35.6, 27.6, 27.5, 18.3 ppm;

Optical Rotation: $[\alpha]_D^{20}$ = +5.3° (c = 1.00 g / 100 mL, $CHCl_3$);

HRMS (m/z): calc'd for $C_{14}H_{21}O_2^+$ [M+H]$^+$ 221.1536, found 221.1537.

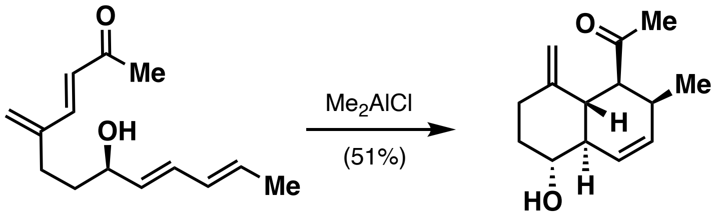

22 **23**

Experimental: To a flame dried flask fixed with a stir bar and sealed under argon was added the tetraene **22** (1.58 g, 7.2 mmol) *via* syringe. DCM (140.0 mL) was then added and the resulting solution was cooled to -20 °C. Me_2AlCl (0.9 M in heptane; 8.0 mL, 7.2 mmol, 1.0 eq.) was then added dropwise. The reaction was then allowed to stir while slowly warming up to room temperature for 18 hours. Upon completion, the reaction was quenched with NH_4Cl (*sat., aq.*, 30 mL), and a solution of Rochelle's salt (*sat., aq.*, 100 mL) was added to the flask. The biphasic mixture was stirred vigorously for 30 min. The mixture was transferred to a separatory funnel and extracted with Et_2O (2 x 150 mL). The combined organic layers were rinsed with brine, dried over $MgSO_4$, and filtered. The solvent was removed under reduced pressure and the crude material was purified by flash column chromatography (SiO_2; 25% to 35% EtOAc in hexanes), yielding the product **23** (802 mg, 51% yield).

Physical state: white crystalline solid;

Melting point: 151-152 °C

R_f Value: 0.36 (35% EtOAc in hexanes);

^{1}H NMR (600 MHz, $CDCl_3$) δ 5.94 (dt, *J* = 10.0, 1.8 Hz, 1H), 5.74 (ddd, *J* = 10.0, 4.7, 2.6 Hz, 1H), 4.76 – 4.72 (s, 1H), 4.19 (s, 1H), 3.47 (td, *J* = 10.4, 4.5 Hz, 1H), 3.08 (dd, *J* = 11.7, 6.1 Hz, 1H), 2.68 – 2.61 (m, 1H), 2.38 – 2.33 (m, 1H), 2.26 – 2.16 (m, 6H), 1.78 (tq, *J* = 10.5, 2.2 Hz, 1H), 1.48 – 1.37 (m, 1H), 0.83 ppm (d, *J* = 7.2 Hz, 3H);

^{13}C NMR (151 MHz, $CDCl_3$) δ 210.2, 150.1, 132.0, 125.4, 105.6, 73.8, 52.2, 50.4, 38.3, 38.2, 34.8, 31.3, 30.4, 17.9 ppm;

Optical Rotation: $[\alpha]_D^{20}$ = +172.2° (c = 1.00 g / 100 mL, $CHCl_3$);

Enantiomeric excess: 99% *ee* (measured by analytical SFC, see part II)

HRMS (m/z): calc'd for $C_{14}H_{21}O_2^+$ $[M+H]^+$ 221.1536, found 221.1543.

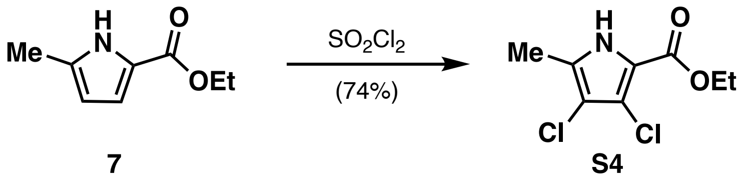

Experimental: In a round bottom flask fixed with a stirbar was placed ethyl 5-methyl-1*H*-pyrrole-2-carboxylate (7.65 g, 50.0 mmol). The flask was placed under argon and CCl_4 (35 mL) was added. The resulting mixture was cooled to -20 °C and SO_2Cl_2 (8.88 ml, 14.85 g, 110 mmol, 2.2 eq.) was added dropwise *via* syringe. The reaction was allowed to stir for 4 h, after which the mixture was filtered to collect a pink solid. The solid was washed by CCl_4 (10 ml) twice to give the crude product. The crude product **S4** (8.18 g, 74 % yield) was dried under reduced pressure and ready for next step without further purification.

Physical State: pink solid;
R_f Value: 0.60 (40% EtOAc in hexanes);
^{1}H NMR (500 MHz, $CDCl_3$): δ (ppm) 9.23 (s, 1H), 4.36 (q, *J* = 7.1 Hz, 2H), 2.29 (s, 3H), 1.39 ppm (t, *J* = 7.1 Hz, 3H);
^{13}C NMR (151 MHz, $CDCl_3$) δ (ppm) 160.1, 129.2, 117.6, 116.2, 111.9, 61.1, 14.5, 11.6 ppm;
HRMS (m/z): calc'd for $C_8H_{10}Cl_2NO_2^+$ $[M+H]^+$ 222.0089, found 222.0083.

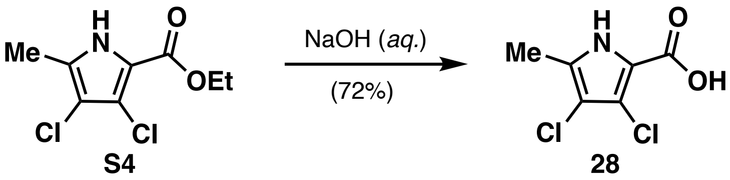

Experimental: The starting material **S4** (3.20 g, 14.48 mmol) was dissolved in MeOH (80.0 mL). To this was added 2.0 M NaOH (*aq.*) (80.0 mL, equal volume). The flask was then fitted with a reflux condenser and the solution was heated to 60 °C for 4 h. Upon completion, the reaction was allowed to cool to room temperature. The MeOH in the reaction mixture was removed first under reduced pressure, then the reaction mixture was brought to a pH of ~2 *via* the addition of 2.0 M HCl (*aq.*) and extracted 3 times with DCM. The combined organic layers were rinsed with brine, dried over Na_2SO_4 then filtered. The solvent was removed under reduced pressure yielding the desired compound **28** (2.0 g, 72% yield), which was used directly without further purification.

Physical State: purple solid;
R_f Value: 0.35 (10% MeOH in DCM);
^{1}H NMR (400 MHz, CD_3OD): δ 2.23 ppm (s, 3H);
NOTE: The data was identical to the reported literature. [2]

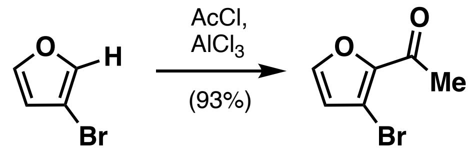

S5 **6**

Experimental: According to Lit.:[3] To 0 °C cold DCM (550 mL) was added $AlCl_3$ (66.67 g, 500.0 mmol, 2.94 eq.) portion wise. After stirring for 15 min at 0 °C, a solution of freshly distilled acetyl chloride (38.8 mL, 544.0 mmol, 3.2 eq.) in DCM (550 mL) was added dropwise. After stirring for 30 minutes at 0 °C, a solution of distilled 3-bromofuran **S5** (15.3 mL, 170.0 mmol) in DCM (550 mL) was added dropwise. The reaction mixture was stirred for 20 min at 0 °C, 10 min at room temperature and then cooled to 0 °C. At 0 °C, H_2O (1.5 L) was added slowly. The reaction mixture was transferred to a separatory funnel and the aqueous phase was extracted with DCM (3x 200 mL). The combined organic phases were washed with H_2O (3x 200 mL), $NaHCO_3$ (*sat., aq.,* 3x 300 mL) and brine (500 mL), dried over anhydrous Na_2SO_4, filtered and evaporated. The obtained crude product was purified *via* column chromatography on SiO_2 (^{n}pentane/Et_2O 10/1 to 5/1) to obtain the acylated bromofuran **6** (29.97 g, 93% yield). The spectral data are in good agreement with previous reports.

Physical State: red-orange solid;

R_f Value: 0.28 (^{n}pentane/Et_2O 10:1);

^{1}H NMR (400 MHz, $CDCl_3$) δ 7.49 (d, J = 1.8 Hz, 1H), 6.62 (d, J = 1.9 Hz, 1H), 2.55 ppm (s, 3H);

^{13}C NMR (126 MHz, $CDCl_3$) δ 186.7, 148.5, 145.5, 117.6, 107.2, 27.6 ppm;

HRMS (m/z) calc'd for $C_6H_6BrO_2^+$ $[M+H]^+$ 188.9546, found 188.9548.

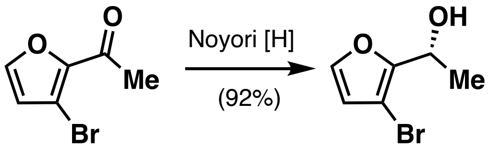

6 **S6**

Experimental: Similar to Lit.:[4] To Ar freeze-pump degassed (4x) H_2O (300 mL) was added $[RuCl_2(^{p}cymene)]_2$ (0.712 g, 1.16 mmol, 0.0075 eq.) and *N*-((1*R*,2*R*)-2-amino-1,2-diphenylethyl)-4-methylbenzenesulfonamide (1.03 g, 2.8 mmol, 0.018 eq.). After stirring the mixture at 70 °C for 1.5 h and cooling to room temperature, a solution of ketone **6** (29.3 g, 155.0 mmol) in THF (150 mL) and sodium formate (52.71 g, 775.0 mmol, 5.0 eq.) was added. The reaction mixture was stirred at 40 °C for 2 days, cooled to room temperature, and extracted with EtOAc (3x 200 mL). The combined org. layers were washed with H_2O (3x 100 mL) and brine (400 mL), dried over anhydrous Na_2SO_4, filtered, and evaporated. The obtained crude product was purified *via* column chromatography on SiO_2 (^{n}pentane/Et_2O 2:1) to obtain the corresponding alcohol **S6** (27.32 g, 92% yield).

Physical State: colorless liquid;
R_f Value: 0.56 (^{n}pentane/Et_2O 2:1);
^{1}H NMR (600 MHz, $CDCl_3$) δ 7.33 – 7.34 (m, 1H), 6.39 – 6.39 (m, 1H), 4.98 – 5.03 (m, 1H), 1.96 (s, broad, 1H), 1.56 ppm (dd, *J* = 1.6, 6.7 Hz, 3H);
^{13}C NMR (151 MHz, $CDCl_3$) δ 153.1, 142.2, 114.1, 96.6, 62.0, 21.0 ppm;
Optical Rotation: $[\alpha]_D^{20}$ = +11.6° (c = 0.50 g / 100 mL, $CHCl_3$);
Enantiomeric excess: 92% (as determined *via* analytical SFC, see part II);
HRMS (m/z): calc'd for $C_6H_6BrO^+$ $[M-OH]^+$ 172.9597, found 172.9602.

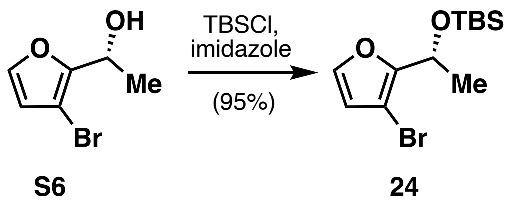

Experimental: To a solution of alcohol **S6** (26.74 g, 140.0 mmol) in DMF (450 mL) was added imidazole (28.59 g, 420.0 mmol, 3.0 eq.) and TBDMSCl (31.65 g, 210.0 mmol, 1.5 eq.). After stirring the reaction mixture overnight at room temperature, H_2O (1.5 L) was added slowly, and the aq. phase was extracted with ^{n}pentane (5x 200 mL). The combined org. layers were washed with brine (500 mL), dried over anhydrous Na_2SO_4, filtered, and evaporated. The obtained crude product was purified via column chromatography on SiO_2 (^{n}pentane/Et_2O 20:1) to obtain the corresponding silyl ether **24** (40.64 g, 95% yield).

Physical State: colorless liquid;
R_f Value: 0.84 (^{n}pentane/Et_2O 20:1);
^{1}H NMR (600 MHz, $CDCl_3$) δ 7.33 (d, J = 1.9 Hz, 1H), 6.35 (d, J = 1.9 Hz, 1H), 4.97 (q, J = 6.6 Hz, 1H), 1.47 (d, J = 6.6 Hz, 3H), 0.86 (s, 9 H), 0.07 (s, 3H), –0.05 ppm (s, 3H);
^{13}C NMR (151 MHz, $CDCl_3$) δ 153.7, 142.0, 113.7, 95.6, 62.5, 25.9 (3C), 22.8, 18.3, –4.9 (2C) ppm;
Optical Rotation: $[\alpha]_D^{20}$ = +9.4° (c = 0.50 g / 100 mL, $CHCl_3$);
HRMS (m/z): calc'd for $C_{12}H_{22}BrO_2Si^+$ $[M+H]^+$ 305.0567, found 305.0574.

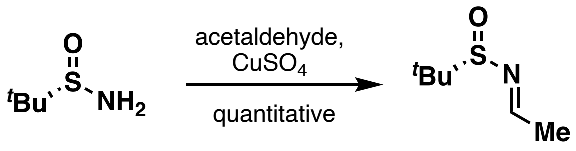

25

Experimental: According to Lit.:[5] To a suspension of anhydrous $CuSO_4$ (143.65 g, 900.0 mmol) and (*S*)-methylpropansulfinamide (36.36 g, 300.0 mmol) in 1,2-dichloroethane (500 mL) was added acetaldehyde (33.75 mL, 600.0 mmol). After stirring the reaction mixture overnight at 60 °C, the mixture was cooled to room temperature, filtered and evaporated. The obtained crude product was purified via column chromatography on SiO_2 ($CHCl_3$/MeOH 25:1) to obtain the corresponding sulfinyl imine **25** (44.11g, quantitative yield). The spectral data are in good agreement with previous reports.

Physical State: slightly orange liquid;
R_f Value: 0.32 ($CHCl_3$/MeOH 25:1);
^{1}H NMR (600 MHz, $CDCl_3$) δ 8.08 (q, *J* = 5.1 Hz, 1H), 2.23 (d, *J* = 5.1 Hz, 3H), 1.19 ppm (s, 3H);
^{13}C NMR (151 MHz, $CDCl_3$) δ 166.1, 56.7, 22.6, 22.5 (3C) ppm;
Optical Rotation: $[\alpha]_D^{20}$ = +310.2° (c = 0.5 g / 100 mL, $CHCl_3$);
HRMS (method, m/z) calcd. for $C_6H_{14}NOS^+$ $[M+H]^+$ 148.0791, found: 148.0794.

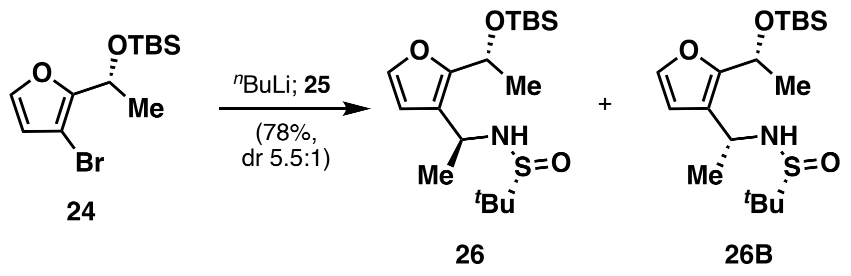

Experimental: To a –78 °C cold solution of chiral bromide **24** (15.27 g, 50.0 mmol) in Et_2O (250 mL) was added ^{n}BuLi (2.5 M in hexanes, 22.0 mL, 55.0 mmol, 1.1 eq.). After stirring the reaction mixture at –40 °C for 1 hour, it was re-cooled to –78 °C and a solution of sulfinimine **25** (8.82 g, 60.0 mmol, 1.2 eq.) in Et_2O (20 mL) was added dropwise. The reaction was stirred for 30 min at –78 °C and 1.5 hours at room temperature, before NH_4Cl (*sat., aq.,* 250 mL) was added and the aq. phase was extracted with Et_2O (3 x 125 mL). The combined organic layers were dried over anhydrous Na_2SO_4, filtered, and evaporated. The obtained crude product was purified *via* column chromatography on SiO_2 (hexanes/EtOAc 3:1 to 1:1) to obtain the corresponding sulfonamide **26** (12.29 g, 66% yield) and related diastereomer **26B** (2.24g, 12%).

Major compound **26:**

Physical State: slightly yellow oil;

R_f Value: 0.47 (hexanes/EtOAc 1:1);

^{1}H NMR (600 MHz, $CDCl_3$) δ 7.25 (d, *J* = 1.9 Hz, 1H), 6.32 (d, *J* = 2.0 Hz, 1H), 4.96 (q, *J* = 6.5 Hz, 1H), 4.72 (dq, *J* = 2.5, 6.5 Hz, 1H), 3.28 (s, 1H), 1.47 (d, *J* = 6.6 Hz, 3H), 1.40 (d, *J* = 6.5 Hz, 3H),1.19 (s, 9H), 0.85 (s, 9H), 0.04 (s, 3H), –0.03 ppm (s, 3H);

^{13}C NMR (151 MHz, $CDCl_3$) δ 152.8, 141.1, 122.2, 109.2, 64.0, 55.4, 45.0, 25.9 (3C), 24.1, 22.7 (3C), 22.5, 18.3, –4.8 (2C) ppm;

Optical Rotation: $[\alpha]_D^{20}$ = +108.2° (c = 0.50 g / 100 mL, $CHCl_3$);

HRMS (m/z) calc'd for $C_{18}H_{36}NO_3SSi^+$ $[M+H]^+$ 374.2180, found 374.2186.

Minor compound **26B:**

Physical State: slightly yellow oil;

R_f Value: 0.42 (hexanes/EtOAc 1:1)

^{1}H NMR (600 MHz, $CDCl_3$) δ 7.31 (d, *J* = 1.9 Hz, 1H), 6.26 (d, *J* = 1.8 Hz, 1H), 5.01 (q, *J* = 6.5 Hz, 1H), 4.65 – 4.59 (m, 1H), 3.25 (s, 1H), 1.52 (d, *J* = 6.5 Hz, 3H), 1.48 (d, *J* = 6.6 Hz, 3H), 1.16 (s, 9H), 0.86 (s, 9H), 0.04 (s, 3H), -0.03 ppm (s, 3H);

^{13}C NMR (151 MHz, $CDCl_3$) δ 153.4, 141.6, 120.9, 108.7, 62.9, 55.4, 45.6, 26.0 (3C), 23.8, 23.6, 22.7 (3C), 18.3, -4.7 (2C) ppm;

Optical Rotation: $[\alpha]_D^{20}$ = +51.9° (c = 0.42 g / 100 mL, $CHCl_3$);

HRMS (m/z) calc'd for $C_{18}H_{36}NO_3SSi^+$ [M+H]$^+$ 374.2180, found 374.2183.

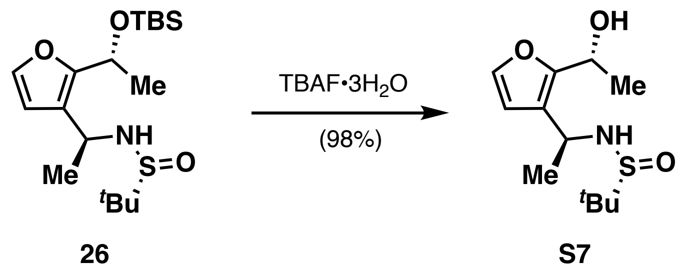

Experimental: To a 0 °C cold solution of chiral silyl ether **26** (34.75 g, 93.0 mmol) in THF (930 mL) was added TBAF·$(H_2O)_3$ (58.68 g, 186.0 mmol, 2.0 eq.). After stirring the reaction mixture at room temperature for 30 min, all volatiles were removed under reduced pressure, before NH_4Cl solution (*sat. aq.*, 500 mL) was added and the aq. phase was extracted with EtOAc (3x 250 mL). The combined org. layers were dried over anhydrous Na_2SO_4, filtered, and evaporated. The obtained crude product was purified *via* column chromatography on SiO_2 (EtOAc) to obtain the corresponding alcohol **S7** (23.62 g, 98% yield).

Physical State: slightly yellow oil;
R_f Value: 0.42 (EtOAc);
^{1}H NMR (600 MHz, $CDCl_3$) δ 7.29 (d, *J* = 1.9 Hz, 1H), 6.32 (d, *J* = 1.9 Hz, 1H), 5.06 (dq, *J* = 3.3, 6.6 Hz, 1H), 4.72 (s, 1H), 4.58 – 4.63 (m, 1H), 3.48 (d, *J* = 6.6 Hz, 1H), 1.55 (d, *J* = 6.6 Hz, 3H), 1.47 (d, *J* = 6.7 Hz, 3H), 1.18 ppm (s, 9H);
^{13}C NMR (151 MHz, $CDCl_3$) δ 153.4, 141.5, 123.2, 108.1, 60.6, 55.9, 47.1, 22.7 (3C), 22.5, 20.2 ppm;
Optical Rotation: $[\alpha]_D^{20}$ = +177.8° (c = 0.50 g / 100 mL, $CHCl_3$);
HRMS (m/z) calc'd for $C_{12}H_{22}NO_3S^+$ $[M+H]^+$ 260.1315, found: 260.1324.

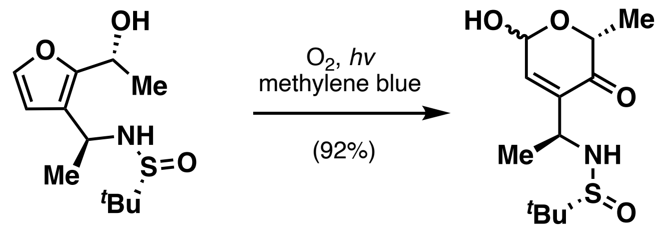

Experimental: A -78 °C cold solution of alcohol **17** (3.501 g, 13.5 mmol) and methylene blue (64.0 mg, 0.135 mmol, 0.01 eq.) in CH_2Cl_2 (200 mL) was irradiated with a 125 W visible-light lamp, while O_2 was constantly bubbled through the solution, until all starting material was consumed (checked *via* TLC, usually about 4 h). After addition of Me_2S (5.0 eq), the mixture was stirred for 1 hour at -78 °C and 1 hour at room temperature. The crude reaction mixture was concentrated under reduced pressure and purified *via* column chromatography on SiO_2 (EtOAc) to obtain the corresponding enone **27** as a mixture of anomers (3.44 g, 92% yield).

Physical State: hygroscopic yellow solid;
R_f Value: 0.27 (EtOAc);
^{1}H NMR (600 MHz, $CDCl_3$) δ 6.79 (d, J = 3.4 Hz, 1H), 5.59 (m, 1H), 4.65 (q, J = 6.7 Hz, 1H), 4.23 – 4.29 (m, 1H), 4.05 (d, J = 8.8 Hz, 1H), 1.38 (d, J = 6.9 Hz, 3H), 1.34 (d, J = 6.9 Hz, 3H), 1.21 ppm (s, 9H);
^{13}C NMR (151 MHz, $CDCl_3$) δ 197.1, 197.0, 143.8, 140.1, 139.8, 139.2, 91.1, 88.0, 75.1, 70.4, 56.4 (2C), 53.8, 51.3, 22.7 (6 C), 21.8 (2C), 16.3, 15.6 ppm;
NOTE: 1 carbon (^{t}Bu) was not observed due to incidental equivalence.
HRMS (m/z) calc'd for $C_{12}H_{21}NO_4SNa^+$ [M+Na]$^+$ 298.1083, found: 298.1091.

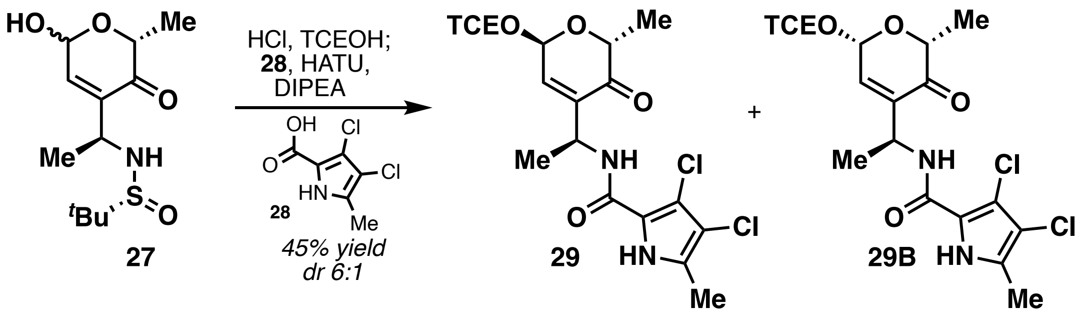

Experimental: To a solution of compound **27** (1.1 g, 4.0 mmol) in 7 mL dry trichloroethanol was added anhydrous PTSA (137.6 mg, 0.8 mmol, 0.2 eq.) at room temperature. The reaction was stirred at room temperature for 1.5 h and HCl (4.0 M in dioxane, 2.0 mL, 8.0 mmol, 2.0 equiv) was added. The resulting reaction mixture was stirred at room temperature for another 1.5 h. Most of the volatiles were removed under reduced pressure and the crude material was placed under high vacuum for at least 1 h.

HATU (2.3 g, 6.0 mmol, 1.5 eq.) and **28** (1.2 g, 6.0 mmol, 1.5 eq.) were dissolved in 50 mL DMF in another flask and DIPEA (2.8 mL, 16.0 mmol, 4.0 eq.) was added. After stirred at room temperature for 15 min, the resulting mixture was transferred to the crude amine salt obtained above. After stirring at room temperature for 4 h, another portion of mixture of HATU (0.8 g, 2.0 mmol, 0.5 eq.), **28** (0.4 g, 2.0 mmol, 0.5 eq.) and DIPEA (1.0 mL, 6.0 mmol, 1.5 eq.) in 17 mL DMF was added. Stirring was continued for another 4 h before the reaction mixture was quenched with sat. aq. $NaHCO_3$. The mixture was extracted with EtOAc (3x50 mL) and the combined organic layers were washed with 10% aq. LiCl (2 x 20 ml), brine, dried over anhydrous Na_2SO_4 and filtered. The solvent was removed under reduced pressure and the crude product was purified *via* column chromatography on SiO_2 (hexanes/EtOAc 7:1 to 3:1) to obtain the corresponding amide **29** (716 mg, 39% yield) and **29B** (119 mg, 6% yield).

Major anomer **29**:

Physical State: yellow foam;

R_f Value: 0.67 (Hexanes/EtOAc 1:1);

^{1}**H NMR** (600 MHz, acetone-*d$_6$*) δ 7.23 (d, *J* = 8.4 Hz, 1H), 6.97 (dd, *J* = 3.7, 1.0 Hz, 1H), 5.66 (d, *J* = 3.7 Hz, 1H), 5.09 (pd, *J* = 7.0, 1.2 Hz, 1H), 4.72 (q, *J* = 6.7 Hz, 1H), 4.46 (d, *J* = 11.7 Hz, 1H), 4.42 (d, *J* = 11.7 Hz, 1H), 2.28 (s, 3H), 1.40 (d, *J* = 7.0 Hz, 3H), 1.35 ppm (d, *J* = 6.7 Hz, 3H);

^{13}**C NMR** (151 MHz, acetone-*d$_6$*) δ 197.1, 158.8, 140.1, 137.6, 129.2, 119.9, 111.0, 110.0, 97.7, 95.1, 80.7, 71.4, 45.4, 21.0, 15.6, 11.0 ppm;

Optical Rotation: $[\alpha]_D^{25}$ = +32.9° (c = 1.00 g / 100 mL, $CHCl_3$);

HRMS (m/z) calc'd for $C_{16}H_{17}Cl_5N_2O_4Na^+$ $[M+Na]^+$ 498.9523, found: 498.9518.

Minor anomer **29B:**

Physical State: yellow oil;

R_f Value: 0.67 (Hexanes/EtOAc 1:1);

^{1}**H NMR** (600 MHz, acetone-*d$_6$*) δ 7.30 (d, *J* = 8.2 Hz, 1H), 7.01 (dd, *J* = 2.1, 1.0 Hz, 1H), 5.84 (dt, *J* = 2.0, 1.0 Hz, 1H), 5.09 – 5.03 (m, 1H), 4.50 (d, *J* = 11.7 Hz, 1H), 4.44 (d, *J* = 11.7 Hz, 1H), 4.39 (qd, *J* = 6.8, 1.0 Hz, 1H), 2.29 (s, 3H), 1.45 (d, *J* = 6.8 Hz, 3H), 1.42 ppm (d, *J* = 7.0 Hz, 3H);

^{13}**C NMR** (151 MHz, acetone-*d$_6$*) δ 197.1, 158.6, 141.0, 140.2, 129.0, 120.2, 110.9, 109.9, 97.5, 97.5, 80.6, 75.8, 45.3, 20.7, 17.5, 11.0 ppm;

Optical Rotation: $[\alpha]_D^{25}$ = +18.0° (c = 0.15 g / 100 mL, $CHCl_3$);

HRMS (m/z) calc'd for $C_{16}H_{17}Cl_5N_2O_4Na^+$ $[M+Na]^+$ 498.9523, found: 498.9525.

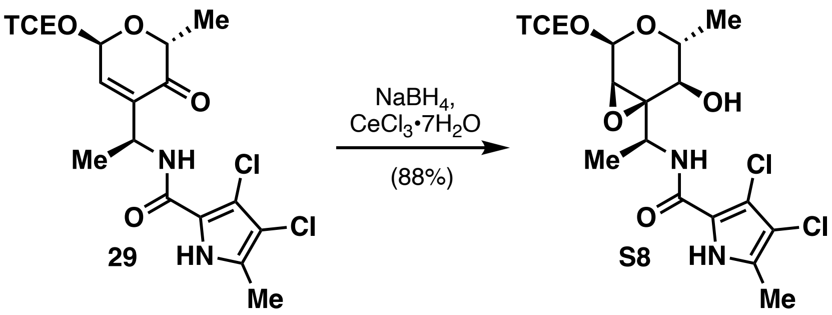

Experimental: Compound **29** (1.60 g, 3.36 mmol) was dissolved in MeOH (36.0 mL). The solution was cooled to 0 °C and $CeCl_3•7H_2O$ (501 mg, 1.34 mmol, 0.4 eq.) was added to the vial. The resulting solution was stirred for 5 min, after which $NaBH_4$ (508 mg, 13.45 mmol, 4.0 eq.) was added. After 20 mins, the reaction was quenched with acetone (10.0 mL), and stirred for 5 min at 0 °C and 5 min at room temperature. The volatiles were removed under reduced pressure and the crude material was placed under high vacuum briefly. The crude material was resuspended in DCM and rinsed with water then brine. The organic layer was dried over $MgSO_4$, and filtered. The solvent was removed under reduced pressure and the crude material was quickly passed through a pad of SiO_2 (Hexane/EtOAc 1:0 to 1:1) to obtain the corresponding semi pure allylic alcohol **S8** (1.41 g, 88% yield), which could be directly used for the next step.

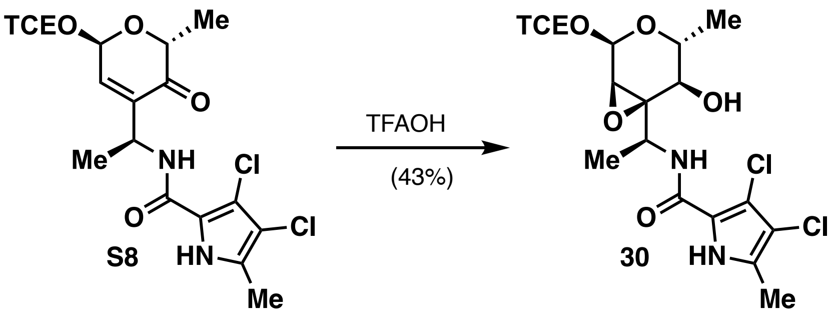

Experimental: To a suspension of urea hydrogen peroxide (320 mg, 3.4 mmol, 1.36 eq.) in dry DCM (20 mL) at 0 °C was added trifluoroacetic anhydride (0.715 g, 0.473 ml, 3.4 mmol, 1.36 eq.) and the reaction mixture was stirred for 1 h at the same temperature before stirring for 1 h at room temperature. This solution was kept at 0 °C and used as trifluoroperacetic acid solution.

Compound **S8** (1.19 g, 2.5 mmol, 1.0 eq) was placed in a flask with a stir bar and dissolved in dry DCM (8 ml). The resulting solution was cooled down to -15 °C then the premade trifluoroperacetic acid solution was added in one portion. Remove the cold bath and let the reaction gradually warm up. After 2 h, the reaction as quenched with aqueous saturated sodium sulfite and stirred at room temperature for 15 min. The aqueous layer was extracted three times with DCM. The combined organic layers were rinsed with sat. aqueous $NaHCO_3$ then brine. The organic layer was dried over $MgSO_4$ and filtered. The solvent was removed under reduced pressure. The crude material was purified by flash column chromatography (SiO_2; 20% to 50% EtOAc in hexanes) to provide epoxide **30** (531 mg, 43% yield).

Physical State: yellow foam;

R_f Value: 0.45 (50% EtOAc in hexanes);

^{1}H NMR (600 MHz, acetone-d_6) δ 6.80 (d, J = 7.7 Hz, 1H), 5.28 (dd, J = 3.1, 0.6 Hz, 1H), 4.58 (p, J = 7.0 Hz, 1H), 4.43 (d, J = 8.3 Hz, 1H), 4.34 – 4.27 (m, 2H), 3.90 – 3.84 (m, 1H), 3.72 – 3.67 (m, 2H), 2.29 (s, 3H), 1.43 (d, J = 6.9 Hz, 3H), 1.21 ppm (d, J = 6.3 Hz, 3H);

^{13}C NMR (151 MHz, acetone-d_6) δ 159.4, 129.0, 120.1, 110.8, 109.8, 98.0, 94.9, 79.8, 71.7, 67.2, 63.1, 58.7, 45.8, 17.8, 17.7, 11.0 ppm;

Optical Rotation: $[\alpha]_D^{25}$ = +58.7° (c = 1.66 g / 100 mL, $CHCl_3$);

HRMS (m/z) calc'd for $C_{16}H_{19}Cl_5N_2O_5Na^+$ $[M+Na]^+$ 516.9629, found: 516.9634.

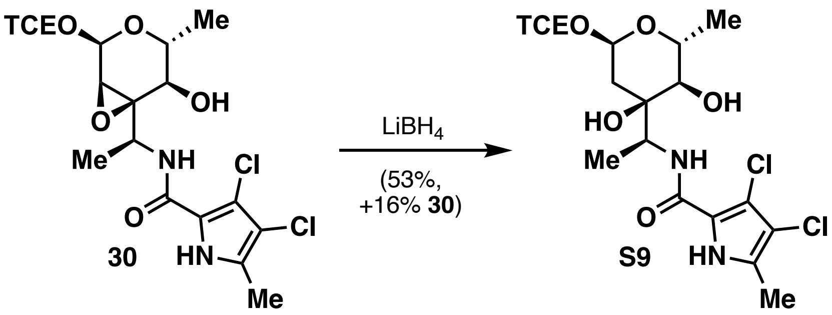

Experimental: To a solution of **30** (220.0 mg, 0.45 mmol) in 9 mL dry toluene was added $LiBH_4$ (2.0 M in THF, 0.45 mL, 0.89 mmol, 2.0 eq.) at room temperature. After stirred at 60 °C for 1 h, $LiBH_4$ (2.0 M in THF, 0.23 mL x 4, 0.45 mmol x 4, 1.0 eq. x 4) was added in four portions every 0.5 h. The reaction was cooled to room temperature and slowly quenched by the addition of NH_4Cl (*sat., aq.,* 15 mL). The reaction mixture was extracted with EtOAc (3 x 20 mL), and the combined organic layers were dried over anhydrous Na_2SO_4, filtered, and evaporated. The obtained crude product was purified *via* column chromatography on SiO_2 (hexanes/EtOAc 4:1 to 2:3) to obtain the corresponding alcohol **S9** (128.0 mg, 53% yield) and recovered **30** (37 mg, 16%).

Physical State: white foam;

R_f Value: 0.45 (Hexanes/EtOAc 1:2);

^{1}H NMR (600 MHz, acetone-d_6) δ 11.02 (s, 1H), 7.06 (d, J = 8.1 Hz, 1H), 5.28 (dd, J = 4.2, 2.0 Hz, 1H), 4.36 (d, J = 11.5 Hz, 1H), 4.33 (dq, J = 8.1, 6.8 Hz, 1H), 4.24 (d, J = 11.5 Hz, 1H), 3.99 (dq, J = 9.1, 6.2 Hz, 1H), 3.93 (s, 1H), 3.72 (d, J = 9.4 Hz, 1H), 3.29 (t, J = 8.8 Hz, 1H), 2.29 (s, 3H), 2.11 (dd, J = 14.7, 2.1 Hz, 1H), 2.05 (dd, J = 14.7, 4.2 Hz, 1H), 1.28 (d, J = 6.2 Hz, 3H), 1.27 ppm (d, J = 6.8 Hz, 3H);

^{13}C NMR (151 MHz, acetone-d_6) δ 159.5, 128.7, 120.7, 110.5, 109.9, 99.1, 97.8, 79.9, 75.0, 73.8, 67.2, 52.0, 36.2, 18.6, 16.6, 11.0 ppm;

Optical Rotation: $[\alpha]_D^{25}$ = +62.6° (c = 0.27 g / 100 mL, $CHCl_3$);

HRMS (m/z) calc'd for $C_{16}H_{21}Cl_5N_2O_5Na^+$ $[M+Na]^+$ 518.9785, found: 518.9786.

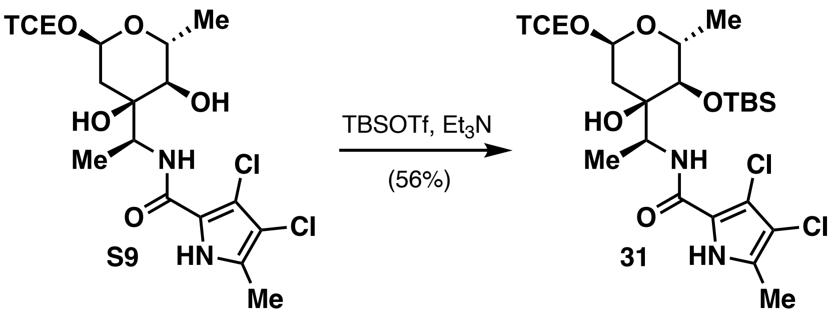

Experimental: To a solution of **S9** (166.0 mg, 0.335 mmol), Et_3N (0.47 mL, 3.35 mmol, 10.0 eq.) in 7 mL DCE was added TBSOTf (0.23 mL, 1.01 mmol, 3.0 eq.) at room temperature. After stirred at 40 °C for 4 h, another portion of TBSOTf (0.23 mL, 1.01 mmol, 3.0 eq.) was added and stirring was continued for another 3 h.

The reaction was cooled to room temperature and quenched by the addition of $NaHCO_3$ (*sat., aq.,* 15 mL). The reaction mixture was extracted with DCM (2 x 15 mL) and the combined organic layers were dried over anhydrous Na_2SO_4, filtered, and evaporated. The obtained crude product was purified *via* column chromatography on SiO_2 (hexanes/EtOAc 10:1 to 6:1) to obtain **31** (115.0 mg, 56% yield).

Physical State: white foam;

R_f Values: 0.56 (Hexanes/EtOAc 4:1);

^{1}H NMR (600 MHz, acetone-d_6) δ 11.02 (s, 1H), 7.02 (d, *J* = 9.0 Hz, 1H), 5.22 (t, *J* = 6.1 Hz, 1H), 4.45 (dq, *J* = 9.0, 7.6 Hz, 1H), 4.34 (d, *J* = 11.6 Hz, 1H), 4.25 (d, *J* = 11.6 Hz, 1H), 3.97 (p, *J* = 6.5 Hz, 1H), 3.51 (dd, *J* = 7.1, 1.3 Hz, 1H), 3.44 (d, *J* = 1.0 Hz, 1H), 2.31 (s, 3H), 2.13 (dd, *J* = 5.9, 1.4 Hz, 1H), 2.05 (dd, *J* = 5.9, 1.4 Hz, 1H), 1.28 (d, *J* = 6.0 Hz, 3H), 1.27 (d, *J* = 6.8 Hz, 3H), 0.92 (s, 9H), 0.12 (s, 3H), 0.07 ppm (s, 3H);

^{13}C NMR (151 MHz, acetone-d_6) δ 159.3, 128.9, 120.4, 110.4, 109.9, 98.5, 98.2, 79.8, 76.3, 74.0, 70.6, 49.2, 34.7, 26.5 (3C), 20.2, 19.0, 16.7, 11.1, -3.4, -3.8 ppm;

Optical Rotation: $[\alpha]_D^{25}$ = +57.4° (c = 1.72 g / 100 mL, $CHCl_3$);

HRMS (m/z) calc'd for $C_{22}H_{35}Cl_5N_2O_5SiNa^+$ $[M+Na]^+$ 633.0650, found: 633.0648.

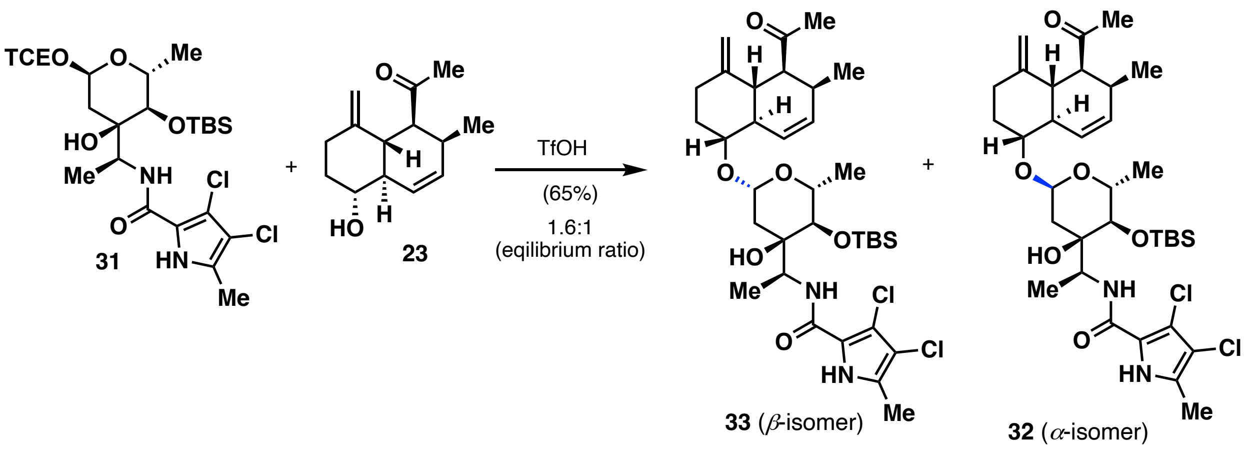

Experimental: A solution of **31** (56 mg, 0.092 mmol), **23** (41 mg, 0.184 mmol, 2.0 eq.), 20 mg 4A MS in 6 mL dry DCM was stirred for 15 min at room temperature. TfOH (8.1 µL, 0.092 mmol, 1.0 eq.) was added, and stirring was continued for 2.5 h at room temperature. The reaction mixture was quenched with $NaHCO_3$ (*sat., aq.,* 15 mL), extracted with DCM (3 x 15 mL). The combined organic layers were dried over anhydrous Na_2SO_4, filtered, and evaporated. The obtained crude product was purified via column chromatography on SiO_2 (hexanes/EtOAc 10:1 to 6:1 to 3:1) to obtain the corresponding β-anomer **33** (25.1 mg, 40% yield) and α-anomer **32** (14.5 mg, 25%).

For β-anomer **33:**

Physical State: white foam;

R_f Value: R_f = 0.57 (Hexanes/EtOAc 4:1);

^{1}H NMR (600 MHz, acetone-d_6): δ 6.73 (d, *J* = 8.0 Hz, 1H), 6.02 (dt, *J* = 10.1, 1.8 Hz, 1H), 5.65 (ddd, *J* = 10.1, 4.6, 2.7 Hz, 1H), 4.93 (dd, *J* = 9.2, 2.1 Hz, 1H), 4.66 (s, 1H), 4.52 (p, *J* = 7.4 Hz, 1H), 4.21 (s, 1H), 3.73 (dq, *J* = 8.8, 6.3 Hz, 1H), 3.59 (td, *J* = 10.6, 4.5 Hz, 1H), 3.49 (d, *J* = 8.7 Hz, 1H), 3.31 (brs, 1H), 3.09 (dd, *J* = 11.7, 6.2 Hz, 1H), 2.69 – 2.62 (s, 1H), 2.29 (s, 3H), 2.29 – 2.24 (m, 2H), 2.19 (t, *J* = 11.3 Hz, 1H), 2.17 (s, 3H), 2.11 (td, *J* = 13.0, 4.4 Hz, 1H), 1.80 (dd, *J* = 13.0, 2.1 Hz, 1H), 1.76 (tq, *J* = 8.6, 2.2 Hz, 1H), 1.53 (dd, *J* = 13.1, 9.2 Hz, 1H), 1.27 (d, *J* = 6.9 Hz, 3H), 1.25 (d, *J* = 6.3 Hz, 3H), 1.24 – 1.20 (m, 1H), 0.97 (s, 9H), 0.78 (d, *J* = 7.2 Hz, 3H), 0.24 (s, 3H), 0.22 ppm (s, 3H);

^{13}C NMR (151 MHz, acetone- *d_6*) δ 209.2, 161.0, 151.5, 131.7, 129.0, 127.3, 120.2, 110.9, 110.0, 105.6, 96.2, 78.1, 77.4, 75.7, 71.2, 52.9, 49.8, 49.4, 39.3, 36.5, 35.1, 35.0, 31.7, 30.4, 26.8 (3C), 19.8, 19.2, 18.4, 16.4, 11.1, -2.8, -2.9 ppm;

Optical Rotation: $[\alpha]_D^{25}$ = +53.7° (c = 0.75 g / 100 mL, $CHCl_3$);

HRMS (m/z) calc'd for $C_{34}H_{53}Cl_2N_2O_6Si^+$ $[M+H]^+$ 683.3044, found: 683.3043.

For α-anomer **32:**

Physical State: white foam;

R_f Value: 0.23 (Hexanes/EtOAc 4:1);

^{1}H NMR (600 MHz, acetone-*d_6*) δ 7.02 (d, *J* = 8.6 Hz, 1H), 5.79 (dt, *J* = 10.0, 1.6 Hz, 1H), 5.73 (ddd, *J* = 10.0, 4.7, 2.5 Hz, 1H), 5.02 (t, *J* = 5.7 Hz, 1H), 4.66 (s, 1H), 4.44 (p, *J* = 7.2 Hz, 1H), 4.21 (s, 1H), 3.89 (p, *J* = 6.5 Hz, 1H), 3.47 (d, *J* = 6.3 Hz, 1H), 3.47 (s, 1H), 3.40 (td, *J* = 10.6, 4.7 Hz, 1H), 3.09 (dd, *J* = 11.7, 6.1 Hz, 1H), 2.71 – 2.64 (m, 1H), 2.35 – 2.28 (m, 2H), 2,30 (s, 3H), 2.22 (t, *J* = 11.2 Hz, 1H), 2.19 – 2.16 (m, 1H), 2.18 (s, 3H), 2.10 – 2.06 (m, 1H), 1.95 (dd, *J* = 13.9, 5.8 Hz, 1H), 1.82 – 1.79 (m, 2H), 1.49 – 1.41 (m, 1H), 1.26 (d, *J* = 6.8 Hz, 3H), 1.24 (d, *J* = 6.3 Hz, 3H), 0.91 (s, 9H), 0.79 (d, *J* = 7.1 Hz, 3H), 0.12 (s, 3H), 0.07 (s, 3H).

^{13}C NMR (151 MHz, acetone-*d_6*) δ 209.0, 159.3, 151.4, 132.9, 128.7, 126.3, 120.5, 110.3, 109.9, 105.4, 99.8, 82.5, 76.5, 74.2, 69.4, 52.5, 50.0, 49.2, 39.4, 37.4, 35.4, 35.2, 31.8, 30.3, 26.6 (3C), 20.2, 19.1, 18.2, 16.7, 11.1, -3.3, -3.6.

Optical Rotation: $[\alpha]_D^{25}$ = +45.5° (c = 0.29 g / 100 mL, $CHCl_3$);

HRMS (m/z) calc'd for $C_{34}H_{53}Cl_2N_2O_6Si^+$ $[M+H]^+$ 683.3044, found: 683.3051.

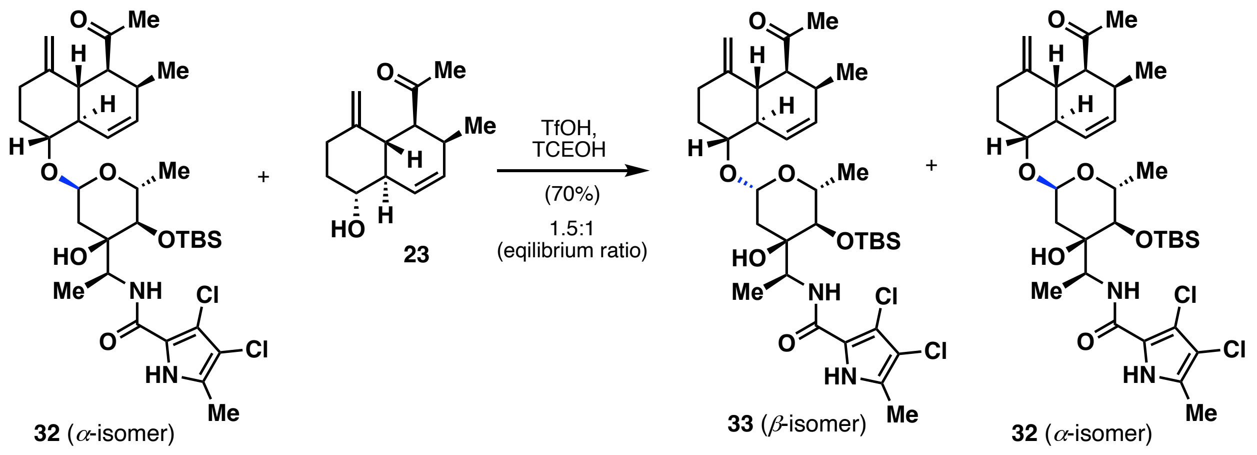

Experimental: A solution of **32** (3.3 mg, 0.0049 mmol), **23** (2.1 mg, 0.0098 mmol, 2.0 eq.), Cl_3CCH_2OH (0.5 µL, 0.0049 mmol, 1.0 eq.) and 1.6 mg 4 A MS in 0.3 mL DCM was stirred at room temperature for 10 min before TfOH (0.8 µL, 0.0098 mmol, 2.0 eq.) was added. After stirred at room temperature for 2.5 h, the reaction mixture was quenched with] $NaHCO_3$ (*sat., aq.,* 10 mL) and extracted with DCM (2 x 10 mL). The combined organic layers were dried over anhydrous Na_2SO_4, filtered, and evaporated. The obtained crude product was purified via column chromatography on SiO_2 (hexanes/EtOAc 10:1 to 6:1 to 3:1) to obtain the corresponding β-anomer **33** (1.4 mg, 42% yield) and recover α-anomer **32** (0.9 mg, 28%).

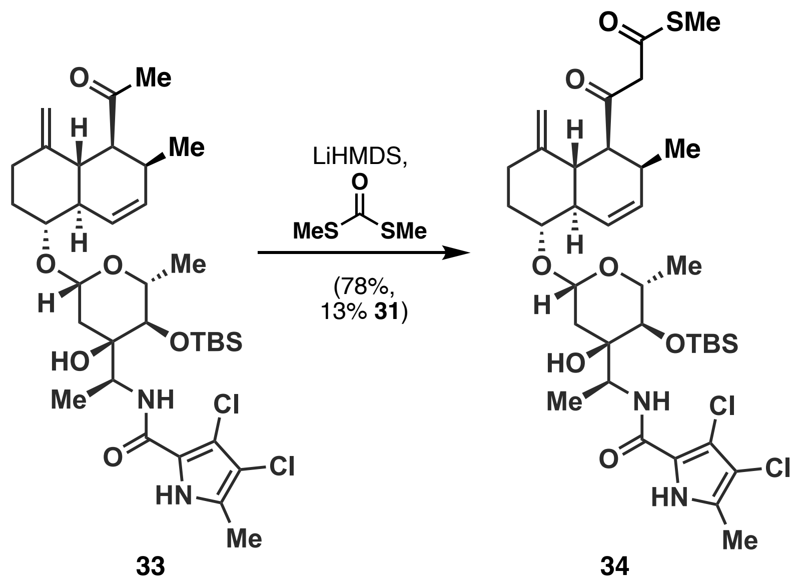

Experimental: To a flame dried vial was added the ketone **33** (30 mg, 0.044 mmol) and THF (2.0 mL) under argon. The solution was cooled to -78 °C, and LiHMDS (1 M solution in THF; 0.88 mL, 0.880 mmol, 20 eq.) was added dropwise. The resulting mixture was stirred at this temperature for 30 min. Thereafter, *S,S'*-dimethyl dithiocarbonate (55 μL, 0.528 mmol, 12.0 eq.) was added dropwise. The reaction was removed from the bath and allowed to warm to room temperature (for about 10 min) before being placed in an oil bath preheated to 30 °C. The reaction was stirred at this temperature for 6 h, after which it was cooled back to room temperature. The reaction was quenched by the addition of NH_4Cl (*sat., aq.,* 6 mL). The aqueous layer was extracted with EtOAc (3 x 12 mL), and the combined organic layers were rinsed with brine, dried over Na_2SO_4, and filtered. The solvent was removed under reduced pressure and the crude material was purified by preparatory thin-layer chromatography (PTLC) (SiO_2; hexanes/EtOAc 3:1), yielding the product **34** (25.9 mg, 78% yield) and recovering start material **33** (4.0 mg, 13%).

Physical State: pale yellow foam;

R_f Value: 0.60 (hexanes/EtOAc 3:1);

^{1}H NMR (600 MHz, acetone-*d*$_6$, major isomer) δ 10.97 (s, 1H), 6.73 (d, *J* = 8.0 Hz, 1H), 6.03 (dd, *J* = 10.1, 2.0 Hz, 1H), 5.68 (m, 1H), 4.95 – 4.90 (m, 1H), 4.68 (s, 1H), 4.52 (p, *J*

= 7.2 Hz, 1H), 4.28 (s, 1H), 4.01 – 3.90 (m, 1H), 3.77 – 3.68 (m, 1H), 3.59 (td, *J* = 10.8, 4.5 Hz, 1H), 3.49 (d, *J* = 8.7 Hz, 1H), 3.31 (d, *J* = 2.1 Hz, 1H), 3.27 (dd, *J* = 11.6, 6.1 Hz, 1H), 2.78 (m, 1H), 2.32 (s, 3H), 2.30 (s, 3H), 2.28 – 2.25 (m, 1H), 2.20 – 2.17 (m, 1H), 2.11 (td, *J* = 12.2, 11.4, 3.3 Hz, 1H), 1.81 – 1.74 (m, 2H), 1.53 (ddd, *J* = 12.9, 9.4, 2.3 Hz, 1H), 1.27 (d, *J* = 6.9 Hz, 3H), 1.24 (d, *J* = 6.3 Hz, 2H), 0.97 (s, 9H), 0.82 (d, *J* = 7.1 Hz, 2H), 0.23 ppm (d, *J* = 10.9 Hz, 6H);

^{13}C NMR (151 MHz, acetone-*d$_6$*, major isomer) δ 202.8, 192.7, 160.8, 150.7, 131.3, 128.9, 127.2, 120.2, 110.7, 109.9, 106.0, 96.1, 77.9, 77.2, 75.6, 71.1, 57.6, 52.4, 49.8, 49.4, 39.1, 36.5, 35.0, 34.9, 31.3, 26.7 (3C), 19.6, 19.1, 18.3, 16.2, 11.9, 10.9, -3.0, -3.1 ppm;

NOTE: The product has an equilibrium between the keto/enol isomers.

Optical Rotation: $[\alpha]_D^{25}$ = +16.3° (c = 1.00 g / 100 mL, $CHCl_3$);

HRMS (m/z) calc'd for $C_{36}H_{55}Cl_2N_2O_7SSi^+$ $[M+H]^+$ 757.2871, found: 757.2862.

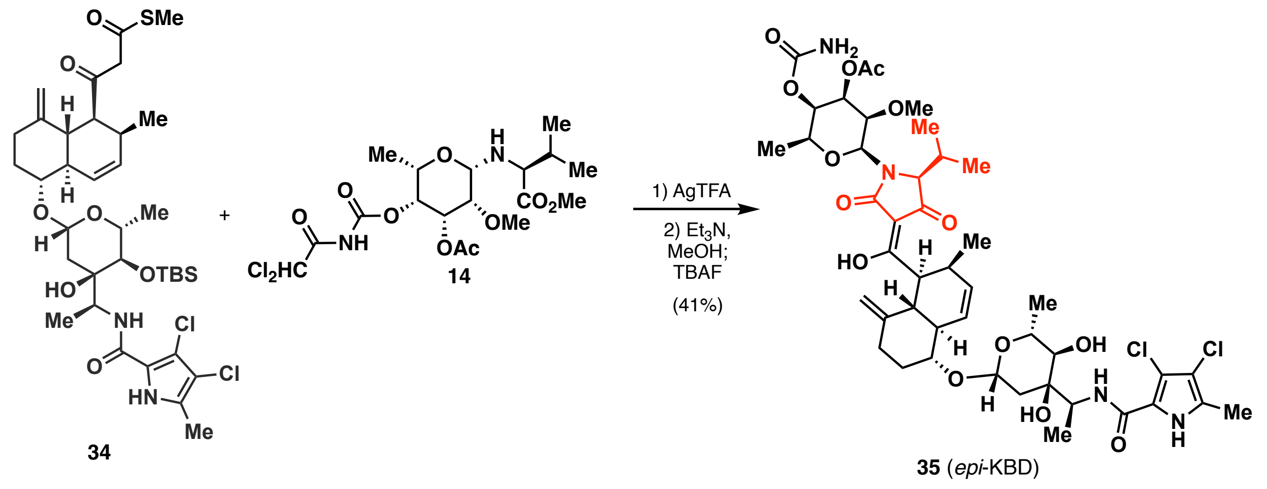

Experimental: To a flame dried vial fixed with a stir bar, and under argon was added the thioester (11.0 mg, 0.014 mmol) and the amino sugar (21.2 mg, 0.044 mmol, 3.0 eq.) as a solution in benzene (1 mL). The benzene was frozen (acetone/dry ice bath) then promptly placed under a high vacuum and left there until all benzene was removed. The fresh-dried 4-A molecular sieves (20 mg) were added to the vial, and it was then evacuated and backfilled with argon. Next, THF (0.2 mL) was added to the vial *via* syringe, and the mixture was stirred for 10 minutes. The vial was wrapped in foil, and $AgCO_2CF_3$ (16.0 mg, 0.073 mmol, 5.0 eq.) was quickly added at once. The reaction was stirred for 2 hours at room temperature after which it was diluted with EtOAc and filtered through a pad of celite. The filtrate was then rinsed with 0.5 M HCl (*aq*., 2 x 10 mL) then brine and $NaHCO_3$ (*sat, aq*., 15 mL). The organic layer was dried over Na_2SO_4 and filtered. The solvent was removed under reduced pressure and the crude material was used in the next step without purification.

The crude material was placed under argon in a flame-dried vial fixed with a stir bar. MeOH (0.8 mL) was added and followed by Et_3N (10 μL, 0.073 mmol, 5 eq.). The reaction was stirred at room temperature for 10 minutes and the solvent was removed quickly under reduced pressure.

The crude mixture above was placed under argon with THF (1 mL). TBAF solution (1.0 M in THF, 0.12 mL, 8 eq.) was added, and the mixture was stirred at room

temperature for 30 minutes. Upon completion, formic acid was added to quench the reaction. The mixture was diluted with acetonitrile (2 mL) and directly purified by prep. HPLC. The eluents containing the product were lyophilized to afford *epi*-KBD **33** (5.6 mg, 41% over 2 steps) as a white solid.

NOTE: The first step is extremely sensitive to H_2O in the reaction. It is necessary to use benzene and fresh dried 4A MS to remove H_2O entirely.

^{1}H NMR (600 MHz, CD_3OD) δ 5.95 (d, *J* = 10.1 Hz, 1H), 5.67 – 5.60 (m, 1H), 5.52 (s, 1H), 5.09 (t, *J* = 3.6 Hz, 1H), 4.98 – 4.95 (m, 1H), 4.59 (s, 1H), 4.44 (s, 1H), 4.41 – 4.35 (m, 2H), 3.90 – 3.85 (m, 1H), 3.85 (d, *J* = 3.0 Hz, 1H), 3.71 – 3.65 (m, 2H), 3.57 (dt, *J* = 10.5, 5.3 Hz, 1H), 3.54 (s, 3H), 3.19 (d, *J* = 9.3 Hz, 1H), 2.71 – 2.63 (m, 2H), 2.31 – 2.24 (m, 3H), 2.23 (s, 3H), 2.17 – 2.09 (m, 1H), 2.06 (s, 3H), 1.85 – 1.77 (m, 2H), 1.59 (dd, *J* = 13.4, 9.3 Hz, 1H), 1.27 (d, *J* = 6.1 Hz, 3H), 1.25 (d, *J* = 7.1 Hz, 3H), 1.19 (d, *J* = 6.3 Hz, 3H), 1.15 (d, *J* = 7.1 Hz, 3H), 0.82 (d, *J* = 7.2 Hz, 3H), 0.77 ppm (d, *J* = 6.8 Hz, 3H);
^{13}C NMR (151 MHz, CD_3OD) δ 197.9, 176.2, 171.2, 161.8, 159.9, 152.3, 133.6, 129.4, 126.6, 120.0, 112.4, 110.7, 106.1, 103.9, 96.9, 80.8, 79.8, 79.6, 76.4, 74.9, 74.1, 73.9, 71.7, 70.4, 68.5, 60.9, 52.4, 50.0, 47.8, 39.4, 38.5, 35.6, 35.3, 31.7, 30.8, 20.9, 19.5, 19.0, 18.6, 16.6, 16.2, 15.8, 10.8 ppm;
NOTE: one ketone's carbon did not show up in NMR.
Optical Rotation: $[\alpha]_D^{25}$ = -26.0° (c = 0.10 g / 100 mL, MeOH);
HRMS (m/z) calc'd for $C_{44}H_{61}Cl_2N_4O_{14}^+$ $[M+H]^+$ 939.3556, found: 939.3560.

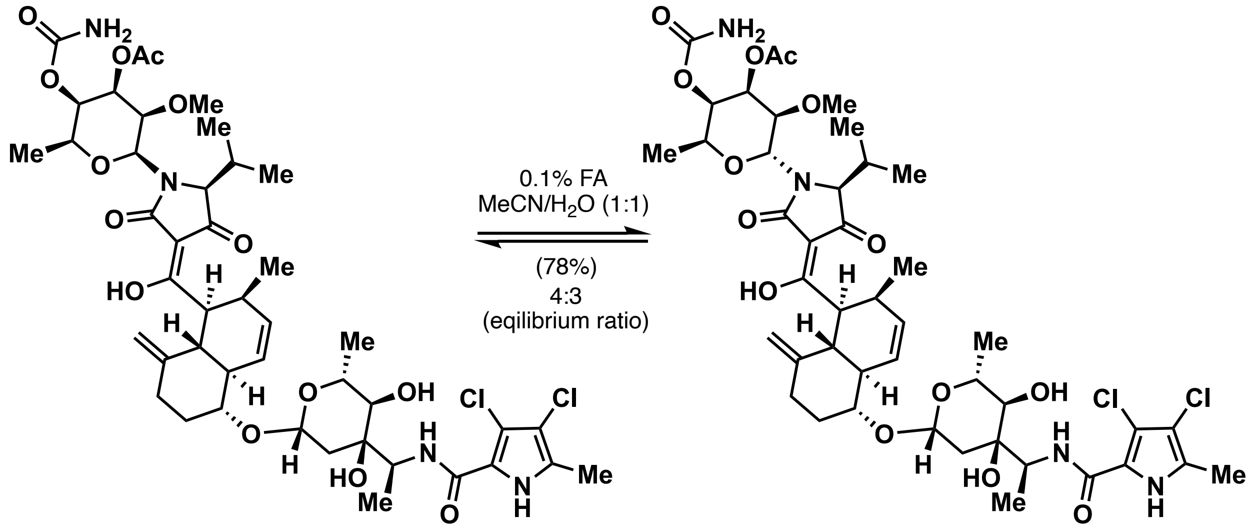

35 (*epi*-KBD) **1b** (KBD/amy)

Experimental: To a vial of purified *epi*-KBD (5.6 mg, 0.006 mmol) was added 1 mL of 0.1% formic acid in acetonitrile/H_2O (1:1). The mixture was shaken well and kept at room temperature for 24 hours. The reaction was monitored by LC-MS until the equilibrium, and the mixture was directly purified by prep. HPLC. The eluents containing *epi*-KBD and KBD were collected separately and lyophilized to afford *epi*-KBD (2.6 mg, 46% yield) and KBD (1.8 mg, 32% yield), respectively. The NMR spectra and Mass fragments are well-matched to the isolated natural product [6] (see **figure S15** to **S17** below).

*NOTE: The mass balance of the reaction was well based on LC-MS results (see **figure S14** below). The major reason of losing material was due to the HPLC purification.*

^{1}H NMR (600 MHz, CD_3OD) δ 5.94 (d, *J* = 10.2 Hz, 1H), 5.90 (t, *J* = 3.4 Hz, 1H), 5.66 – 5.60 (m, 1H), 5.06 (d, *J* = 8.0 Hz, 1H), 4.96 (dd, *J* = 9.5, 2.2 Hz, 1H), 4.92 (dd, *z* = 6.2, 3.5 Hz, 1H), 4.58 (s, 1H), 4.45 (s, 1H), 4.39 (q, *J* = 6.8 Hz, 1H), 4.36 – 4.28 (m, 3H), 3.73 – 3.65 (m, 1H), 3.61 – 3.53 (m, 2H), 3.30 (s, 3H), 3.19 (d, *J* = 9.1 Hz, 1H), 2.66 (s, 1H), 2.32 – 2.24 (m, 3H), 2.23 (s, 3H), 2.19 – 2.13 (m, 2H), 2.12 (s, 3H), 1.84 – 1.77 (m, 2H), 1.58 (dd, *J* = 13.4, 9.4 Hz, 1H), 1.41 (d, *J* = 7.0 Hz, 3H), 1.27 (d, *J* = 6.3 Hz, 3H), 1.25 (d, *J* = 6.9 Hz, 3H), 1.09 (d, *J* = 6.9 Hz, 3H), 0.98 (d, *J* = 6.9 Hz, 3H), 0.82 (d, *J* = 7.2 Hz, 3H).

^{13}**C NMR** (151 MHz, CD_3OD) δ 198.1, 196.7, 178.2, 172.0, 161.7, 158.4, 152.2, 133.7, 129.4, 126.5, 120.0, 112.4, 110.7, 106.2, 105.3, 96.8, 79.6, 77.4, 76.4, 75.6, 74.9, 71.7, 71.4, 69.9, 69.8, 69.6, 57.4, 52.4, 49.9, 48.0, 39.5, 38.5, 35.6, 35.3, 32.1, 32.0, 21.0, 19.0, 18.5, 17.9, 17.7, 16.2, 14.6, 10.8 ppm;

Optical Rotation: $[\alpha]_D^{25}$ = -20.0° (c = 0.23 g / 100 mL, MeOH);

HRMS (m/z) calc'd for $C_{44}H_{61}Cl_2N_4O_{14}^+$ $[M+H]^+$ 939.3556, found: 393.3566.

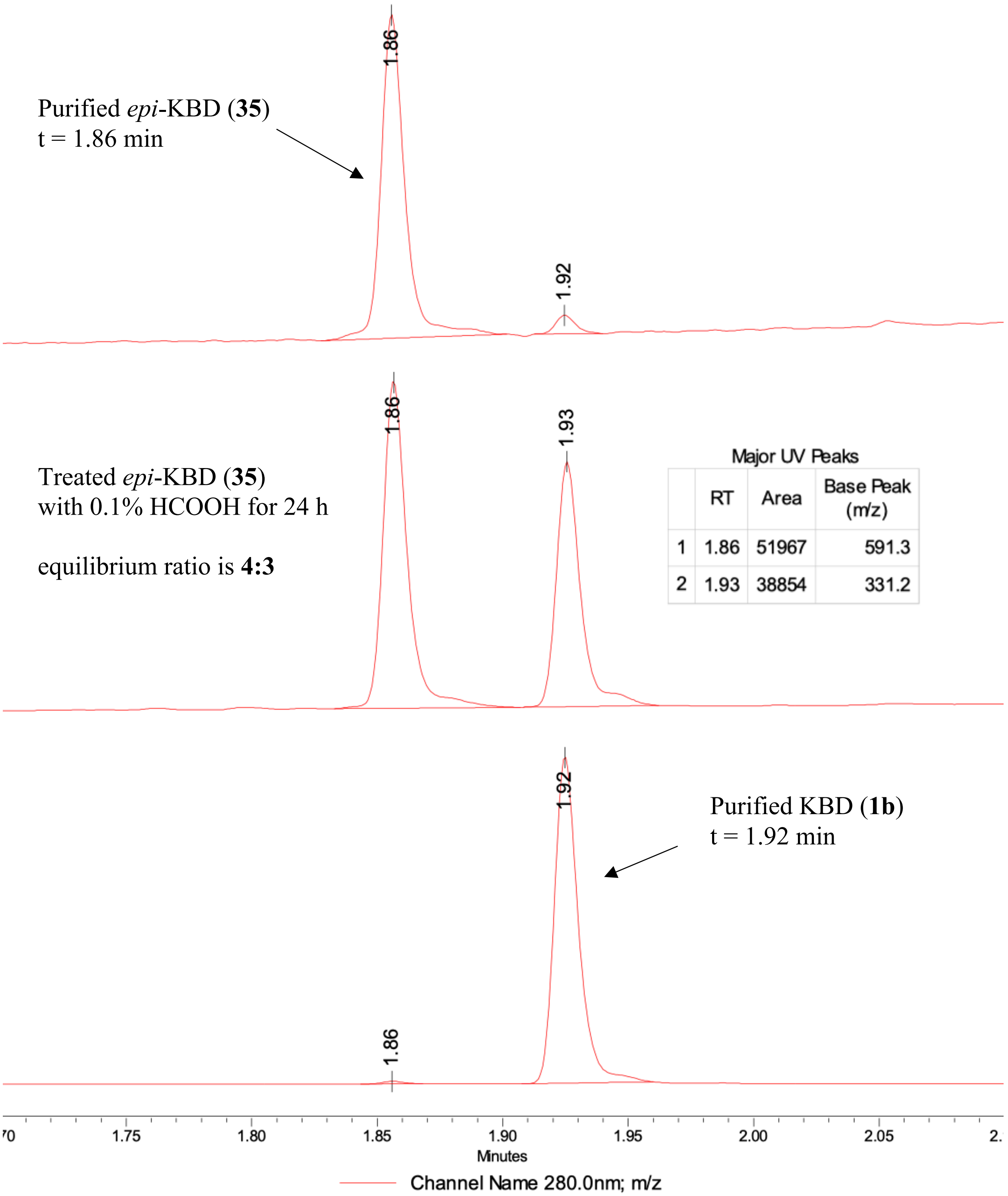

Figure S16. The equilibrium of *epi*-KBD and KBD under 0.1% HCOOH condition

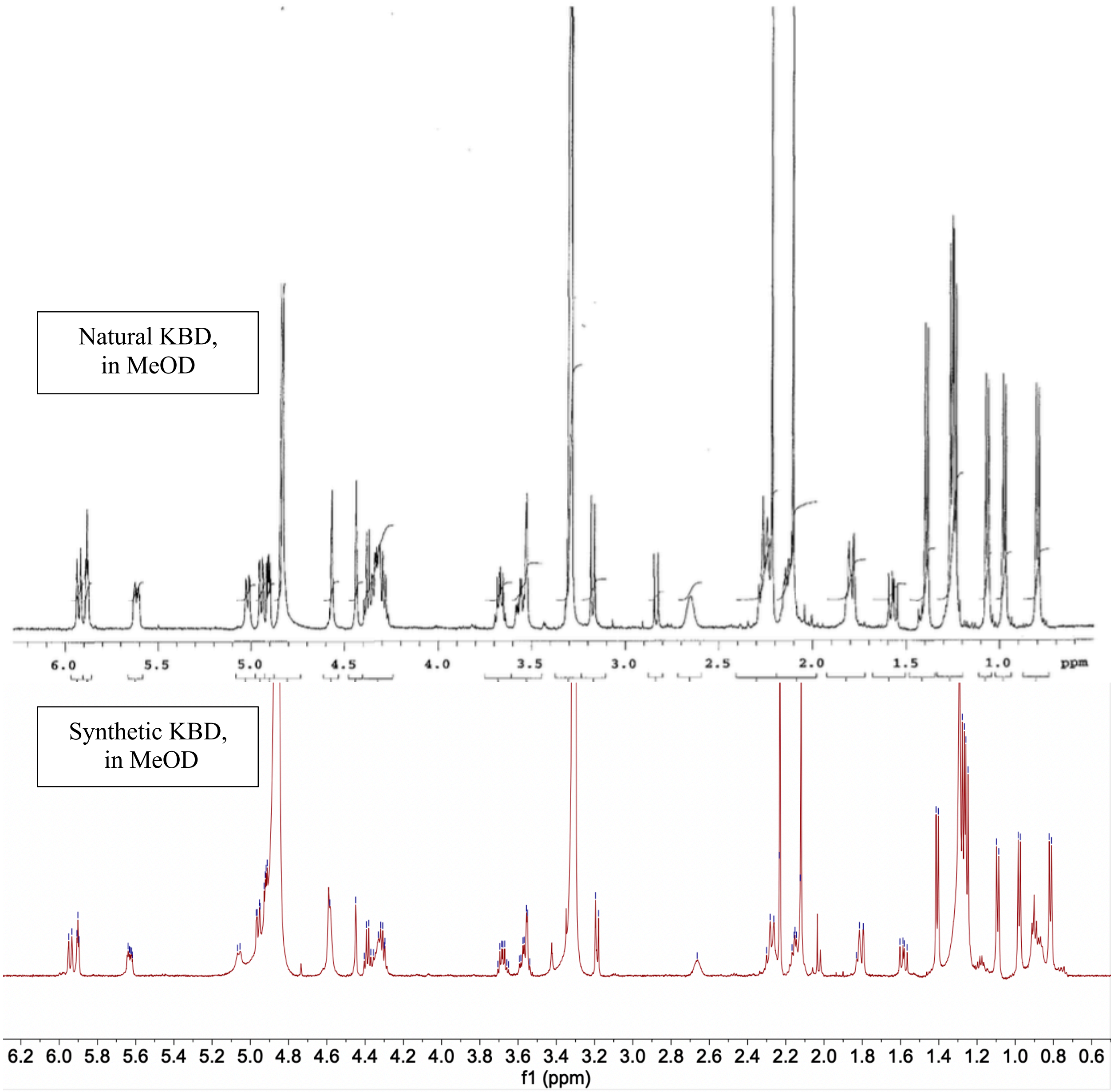

Figure S17. Comparison of ^{1}H NMR of the Natural [6] and Synthetic Kibdelomycin (**1b**)

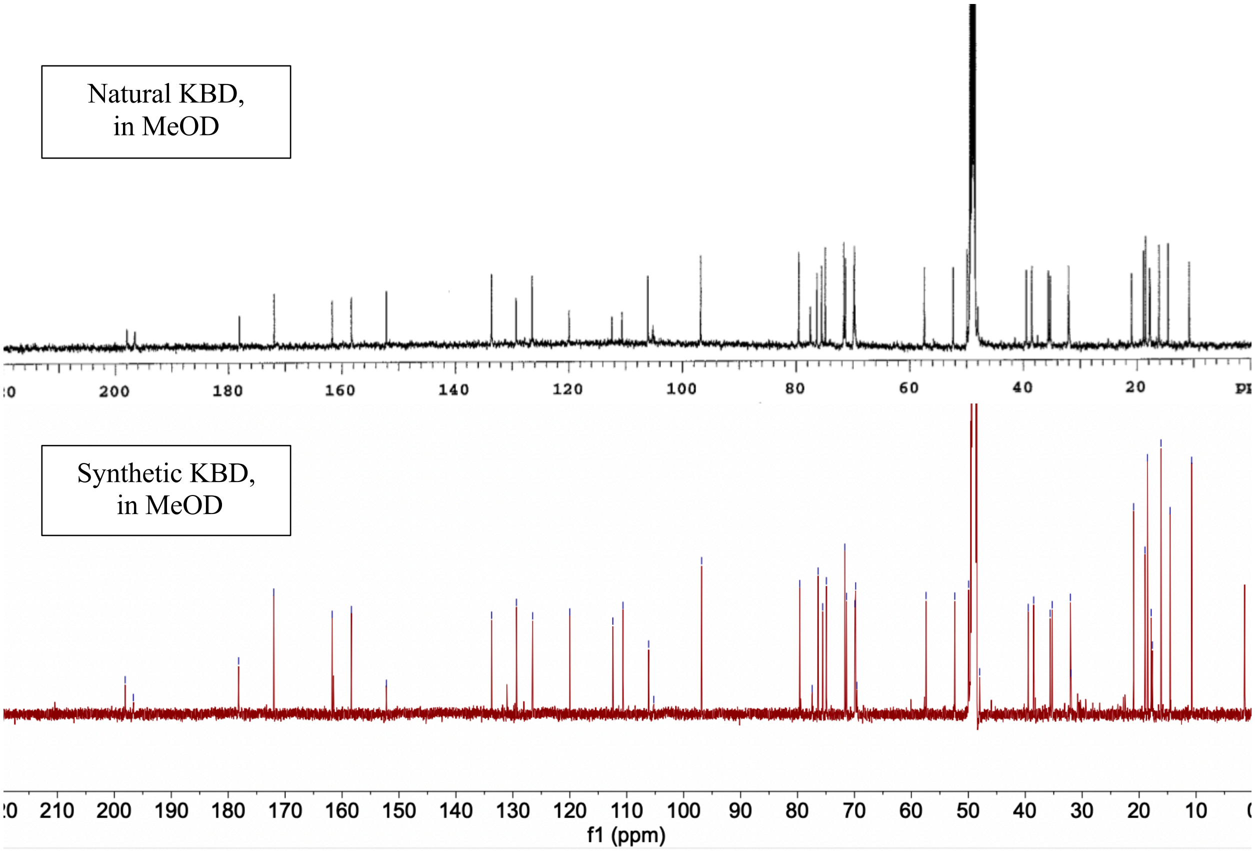

Figure S18. Comparison of ^{13}C NMR of the Natural [6] and Synthetic Kibdelomycin (**1b**)

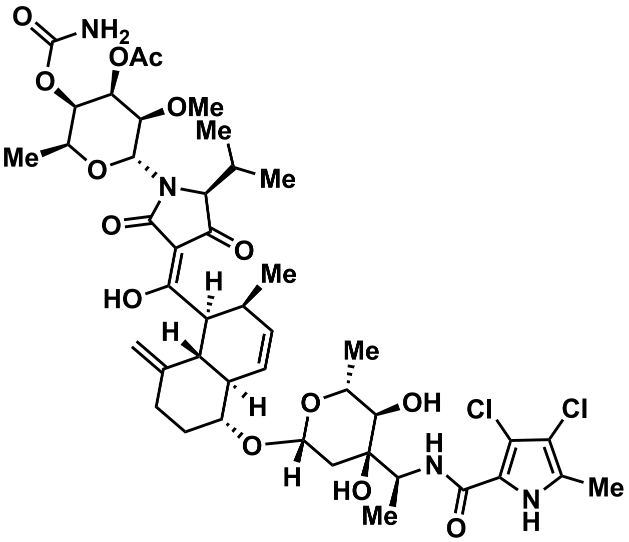

1b (KBD/amy)

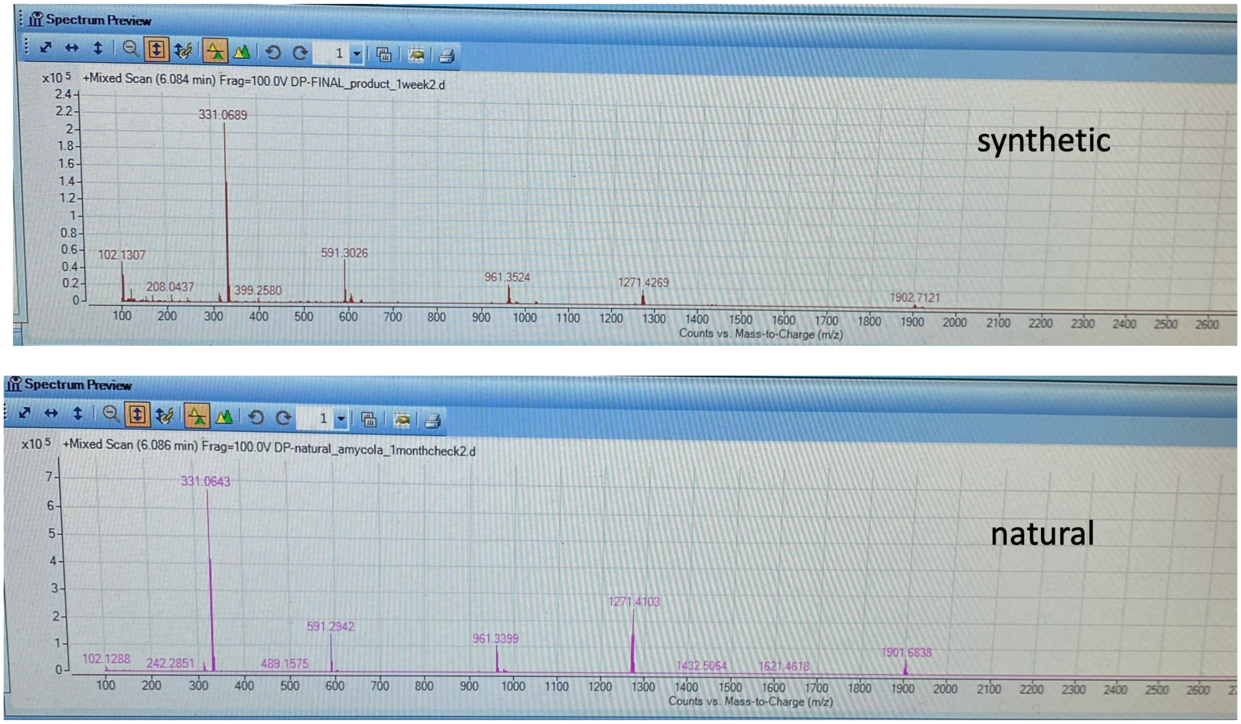

Figure S19. Comparison of the Mass Fragments of the Natural [6] and Synthetic Kibdelomycin (**1b**)

The procedures for analogs synthesis:

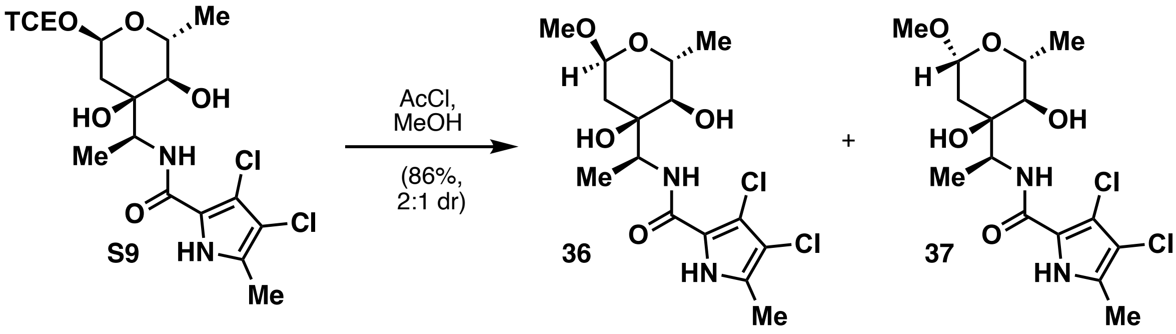

Experimental: To a solution of **S9** (40 mg, 0.115 mmol) in 1.5 mL dry MeOH, AcCl (8.1 μL, 0.115 mmol, 1.0 eq.) was added at room temperature. The stirring was continued for 30 min at room temperature. The reaction mixture was quenched with $NaHCO_3$ (*sat., aq.,* 15 mL), extracted with EtOAc (3 x 15 mL). The combined organic layers were dried over anhydrous Na_2SO_4, filtered, and evaporated. The obtained crude product was purified preparatory thin-layer chromatography (PTLC) (SiO_2; Hexanes/EtOAc 1:2) to obtain the corresponding α-anomer **36** (12.7 mg, 29% yield) and β-anomer **37** (24.9 mg, 57%).

For α-anomer **36:**

Physical State: white foam;

R_f Value: R_f = 0.5 (Hexanes/EtOAc 1:2);

^{1}H NMR (600 MHz, acetone-d_6): δ 7.02 (d, J = 7.8 Hz, 1H), 4.82 (dd, J = 3.8, 1.8 Hz, 1H), 4.31 – 4.25 (m, 1H), 3.79 – 3.73 (m, 1H), 3.34 (s, 3H), 3.20 (d, J = 9.4 Hz, 1H), 2.28 (s, 3H), 1.96 – 1.87 (m, 2H), 1.25 (d, J = 6.2 Hz, 3H), 1.23 ppm (d, J = 6.8 Hz, 3H);

^{13}C NMR (151 MHz, acetone-d_6) δ 159.3, 128.4, 120.7, 110.2, 109.7, 99.0, 75.1, 73.9, 65.9, 55.0, 51.7, 36.2, 18.5, 16.4, 10.9 ppm;

Optical Rotation: $[\alpha]_D^{25}$ = +61.5° (c = 0.4 g / 100 mL, MeOH);

HRMS (m/z) calc'd for $C_{15}H_{22}Cl_2N_2O_5Na^+$ $[M+Na]^+$ 381.0798, found: 403.0799.

For β-anomer **37:**

Physical State: white foam;

R_f Value: 0.55 (Hexanes/EtOAc 1:2);

^{1}H NMR (600 MHz, acetone-*d_6*) δ 11.08 (s, 1H), 6.87 (d, *J* = 7.7 Hz, 1H), 4.65 (dd, *J* = 9.3, 2.1 Hz, 1H), 4.42 (p, *J* = 7.1 Hz, 1H), 4.25 (s, 1H), 3.88 (d, J = 8.8 Hz, 1H), 3.64 (dq, *J* = 9.0, 6.2 Hz, 1H), 3.37 (s, 3H), 3.17 (t, *J* = 8.9 Hz, 1H), 2.29 (s, 3H), 1.82 (dd, *J* = 13.2, 2.1 Hz, 1H), 1.48 (ddd, *J* = 13.2, 9.4, 1.3 Hz, 1H), 1.26 (d, *J* = 6.9 Hz, 3H), 1.24 ppm (d, *J* = 6.2 Hz, 3H);

^{13}C NMR (151 MHz, acetone-*d_6*) δ 160.8, 129.0, 120.0, 111.1, 110.0, 100.5, 76.2, 74.6, 71.1, 56.1, 52.2, 37.3, 18.6, 16.1, 10.9 ppm;

Optical Rotation: $[\alpha]_D^{25}$ = +11.4° (c = 0.48 g / 100 mL, MeOH);

HRMS (m/z) calc'd for $C_{15}H_{23}Cl_2N_2O_5^+$ $[M+H]^+$ 381.0979, found: 381.0968.

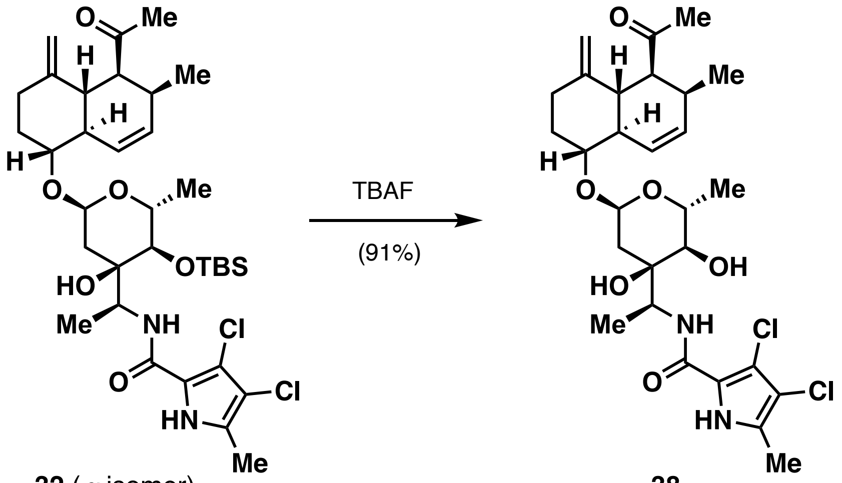

32 (*α*-isomer) **38**

Experimental: To a solution of **32** (45 mg, 0.066 mmol) in 2 mL THF, TBAF (1.0 M in THF, 132 µL, 0.132 mmol, 2.0 eq.) was added at room temperature. After 20 min, the reaction mixture was quenched with NH_4Cl (*sat., aq.,* 15 mL), extracted with EtOAc (3 x 15 mL). The combined organic layers were dried over anhydrous Na_2SO_4, filtered, and evaporated. The obtained crude product was purified preparatory thin-layer chromatography (PTLC) (SiO_2; Hexanes/EtOAc: 1:2) to obtain the corresponding analog **38** (34.1 mg, 91% yield).

Physical State: white foam;

TLC: R_f = 0.45 (Hexanes/EtOAc: 1:2);

^{1}H NMR (600 MHz, acetone-*d6*): δ 7.04 (d, *J* = 7.7 Hz, 1H), 5.81 – 5.72 (m, 2H), 5.07 (dd, *J* = 4.3, 1.8 Hz, 1H), 4.67 (d, *J* = 1.4 Hz, 1H), 4.32 – 4.26 (m, 1H), 4.24 – 4.21 (m, 1H), 4.02 (s, 1H), 3.92 (dd, *J* = 9.4, 6.2 Hz, 1H), 3.46 (d, *J* = 10.0 Hz, 1H), 3.42 (td, *J* = 10.7, 4.7 Hz, 1H), 3.22 (t, *J* = 9.6 Hz, 1H), 3.11 (dd, *J* = 11.7, 6.1 Hz, 1H), 2.73 – 2.65 (m, 1H), 2.38 – 2.34 (m, 1H), 2.33 – 2.30 (m, 1H), 2.28 (s, 3H), 2.24 (t, *J* = 11.2 Hz, 1H), 2.18 (s, 3H), 2.09 – 2.06 (m, 1H), 1.93 (dd, *J* = 14.3, 4.2 Hz, 1H), 1.89 – 1.83 (m, 1H), 1.56 – 1.48 (m, 1H), 1.24 (d, *J* = 4.3 Hz, 3H), 1.23 (d, *J* = 3.8 Hz, 3H), 0.79 ppm (d, *J* = 7.1 Hz, 3H);

^{13}C NMR (151 MHz, acetone- d_6): δ 208.8, 159.3, 151.1, 133.1, 128.4, 125.9, 120.8, 110.2, 109.7, 105.5, 100.4, 83.7, 75.1, 73.8, 66.6, 52.4, 51.6, 49.9, 39.2, 37.0, 36.5, 35.2, 31.6, 30.2, 18.3, 18.0, 16.4, 10.9 ppm;

Optical Rotation: $[\alpha]_D^{25}$ = +114.3° (c = 0.23 g / 100 mL, $CHCl_3$);

HRMS (m/z) calc'd for $C_{28}H_{38}Cl_2N_2O_6Na^+$ $[M+Na]^+$ 591.1999, found: 591.2008.

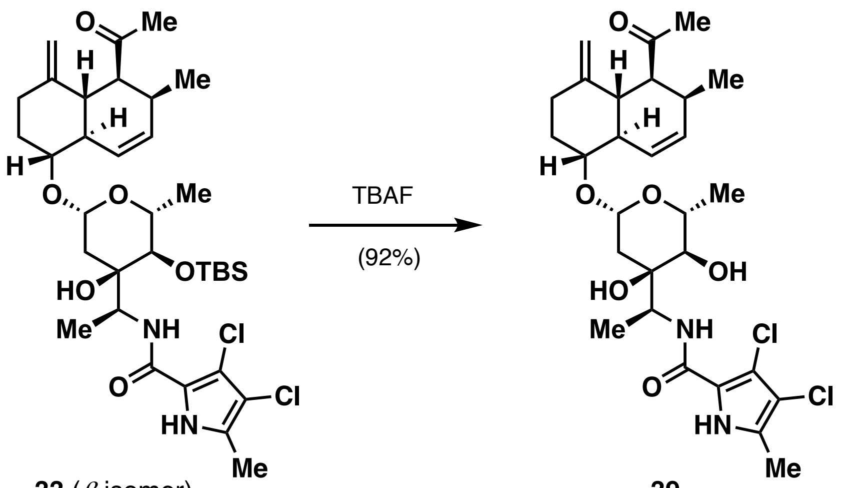

33 (*β*-isomer) **39**

Experimental: To a solution of **33** (33 mg, 0.048 mmol) in 2 mL THF, TBAF (1.0 M in THF, 96 µL, 0.096 mmol, 2.0 eq.) was added at room temperature. After 20 min, the reaction mixture was quenched with NH_4Cl (*sat., aq.,* 15 mL), extracted with EtOAc (3 x 15 mL). The combined organic layers were dried over anhydrous Na_2SO_4, filtered, and evaporated. The obtained crude product was purified preparatory thin-layer chromatography (PTLC) (SiO_2; Hexanes/EtOAc: 1:2) to obtain the corresponding analog **39** (25.3 mg, 92% yield).

Physical State: white foam;

TLC: R_f = 0.45 (Hexanes/EtOAc: 1:2);

^{1}H NMR (600 MHz, acetone-*d6*): δ 6.87 (d, *J* = 7.7 Hz, 1H), 6.02 (dt, *J* = 10.0, 1.8 Hz, 1H), 5.65 (ddd, *J* = 10.1, 4.6, 2.6 Hz, 1H), 4.94 (dd, *J* = 9.3, 2.1 Hz, 1H), 4.66 (s, 1H), 4.46 – 4.39 (m, 1H), 4.32 (s, 1H), 4.21 (s, 1H), 3.82 (d, *J* = 8.9 Hz, 1H), 3.67 – 3.62 (m, 1H), 3.59 (td, *J* = 10.6, 4.4 Hz, 1H), 3.18 (t, *J* = 9.0 Hz, 1H), 3.09 (dd, *J* = 11.7, 6.2 Hz, 1H), 2.70 – 2.63 (m, 1H), 2.32 – 2.25 (m, 5H), 2.20 (d, *J* = 11.2 Hz, 1H), 2.17 (s, 3H), 2.15 – 2.10 (m, 1H), 1.80 – 1.72 (m, 2H), 1.53 (ddd, *J* = 13.2, 9.3, 1.2 Hz, 1H), 1.28 (d, *J* = 6.9 Hz, 3H), 1.23 (d, *J* = 6.2 Hz, 3H), 0.77 ppm (d, *J* = 7.1 Hz, 3H);

^{13}C NMR (151 MHz, acetone-*d6*): δ 207.6, 159.5, 150.1, 130.2, 127.7, 125.9, 118.7, 109.8, 108.6, 104.1, 94.9, 76.5, 75.0, 73.3, 69.8, 51.4, 50.9, 48.0, 37.8, 36.8, 33.7, 33.5, 30.2, 28.8, 17.3, 16.9, 14.8, 9.6 ppm;

Optical Rotation: $[\alpha]_D^{25}$ = +19.7° (c = 0.32 g / 100 mL, $CHCl_3$);

HRMS (m/z) calc'd for $C_{28}H_{39}Cl_2N_2O_6^+$ $[M+H]^+$ 569.2180, found: 569.2189.

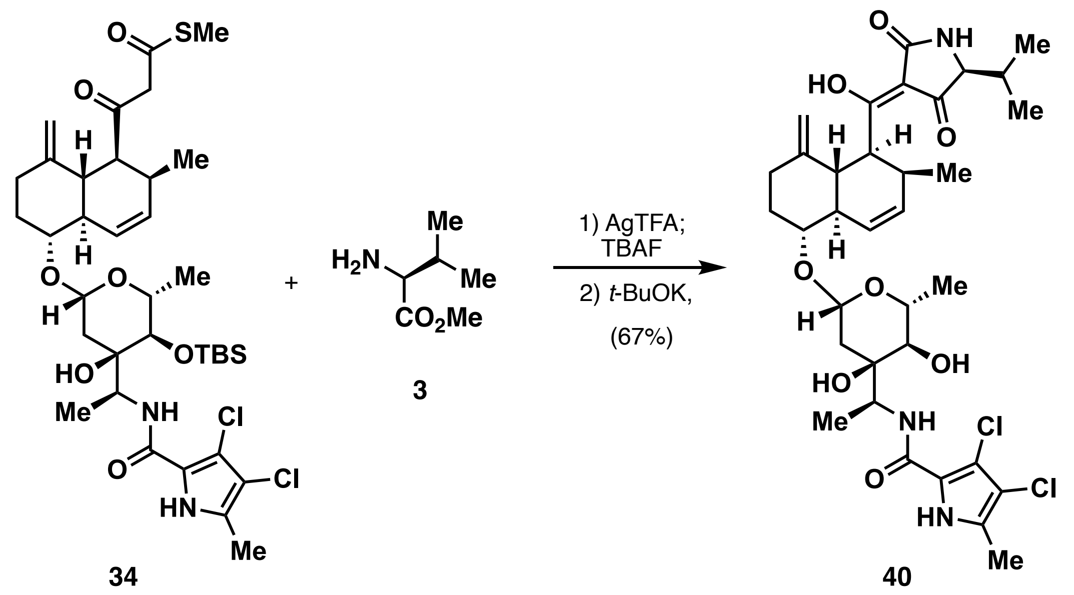

Experimental: A flame-dried vial of the thioester **34** (11.0 mg, 0.014 mmol), the valine methyl ester **3** (5.7 mg, 0.044 mmol, 3.0 eq.), and the fresh-dried 4A MS (10 mg) were evacuated and backfilled with argon. THF (0.4 mL) was added to the vial *via* syringe, and the mixture was stirred for 10 min. The vial was wrapped in foil, and $AgCO_2CF_3$ (16.0 mg, 0.073 mmol, 5.0 eq.) was quickly added at once. The reaction was stirred for 2 h at room temperature, after which it was added TBAF (1.0 M in THF, 44 µL, 0.044 mmol, 3.0 eq.). After 10 min, the reaction mixture was diluted with EtOAc and filtered through a pad of SiO_2. The filtrate was then rinsed two times with 0.5 M HCl (*aq*., 10 mL), then brine and $NaHCO_3$ (*sat., aq*., 10 mL). The organic layer was dried over Na_2SO_4 and filtered. The solvent was removed under reduced pressure and the crude material was quickly passed through a SiO_2 pipet column (Acetone/DCM 1:3), and the semi pure product was directly used in the next step.

The semi pure mixture above was placed under argon with THF (1 mL). *t*BuOK solution (1.0 M in THF, 73 µL, 0.073 mmol, 5 eq.) was added at 0 °C, and the mixture was stirred at the same temperature for 30 min. Upon completion, the reaction was quenched with 0.5M HCl (*aq.*, 15 mL), and the mixture was extracted with EtOAc (3 x 15 mL). The combined organic layers were washed with brine and dried over Na_2SO_4, and filtered. The solvent was removed under reduced pressure and the crude material was

purified by prep. HPLC. The eluents containing the product were lyophilized to afford **40** (6.8 mg, 67% over 2 steps).

^{1}H NMR (600 MHz, CD_3OD) δ 6.01 (d, *J* = 10.1 Hz, 1H), 5.68 (ddd, *J* = 10.0, 4.8, 2.6 Hz, 1H), 4.95 (dd, *J* = 9.5, 2.1 Hz, 1H), 4.70 (d, *J* = 14.2 Hz, 1H), 4.38 (q, *J* = 6.8 Hz, 1H), 4.30 (d, *J* = 10.1 Hz, 1H), 4.26 (t, *J* = 5.2 Hz, 1H), 3.72 – 3.65 (m, 1H), 3.57 (td, *J* = 10.6, 4.6 Hz, 1H), 3.18 (d, *J* = 9.2 Hz, 1H), 2.82 – 2.72 (s, 1H), 2.35 – 2.26 (m, 2H), 2.23 (s, 3H), 2.22 – 2.10 (m, 2H), 1.80 (dd, *J* = 13.3, 2.1 Hz, 1H), 1.58 (dd, *J* = 13.4, 9.4 Hz, 1H), 1.26 (d, *J* = 6.1 Hz, 3H), 1.25 (d, *J* = 6.9 Hz, 3H), 0.97 – 0.90 (m, 6H), 0.82 ppm (dd, *J* = 8.2, 7.1 Hz, 3H);

^{13}C NMR (151 MHz, CD_3OD) δ 208.2, 208.0, 170.3, 167.9, 167.8, 161.7, 150.8, 131.8, 129.4, 127.2 (2C), 120.0, 112.4, 110.6, 106.6 (2C), 96.9, 79.2, 76.3, 74.9, 71.7, 61.5, 53.4, 53.2, 52.4, 49.7 (2C), 39.6, 39.5, 38.5, 35.4, 35.2, 32.7 (2C), 32.0, 31.9, 20.3, 20.2, 18.5, 18.4 (2C), 18.2 (2C), 16.2, 10.8 ppm;

NOTE: The product contains a pair of E/Z isomers on tetramic acid part.

Optical Rotation: $[\alpha]_D^{25}$ = +17.2° (c = 0.29 g / 100 mL, MeOH);

HRMS (m/z): calc'd for $C_{34}H_{46}Cl_2N_3O_8^+$ $[M+H]^+$ 694.2656, found 694.2664.

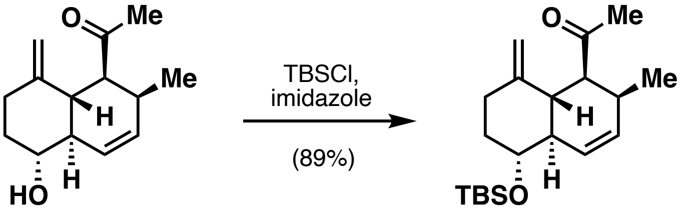

23 S10

Experimental: To a solution of **23** (85 mg, 0.386 mmol, 1.0 equiv) in 3 mL DMF, imidazole (131 mg, 1.93 mmol, 5.0 eq.) and TBSCl (145, 0.966 mmol, 2.5 eq.) were added at 0 °C. The reaction mixture was warmed up to room temperature. After 3 h, the reaction was quenched with NH_4Cl (*sat., aq.,* 25 mL), extracted with EtOAc (3 x 30 mL). The combined organic layers were washed with brine (2 x 50 mL), dried over anhydrous Na_2SO_4, filtered, and evaporated. The obtained crude product was purified by column chromatography (SiO_2; Hexanes/EtOAc 50:1) to obtain the corresponding **S10** (115 mg, 89% yield).

Physical State: colorless oil;

TLC: R_f = 0.2 (Hexanes/EtOAc 50:1);

^{1}H NMR (600 MHz, $CDCl_3$) δ 5.89 – 5.83 (m, 1H), 5.68 (ddd, *J* = 10.1, 4.8, 2.6 Hz, 1H), 4.70 (s, 1H), 4.16 (s, 1H), 3.44 (td, *J* = 10.3, 4.6 Hz, 1H), 3.05 (dd, *J* = 11.7, 6.1 Hz, 1H), 2.66 – 2.57 (m, 1H), 2.34 – 2.27 (m, 1H), 2.21 – 2.14 (s, 5H), 2.06 (dtd, *J* = 12.2, 4.8, 2.3 Hz, 1H), 1.85 – 1.77 (m, 1H), 1.45 (dddd, *J* = 13.8, 12.5, 10.6, 4.9 Hz, 1H), 0.89 (s, 9H), 0.82 (d, *J* = 7.1 Hz, 3H), 0.07 (s, 3H), 0.06 ppm (s, 3H);

^{13}C NMR (151 MHz, $CDCl_3$) δ 210.4, 150.4, 131.2, 126.5, 105.1, 74.5, 52.3, 50.6, 38.3, 34.8, 31.3, 30.3, 26.0 (3C), 18.2, 18.0, -3.9 (2C), -4.5 ppm;

Optical Rotation: $[\alpha]_D^{25}$ = +62.0° (c = 0.64 g / 100 mL, $CHCl_3$);

HRMS (m/z): calc'd for $C_{20}H_{35}O_2Si^+$ $[M+H]^+$ 335.2401, found 335.2400.

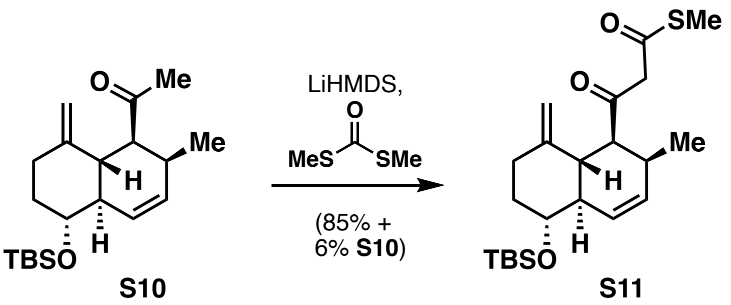

Experimental: To a solution of **S10** (112 mg, 0.335 mmol) in 3 mL THF at -78 °C, LiHMDS (1.0 M in THF, 2.0 mL, 2.0 mmol, 6.0 eq.) was added dropwise. The resulting solution was allowed to stir at this temperature for 30 min. After this period, *S,S'*-dimethyl dithiocarbonate (140 μL, 0.1.34 mmol, 4.0 eq.) was added dropwise. The reaction was removed from the bath and allowed to warm to room temperature (for about 10 min) before being placed in an oil bath preheated to 30 °C. The reaction was stirred at this temperature for 8 h, after which it was cooled back to room temperature. The reaction was quenched by the addition of NH_4Cl (*sat., aq.,* 6 mL). The aqueous layer was extracted three times with EtOAc (3 x 15 mL), and the combined organic layers were rinsed with brine, dried over Na_2SO_4, and filtered. The solvent was removed under reduced pressure and the crude material was purified by column chromatography (SiO_2; Hexanes/EtOAc 60:1) to obtain the corresponding **S11** (116 mg, 85% yield) and recover **S10** (8.2 mg, 6% yield).

Physical State: pale yellow oil;

TLC: R_f = 0.25 (Hexanes/EtOAc 50:1);

^{1}H NMR (600 MHz, acetone-*d6*) δ 5.91 – 5.87 (m, 1H), 5.77 – 5.73 (m, 1H), 4.71 (s, 0.34 H), 4.67 (s, 0.7H), 4.61 (s, 0.34H), 4.28 (s, 0.7H), 4.00 – 3.89 (m, 1H), 3.55 (td, *J* = 10.4, 4.6 Hz, 1H), 3.25 (dd, *J* = 11.7, 6.0 Hz, 0.7H), 2.83 – 2.75 (m, 2H), 2.33 (s, 1H), 2.31 (s, 2H), 2.30 – 2.09 (m, 4H), 1.82 – 1.73 (m, 1H), 1.45 – 1.36 (m, 1H), 0.93 (d, *J* = 7.1 Hz, 1H), 0.91 (s, 3H), 0.91 (s, 6H), 0.81 (d, *J* = 7.1 Hz, 2H), 0.12 – 0.08 ppm (m, 6H);

^{13}C NMR (151 MHz, acetone-*d6*) δ 202.9, 192.7, 151.1, 150.9, 132.4, 132.0, 126.8, 126.7, 105.6, 101.2, 75.2, 75.1, 57.6, 52.2, 52.1, 51.6, 45.3, 39.3 (2C), 38.8, 37.9, 35.4, 35.2, 31.4, 26.2 (6C), 18.6, 18.2, 18.1, 11.9, 10.7, -3.8 (4C), -4.5, -4.6 ppm;

NOTE: contains 2:1 keto/enol isomers.

Optical Rotation: $[\alpha]_D^{25}$ = +51.0° (c = 0.87 g / 100 mL, $CHCl_3$);

HRMS (m/z): calc'd for $C_{22}H_{37}O_3SSi^+$ $[M+H]^+$ 409.2227, found 409.2229.

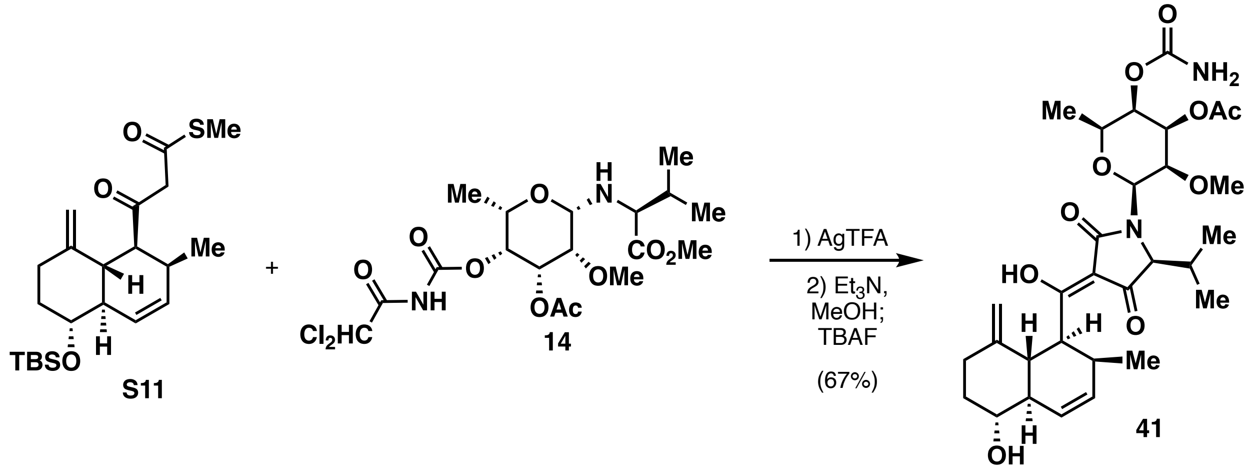

Experimental: To a flame dried vial fixed with a stir bar, and under argon was added the thioester **S11** (22.0 mg, 0.054 mmol) and the amino sugar **14** (78.6 mg, 0.162 mmol, 3.0 eq.) as a solution in benzene (1 mL). The benzene was frozen (acetone/dry ice bath) then promptly placed under a high vacuum and left there until all benzene was removed. The fresh-dried 4A MS (20 mg) were added to the vial, and it was then evacuated and backfilled with argon. Next, THF (0.4 mL) was added to the vial *via* syringe, and the mixture was stirred for 10 min. The vial was wrapped in foil, and $AgCO_2CF_3$ (59 mg, 0.270 mmol, 5.0 eq.) was quickly added at once. The reaction was stirred for 2 h at room temperature after which it was diluted with EtOAc and filtered through a pad of celite. The filtrate was then rinsed two times with 0.5 M HCl (*aq.,* 2 x 15 mL), then brine and $NaHCO_3$ (sat, aq., 10 mL). The organic layer was dried over Na_2SO_4, and filtered. The solvent was removed under reduced pressure and the crude material was used in the next step without purification.

The above crude material was placed under argon in a flame-dried vial fixed with a stir bar. MeOH (0.8 mL) was added and followed by Et_3N (37 μL, 0.270 mmol, 5 eq.). The reaction was stirred at room temperature for 10 min and the solvent was removed quickly under reduced pressure.

The crude mixture above was placed under argon with THF (1 mL). TBAF solution (1.0 M in THF, 0.43 mL, 0.43 mmol, 8 eq.) was added, and the mixture was stirred at room temperature for 30 min. Upon completion, formic acid was added to quench the reaction. The mixture was diluted with acetonitrile (2 mL) and directly purified by prep.

HPLC. The eluents containing the product were lyophilized to afford **41** (21.3 mg, 67% over 2 steps) as a white solid.

NOTE: The first step is extremely sensitive to H_2O in the reaction. It is necessary to use benzene and fresh dried 4A MS to remove H_2O entirely.

^{1}H NMR (600 MHz, CD_3OD) δ 5.92 (dt, *J* = 10.2, 1.7 Hz, 1H), 5.71 (ddd, *J* = 10.1, 4.7, 2.6 Hz, 1H), 5.53 (s, 1H), 5.10 (t, *J* = 3.5 Hz, 1H), 4.97 (dt, *J* = 3.8, 1.4 Hz, 1H), 4.60 (s, 1H), 4.41 (s, 1H), 4.29 (dd, *J* = 11.9, 6.1 Hz, 1H), 3.96 (s, 1H), 3.89 (dd, *J* = 6.4, 1.8 Hz, 1H), 3.72 (d, *J* = 3.1 Hz, 1H), 3.55 (s, 3H), 3.40 – 3.33 (m, 1H), 2.76 – 2.60 (m, 2H), 2.32 – 2.24 (m, 1H), 2.23 – 2.15 (m, 1H), 2.15 – 2.09 (m, 1H), 2.06 (s, 3H), 1.77 (td, *J* = 10.6, 2.3 Hz, 1H), 1.40 (dt, *J* = 12.2, 6.2 Hz, 1H), 1.20 (d, *J* = 6.4 Hz, 3H), 1.16 (d, *J* = 7.2 Hz, 3H), 0.85 (d, *J* = 7.2 Hz, 3H), 0.76 ppm (d, *J* = 6.9 Hz, 3H);
^{13}C NMR (151 MHz, CD_3OD) δ 171.5, 159.9, 152.3, 133.8, 126.2, 105.7, 80.8, 79.4, 74.5, 74.0, 70.4, 60.8, 51.8, 49.6, 46.5, 39.0 (2C), 35.9, 32.6, 31.7, 20.8, 19.4, 18.7, 16.6, 15.6 ppm;
Optical Rotation: $[\alpha]_D^{25}$ = -7.9° (c = 0.19 g / 100 mL, MeOH);
HRMS (m/z): calc'd for $C_{30}H_{43}N_2O_{10}^+$ $[M+H]^+$ 591.2913, found 591.2916.

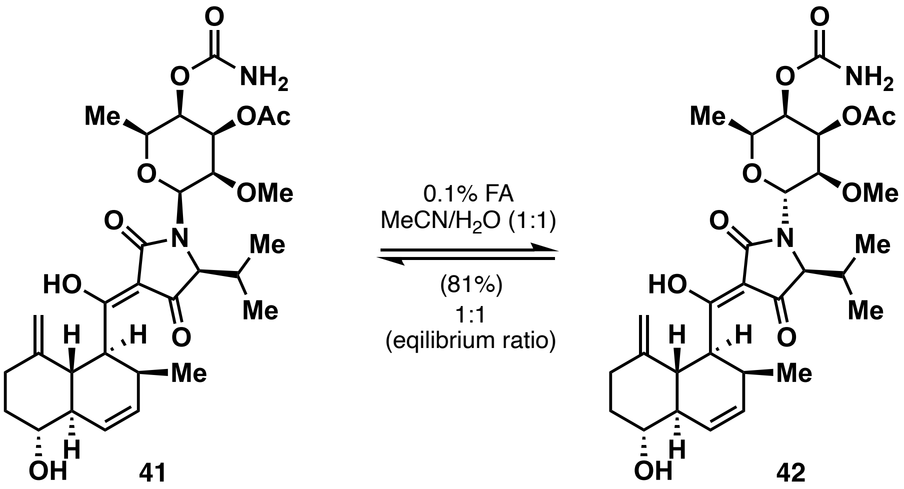

Experimental: To a vial of **41** (9.8 mg, 0.017 mmol) was added 1 mL of 0.1% formic acid in acetonitrile/H_2O (1:1). The mixture was shaken well and kept at room temperature for 24 h. The reaction was monitored by LC-MS until the equilibrium, and the mixture was directly purified by prep. HPLC. The eluents containing **41** and **42** were collected separately and lyophilized to afford **41** (4.0 mg, 41% yield) and **42** (3.9 mg, 40% yield), respectively.

^{1}H NMR (600 MHz, CD_3OD) δ 5.92 – 5.89 (m, 2H), 5.71 (ddd, J = 10.0, 4.7, 2.6 Hz, 1H), 5.07 (d, J = 8.9 Hz, 1H), 4.93 (dd, J = 6.2, 3.4 Hz, 1H), 4.60 (s, 1H), 4.42 (s, 1H), 4.35 – 4.25 (m, 3H), 3.64 (d, J = 2.9 Hz, 1H), 3.36 (dt, J = 10.6, 5.3 Hz, 1H), 3.30 (s, 3H), 2.71 – 2.61 (s, 1H), 2.31 – 2.24 (m, 2H), 2.23 – 2.15 (m, 2H), 2.12 (s, 3H), 2.11 – 2.09 (m, 1H), 1.76 (td, J = 10.4, 2.2 Hz, 1H), 1.41 (d, J = 7.0 Hz, 3H), 1.40 – 1.34 (m, 1H), 1.11 (d, J = 7.0 Hz, 3H), 0.97 (d, J = 6.9 Hz, 3H), 0.84 ppm (d, J = 7.2 Hz, 3H);

^{13}C NMR (151 MHz, CD_3OD) δ 171.9, 158.3, 152.3, 134.0, 126.1, 105.8, 77.3, 75.6, 74.6, 71.5, 69.8, 69.7, 57.4, 51.9, 47.0, 39.2, 39.0, 35.9, 32.4, 32.0, 21.0, 18.7, 18.0, 17.3, 14.5 ppm;

Optical Rotation: $[\alpha]_D^{25}$ = -8.8° (c = 0.16 g / 100 mL, MeOH);

HRMS (m/z): calc'd for $C_{30}H_{43}N_2O_{10}^+$ $[M+H]^+$ 591.2913, found 591.2913.

II) Chiral SFC Results

Compound S6

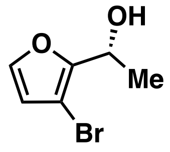

(92% *ee*)

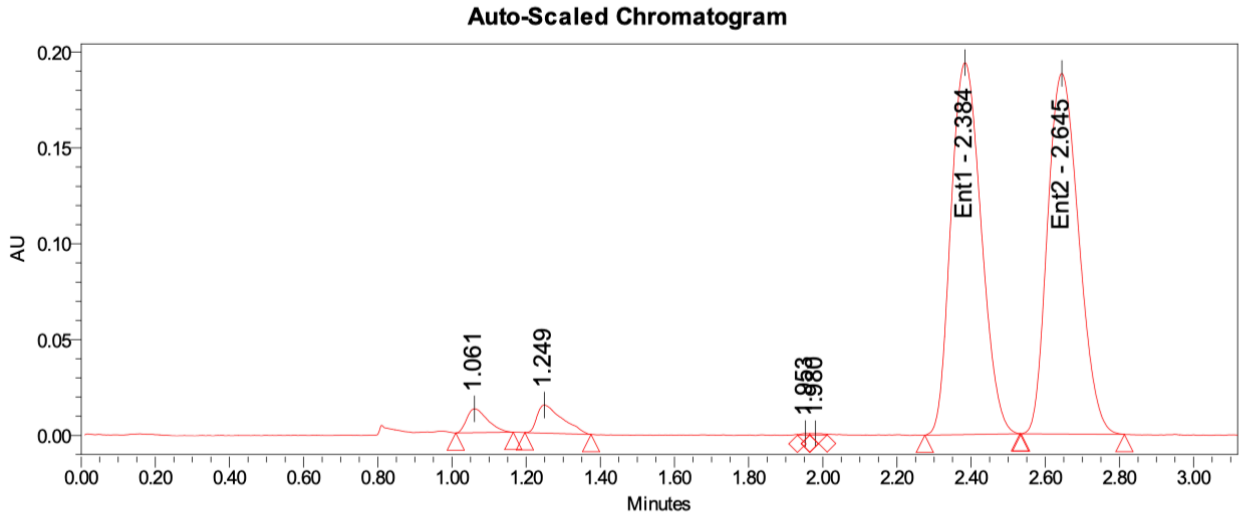

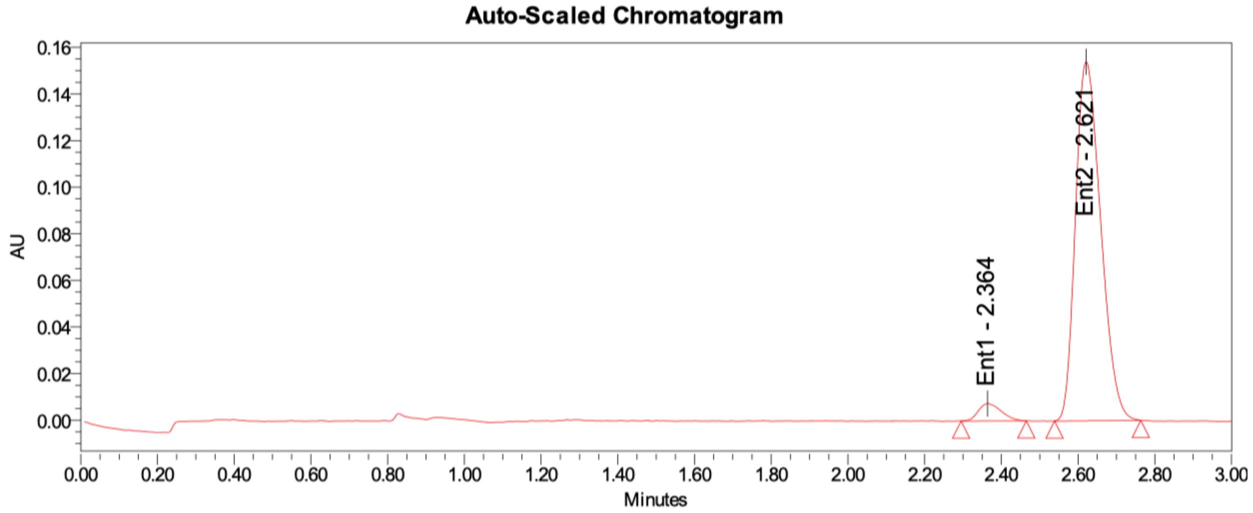

Area Summarized by Name

JT1-020-P	50.02	49.98	0.04	1100965	1100185
JT1-027-P	4.22	95.78	-91.57	31008	704352
JT1-026-P	4.06	95.94	-91.88	21991	519563

Compound 23

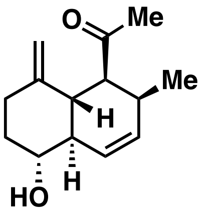

(99% *ee*)

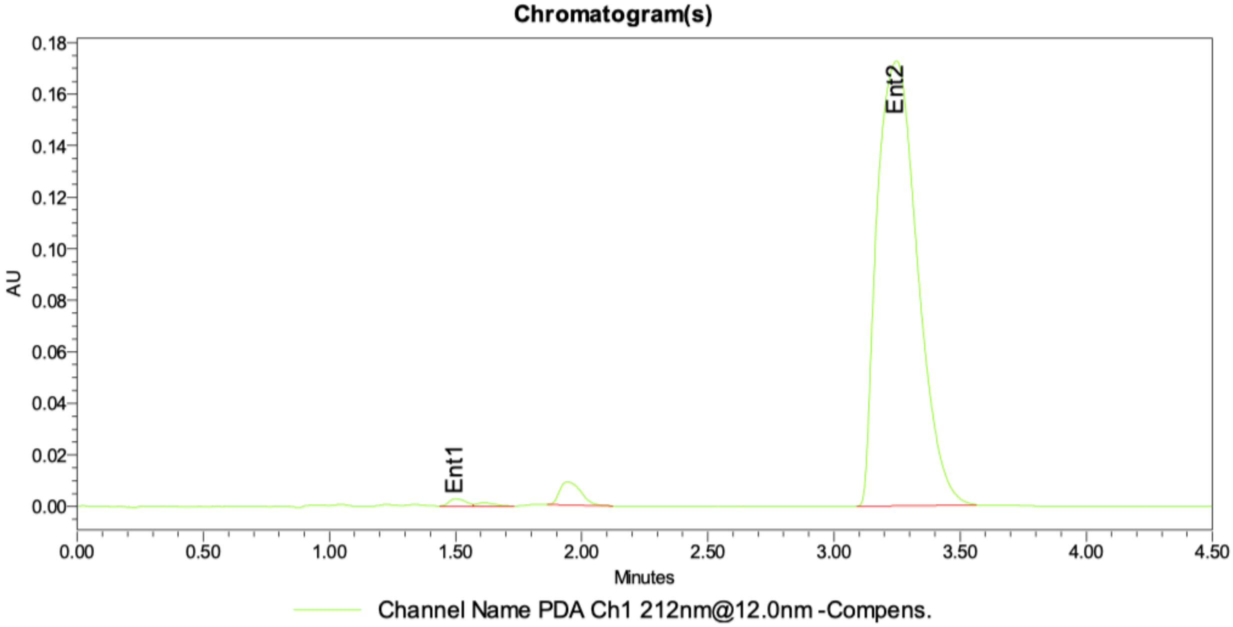

Peak Info

	Channel Name	Name	RT	Area	Height (µV)	ent1	ent2	ee
1	PDA Ch1 212nm@12.0nm -Compens.	Ent1	1.50	12459	2798	0.64	99.36	-98.71
2	PDA Ch1 212nm@12.0nm -Compens.		1.61	5935	1223	0.64	99.36	-98.71
3	PDA Ch1 212nm@12.0nm -Compens.		1.94	52939	9044	0.64	99.36	-98.71
4	PDA Ch1 212nm@12.0nm -Compens.	Ent2	3.25	1920511	172689	0.64	99.36	-98.71

III) X-ray data of **23** (CCDC# 2086623)

The single crystal X-ray diffraction studies were carried out on a Bruker SMART Apex II CCD diffractometer equipped with Cu K_α radiation (λ = 1.54178). Crystals of the subject compound were used as received (grown from Ethylacetate and Pentane). A 0.280 x 0.12 x 0.110 mm colorless block crystal was mounted on a Cryoloop with Paratone oil.

Data were collected in a nitrogen gas stream at 100(2) K using ϕ and ϖ scans. Crystal-to-detector distance was 40 mm using exposure time 2.0, 3.0, and 6.0 second (depending on the 2θ position) with a scan width of 1.40°. Data collection was 100.0% complete to 67.679° in θ. A total of 20851 reflections were collected. 2353 reflections were found to be symmetry independent, with a R_{int} of 0.0353. Indexing and unit cell refinement indicated a **Primitive Orthorhombic** lattice. The space group was found to be $\boldsymbol{P2_12_12_1}$**.** The data were integrated using the Bruker SAINT Software program and scaled using the SADABS software program. Solution by direct methods (SHELXT) produced a complete phasing model consistent with the proposed structure.

All nonhydrogen atoms were refined anisotropically by full-matrix least-squares (SHELXL-2014). All carbon bonded hydrogen atoms were placed using a riding model. Their positions were constrained relative to their parent atom using the appropriate HFIX command in SHELXL-2014.

Notes: Excellent data and refinement.

Absolute structure parameter 0.07(7) (conclusive)

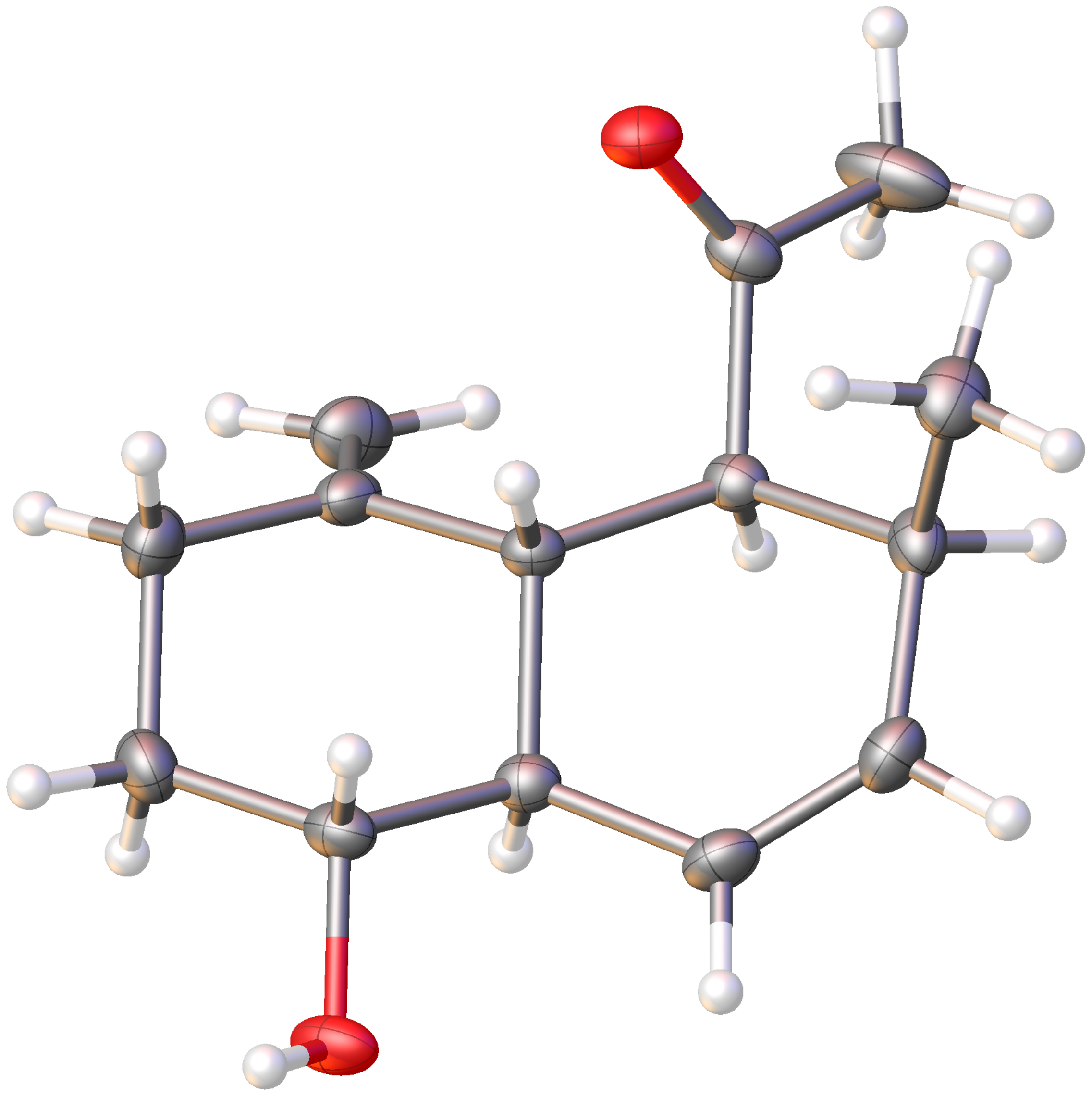

Figure S20. X-ray structure of **23** (CCDC# 2086623)

Table S2. Crystal data and structure refinement for **23**.

Identification code	baran786	
Empirical formula	C14 H20 O2	
Formula weight	220.30	
Temperature	100.0 K	
Wavelength	1.54178 Å	
Crystal system	Orthorhombic	
Space group	$P2_12_12_1$	
Unit cell dimensions	a = 5.0390(2) Å	α= 90°.
	b = 14.3672(6) Å	β= 90°.
	c = 17.1969(8) Å	γ = 90°.
Volume	1244.99(9) $Å^3$	
Z	4	
Density (calculated)	1.175 Mg/m^3	
Absorption coefficient	0.604 mm^{-1}	
F(000)	480	
Crystal size	0.28 x 0.12 x 0.11 mm^3	
Theta range for data collection	4.009 to 70.245°.	
Index ranges	-6<=h<=6, -17<=k<=17, -21<=l<=19	
Reflections collected	20851	
Independent reflections	2353 [R(int) = 0.0353]	
Completeness to theta = 67.679°	100.0 %	
Absorption correction	Semi-empirical from equivalents	
Max. and min. transmission	0.6620 and 0.5685	
Refinement method	Full-matrix least-squares on F^2	
Data / restraints / parameters	2353 / 0 / 148	
Goodness-of-fit on F^2	1.049	
Final R indices [I>2sigma(I)]	R1 = 0.0332, wR2 = 0.0864	
R indices (all data)	R1 = 0.0351, wR2 = 0.0881	
Absolute structure parameter	0.07(7)	
Extinction coefficient	n/a	
Largest diff. peak and hole	0.184 and -0.184 e.$Å^{-3}$	

Table S3. Atomic coordinates (x 10^4) and equivalent isotropic displacement parameters ($Å^2$x 10^3) for **23**. U(eq) is defined as one-third of the trace of the orthogonalized U^{ij} tensor.

	x	y	z	U(eq)
O(1)	3551(3)	7155(1)	5205(1)	26(1)
O(2)	6019(3)	3026(1)	6525(1)	25(1)
C(1)	4519(4)	6221(1)	5285(1)	21(1)
C(2)	4569(4)	5742(1)	4496(1)	26(1)
C(3)	5324(4)	4707(1)	4557(1)	26(1)
C(4)	3666(4)	4224(1)	5164(1)	21(1)
C(5)	3896(3)	4699(1)	5948(1)	17(1)
C(6)	2751(3)	5690(1)	5854(1)	18(1)
C(7)	2438(4)	6178(1)	6620(1)	23(1)
C(8)	2494(4)	5744(1)	7298(1)	24(1)
C(9)	2959(4)	4719(1)	7408(1)	21(1)
C(10)	2621(3)	4183(1)	6626(1)	18(1)
C(11)	2069(4)	3518(1)	5004(1)	28(1)
C(12)	5670(4)	4581(1)	7796(1)	29(1)
C(13)	3789(4)	3212(1)	6732(1)	22(1)
C(14)	2069(4)	2521(1)	7150(2)	40(1)

IV) References:

[1] Qin, T.; Cornella, J.; Li, C.; Malins, L.; Edwards, J. T.; Kawamura, S.; Maxwell, B. D.; Eastgate, M. D.; Baran, P. S. *Science* **2016**, *352*, 801.

[2] Yu, L.-F., Hu, H.-N., Nan, F.-J. J*. Org. Chem.* **2011**, *76*,1448.

[3] Pelly, S. C.; Parkinson, C. J.; van Otterlo, W. A. L.; de Koning, C. B. *J. Org. Chem.* **2005**, *70*, 10474.

[4] Mackman, R. L.; Steadman, V. A.; Dean, D. K. *et. al. J. Med. Chem.* **2018**, *61*, 9473.

[5] Yao, Q.; Yuan, C. *J. Org. Chem.* **2013**, *78*, 6962.

[6] Phillips, J. W.; Goetz, M. A.; Smith, S. K. *et. al. Chem. & Bio.* **2011**, *18*, 955.

V) NMR Spectra

^{1}H NMR of **8**

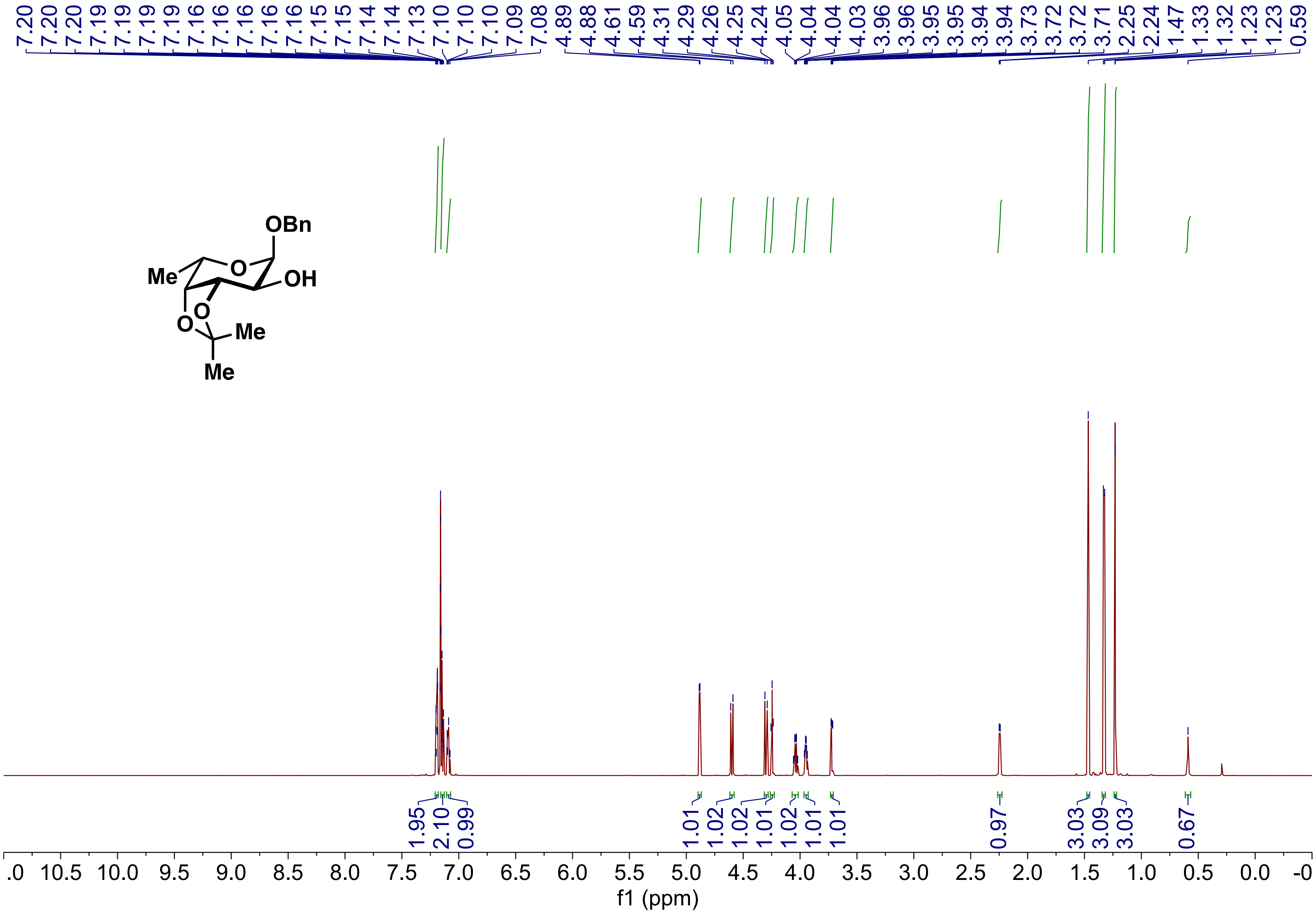
OBn
Me
O
OH
O
O
Me
Me
7.20 7.20 7.20 7.19 7.19 7.19 7.19 7.16 7.16 7.16 7.16 7.16 7.15 7.15 7.14 7.14 7.13 7.10 7.10 7.10 7.09 7.08 4.89 4.88 4.61 4.59 4.31 4.29 4.26 4.25 4.24 4.05 4.04 4.04 4.03 3.96 3.96 3.95 3.95 3.94 3.94 3.73 3.72 3.72 3.71 2.25 2.24 1.47 1.33 1.32 1.23 1.23 0.59
1.95 2.10 0.99 1.01 1.02 1.02 1.01 1.02 1.01 1.01 0.97 3.03 3.09 3.03 0.67
.0 10.5 10.0 9.5 9.0 8.5 8.0 7.5 7.0 6.5 6.0 5.5 5.0 4.5 4.0 3.5 3.0 2.5 2.0 1.5 1.0 0.5 0.0 -0
f1 (ppm)

^{13}C NMR of **8**

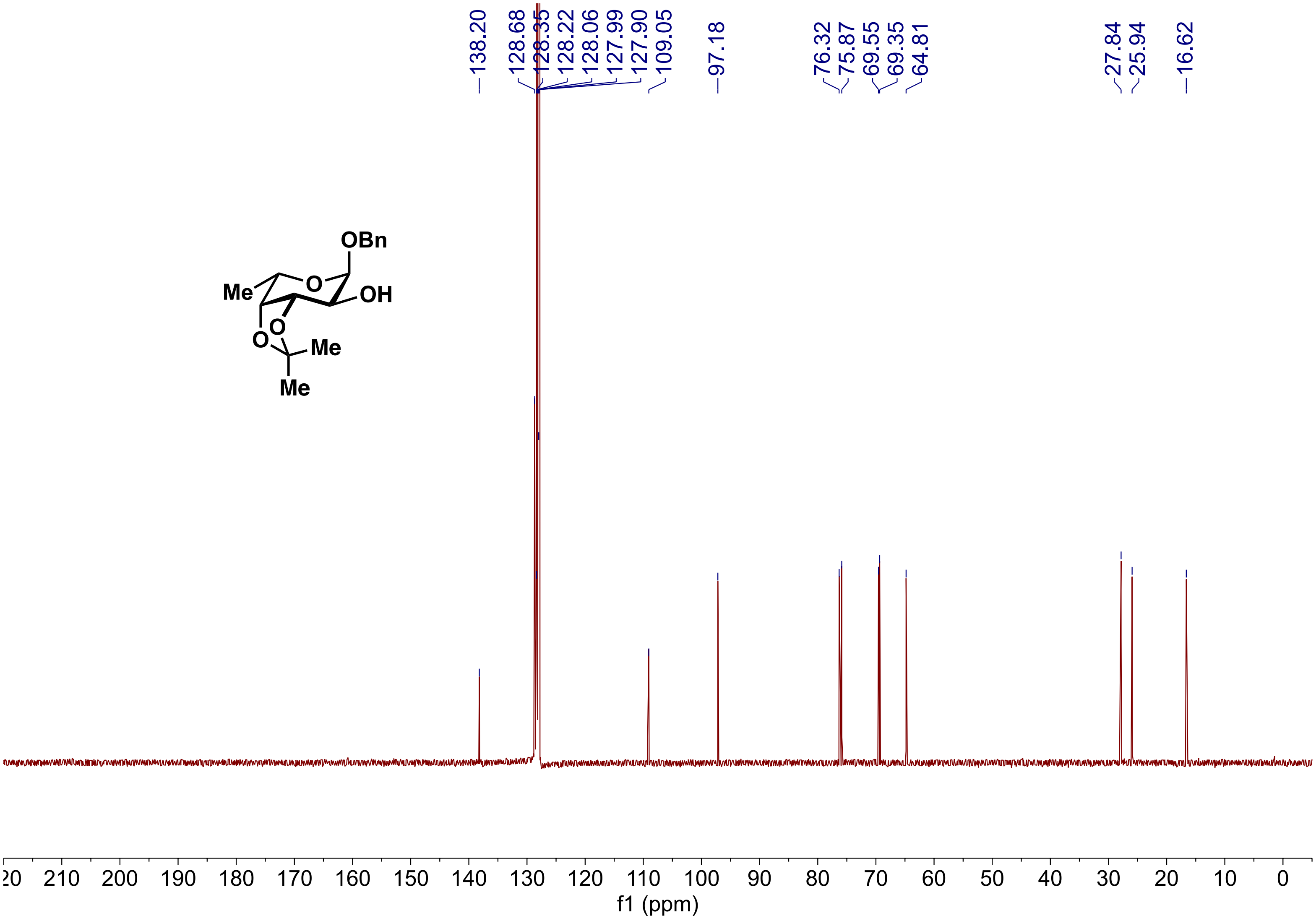

138.20
128.68
128.35
128.22
128.06
127.99
127.90
109.05
97.18
76.32
75.87
69.55
69.35
64.81
27.84
25.94
16.62
OBn
Me
O
OH
O
O
Me
Me
20 210 200 190 180 170 160 150 140 130 120 110 100 90 80 70 60 50 40 30 20 10 0
f1 (ppm)

^{1}H NMR of **9**

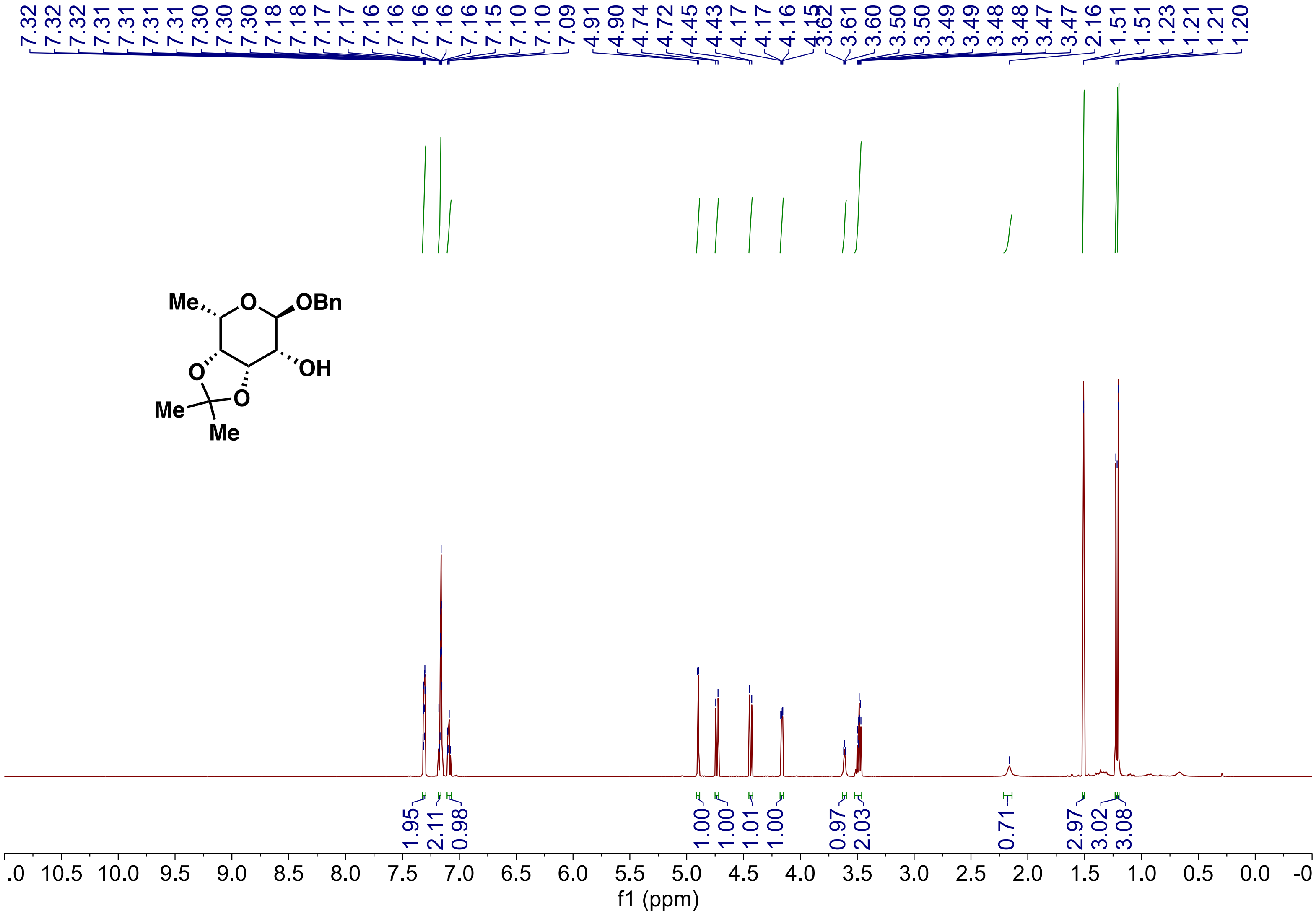

^{13}C NMR of **9**

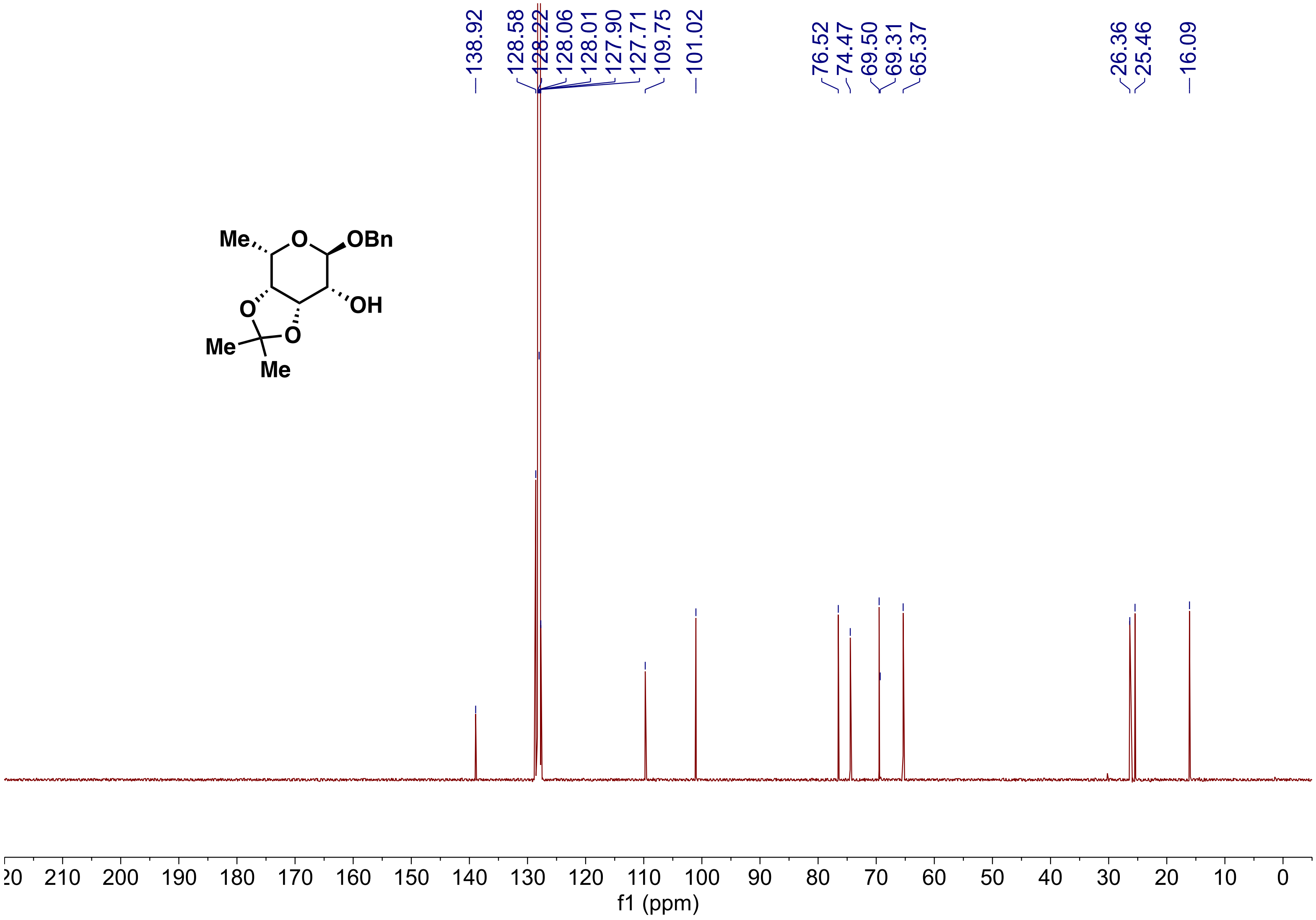

138.92
128.58
128.22
128.06
128.01
127.90
127.71
109.75
101.02
76.52
74.47
69.50
69.31
65.37
26.36
25.46
16.09
Me
O
OBn
OH
O
O
Me
Me
20 210 200 190 180 170 160 150 140 130 120 110 100 90 80 70 60 50 40 30 20 10 0
f1 (ppm)

^{1}H NMR of **10**

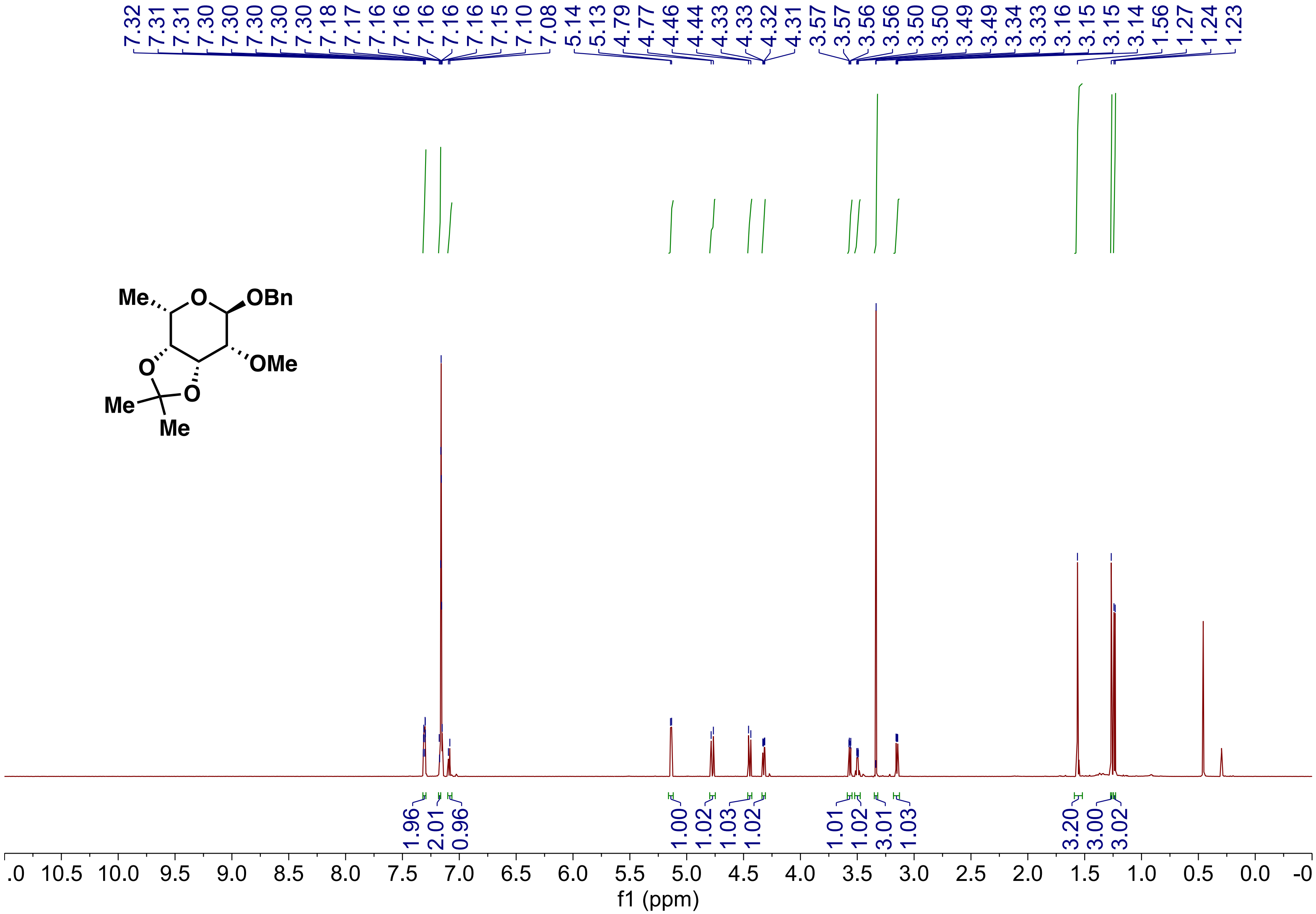

^{13}C NMR of **10**

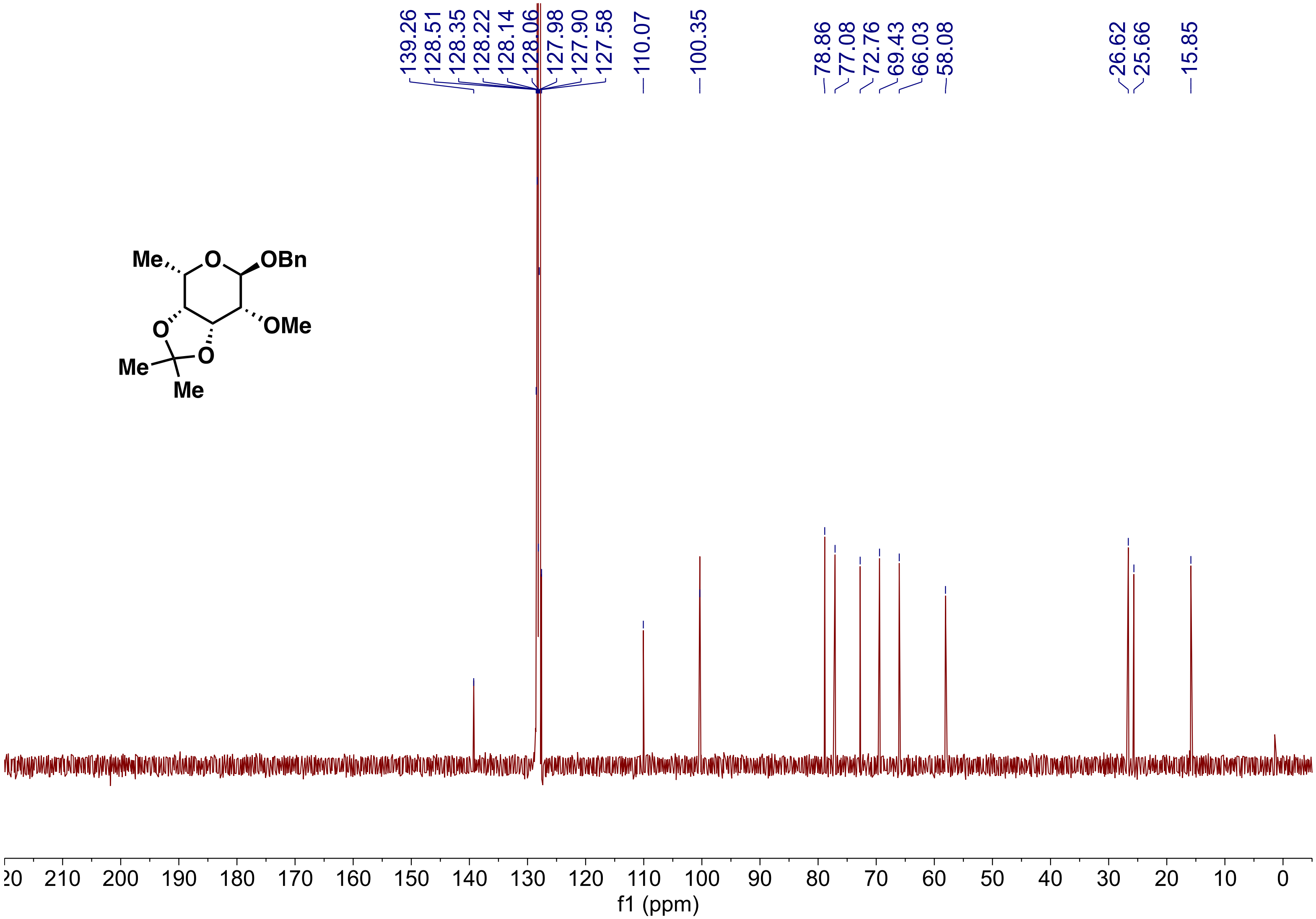

139.26
128.51
128.35
128.22
128.14
128.06
127.98
127.90
127.58
110.07
100.35
78.86
77.08
72.76
69.43
66.03
58.08
26.62
25.66
15.85
Me
O
OBn
OMe
O
O
Me
Me
210 200 190 180 170 160 150 140 130 120 110 100 90 80 70 60 50 40 30 20 10 0
f1 (ppm)

^{1}H NMR of **11**

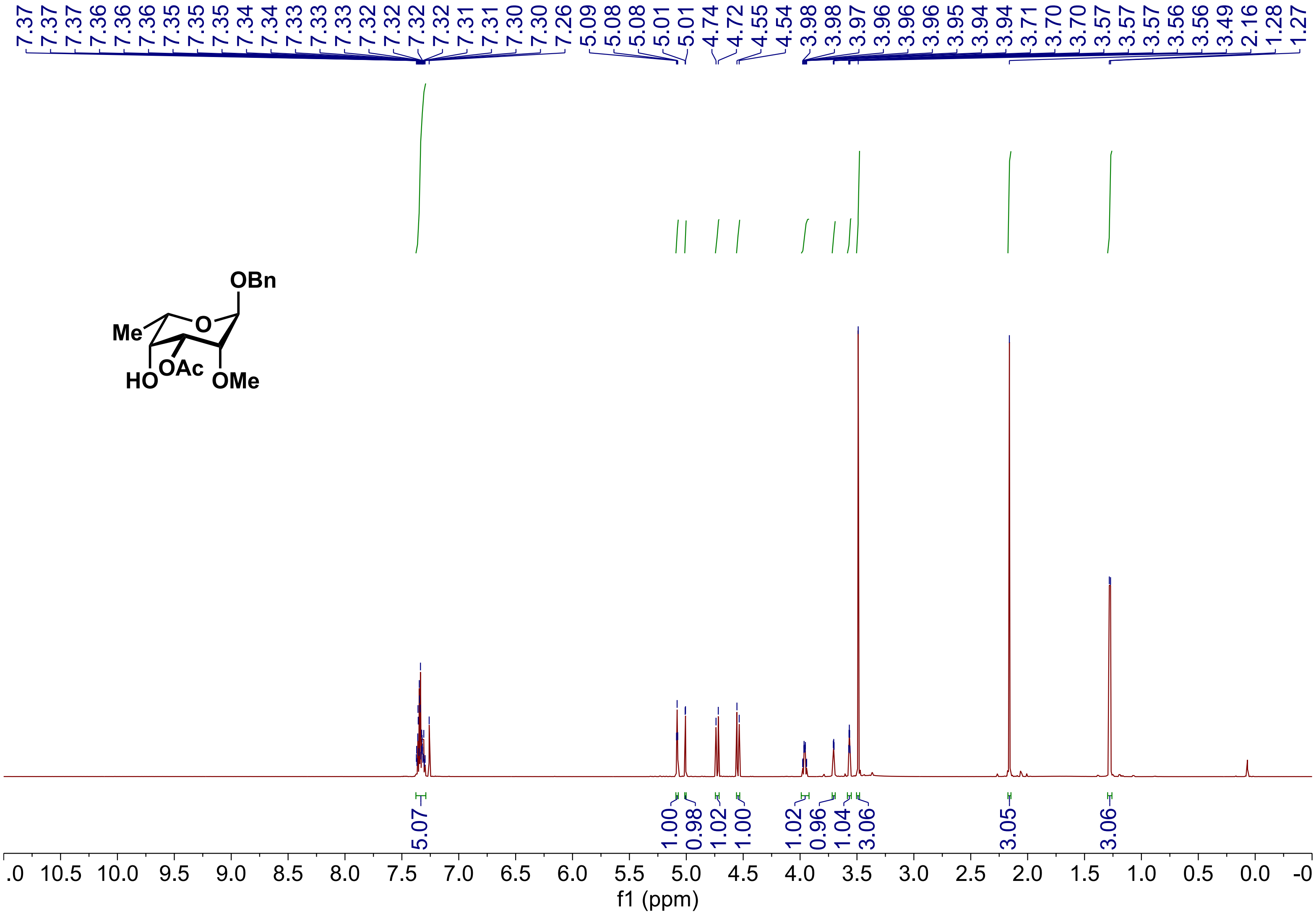

7.37 7.37 7.37 7.36 7.36 7.36 7.35 7.35 7.35 7.34 7.34 7.33 7.33 7.33 7.32 7.32 7.32 7.32 7.31 7.31 7.30 7.30 7.26 5.09 5.08 5.08 5.01 5.01 4.74 4.72 4.55 4.54 3.98 3.98 3.97 3.96 3.96 3.96 3.95 3.94 3.94 3.71 3.70 3.70 3.57 3.57 3.57 3.56 3.56 3.49 2.16 1.28 1.27
OBn
Me
O
HO
OAc
OMe
5.07 1.00 0.98 1.02 1.00 1.02 0.96 1.04 3.06 3.05 3.06
.0 10.5 10.0 9.5 9.0 8.5 8.0 7.5 7.0 6.5 6.0 5.5 5.0 4.5 4.0 3.5 3.0 2.5 2.0 1.5 1.0 0.5 0.0 -0
f1 (ppm)

^{13}C NMR of **11**

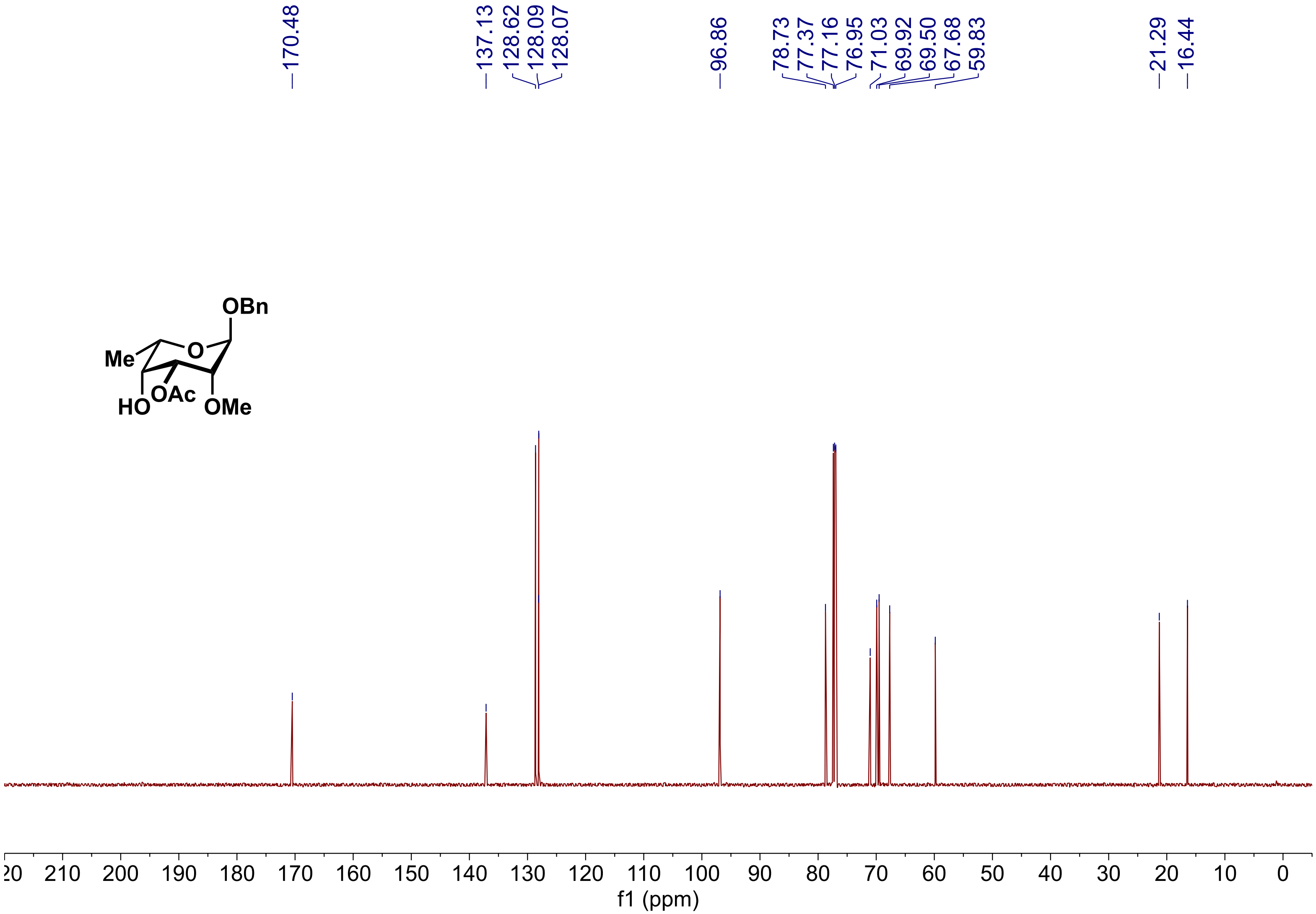

170.48
137.13
128.62
128.09
128.07
96.86
78.73
77.37
77.16
76.95
71.03
69.92
69.50
67.68
59.83
21.29
16.44
OBn
Me
O
HO
OAc
OMe
220 210 200 190 180 170 160 150 140 130 120 110 100 90 80 70 60 50 40 30 20 10 0
f1 (ppm)

^{1}H NMR of **13**

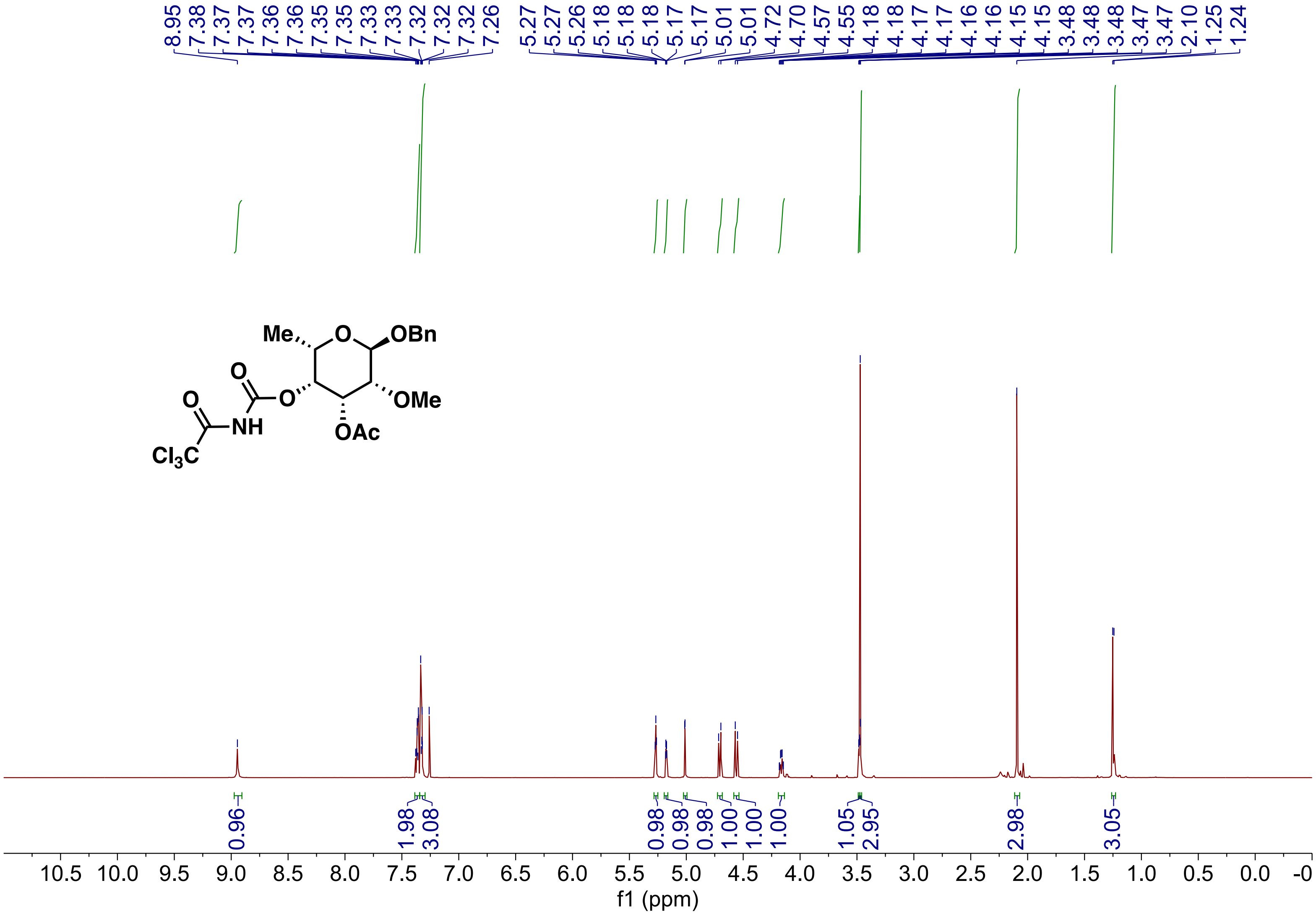
8.95
7.38
7.37
7.37
7.36
7.36
7.35
7.35
7.33
7.33
7.32
7.32
7.32
7.26
5.27
5.27
5.26
5.18
5.18
5.18
5.17
5.17
5.01
5.01
4.72
4.70
4.57
4.55
4.18
4.18
4.17
4.17
4.16
4.16
4.15
4.15
3.48
3.48
3.48
3.47
3.47
2.10
1.25
1.24
Me
O
OBn
O
O
NH
O
OMe
OAc
O
Cl3C
0.96
1.98
3.08
0.98
0.98
0.98
1.00
1.00
1.00
1.05
2.95
2.98
3.05
10.5
10.0
9.5
9.0
8.5
8.0
7.5
7.0
6.5
6.0
5.5
5.0
4.5
4.0
3.5
3.0
2.5
2.0
1.5
1.0
0.5
0.0
-0
f1 (ppm)

^{13}C NMR of **13**

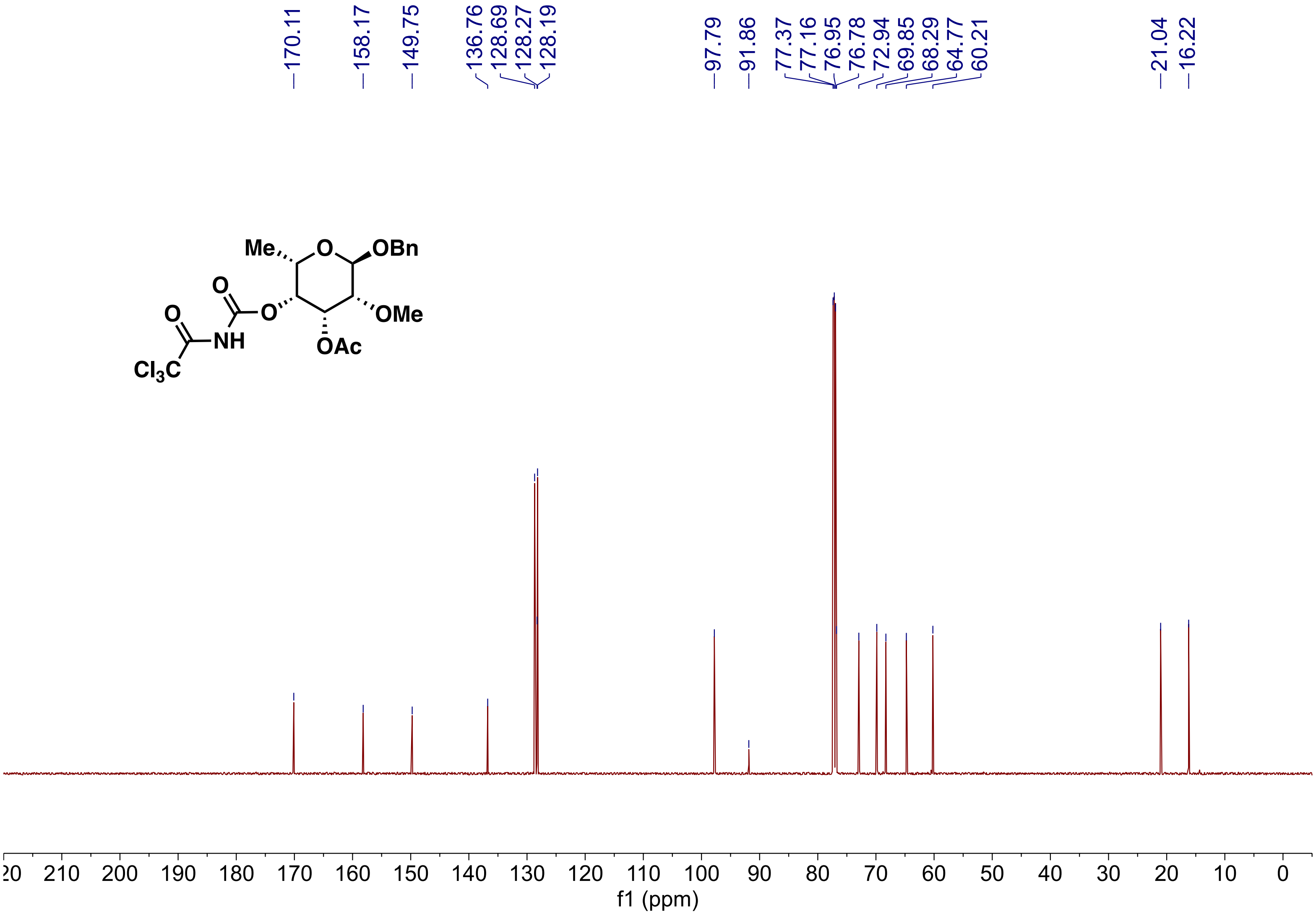

170.11
158.17
149.75
136.76
128.69
128.27
128.19
97.79
91.86
77.37
77.16
76.95
76.78
72.94
69.85
68.29
64.77
60.21
21.04
16.22
Me
O
OBn
O
O
O
NH
OMe
OAc
Cl3C
20
210
200
190
180
170
160
150
140
130
120
110
100
90
80
70
60
50
40
30
20
10
0
f1 (ppm)

^{1}H NMR of **14**

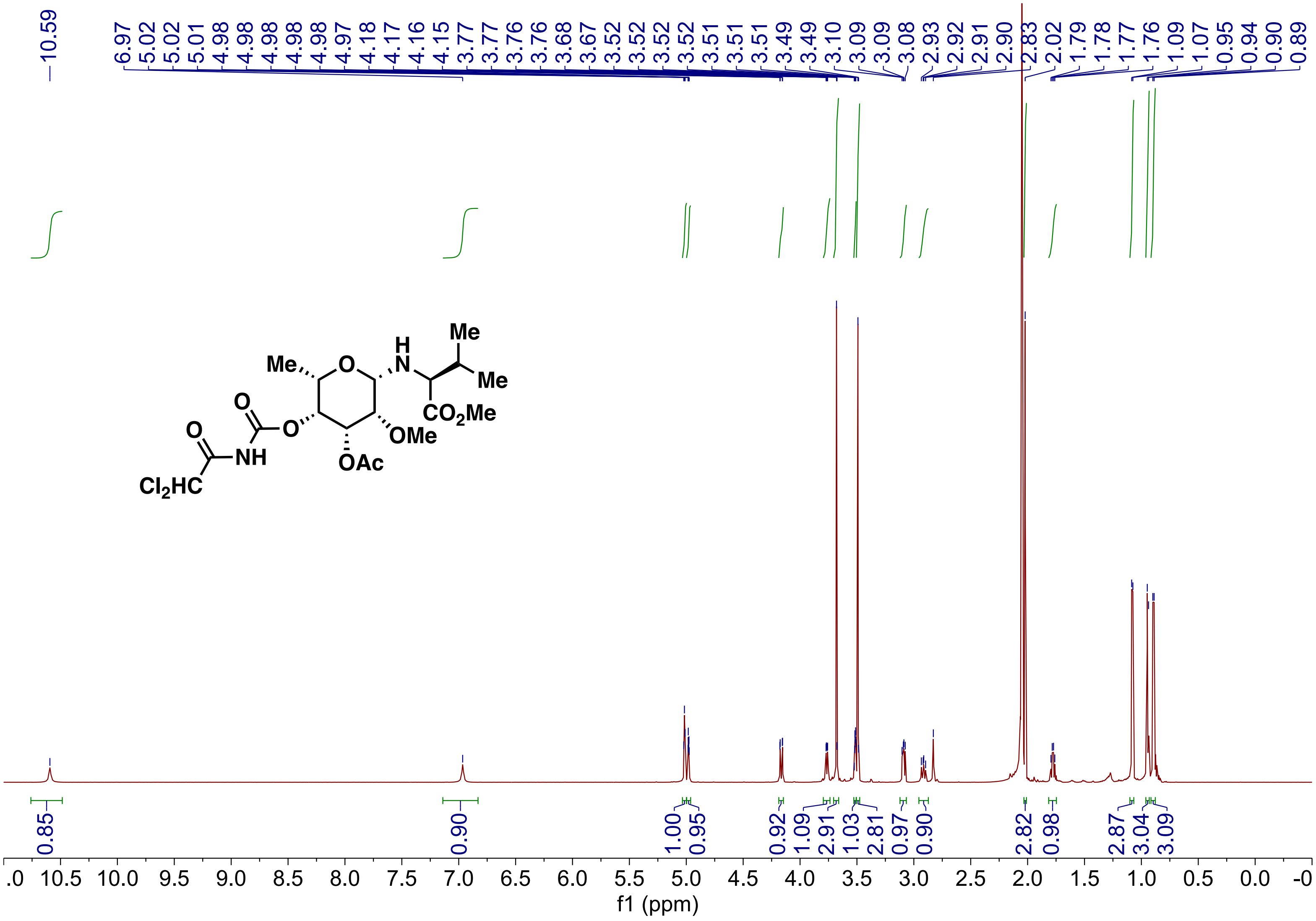

^{13}C NMR of **14**

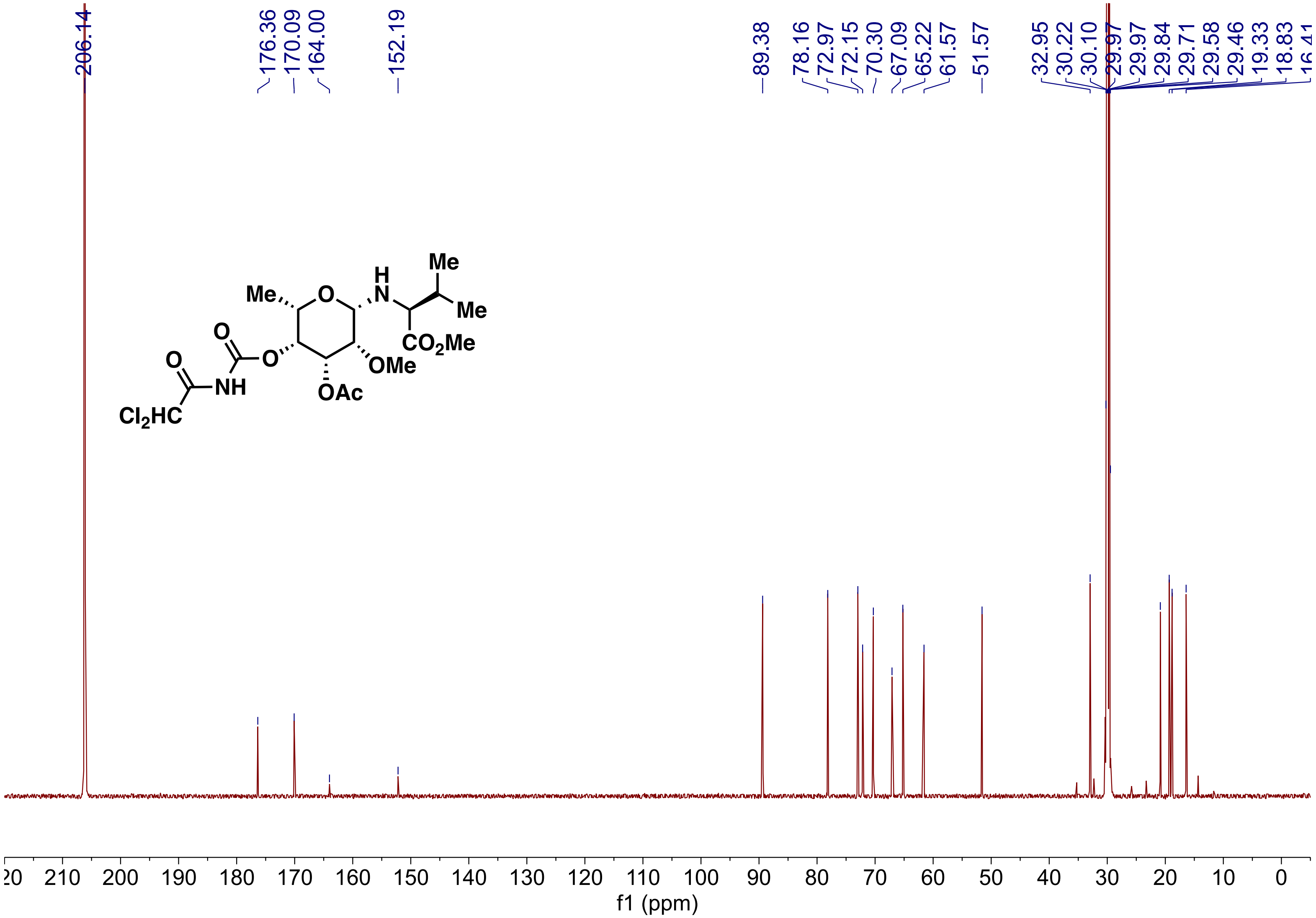

HSQC of **14**

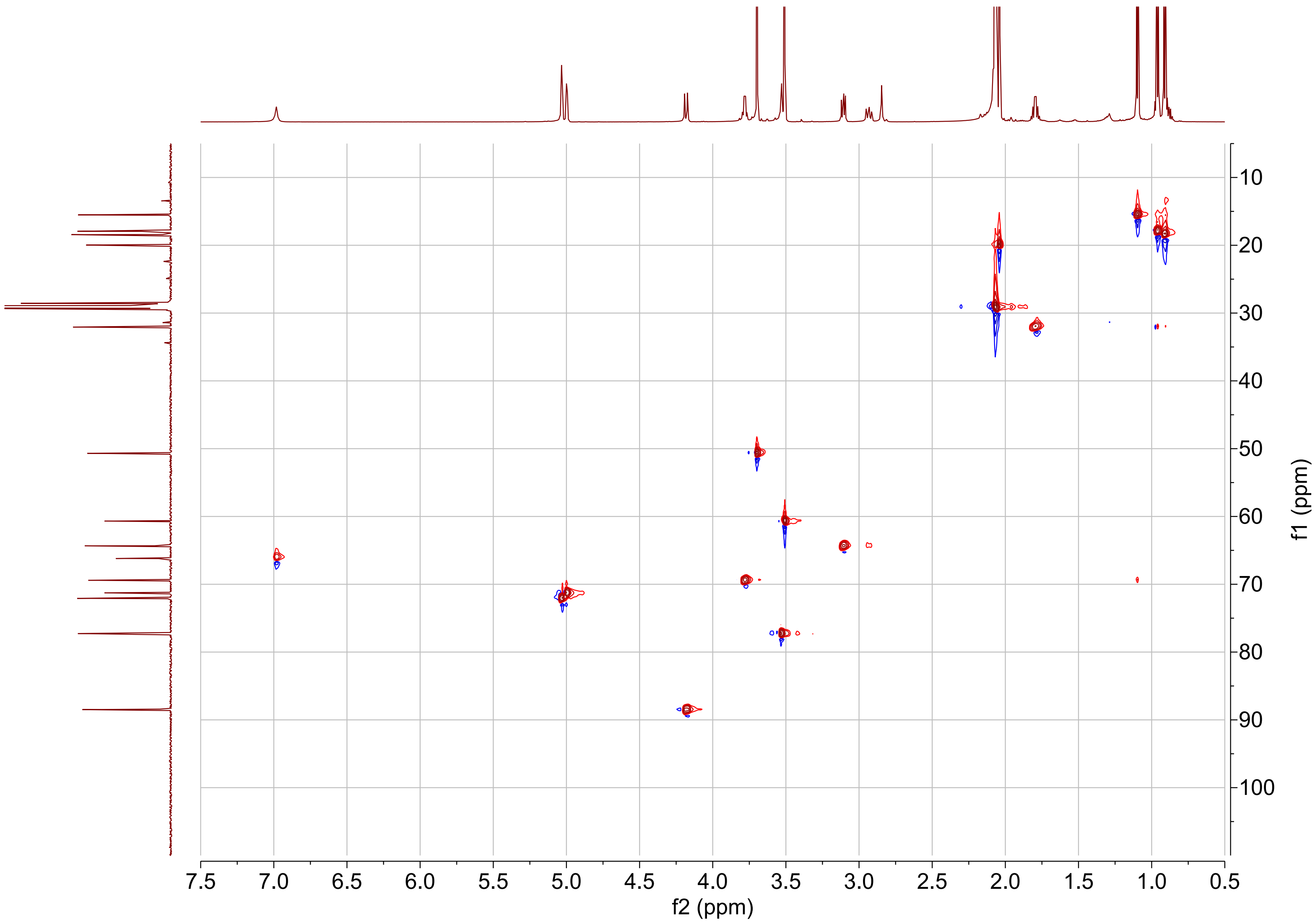

10
20
30
40
50
60
70
80
90
100
f1 (ppm)
7.5
7.0
6.5
6.0
5.5
5.0
4.5
4.0
3.5
3.0
2.5
2.0
1.5
1.0
0.5
f2 (ppm)

COSY of **14**

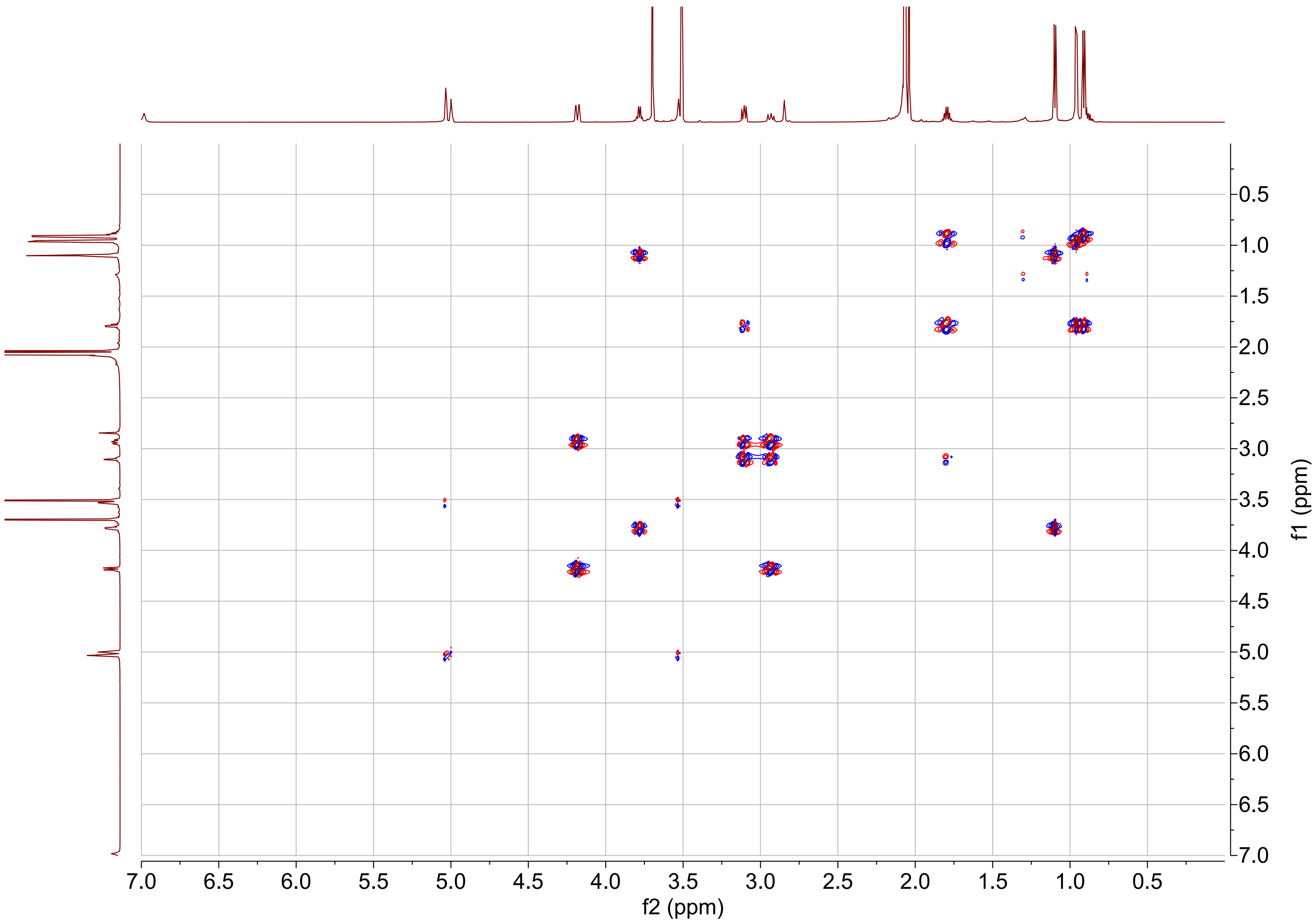

NOESY of **14**

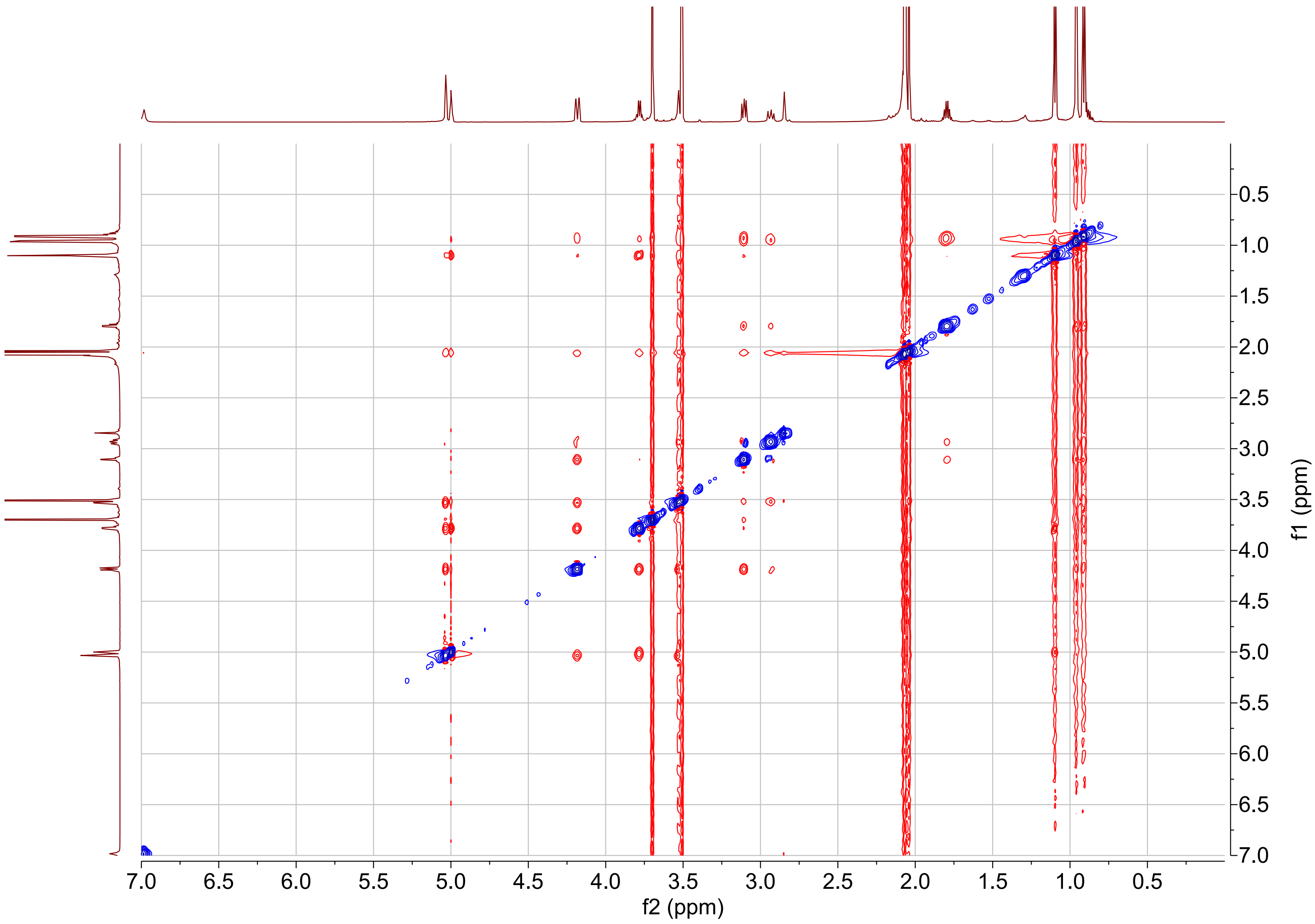

^{1}H NMR of **S1**

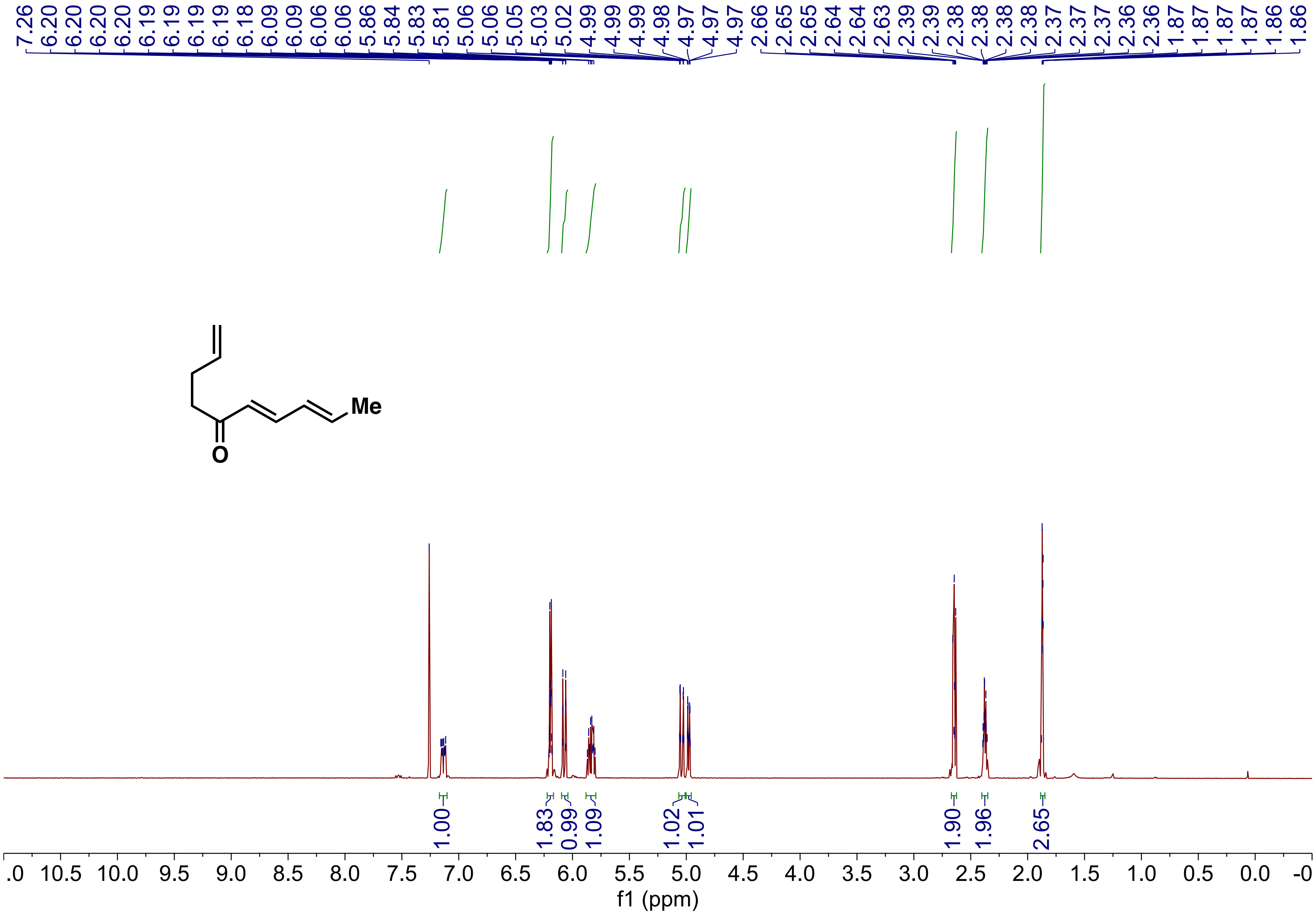

7.26 6.20 6.20 6.20 6.20 6.19 6.19 6.19 6.19 6.18 6.09 6.09 6.06 6.06 5.86 5.84 5.83 5.81 5.06 5.06 5.05 5.03 5.02 4.99 4.99 4.99 4.98 4.97 4.97 4.97 2.66 2.65 2.65 2.64 2.64 2.63 2.39 2.39 2.38 2.38 2.38 2.38 2.37 2.37 2.37 2.36 2.36 1.87 1.87 1.87 1.87 1.86 1.86
Me
O
1.00
1.83
0.99
1.09
1.02
1.01
1.90
1.96
2.65
.0 10.5 10.0 9.5 9.0 8.5 8.0 7.5 7.0 6.5 6.0 5.5 5.0 4.5 4.0 3.5 3.0 2.5 2.0 1.5 1.0 0.5 0.0 -0
f1 (ppm)

^{13}C NMR of **S1**

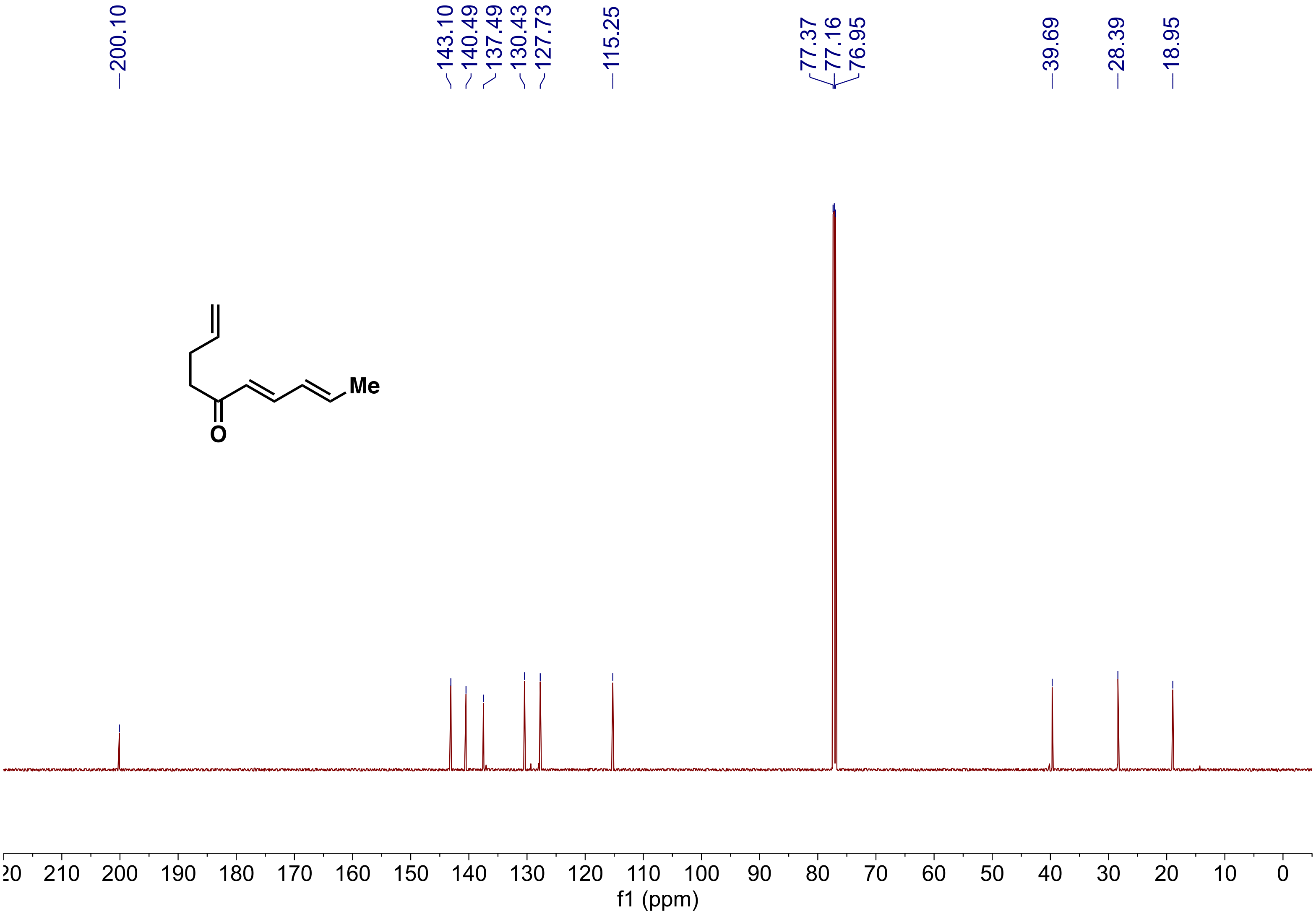
200.10
143.10
140.49
137.49
130.43
127.73
115.25
77.37
77.16
76.95
39.69
28.39
18.95
Me
O
f1 (ppm)

^{1}H NMR of **S2**

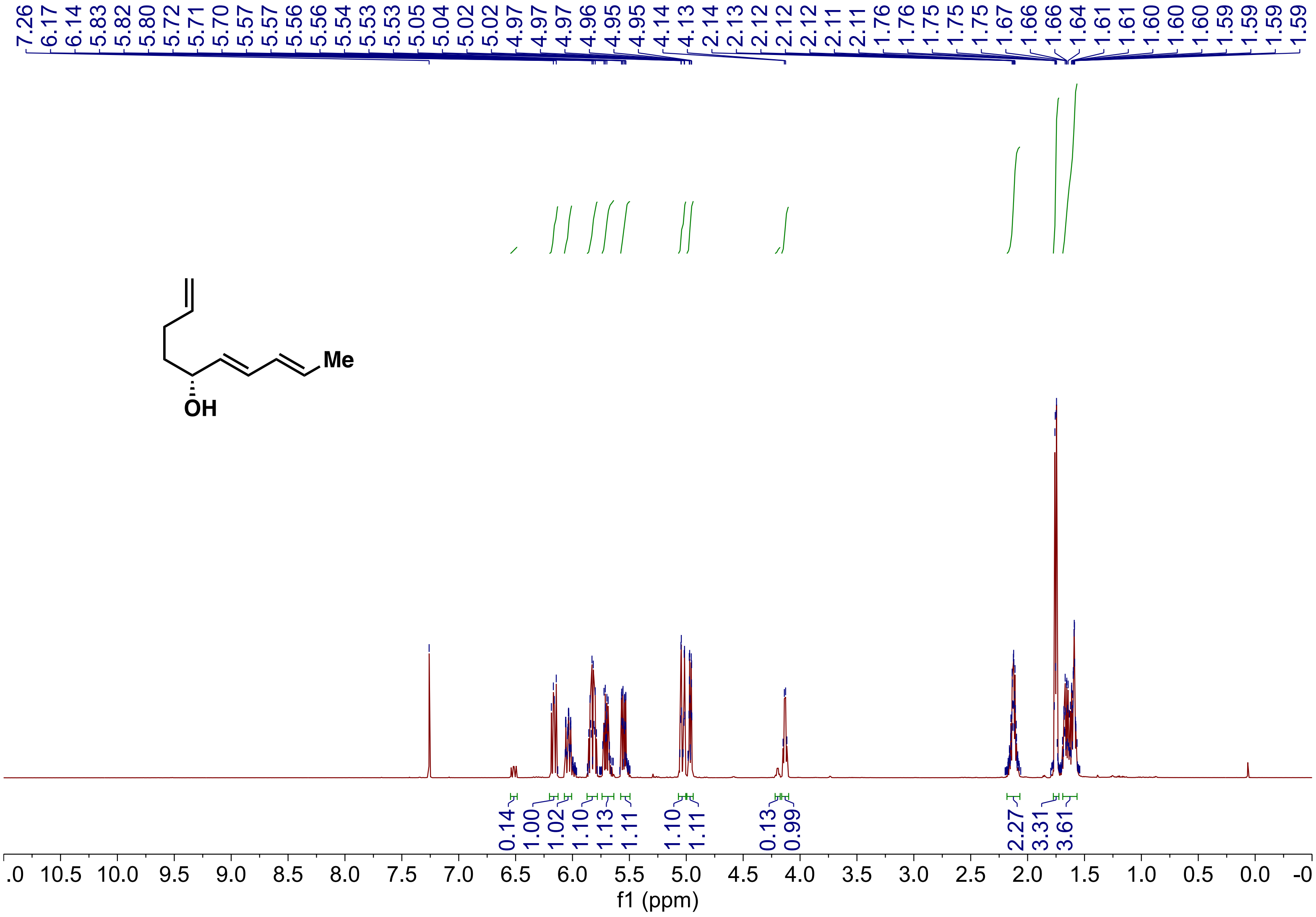

7.26 6.17 6.14 5.83 5.82 5.80 5.72 5.71 5.70 5.57 5.57 5.56 5.56 5.54 5.53 5.53 5.05 5.04 5.02 5.02 4.97 4.97 4.96 4.95 4.95 4.14 4.13 2.14 2.13 2.12 2.12 2.12 2.11 2.11 1.76 1.76 1.75 1.75 1.75 1.67 1.66 1.66 1.64 1.61 1.61 1.60 1.60 1.60 1.59 1.59 1.59 1.59
Me
OH
0.14 1.00 1.02 1.10 1.13 1.11 1.10 1.11 0.13 0.99 2.27 3.31 3.61
.0 10.5 10.0 9.5 9.0 8.5 8.0 7.5 7.0 6.5 6.0 5.5 5.0 4.5 4.0 3.5 3.0 2.5 2.0 1.5 1.0 0.5 0.0 -0
f1 (ppm)

^{13}C NMR of **S2**

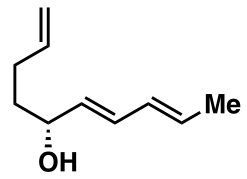

OH
Me

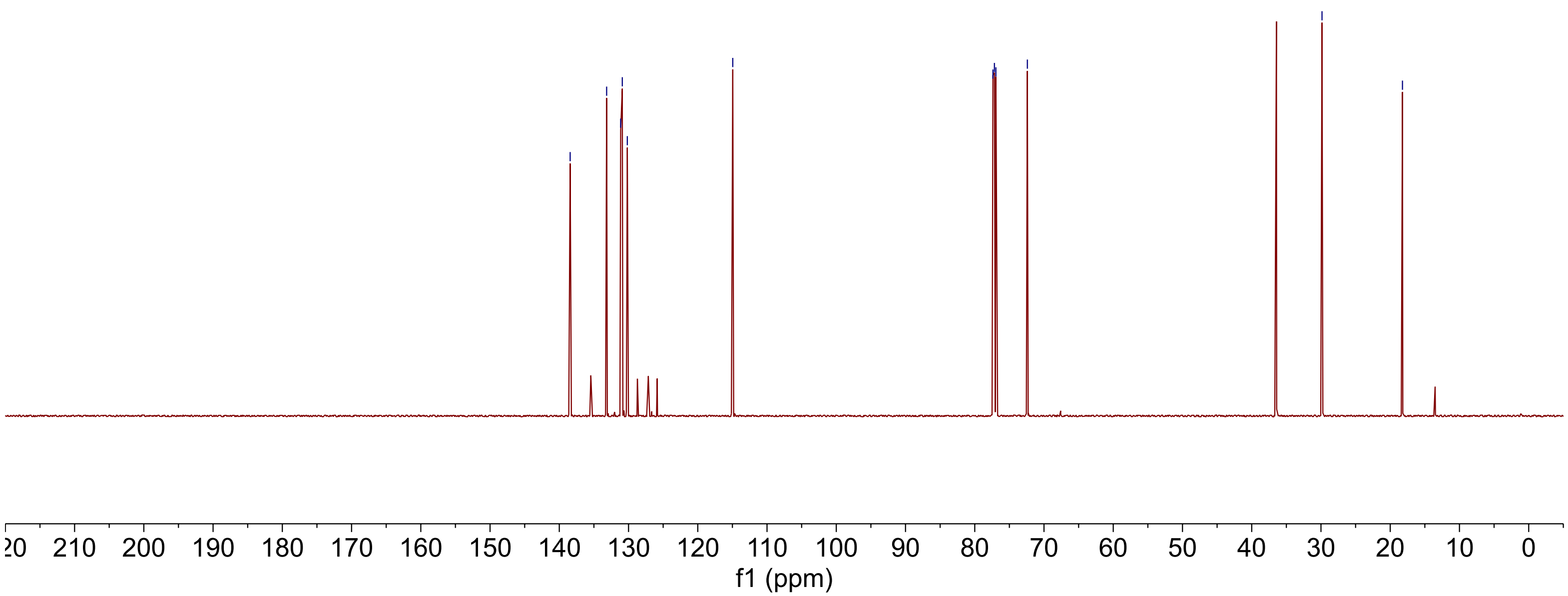

138.43
133.16
131.14
130.90
130.18
114.95
77.37
77.16
76.95
72.40
29.85
18.23
20
210
200
190
180
170
160
150
140
130
120
110
100
90
80
70
60
50
40
30
20
10
0
f1 (ppm)

^{1}H NMR of **17**

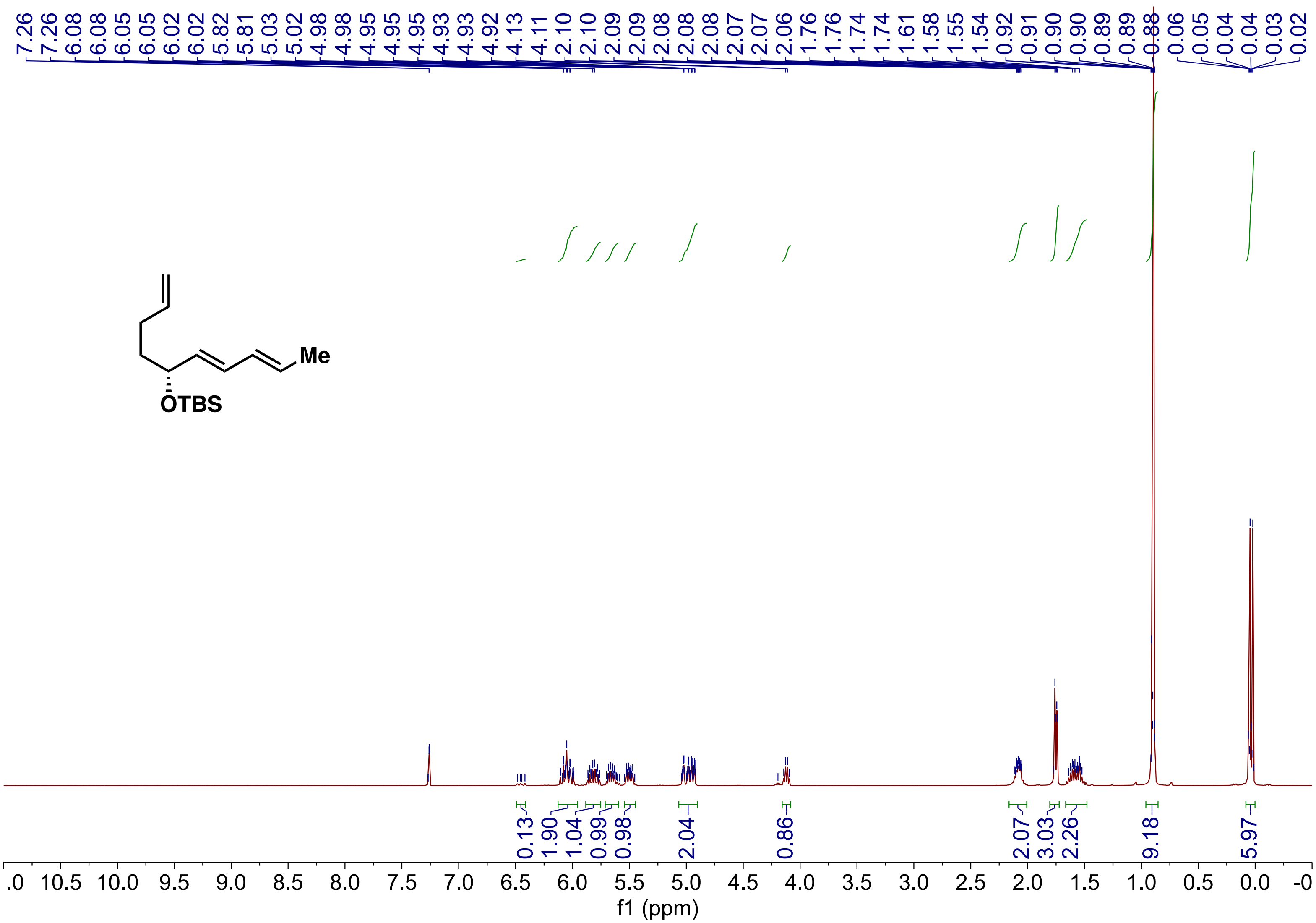

7.26 7.26 6.08 6.08 6.05 6.05 6.02 6.02 5.82 5.81 5.03 5.02 4.98 4.98 4.95 4.95 4.95 4.93 4.93 4.92 4.13 4.11 2.10 2.10 2.09 2.09 2.08 2.08 2.08 2.07 2.07 2.06 1.76 1.76 1.74 1.74 1.61 1.58 1.55 1.54 0.92 0.91 0.90 0.90 0.89 0.89 0.88 0.06 0.05 0.04 0.04 0.03 0.02
Me
OTBS
0.13 1.90 1.04 0.99 0.98 2.04 0.86 2.07 3.03 2.26 9.18 5.97
.0 10.5 10.0 9.5 9.0 8.5 8.0 7.5 7.0 6.5 6.0 5.5 5.0 4.5 4.0 3.5 3.0 2.5 2.0 1.5 1.0 0.5 0.0 -0
f1 (ppm)

^{13}C NMR of **17**

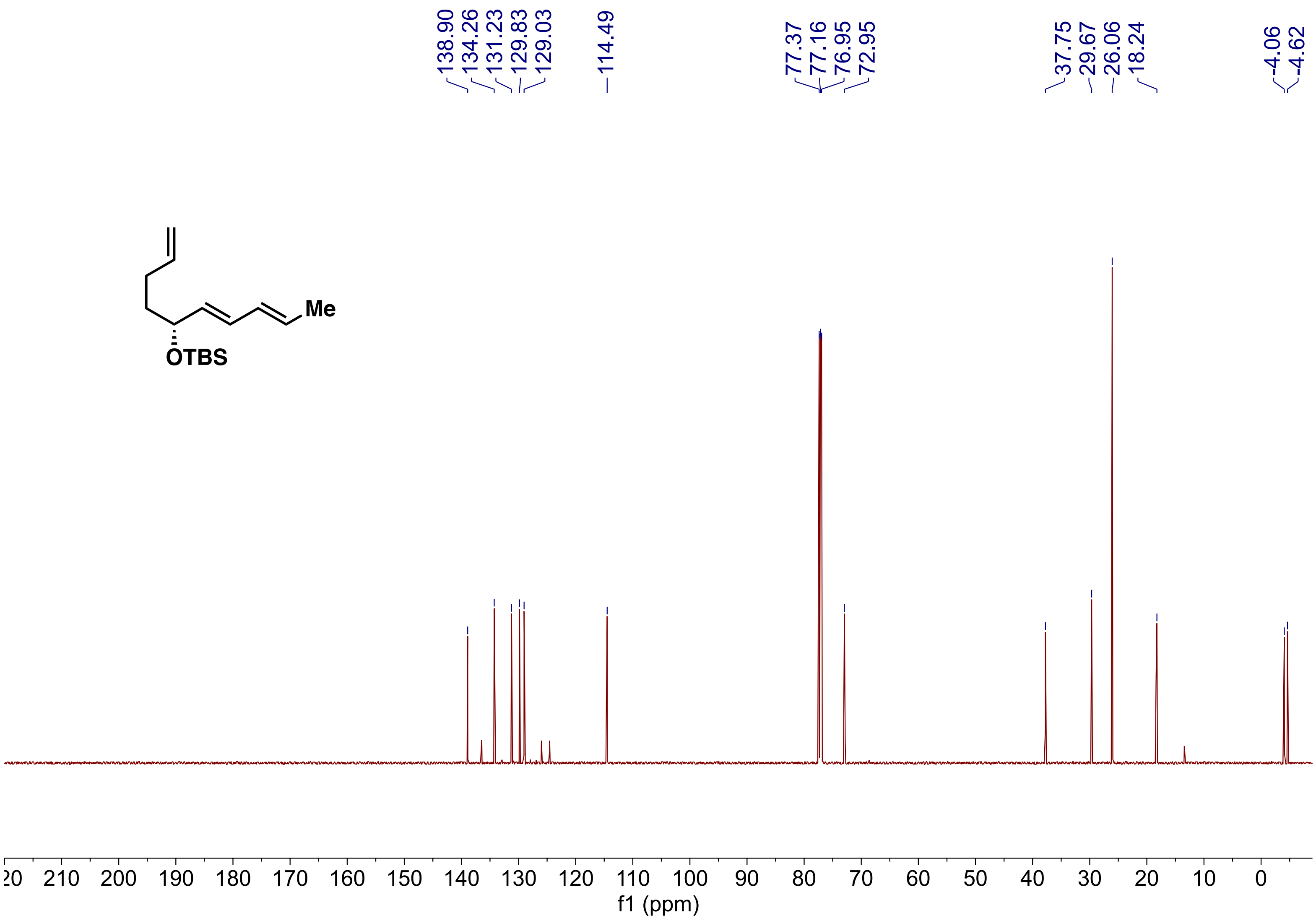
138.90
134.26
131.23
129.83
129.03
114.49
77.37
77.16
76.95
72.95
37.75
29.67
26.06
18.24
-4.06
-4.62
Me
OTBS
f1 (ppm)

^{1}H NMR of **S3**

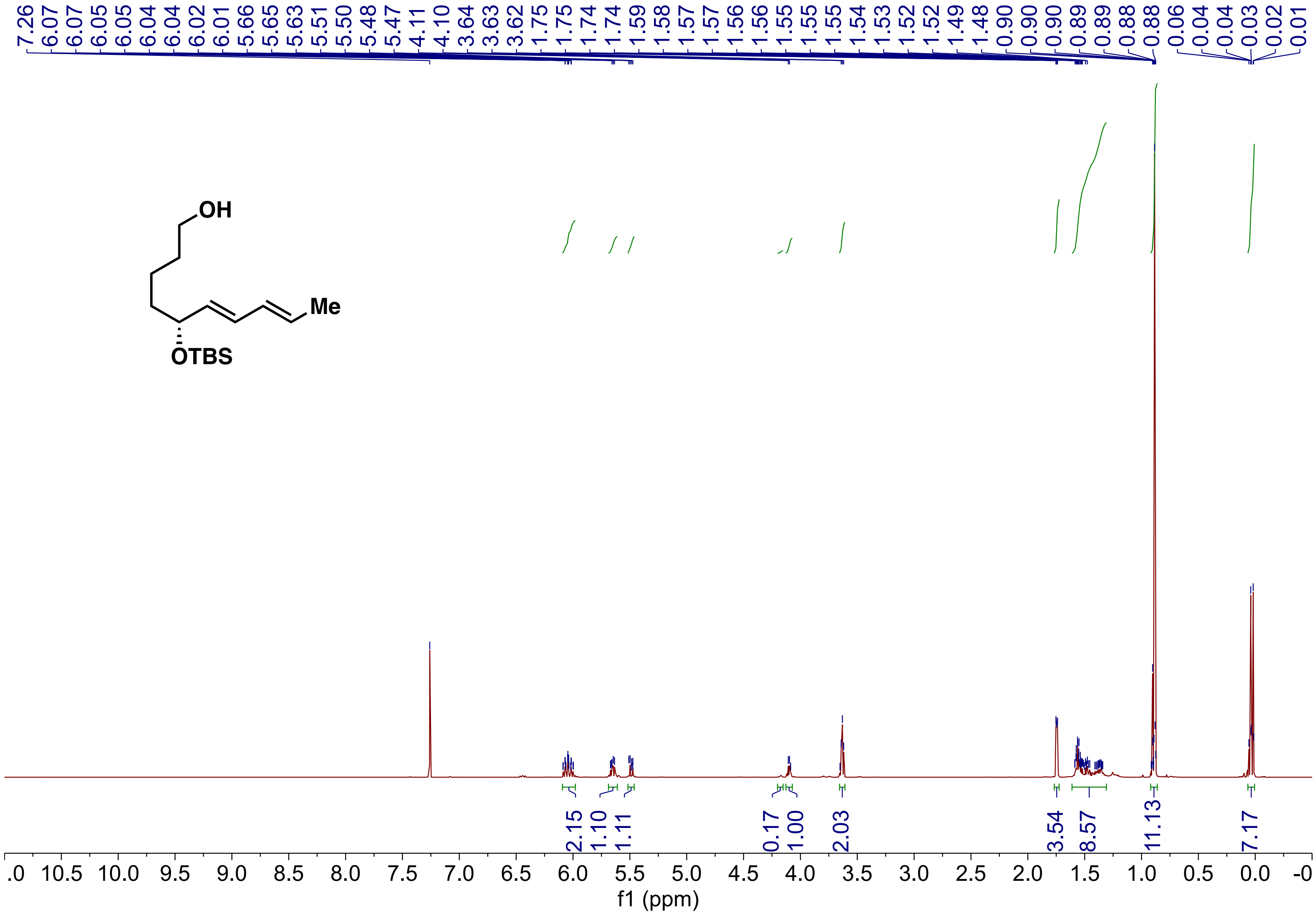

OH
Me
OTBS
7.26
6.07
6.07
6.05
6.05
6.04
6.04
6.02
6.01
5.66
5.65
5.63
5.51
5.50
5.48
5.47
4.11
4.10
3.64
3.63
3.62
1.75
1.75
1.74
1.74
1.59
1.58
1.57
1.57
1.56
1.56
1.55
1.55
1.55
1.54
1.53
1.52
1.52
1.49
1.48
0.90
0.90
0.90
0.89
0.89
0.88
0.88
0.06
0.04
0.04
0.03
0.02
0.01
2.15
1.10
1.11
0.17
1.00
2.03
3.54
8.57
11.13
7.17
f1 (ppm)

^{13}C NMR of **S3**

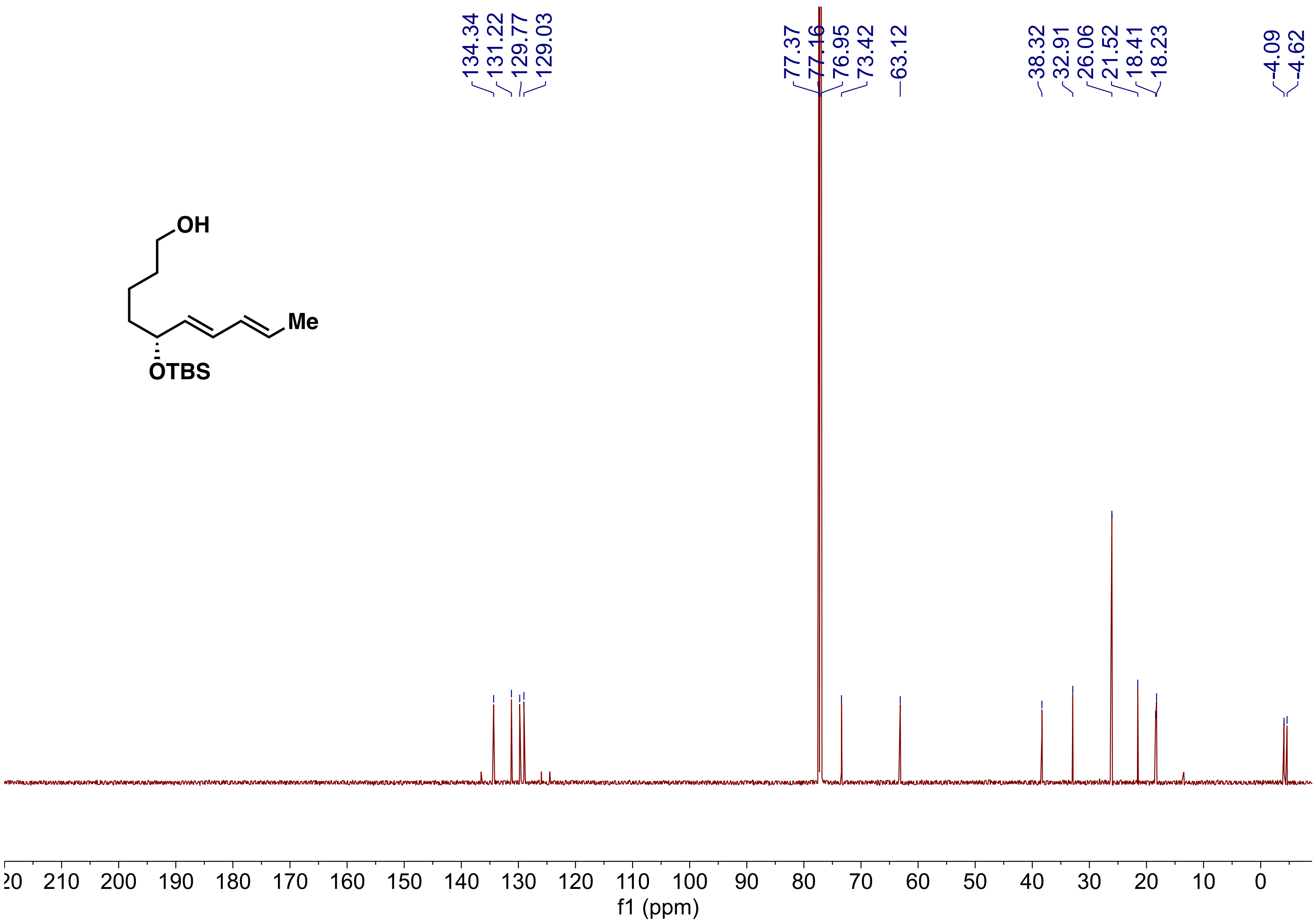

134.34
131.22
129.77
129.03
77.37
77.16
76.95
73.42
63.12
38.32
32.91
26.06
21.52
18.41
18.23
-4.09
-4.62
OH
Me
OTBS
20 210 200 190 180 170 160 150 140 130 120 110 100 90 80 70 60 50 40 30 20 10 0
f1 (ppm)

^{1}H NMR of **18**

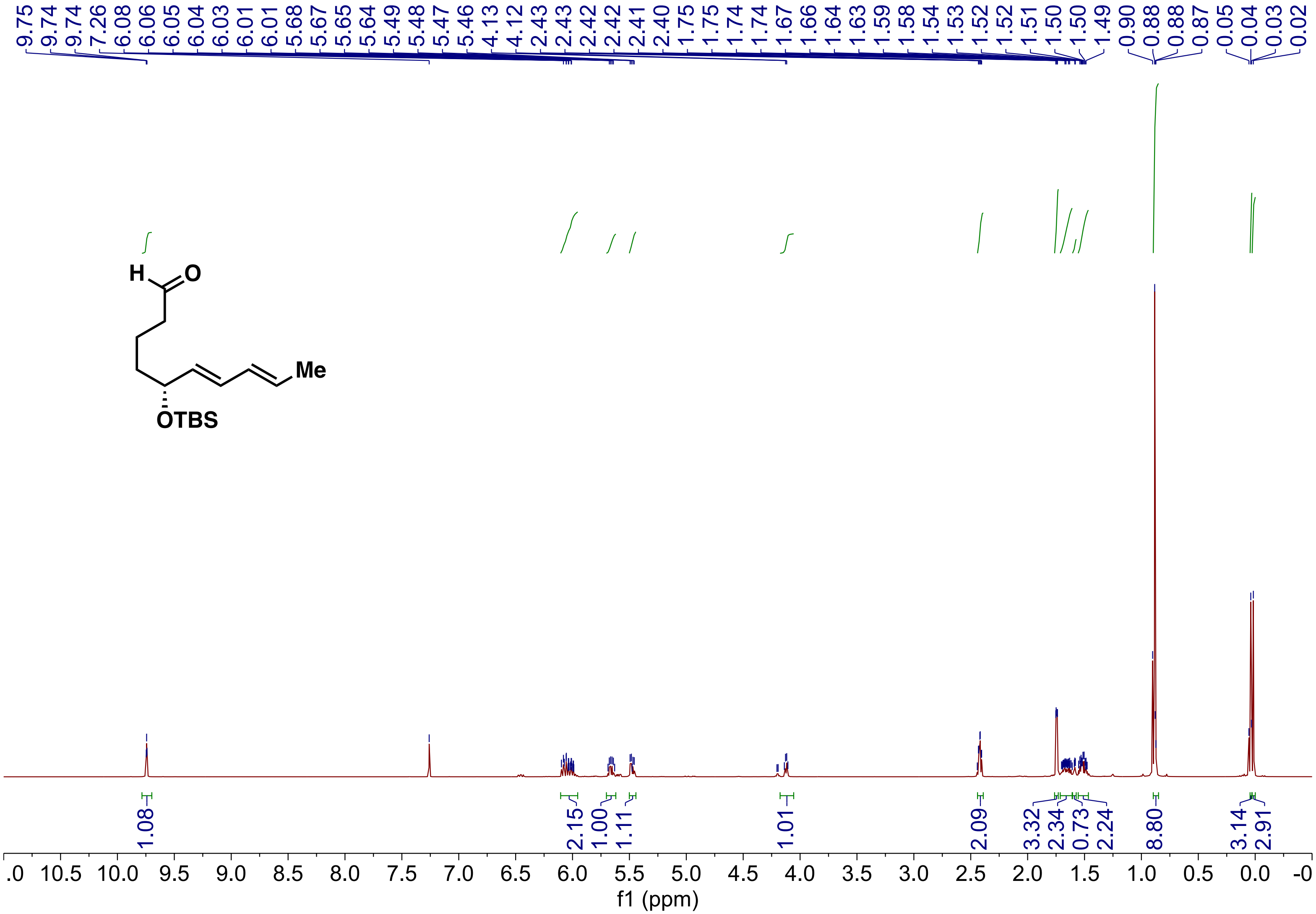
9.75 9.74 9.74 7.26 6.08 6.06 6.05 6.04 6.03 6.01 6.01 5.68 5.67 5.65 5.64 5.49 5.48 5.47 5.46 4.13 4.12 2.43 2.43 2.42 2.42 2.41 2.40 1.75 1.75 1.74 1.74 1.67 1.66 1.64 1.63 1.59 1.58 1.54 1.53 1.52 1.52 1.51 1.50 1.50 1.49 0.90 0.88 0.88 0.87 0.05 0.04 0.03 0.02
H
O
Me
OTBS
1.08
2.15
1.00
1.11
1.01
2.09
3.32
2.34
0.73
2.24
8.80
3.14
2.91
.0 10.5 10.0 9.5 9.0 8.5 8.0 7.5 7.0 6.5 6.0 5.5 5.0 4.5 4.0 3.5 3.0 2.5 2.0 1.5 1.0 0.5 0.0 -0
f1 (ppm)

^{13}C NMR of **18**

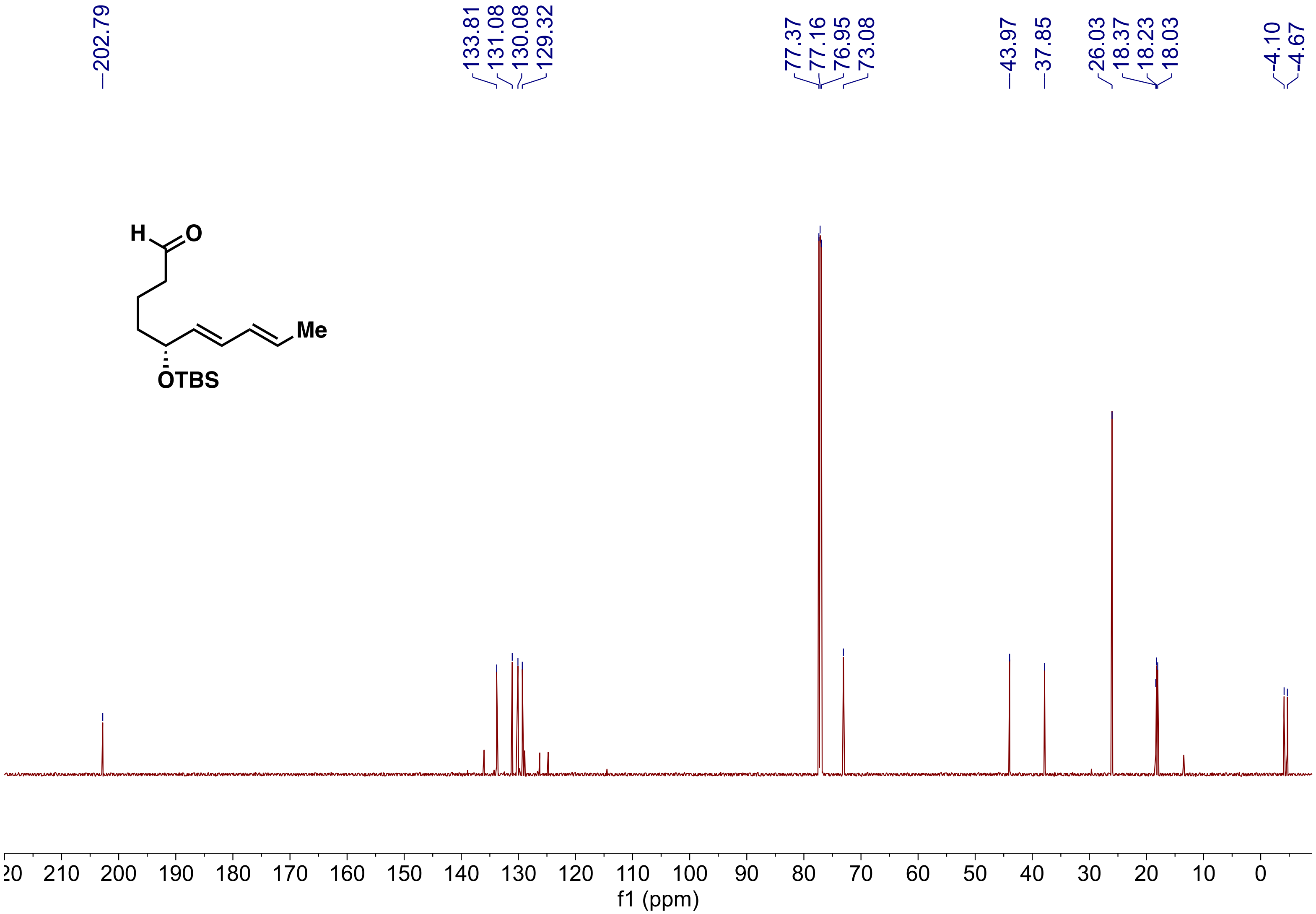

^{1}H NMR of **19**

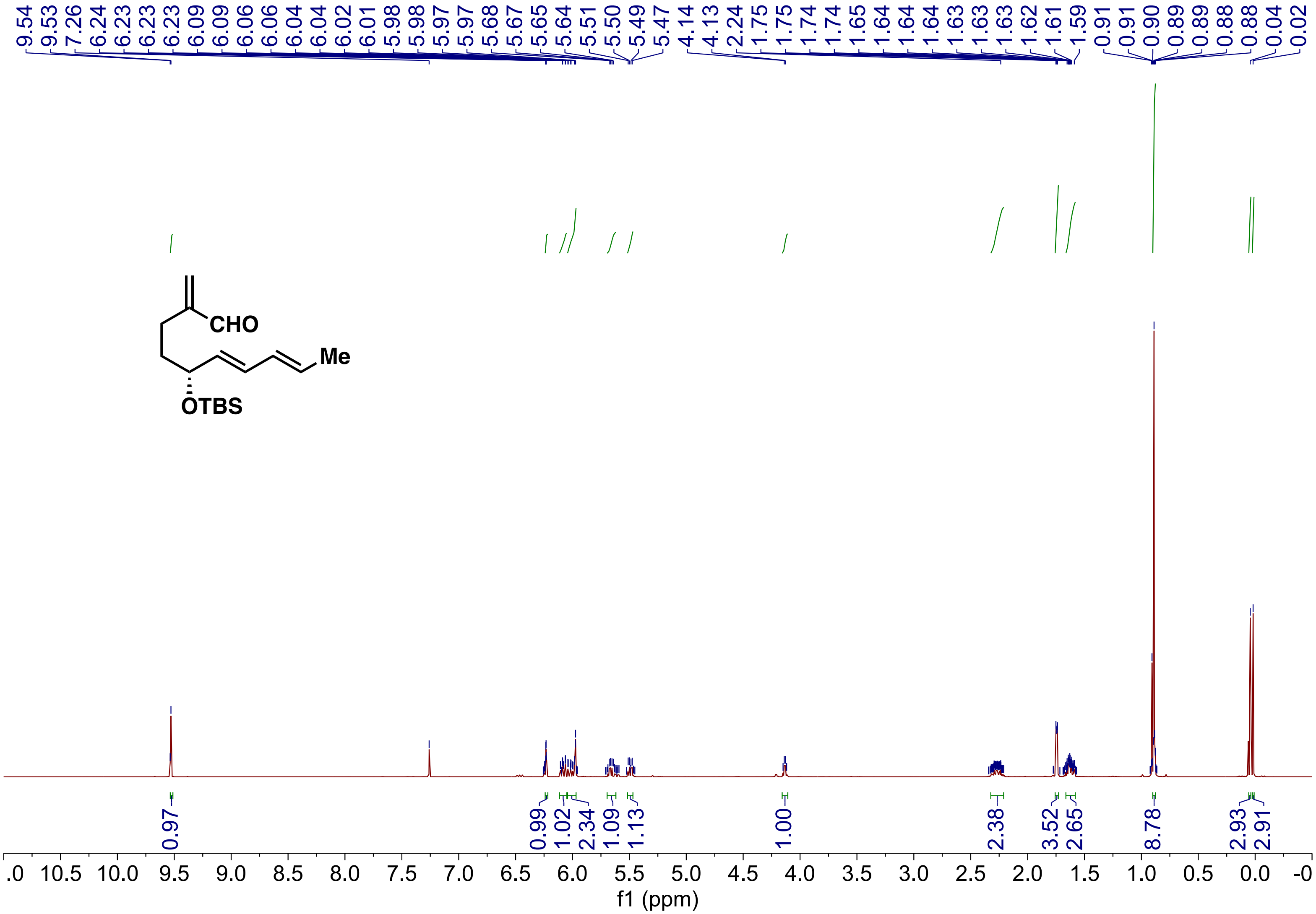
9.54
9.53
7.26
6.24
6.23
6.23
6.23
6.09
6.09
6.06
6.06
6.04
6.04
6.02
6.01
5.98
5.98
5.97
5.97
5.68
5.67
5.65
5.64
5.51
5.50
5.49
5.47
4.14
4.13
2.24
1.75
1.75
1.74
1.74
1.65
1.64
1.64
1.64
1.63
1.63
1.63
1.62
1.61
1.59
0.91
0.91
0.90
0.89
0.89
0.88
0.88
0.04
0.02
CHO
Me
OTBS
0.97
0.99
1.02
2.34
1.09
1.13
1.00
2.38
3.52
2.65
8.78
2.93
2.91
f1 (ppm)

^{13}C NMR of **19**

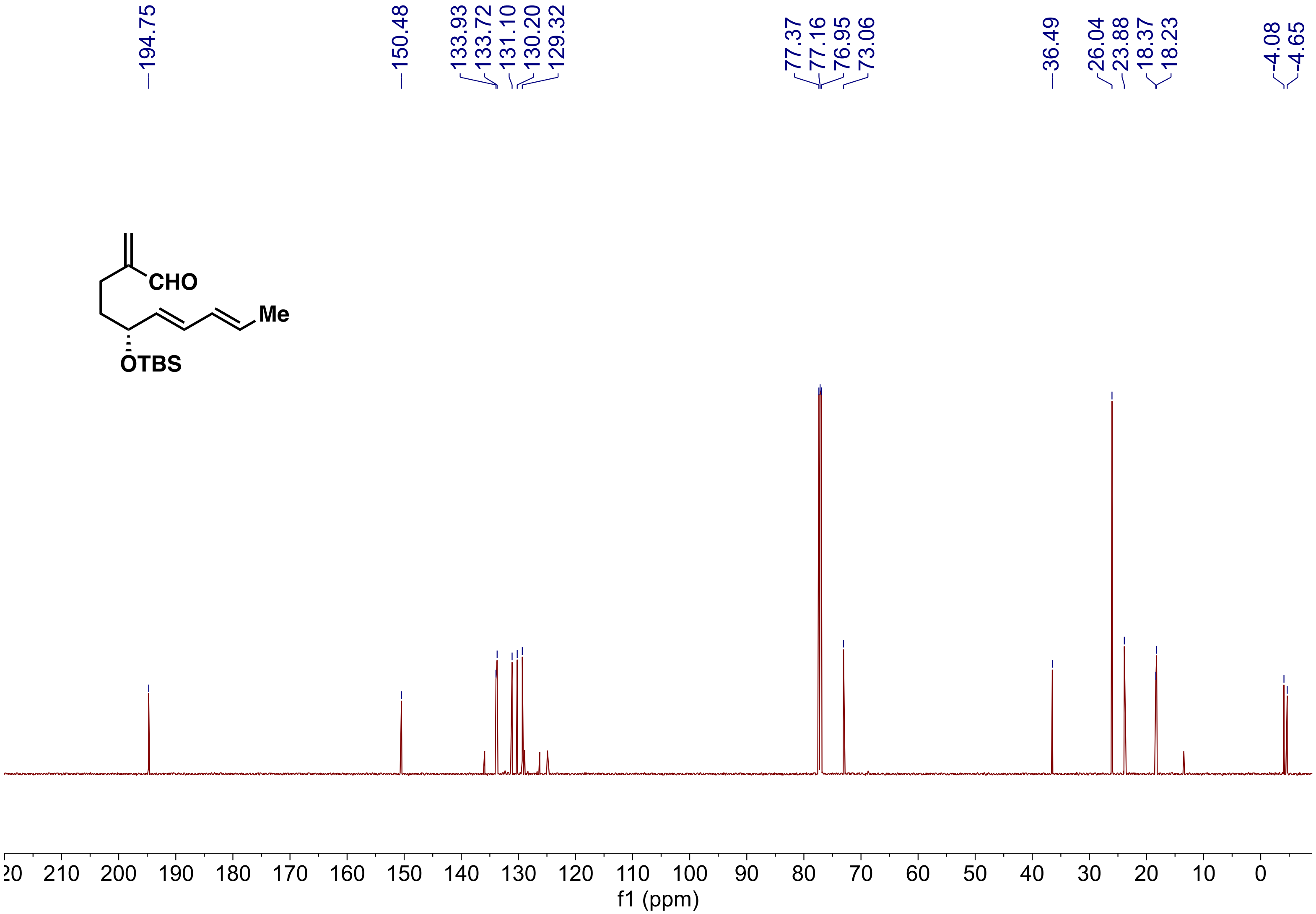

194.75
150.48
133.93
133.72
131.10
130.20
129.32
77.37
77.16
76.95
73.06
36.49
26.04
23.88
18.37
18.23
-4.08
-4.65
CHO
Me
OTBS
20 210 200 190 180 170 160 150 140 130 120 110 100 90 80 70 60 50 40 30 20 10 0
f1 (ppm)

^{1}H NMR of **21**

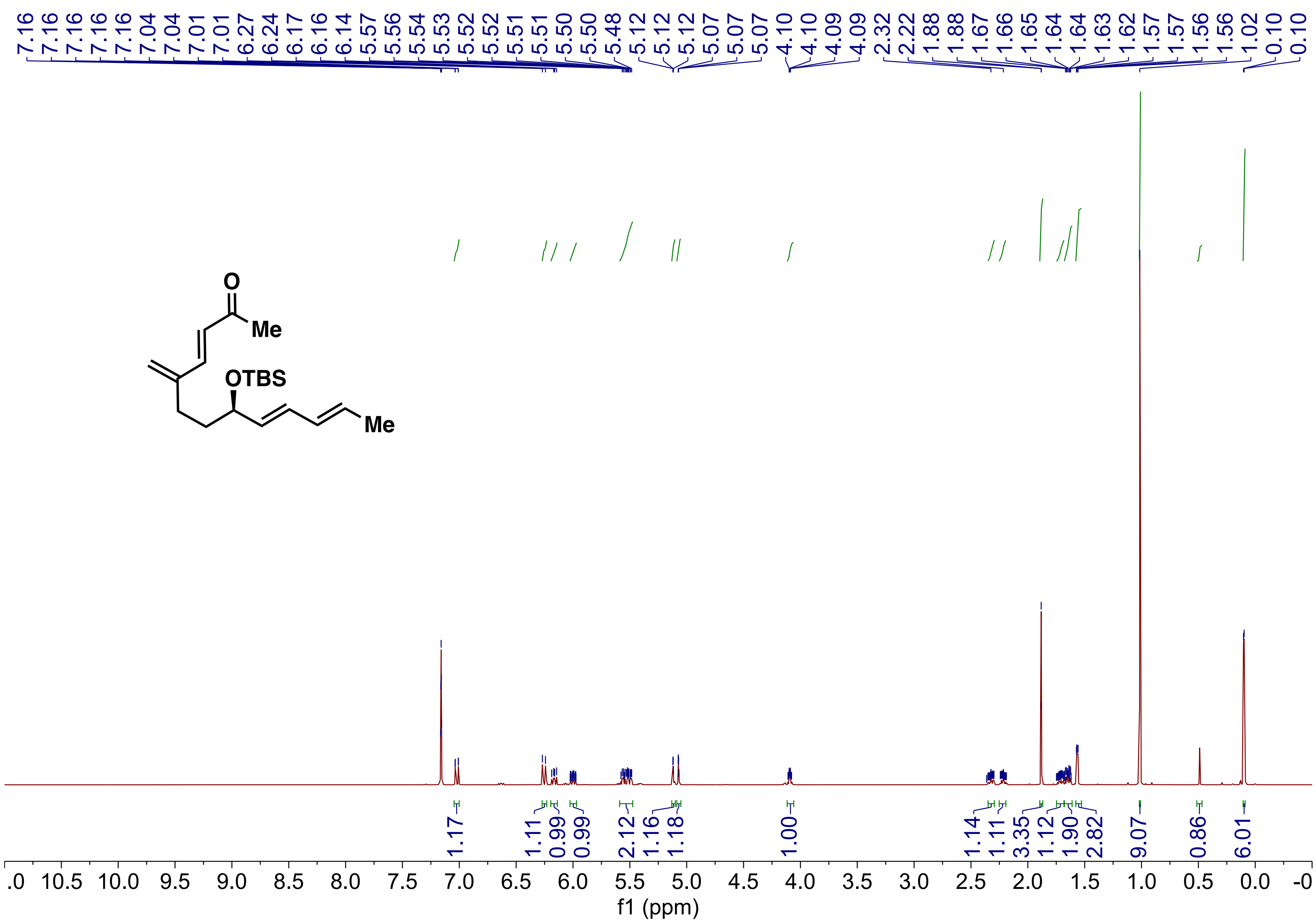

O
Me
OTBS
Me
7.16
7.16
7.16
7.16
7.16
7.04
7.04
7.01
7.01
6.27
6.24
6.17
6.16
6.14
5.57
5.56
5.54
5.53
5.52
5.52
5.51
5.51
5.50
5.50
5.48
5.12
5.12
5.12
5.07
5.07
5.07
4.10
4.10
4.09
4.09
2.32
2.22
1.88
1.88
1.67
1.66
1.65
1.64
1.64
1.63
1.62
1.57
1.57
1.56
1.56
1.02
0.10
0.10
1.17
1.11
0.99
0.99
2.12
1.16
1.18
1.00
1.14
1.11
3.35
1.12
1.90
2.82
9.07
0.86
6.01
f1 (ppm)

^{13}C NMR of **21**

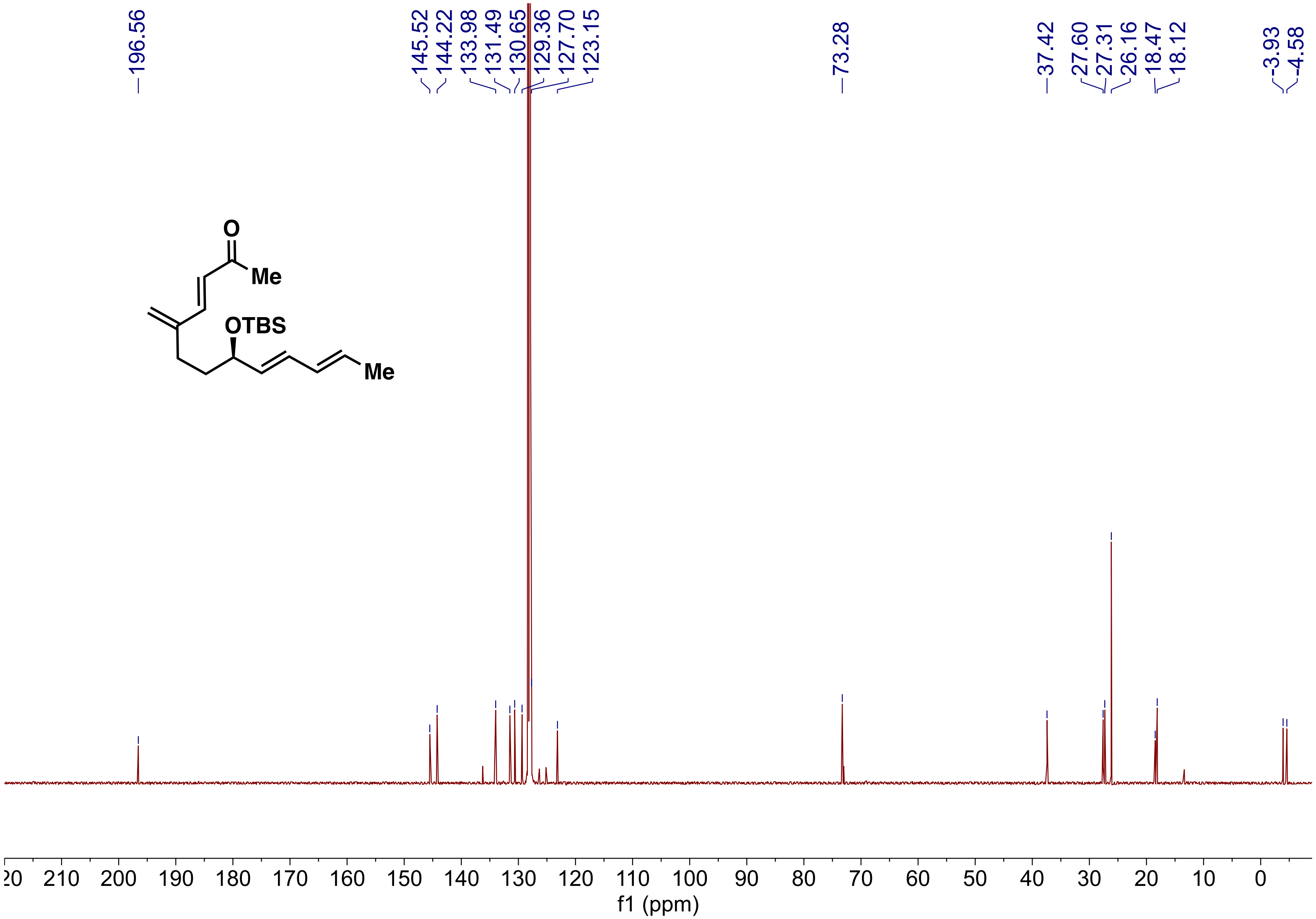
196.56
145.52
144.22
133.98
131.49
130.65
129.36
127.70
123.15
73.28
37.42
27.60
27.31
26.16
18.47
18.12
-3.93
-4.58
O
Me
OTBS
Me
20
210
200
190
180
170
160
150
140
130
120
110
100
90
80
70
60
50
40
30
20
10
0
f1 (ppm)

^{1}H NMR of **22**

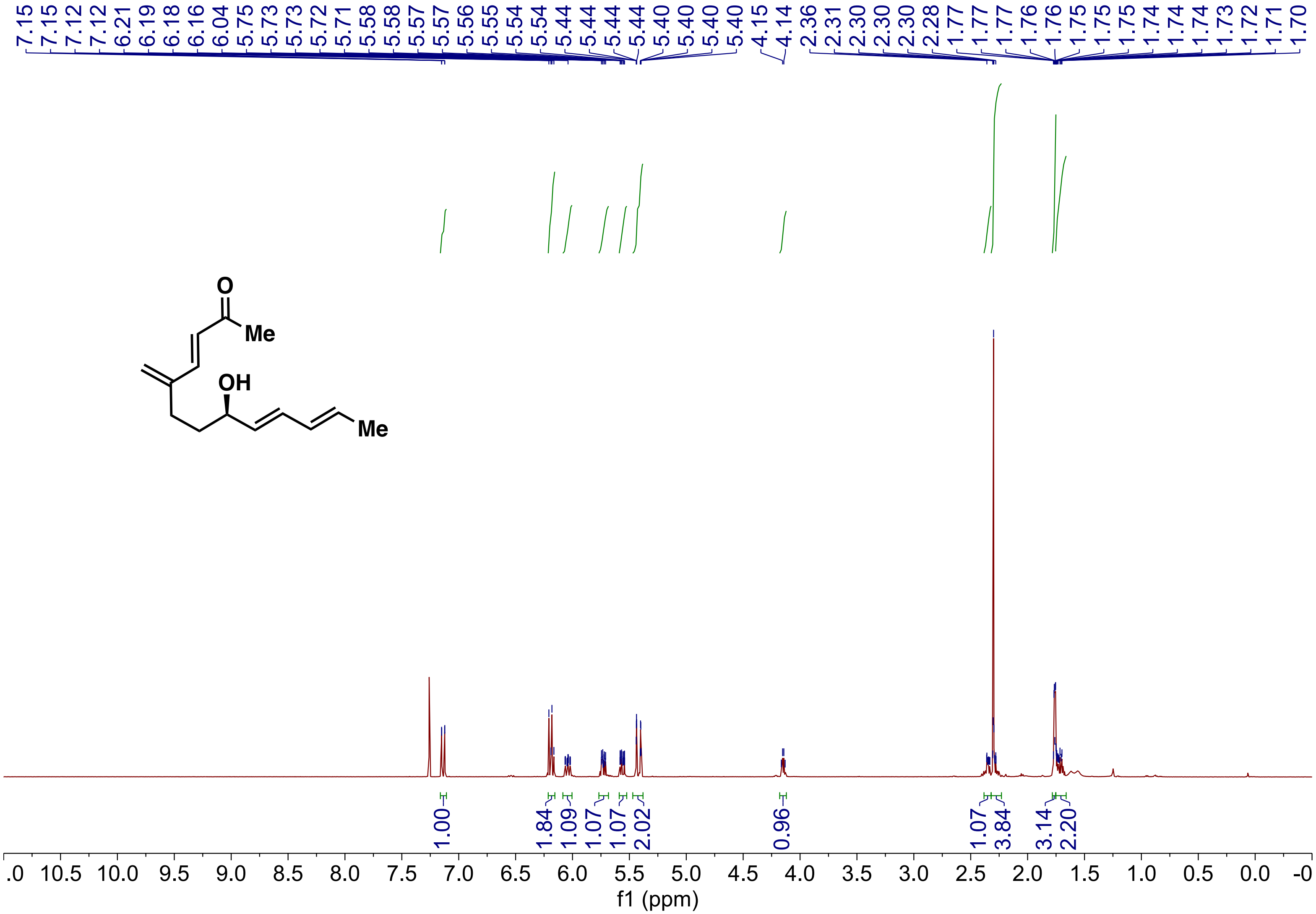
7.15 7.15 7.12 7.12 6.21 6.19 6.18 6.16 6.04 5.75 5.73 5.73 5.72 5.71 5.58 5.58 5.57 5.57 5.56 5.55 5.54 5.54 5.44 5.44 5.44 5.44 5.40 5.40 5.40 5.40 4.15 4.14 2.36 2.31 2.30 2.30 2.30 2.28 1.77 1.77 1.77 1.76 1.76 1.75 1.75 1.75 1.74 1.74 1.74 1.73 1.72 1.71 1.70
O
Me
OH
Me
1.00 1.84 1.09 1.07 1.07 2.02 0.96 1.07 3.84 3.14 2.20
.0 10.5 10.0 9.5 9.0 8.5 8.0 7.5 7.0 6.5 6.0 5.5 5.0 4.5 4.0 3.5 3.0 2.5 2.0 1.5 1.0 0.5 0.0 -0
f1 (ppm)

^{13}C NMR of **22**

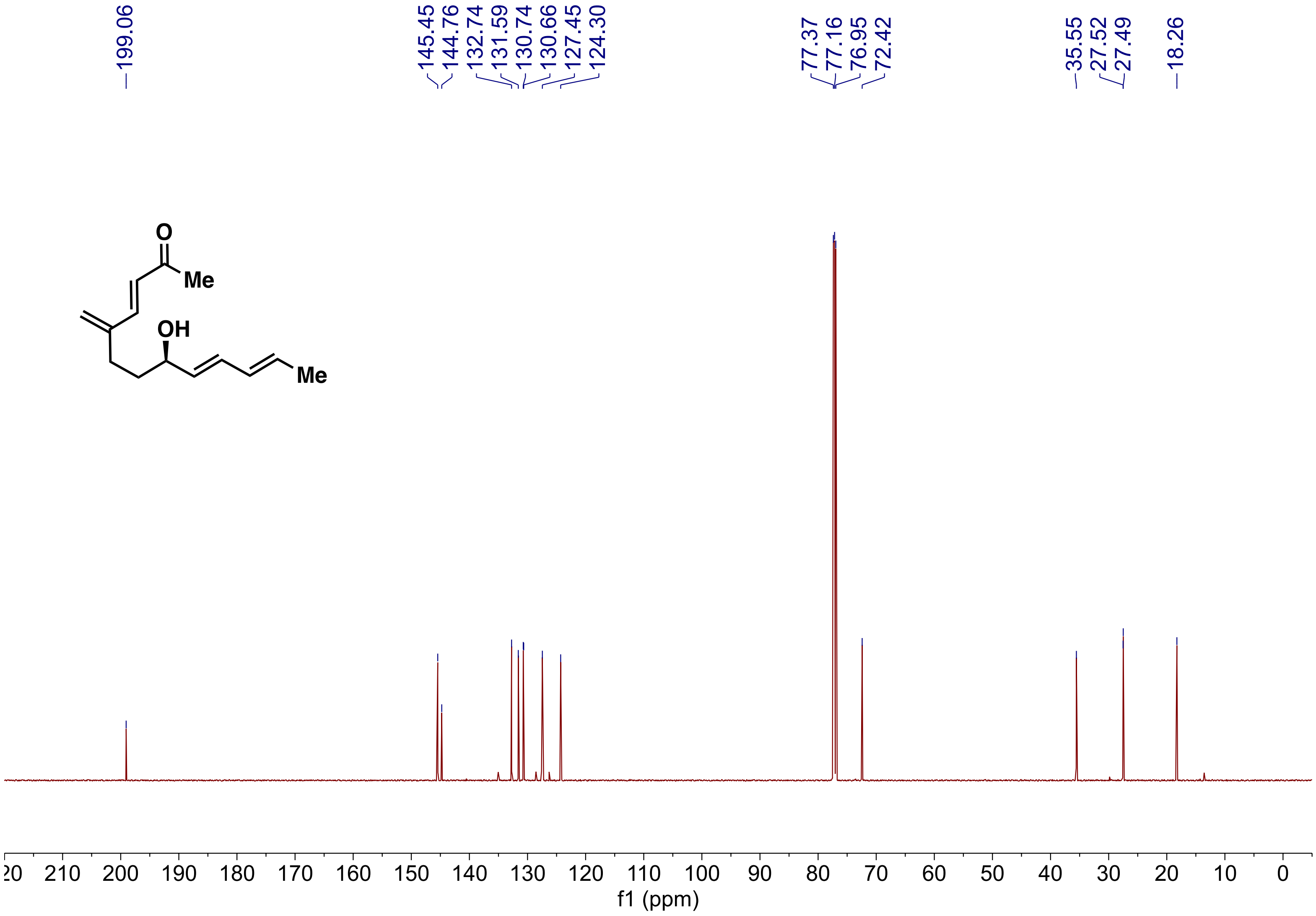
199.06
145.45
144.76
132.74
131.59
130.74
130.66
127.45
124.30
77.37
77.16
76.95
72.42
35.55
27.52
27.49
18.26
O
Me
OH
Me
20 210 200 190 180 170 160 150 140 130 120 110 100 90 80 70 60 50 40 30 20 10 0
f1 (ppm)

^{1}H NMR of **23**

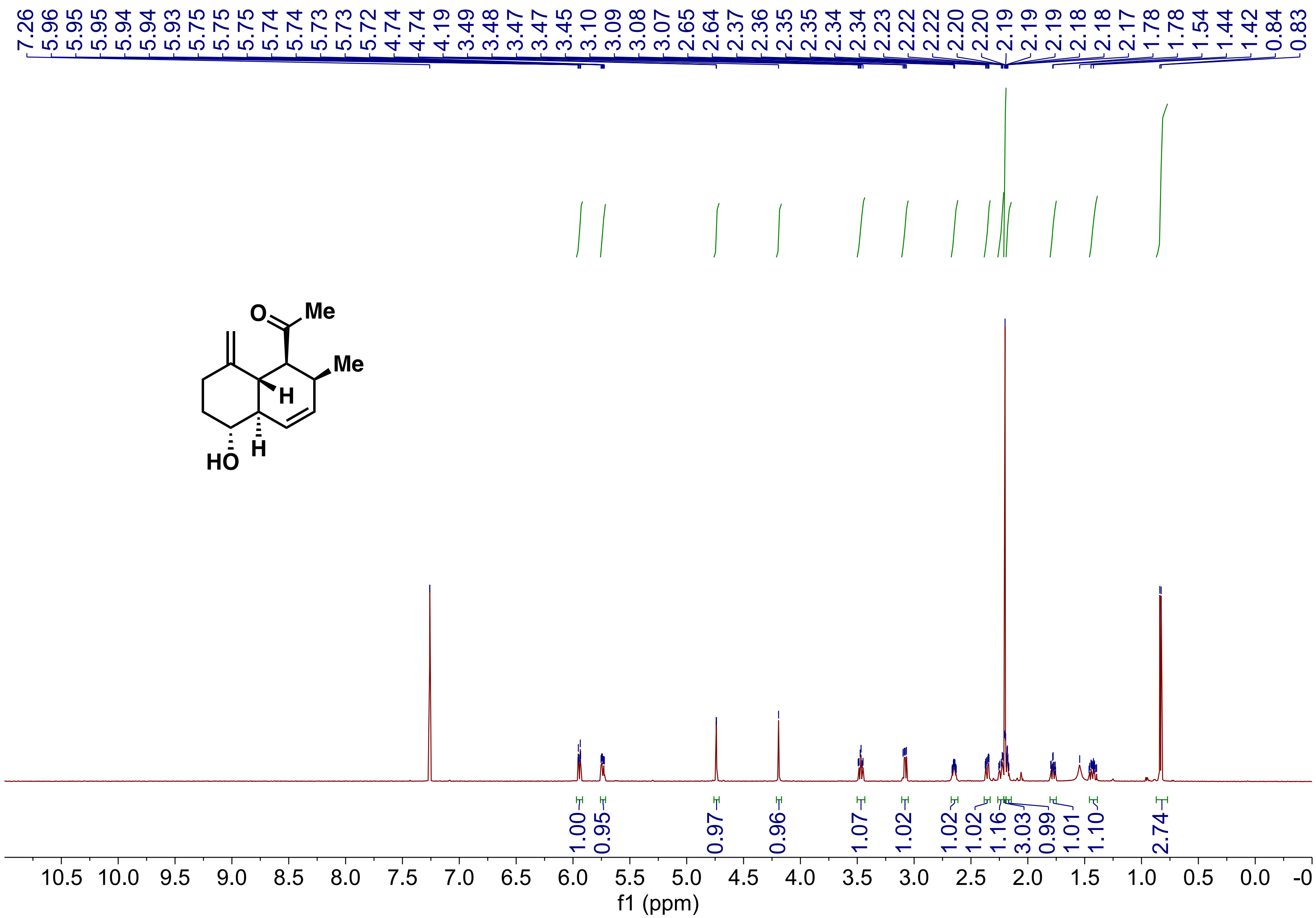

7.26
5.96
5.95
5.95
5.94
5.94
5.93
5.75
5.75
5.75
5.74
5.74
5.73
5.73
5.72
4.74
4.74
4.19
3.49
3.48
3.47
3.47
3.45
3.10
3.09
3.08
3.07
2.65
2.64
2.37
2.36
2.35
2.35
2.34
2.34
2.23
2.22
2.22
2.20
2.20
2.19
2.19
2.19
2.18
2.18
2.17
1.78
1.78
1.54
1.44
1.42
0.84
0.83
O
Me
Me
H
H
HO
1.00
0.95
0.97
0.96
1.07
1.02
1.02
1.02
1.16
3.03
0.99
1.01
1.10
2.74
10.5 10.0 9.5 9.0 8.5 8.0 7.5 7.0 6.5 6.0 5.5 5.0 4.5 4.0 3.5 3.0 2.5 2.0 1.5 1.0 0.5 0.0 -0
f1 (ppm)

^{13}C NMR of **23**

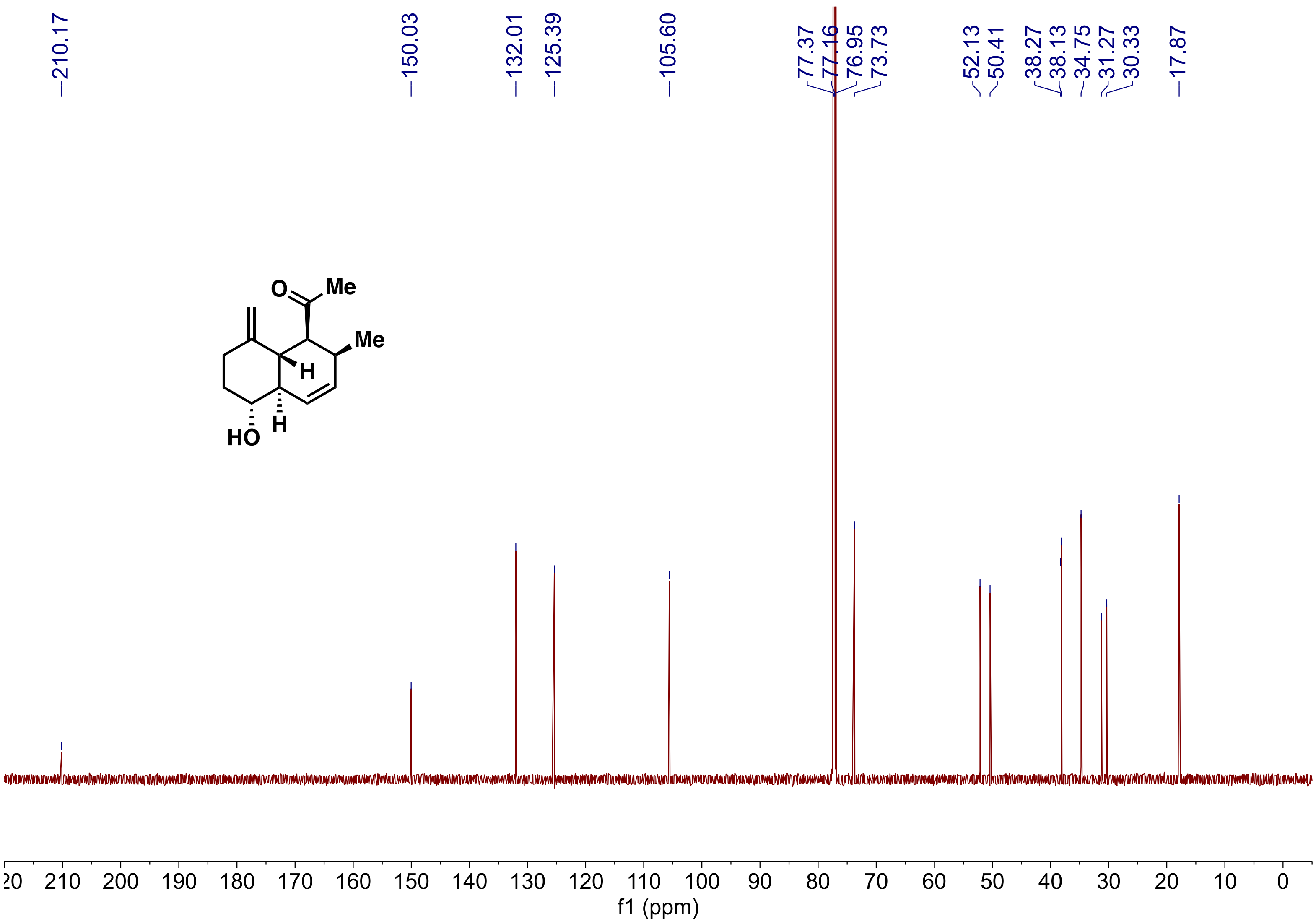

210.17
150.03
132.01
125.39
105.60
77.37
77.16
76.95
73.73
52.13
50.41
38.27
38.13
34.75
31.27
30.33
17.87
O
Me
Me
H
H
HO
20 210 200 190 180 170 160 150 140 130 120 110 100 90 80 70 60 50 40 30 20 10 0
f1 (ppm)

^{1}H NMR of **S4**

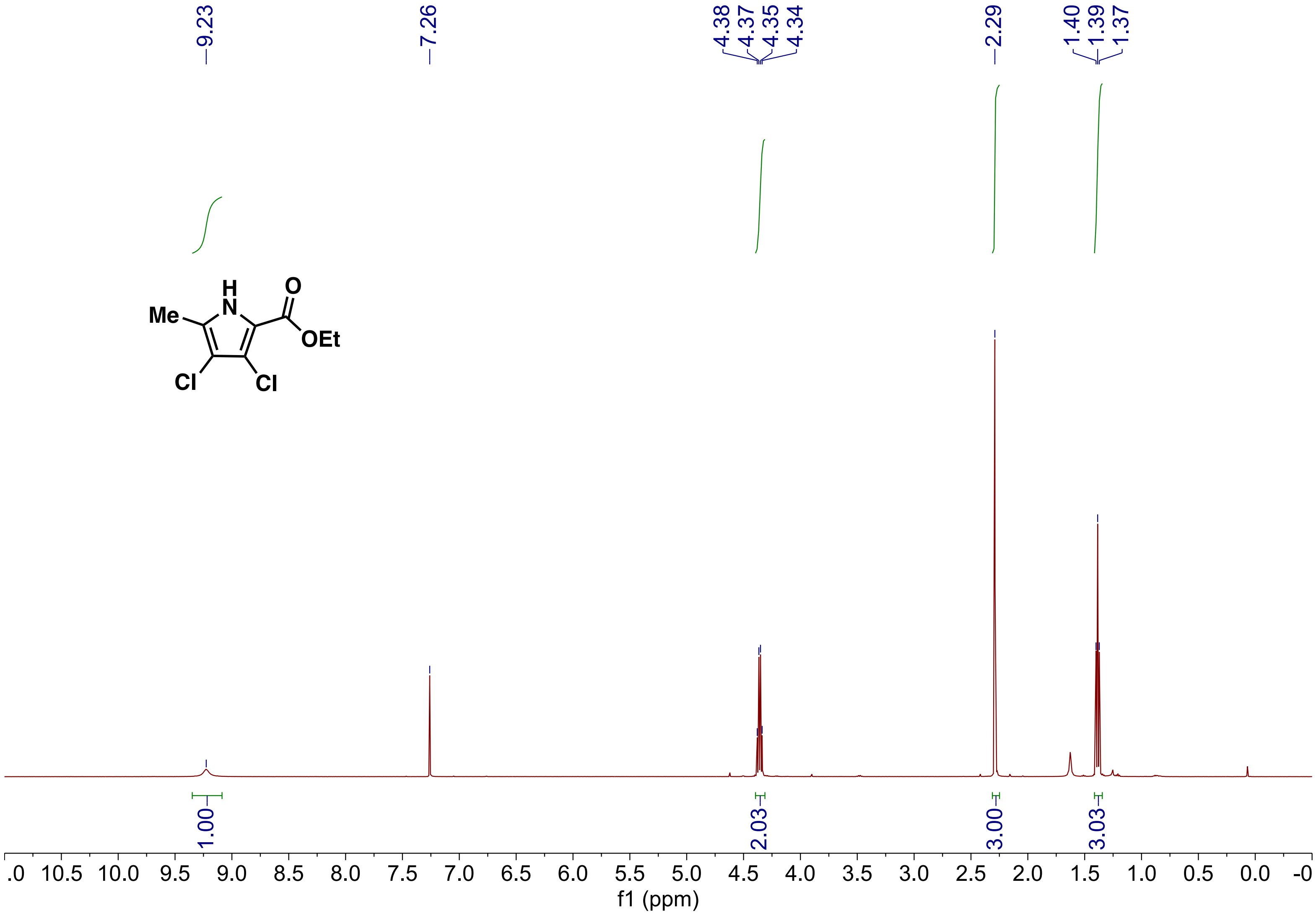

9.23
7.26
4.38
4.37
4.35
4.34
2.29
1.40
1.39
1.37
Me
H
N
O
OEt
Cl
Cl
1.00
2.03
3.00
3.03
.0
10.5
10.0
9.5
9.0
8.5
8.0
7.5
7.0
6.5
6.0
5.5
5.0
4.5
4.0
3.5
3.0
2.5
2.0
1.5
1.0
0.5
0.0
-0
f1 (ppm)

^{13}C NMR of **S4**

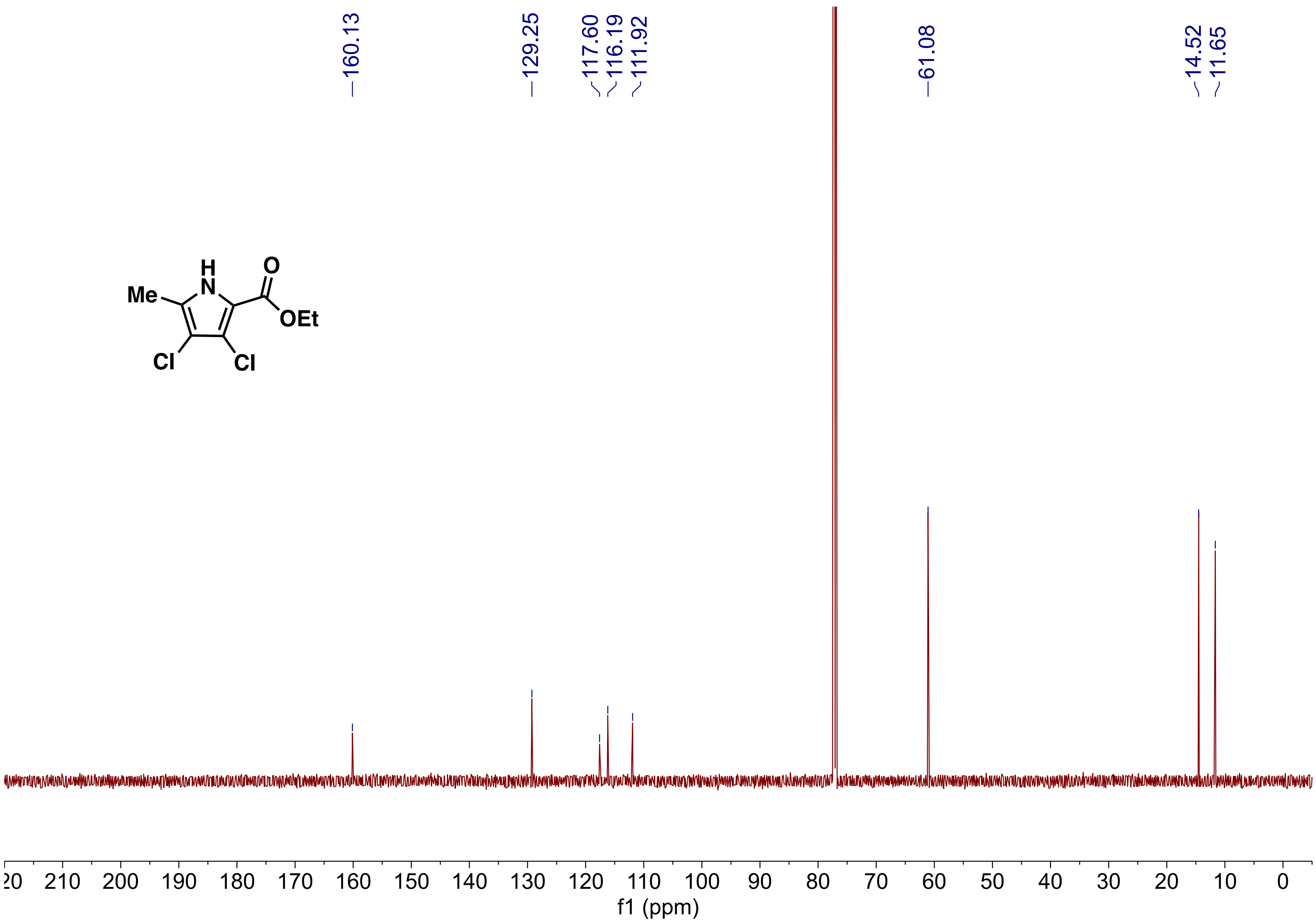

H
N
Me
O
OEt
Cl
Cl
160.13
129.25
117.60
116.19
111.92
61.08
14.52
11.65
20
210
200
190
180
170
160
150
140
130
120
110
100
90
80
70
60
50
40
30
20
10
0
f1 (ppm)

^{1}H NMR of **28**

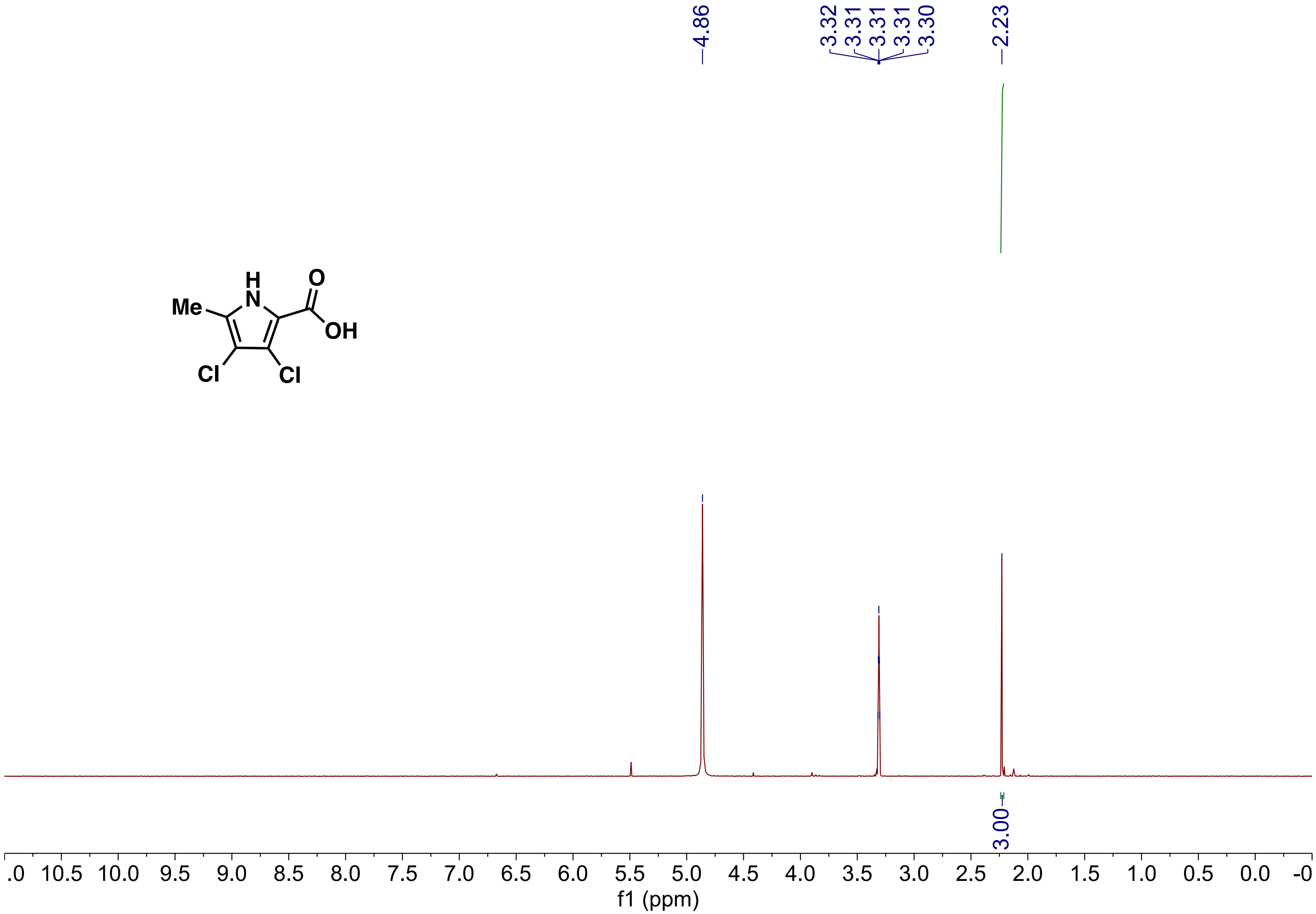

4.86
3.32
3.31
3.31
3.31
3.30
2.23
3.00
.0
10.5
10.0
9.5
9.0
8.5
8.0
7.5
7.0
6.5
6.0
5.5
5.0
4.5
4.0
3.5
3.0
2.5
2.0
1.5
1.0
0.5
0.0
-0
f1 (ppm)

^{1}H NMR of **6**

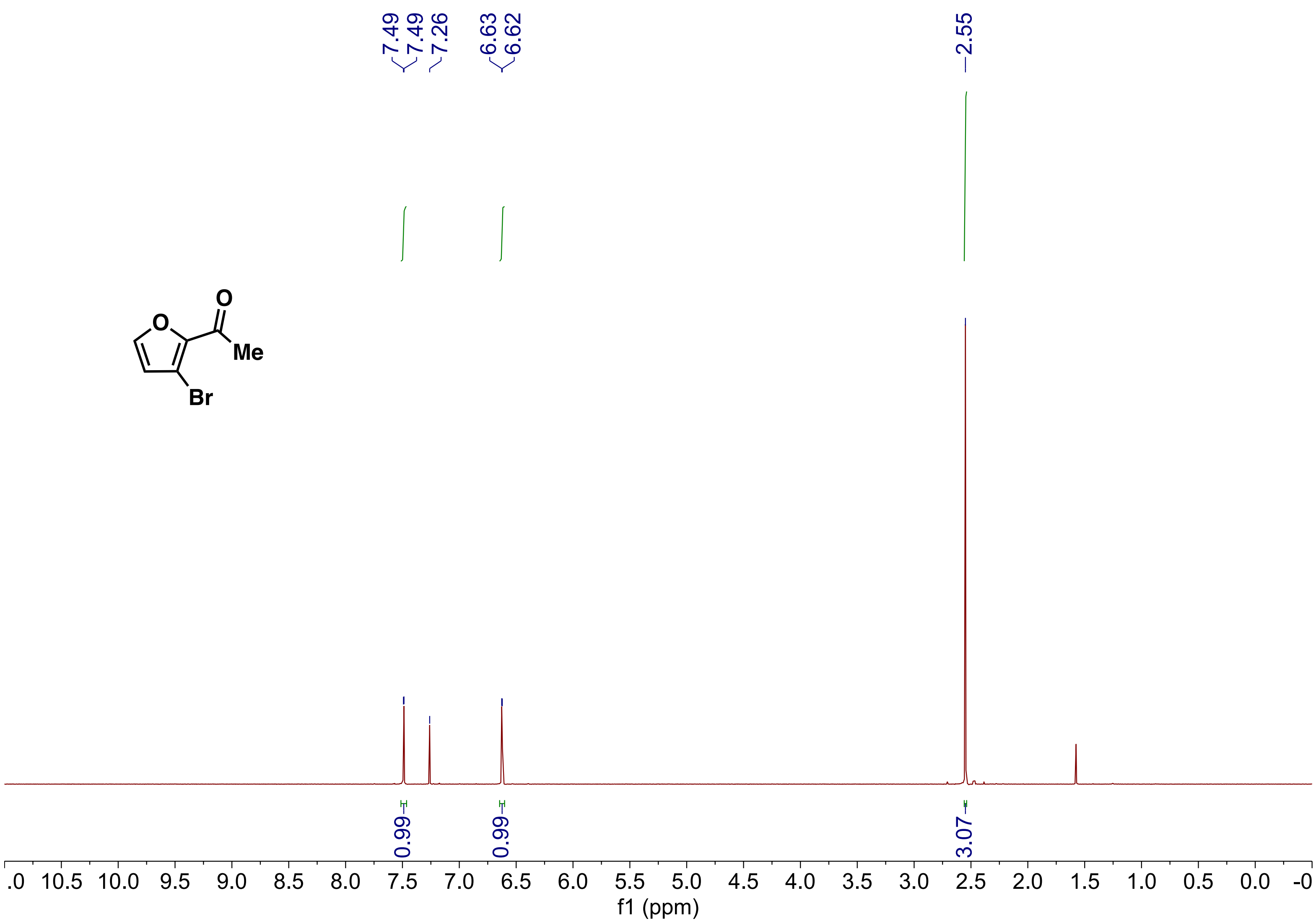

7.49
7.49
7.26
6.63
6.62
2.55
O
O
Me
Br
0.99
0.99
3.07
.0 10.5 10.0 9.5 9.0 8.5 8.0 7.5 7.0 6.5 6.0 5.5 5.0 4.5 4.0 3.5 3.0 2.5 2.0 1.5 1.0 0.5 0.0 -0
f1 (ppm)

^{13}C NMR of **6**

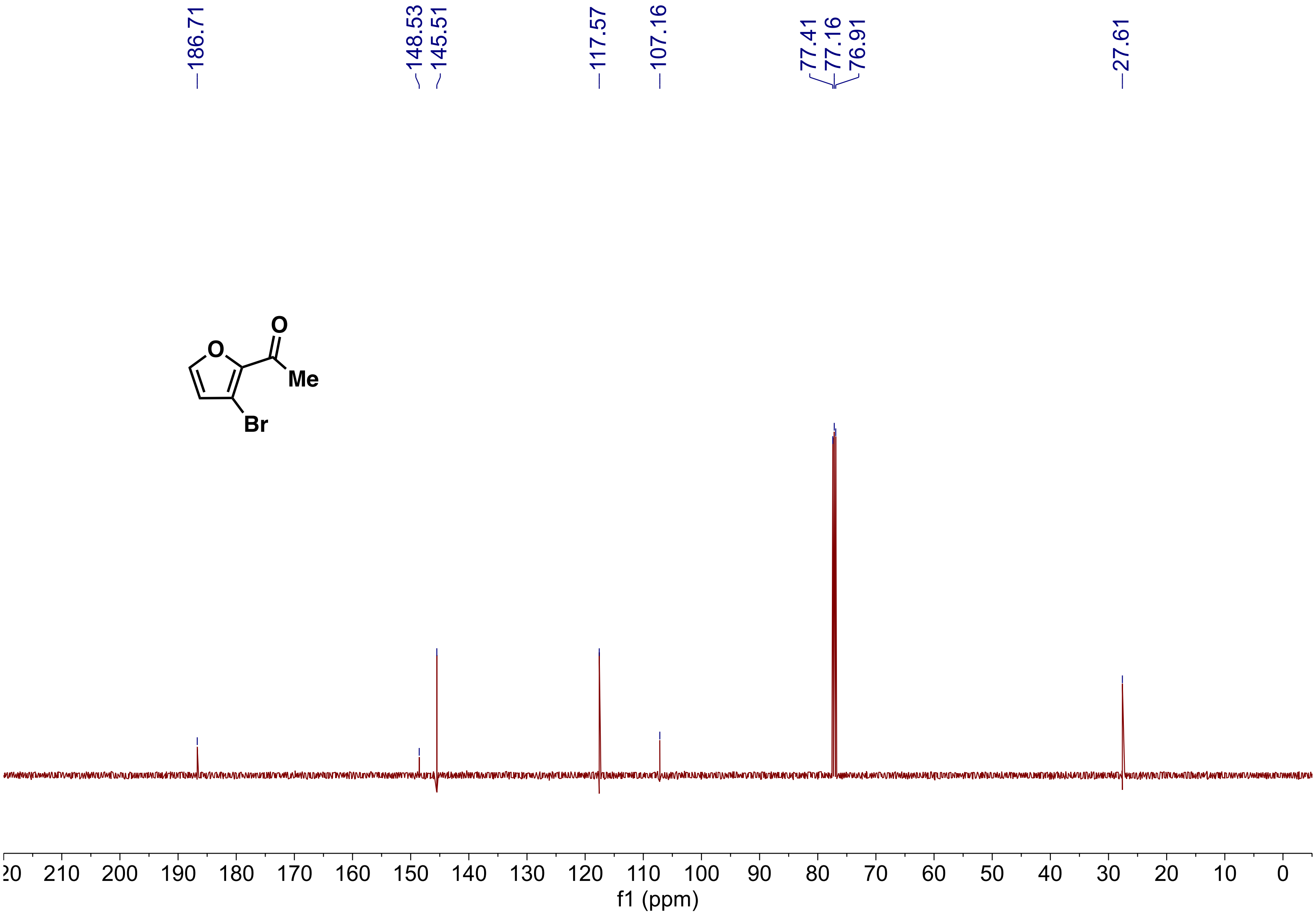

186.71
148.53
145.51
117.57
107.16
77.41
77.16
76.91
27.61
O
Me
O
Br
20 210 200 190 180 170 160 150 140 130 120 110 100 90 80 70 60 50 40 30 20 10 0
f1 (ppm)

^{1}H NMR of **S6**

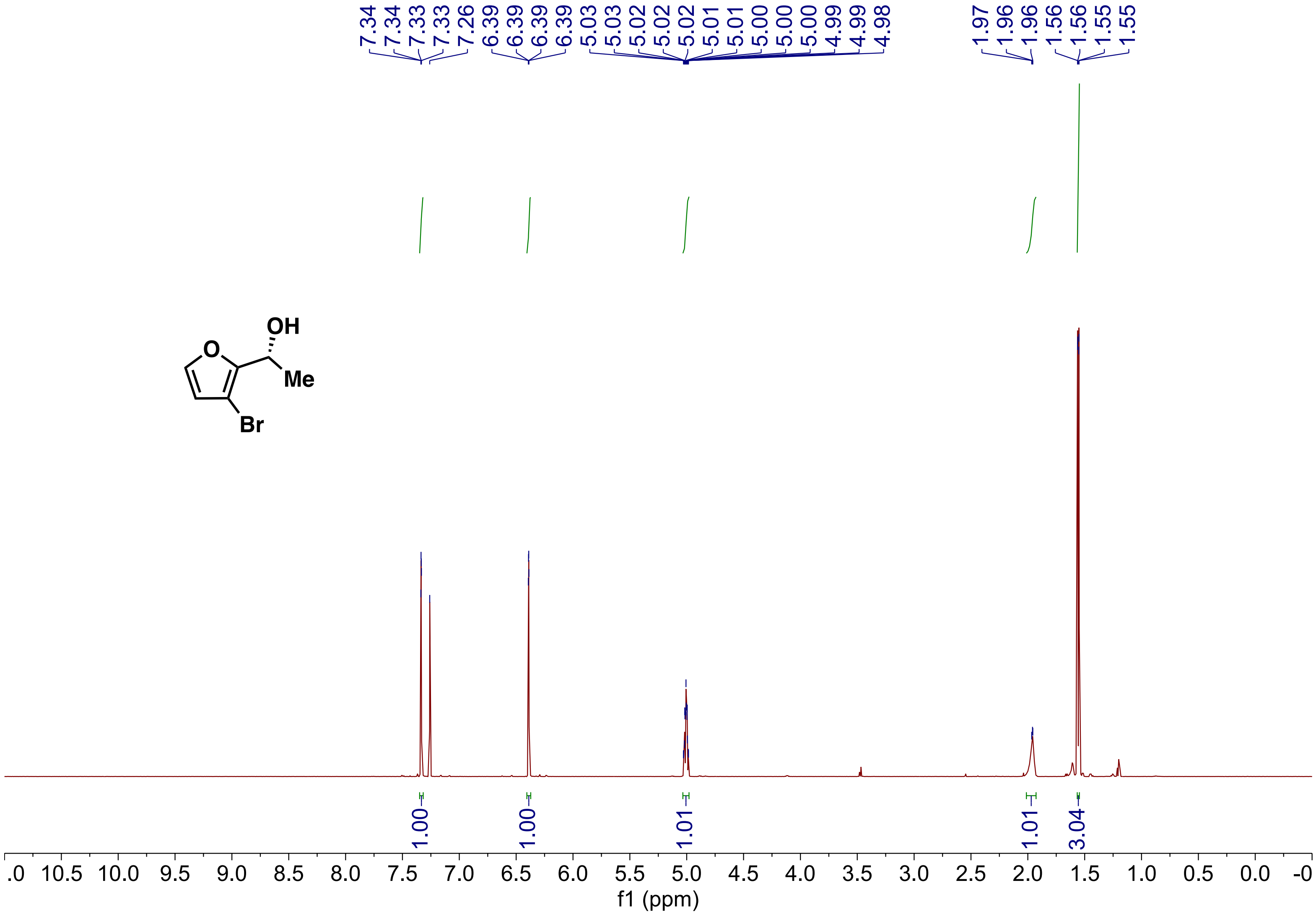
7.34
7.34
7.33
7.33
7.26
6.39
6.39
6.39
6.39
5.03
5.03
5.02
5.02
5.02
5.01
5.01
5.00
5.00
5.00
4.99
4.99
4.98
1.97
1.96
1.96
1.56
1.56
1.55
1.55
OH
O
Me
Br
1.00
1.00
1.01
1.01
3.04
.0
10.5
10.0
9.5
9.0
8.5
8.0
7.5
7.0
6.5
6.0
5.5
5.0
4.5
4.0
3.5
3.0
2.5
2.0
1.5
1.0
0.5
0.0
-0
f1 (ppm)

^{13}C NMR of **S6**

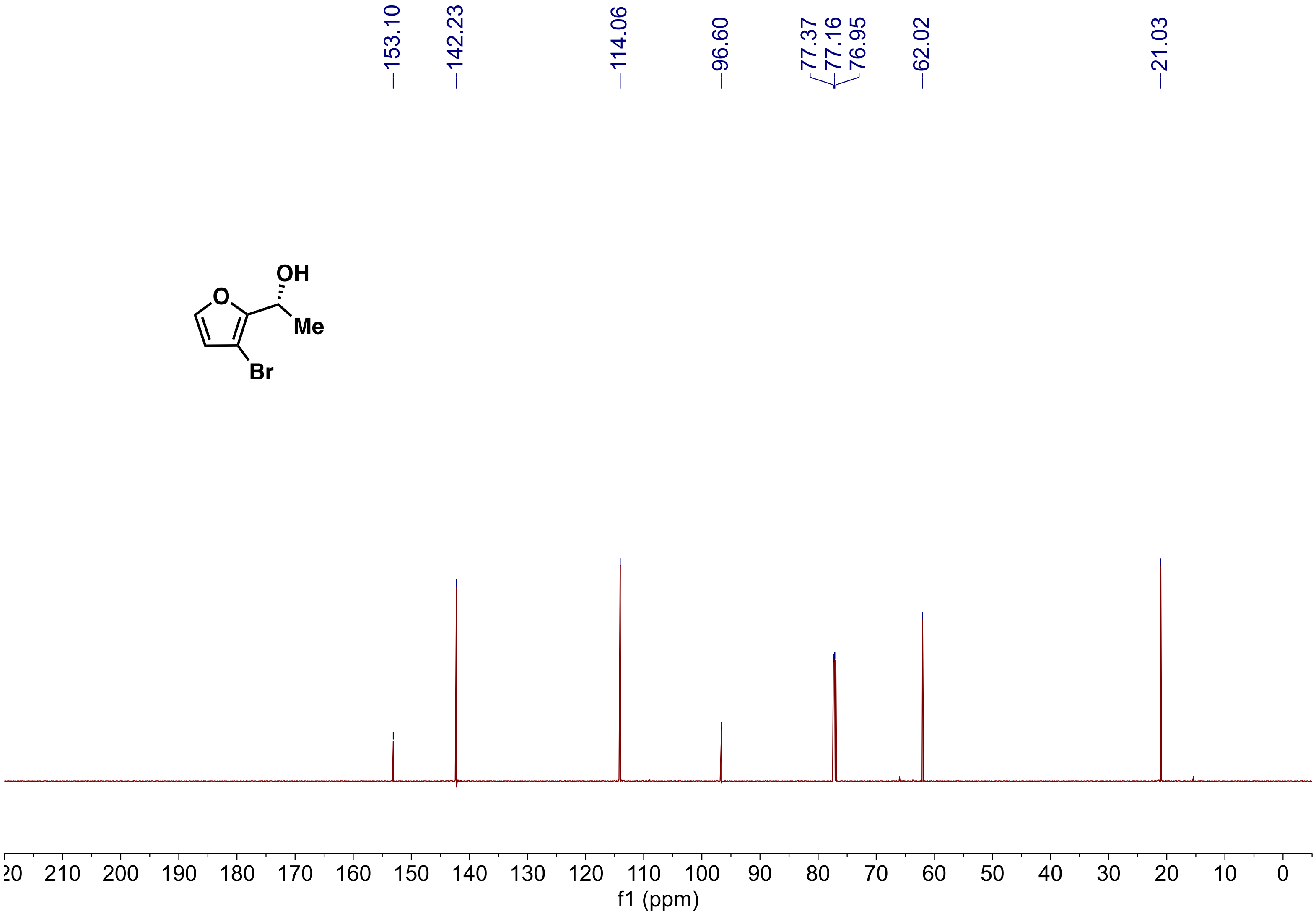

OH
O
Me
Br
153.10
142.23
114.06
96.60
77.37
77.16
76.95
62.02
21.03
20
210
200
190
180
170
160
150
140
130
120
110
100
90
80
70
60
50
40
30
20
10
0
f1 (ppm)

^{1}H NMR of **24**

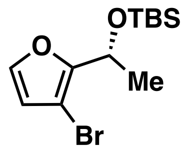
OTBS
O
Me
Br

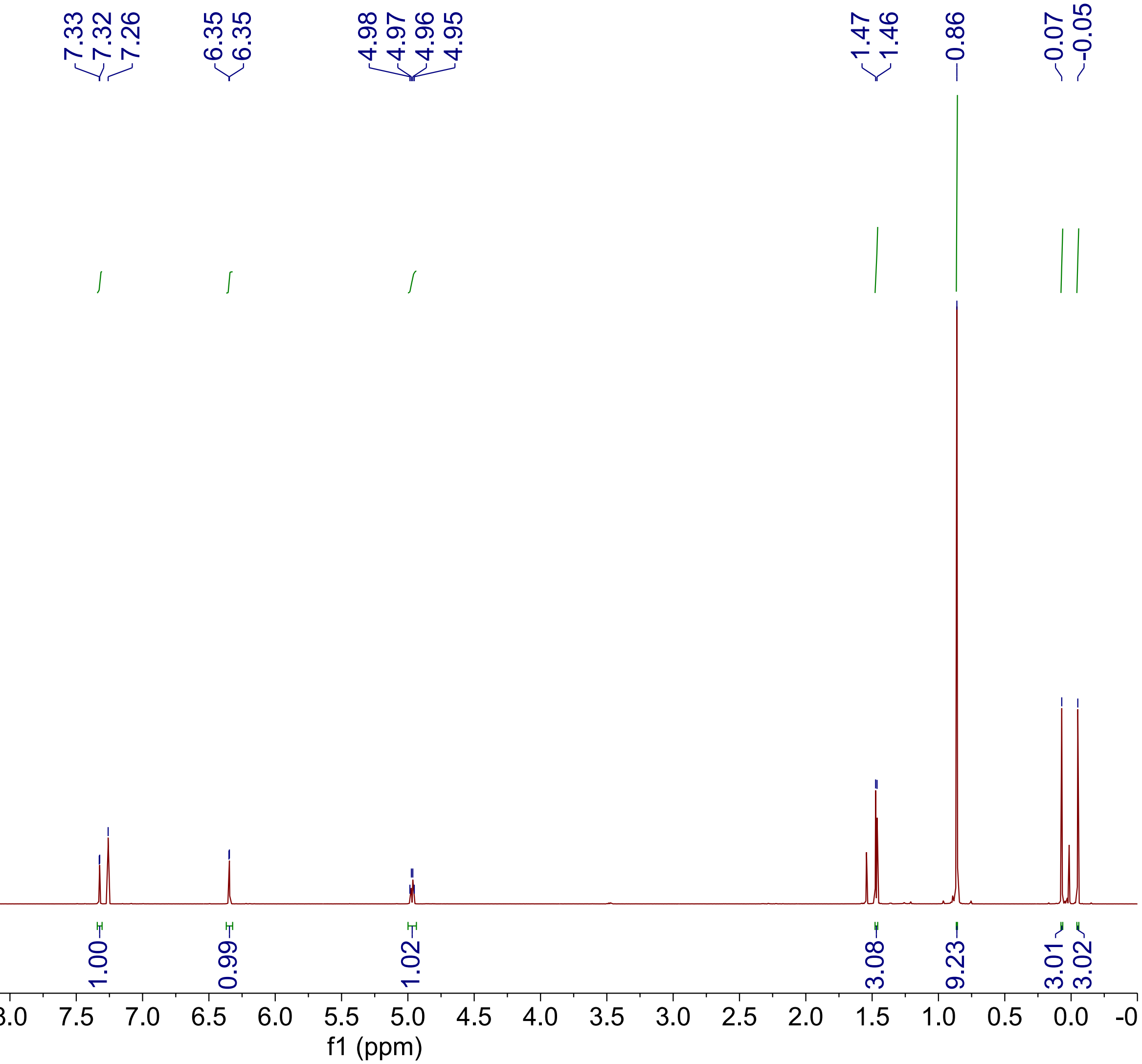
7.33
7.32
7.26
6.35
6.35
4.98
4.97
4.96
4.95
1.47
1.46
0.86
0.07
-0.05
1.00
0.99
1.02
3.08
9.23
3.01
3.02
10.5 10.0 9.5 9.0 8.5 8.0 7.5 7.0 6.5 6.0 5.5 5.0 4.5 4.0 3.5 3.0 2.5 2.0 1.5 1.0 0.5 0.0 -0
f1 (ppm)

^{13}C NMR of **24**

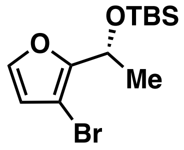
OTBS
O
Me
Br

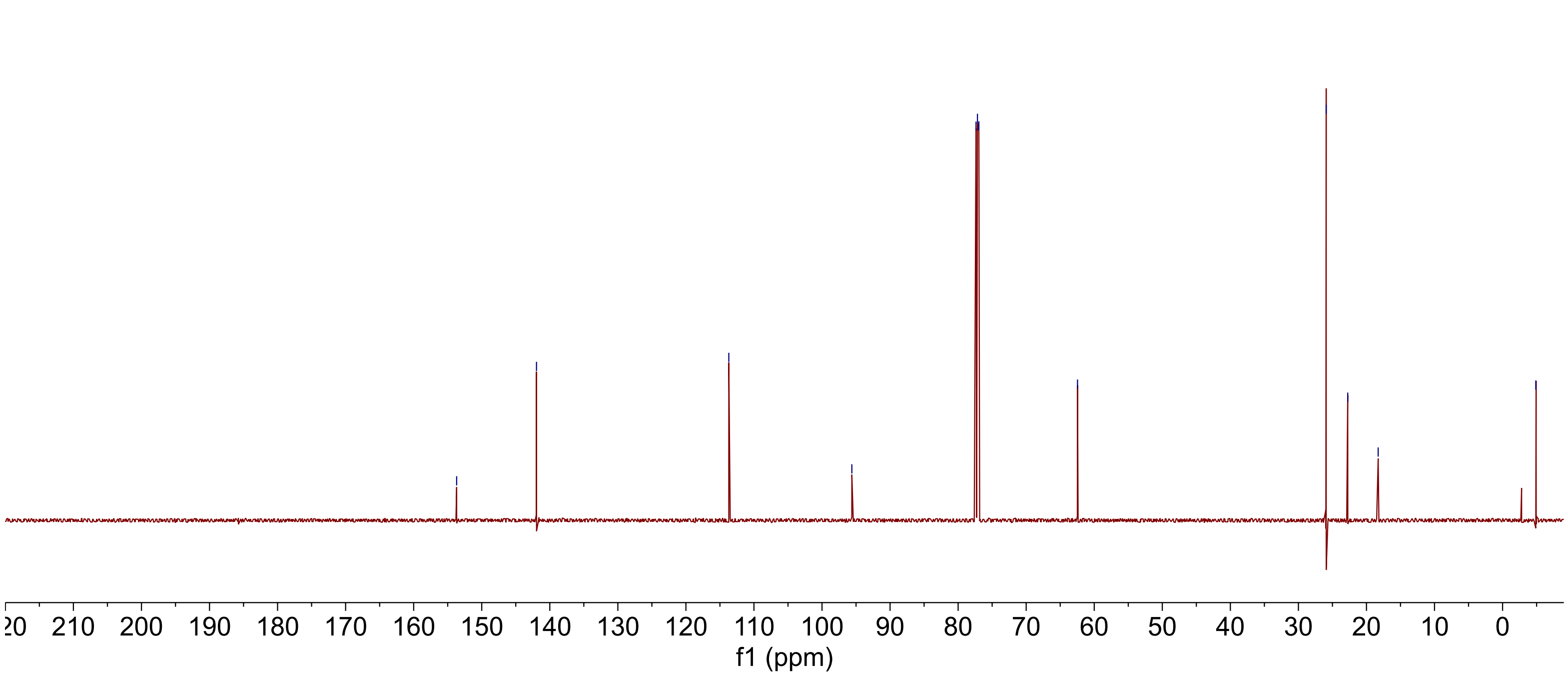
153.69
141.95
113.69
95.61
77.37
77.16
76.95
62.46
25.92
22.75
18.28
-4.88
20 210 200 190 180 170 160 150 140 130 120 110 100 90 80 70 60 50 40 30 20 10 0
f1 (ppm)

^{1}H NMR of **25**

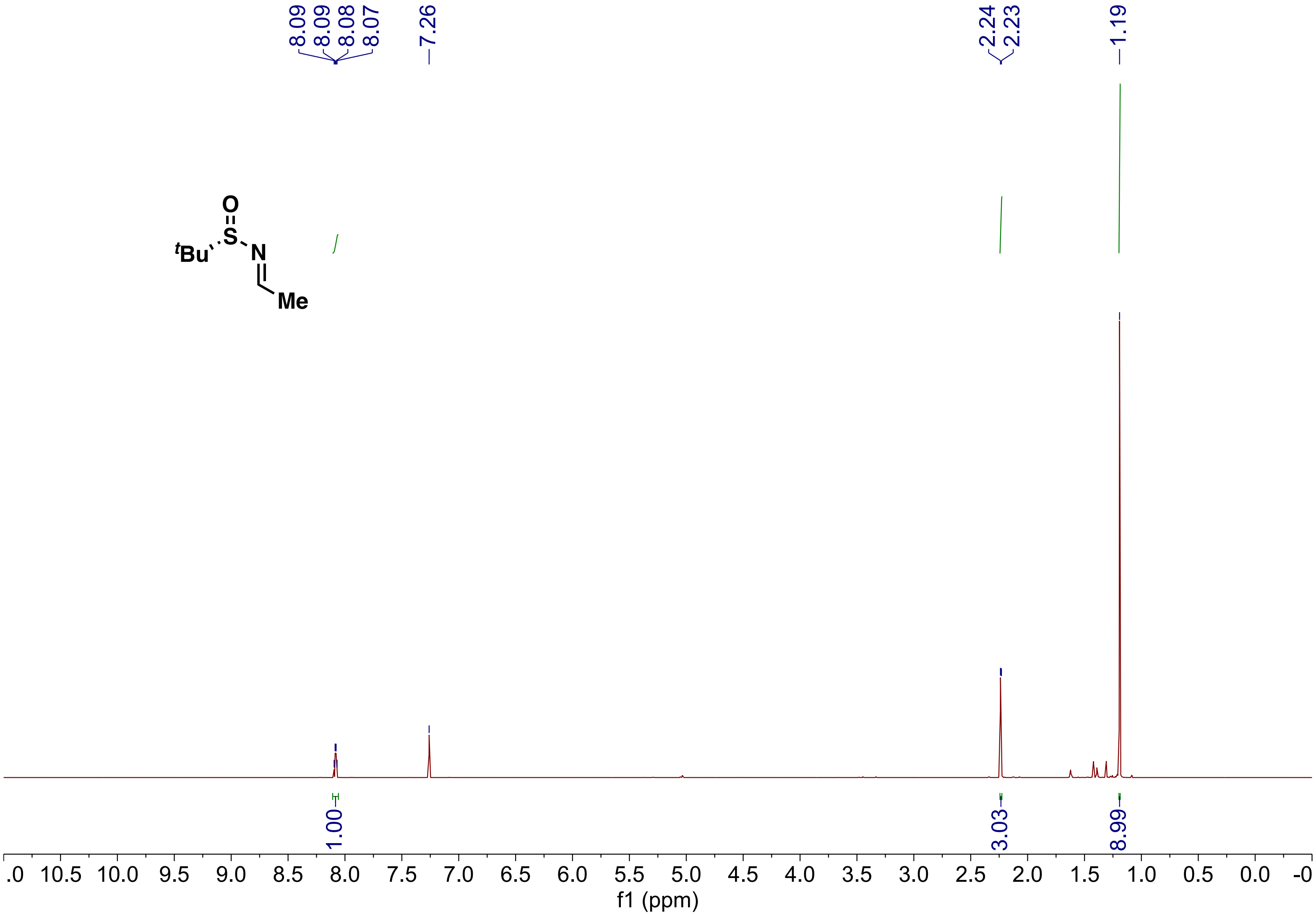

8.09
8.09
8.08
8.07
7.26
2.24
2.23
1.19
O
S
N
ᵗBu
Me
1.00
3.03
8.99
.0 10.5 10.0 9.5 9.0 8.5 8.0 7.5 7.0 6.5 6.0 5.5 5.0 4.5 4.0 3.5 3.0 2.5 2.0 1.5 1.0 0.5 0.0 -0
f1 (ppm)

^{13}C NMR of **25**

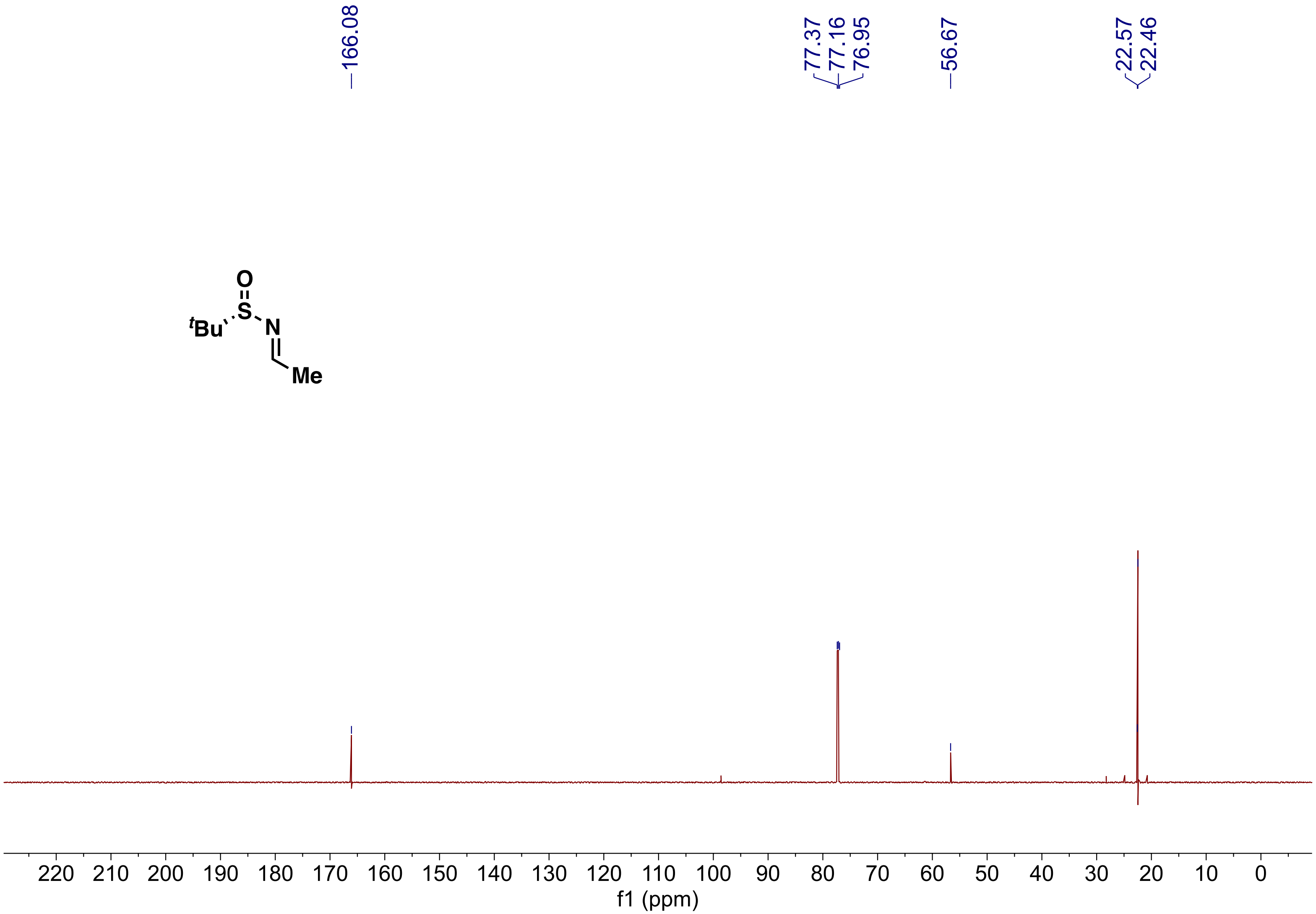
166.08
77.37
77.16
76.95
56.67
22.57
22.46
O
S
tBu
N
Me
220 210 200 190 180 170 160 150 140 130 120 110 100 90 80 70 60 50 40 30 20 10 0
f1 (ppm)

^{1}H NMR of **26**

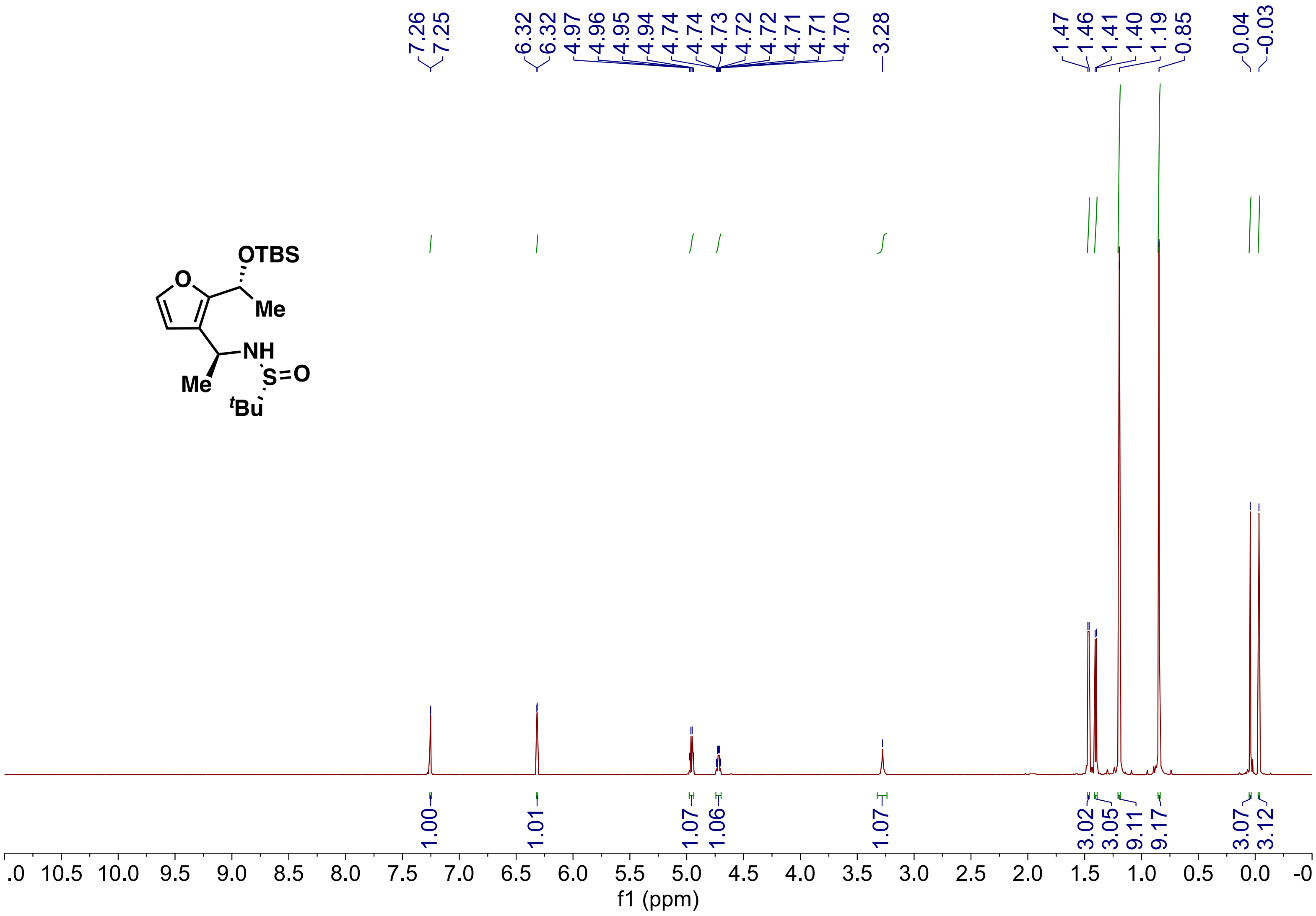
7.26
7.25
6.32
6.32
4.97
4.96
4.95
4.94
4.74
4.74
4.73
4.72
4.72
4.71
4.71
4.70
3.28
1.47
1.46
1.41
1.40
1.19
0.85
0.04
-0.03
OTBS
O
Me
NH
S=O
Me
tBu
1.00
1.01
1.07
1.06
1.07
3.02
3.05
9.11
9.17
3.07
3.12
.0
10.5
10.0
9.5
9.0
8.5
8.0
7.5
7.0
6.5
6.0
5.5
5.0
4.5
4.0
3.5
3.0
2.5
2.0
1.5
1.0
0.5
0.0
-0
f1 (ppm)

^{13}C NMR of **26**

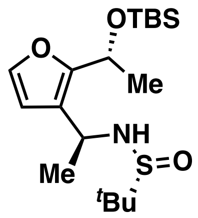

OTBS
O
Me
NH
S=O
Me
tBu

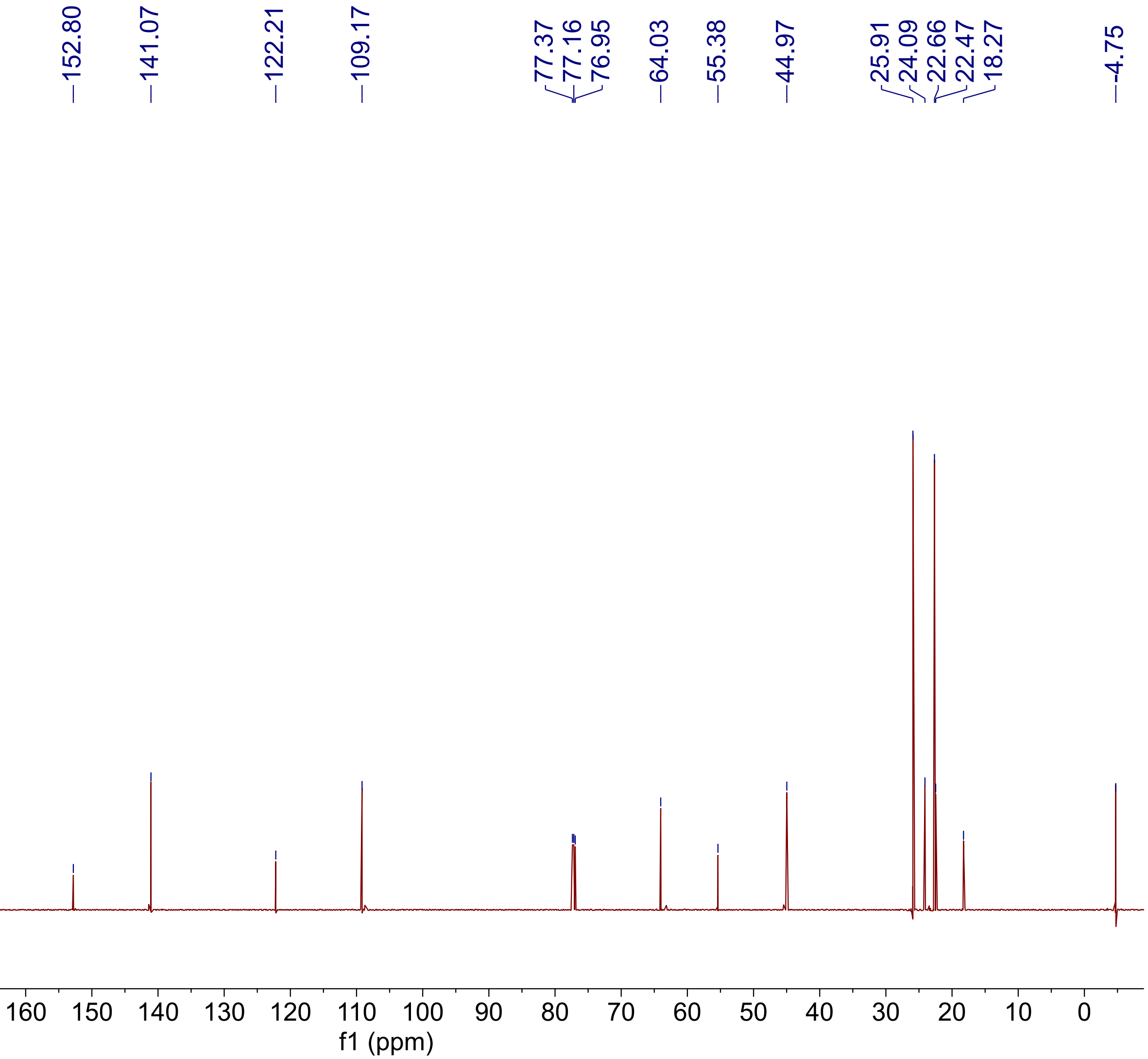

152.80
141.07
122.21
109.17
77.37
77.16
76.95
64.03
55.38
44.97
25.91
24.09
22.66
22.47
18.27
-4.75
20
210
200
190
180
170
160
150
140
130
120
110
100
90
80
70
60
50
40
30
20
10
0
f1 (ppm)

^{1}H NMR of **26B**

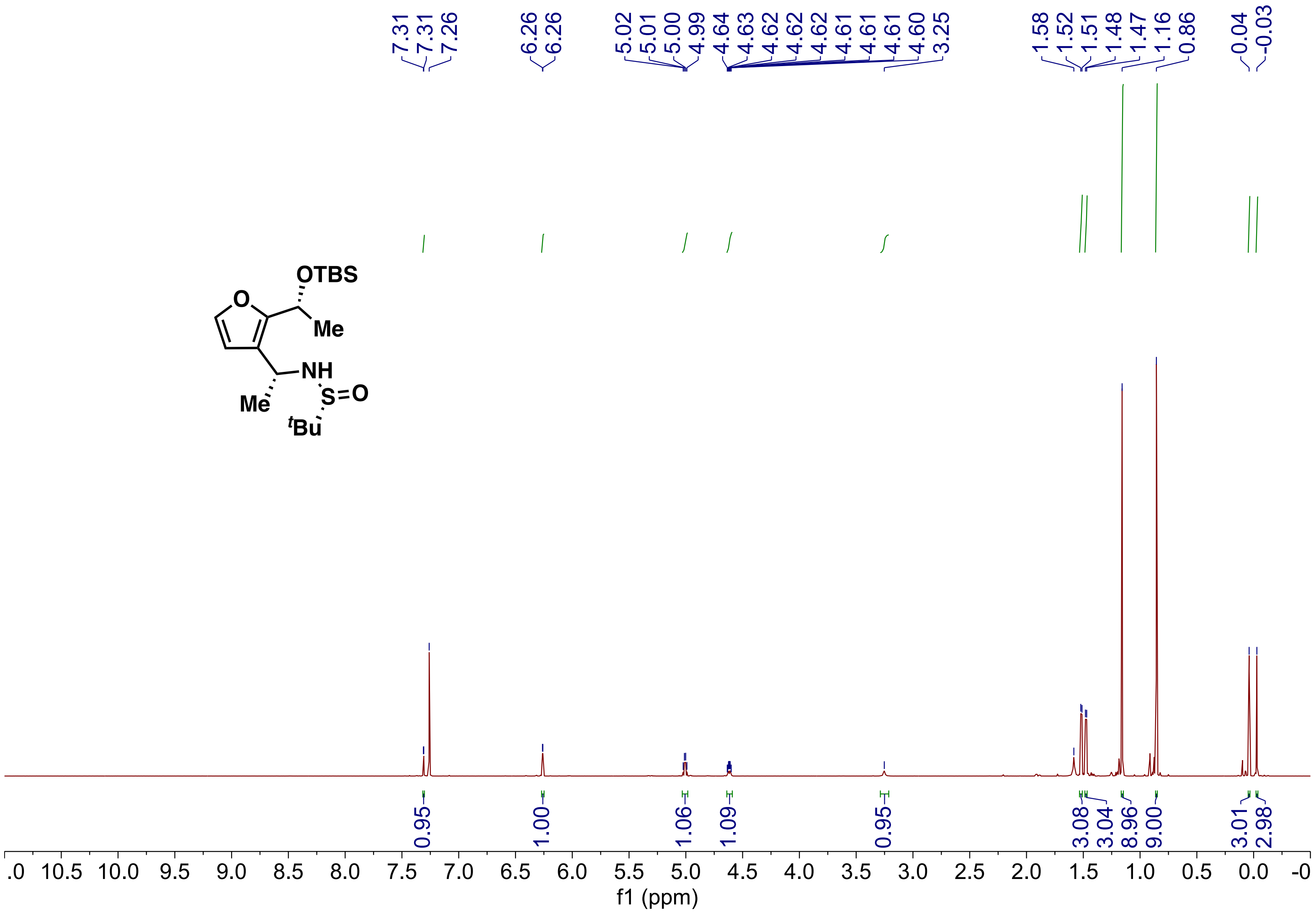

7.31
7.31
7.26
6.26
6.26
5.02
5.01
5.00
4.99
4.64
4.63
4.62
4.62
4.62
4.61
4.61
4.61
4.60
3.25
1.58
1.52
1.51
1.48
1.47
1.16
0.86
0.04
-0.03
OTBS
O
Me
NH
S=O
Me
tBu
0.95
1.00
1.06
1.09
0.95
3.08
3.04
8.96
9.00
3.01
2.98
.0 10.5 10.0 9.5 9.0 8.5 8.0 7.5 7.0 6.5 6.0 5.5 5.0 4.5 4.0 3.5 3.0 2.5 2.0 1.5 1.0 0.5 0.0 -0
f1 (ppm)

^{13}C NMR of **26B**

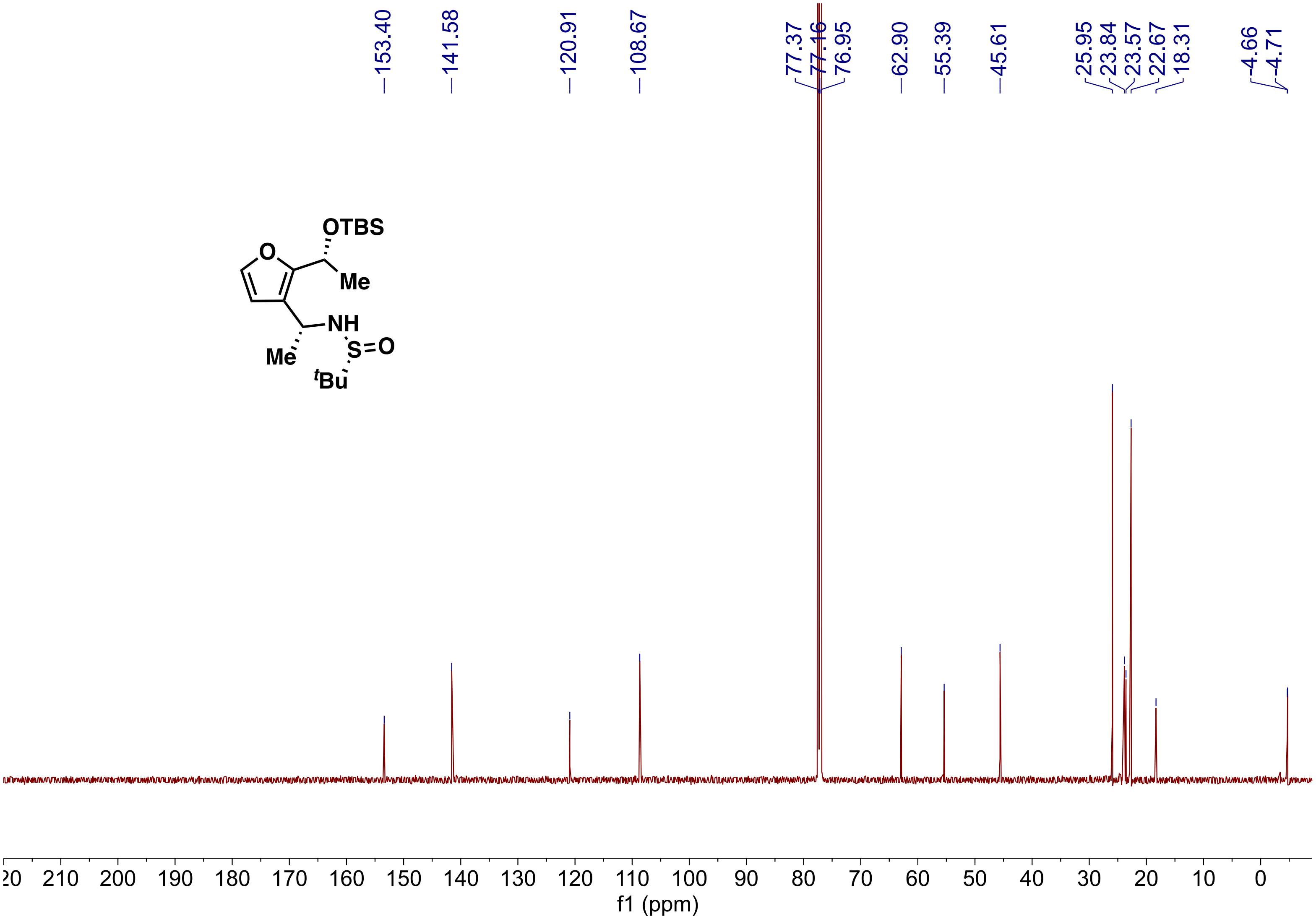

^{1}H NMR of **S7**

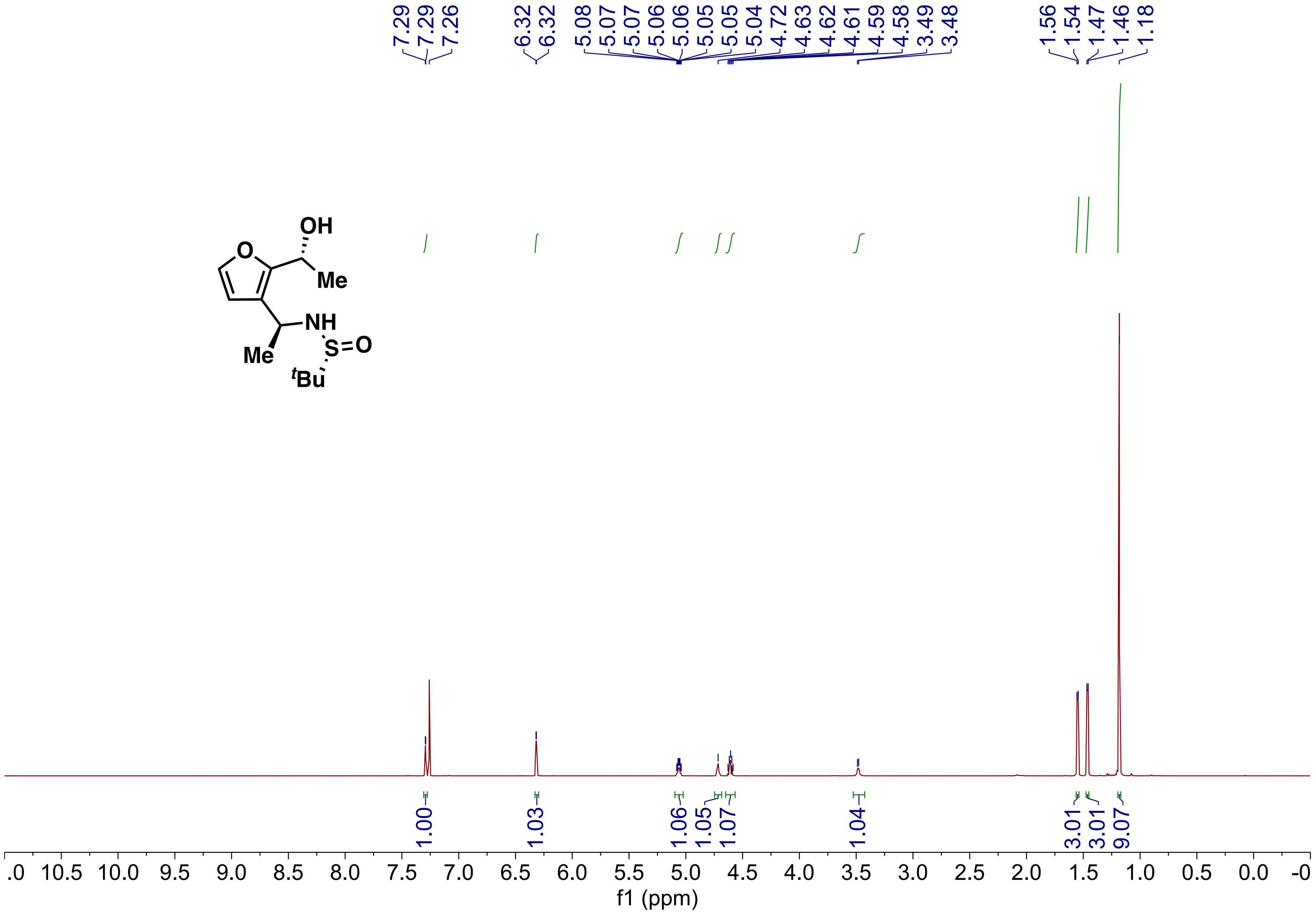

^{13}C NMR of **S7**

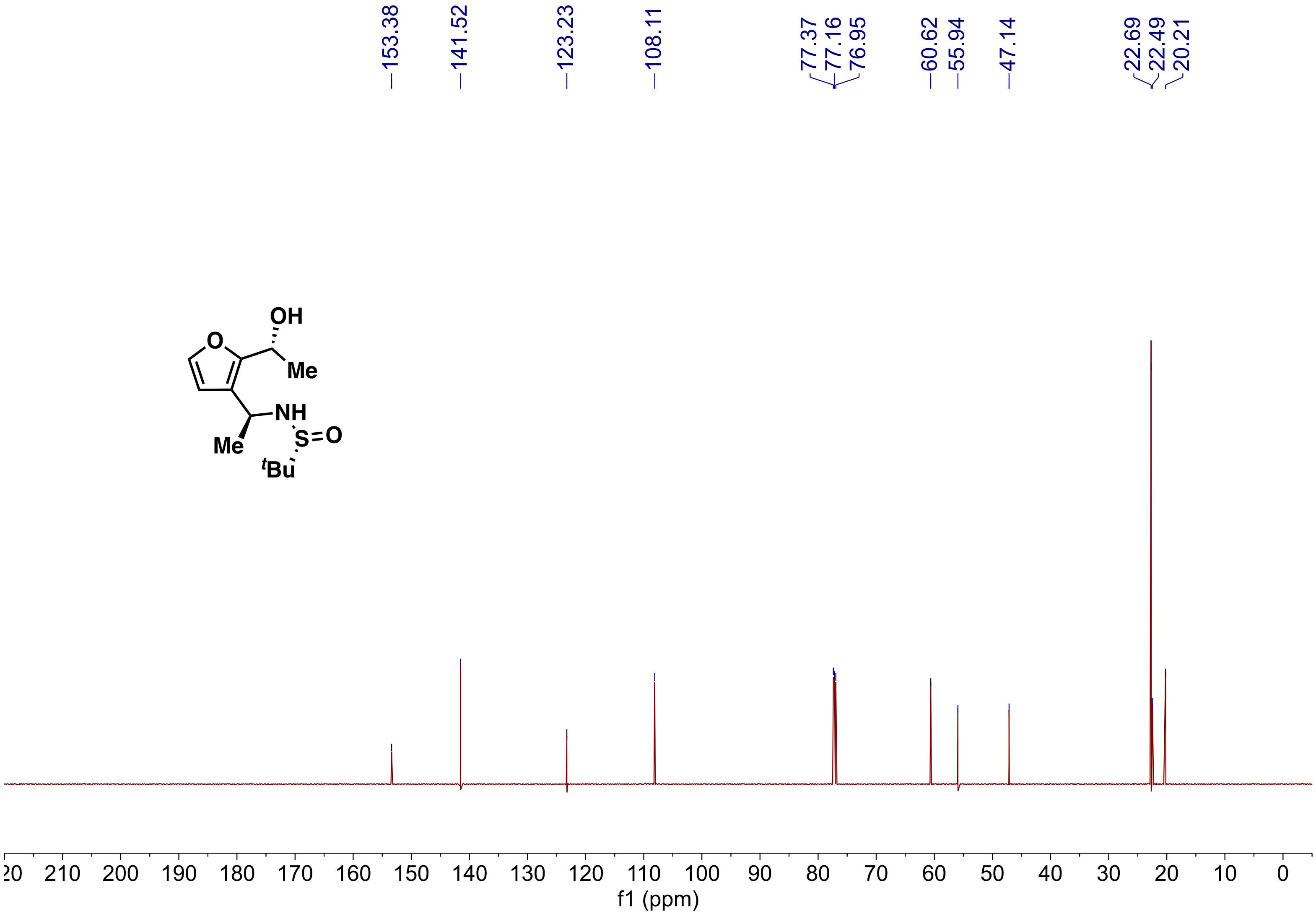

153.38
141.52
123.23
108.11
77.37
77.16
76.95
60.62
55.94
47.14
22.69
22.49
20.21
OH
O
Me
NH
S=O
Me
^{t}Bu
20
210
200
190
180
170
160
150
140
130
120
110
100
90
80
70
60
50
40
30
20
10
0
f1 (ppm)

^{1}H NMR of **27**

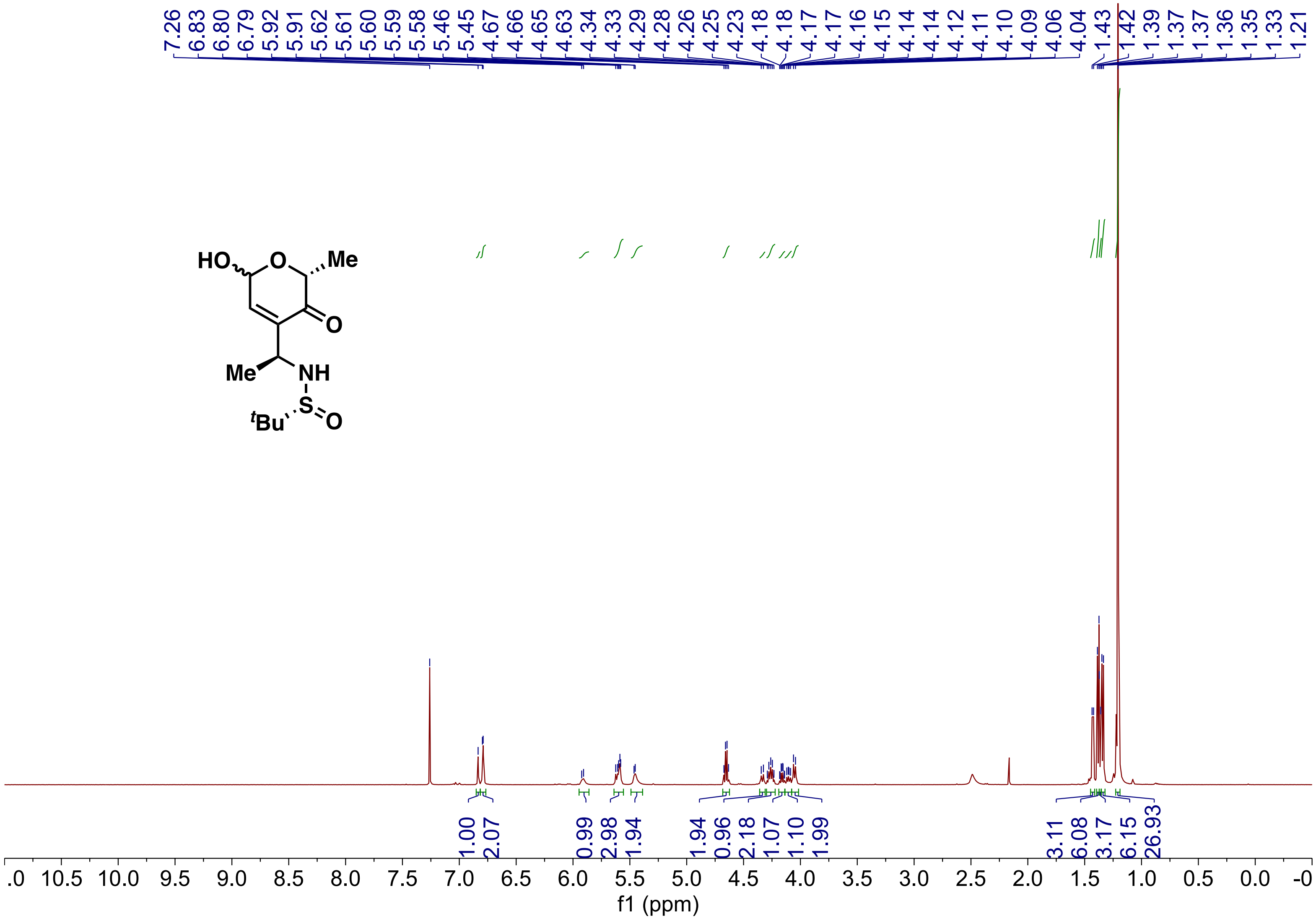

7.26
6.83
6.80
6.79
5.92
5.91
5.62
5.61
5.60
5.59
5.58
5.46
5.45
4.67
4.66
4.65
4.63
4.34
4.33
4.29
4.28
4.26
4.25
4.23
4.18
4.18
4.17
4.17
4.16
4.15
4.14
4.14
4.12
4.11
4.10
4.09
4.06
4.04
1.43
1.42
1.39
1.37
1.37
1.36
1.35
1.33
1.21
HO
O
Me
O
Me
NH
S
O
tBu
1.00
2.07
0.99
2.98
1.94
1.94
0.96
2.18
1.07
1.10
1.99
3.11
6.08
3.17
6.15
26.93
f1 (ppm)

^{13}C NMR of **27**

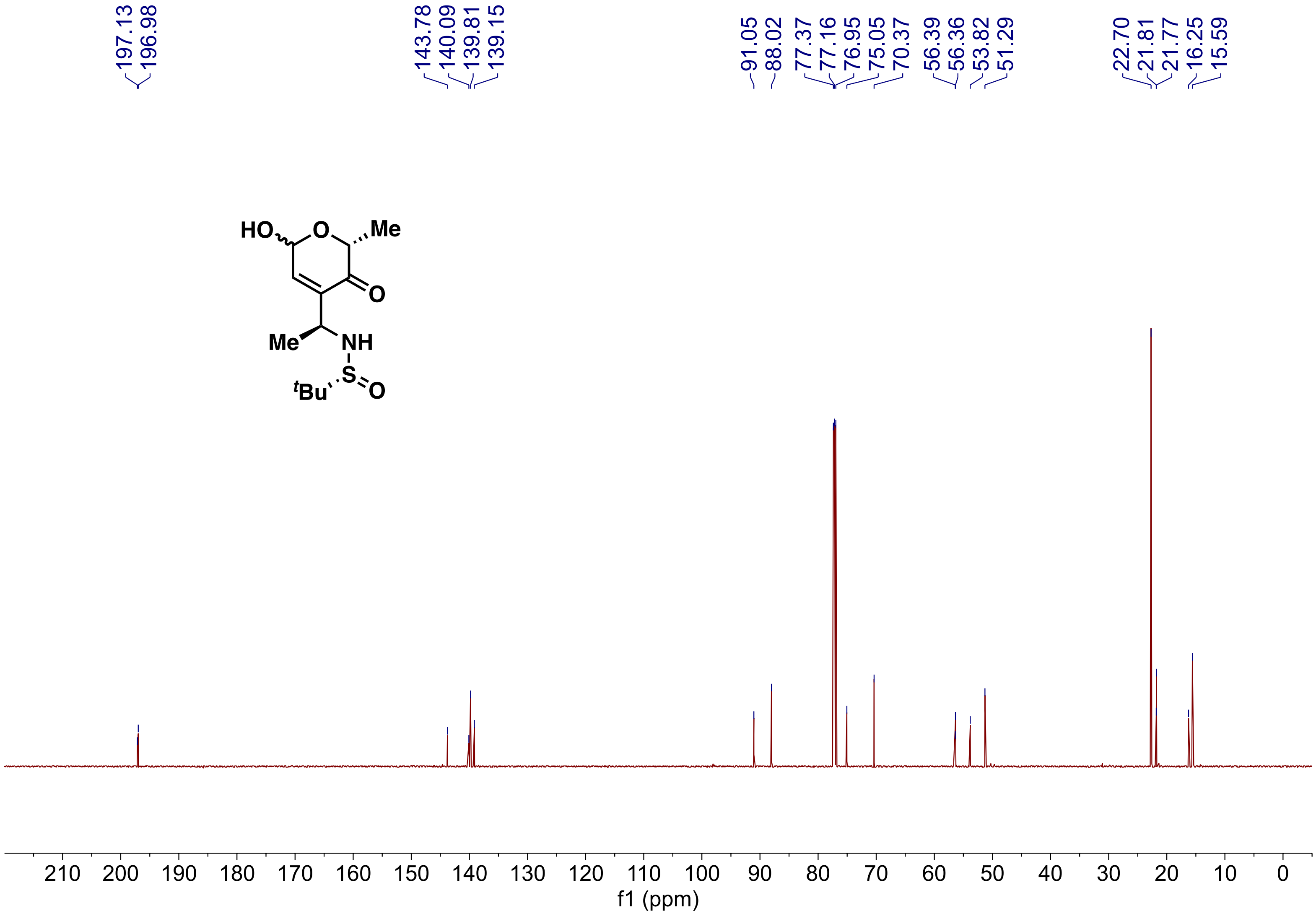
197.13
196.98
143.78
140.09
139.81
139.15
91.05
88.02
77.37
77.16
76.95
75.05
70.37
56.39
56.36
53.82
51.29
22.70
21.81
21.77
16.25
15.59
HO
O
Me
O
Me
NH
S
O
tBu
210 200 190 180 170 160 150 140 130 120 110 100 90 80 70 60 50 40 30 20 10 0
f1 (ppm)

^{1}H NMR of **29**

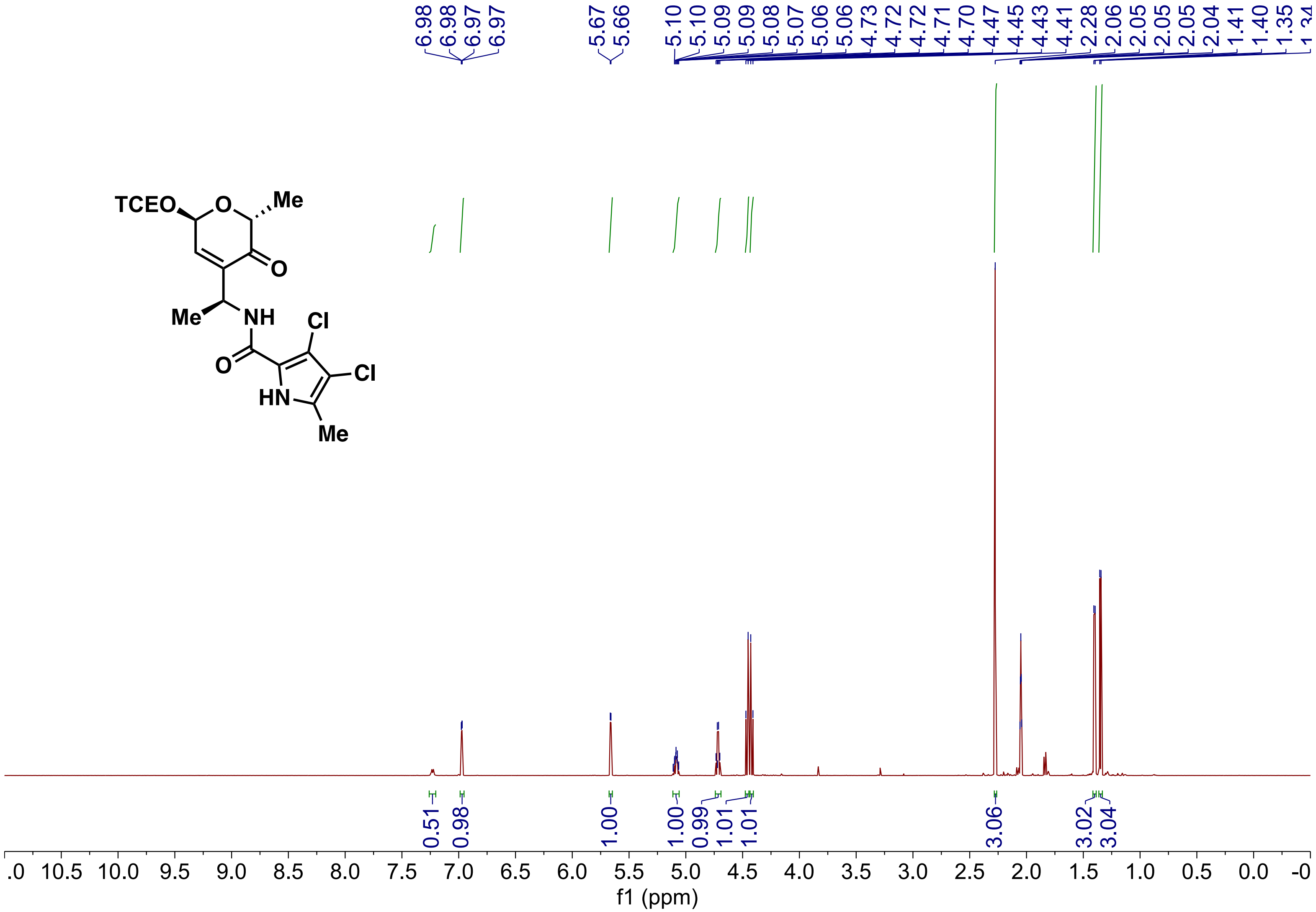

TCEO
O
Me
O
Me
NH
Cl
Cl
O
HN
Me
6.98
6.98
6.97
6.97
5.67
5.66
5.10
5.10
5.09
5.09
5.08
5.07
5.06
5.06
4.73
4.72
4.72
4.71
4.70
4.47
4.45
4.43
4.41
2.28
2.06
2.05
2.05
2.05
2.04
1.41
1.40
1.35
0.51
0.98
1.00
1.00
0.99
1.01
1.01
3.06
3.02
3.04
10.5
10.0
9.5
9.0
8.5
8.0
7.5
7.0
6.5
6.0
5.5
5.0
4.5
4.0
3.5
3.0
2.5
2.0
1.5
1.0
0.5
0.0
f1 (ppm)

^{13}C NMR of **29**

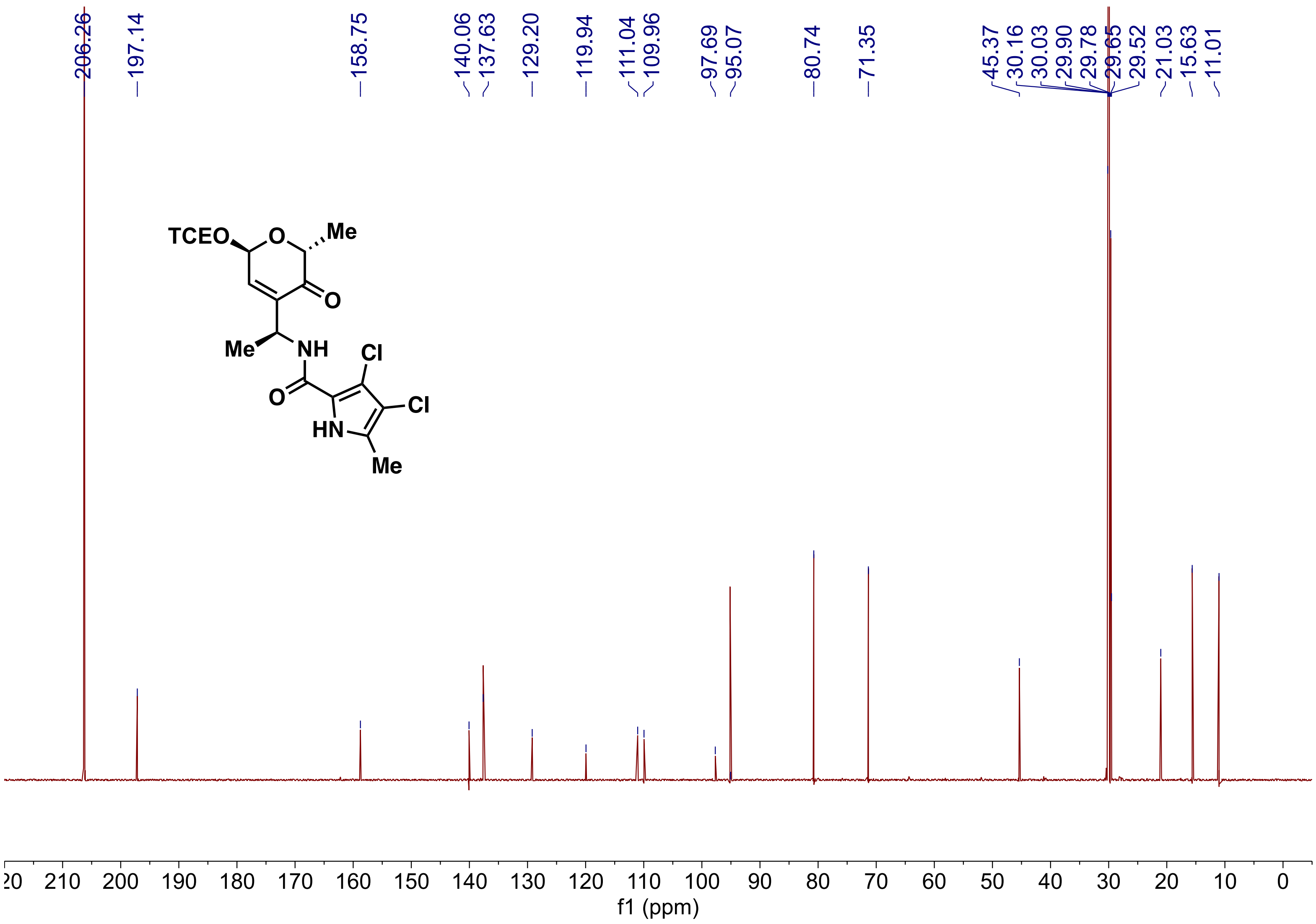

206.26
197.14
158.75
140.06
137.63
129.20
119.94
111.04
109.96
97.69
95.07
80.74
71.35
45.37
30.16
30.03
29.90
29.78
29.65
29.52
21.03
15.63
11.01
TCEO
O
Me
O
Me
NH
Cl
O
Cl
HN
Me
f1 (ppm)

HSQC of **29**

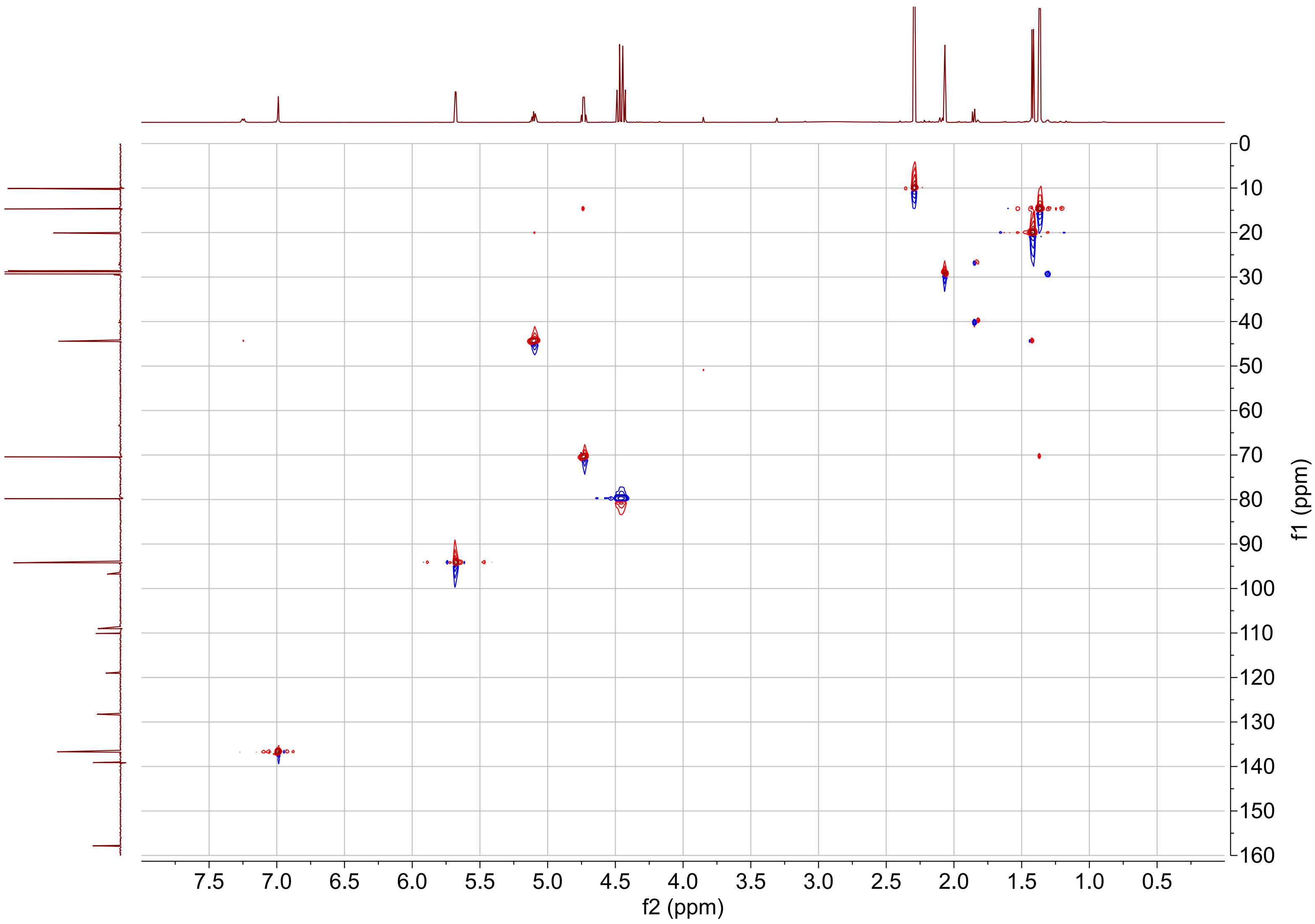

7.5
7.0
6.5
6.0
5.5
5.0
4.5
4.0
3.5
3.0
2.5
2.0
1.5
1.0
0.5
f2 (ppm)
0
10
20
30
40
50
60
70
80
90
100
110
120
130
140
150
160
f1 (ppm)

COSY of **29**

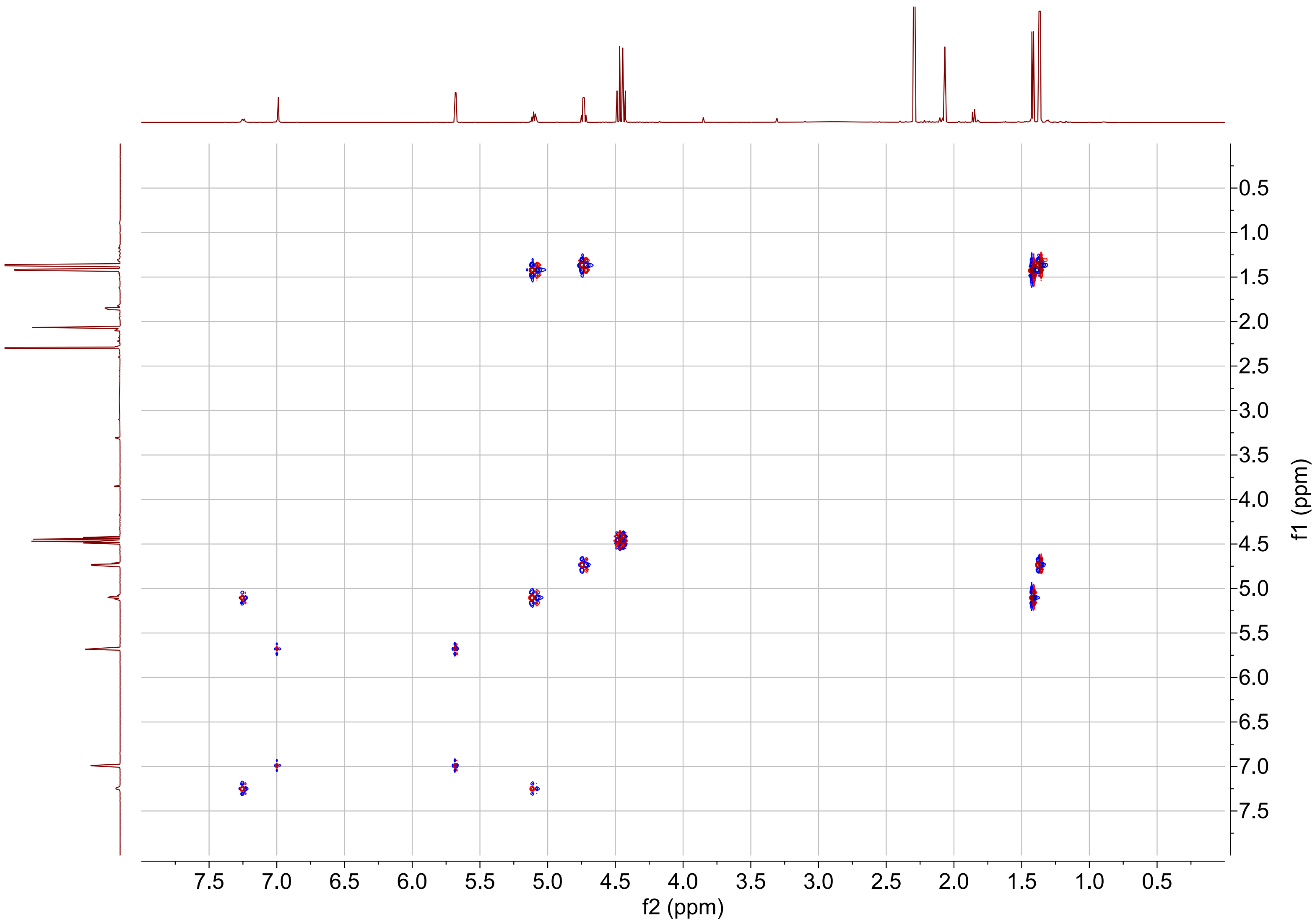

7.5
7.0
6.5
6.0
5.5
5.0
4.5
4.0
3.5
3.0
2.5
2.0
1.5
1.0
0.5
f2 (ppm)
0.5
1.0
1.5
2.0
2.5
3.0
3.5
4.0
4.5
5.0
5.5
6.0
6.5
7.0
7.5
f1 (ppm)

NOESY of **29**

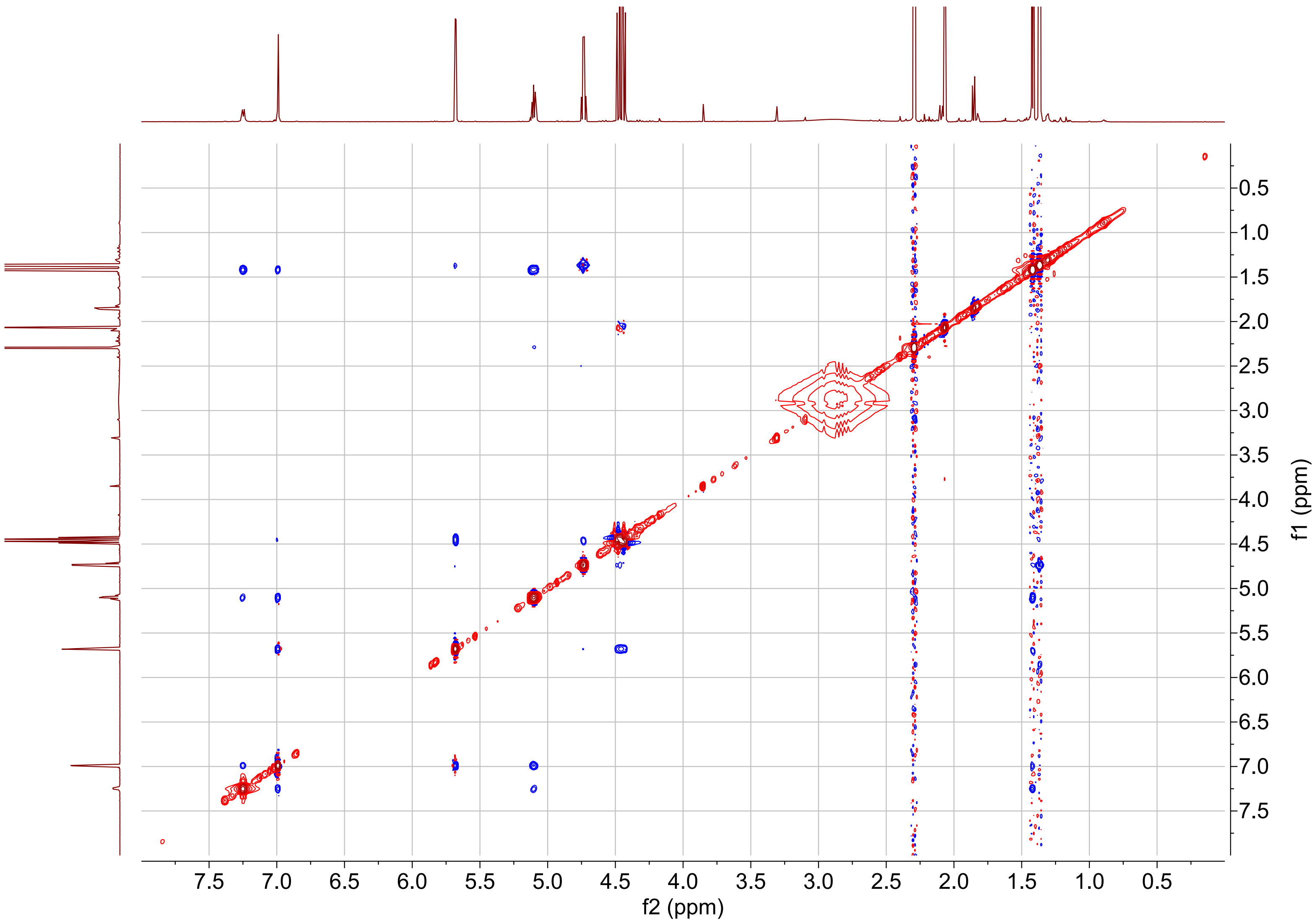

^{1}H NMR of **29B**

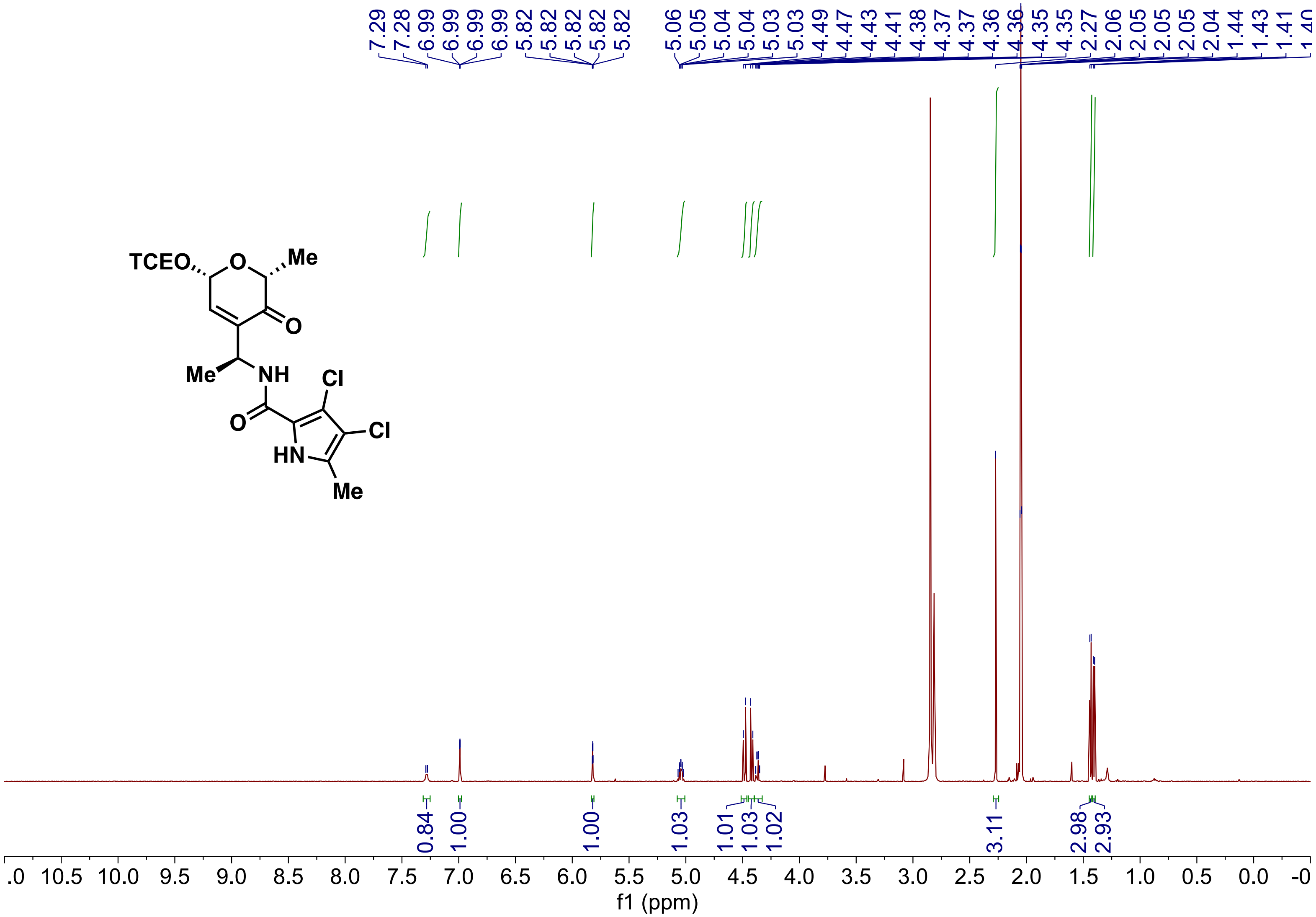

^{13}C NMR of **29B**

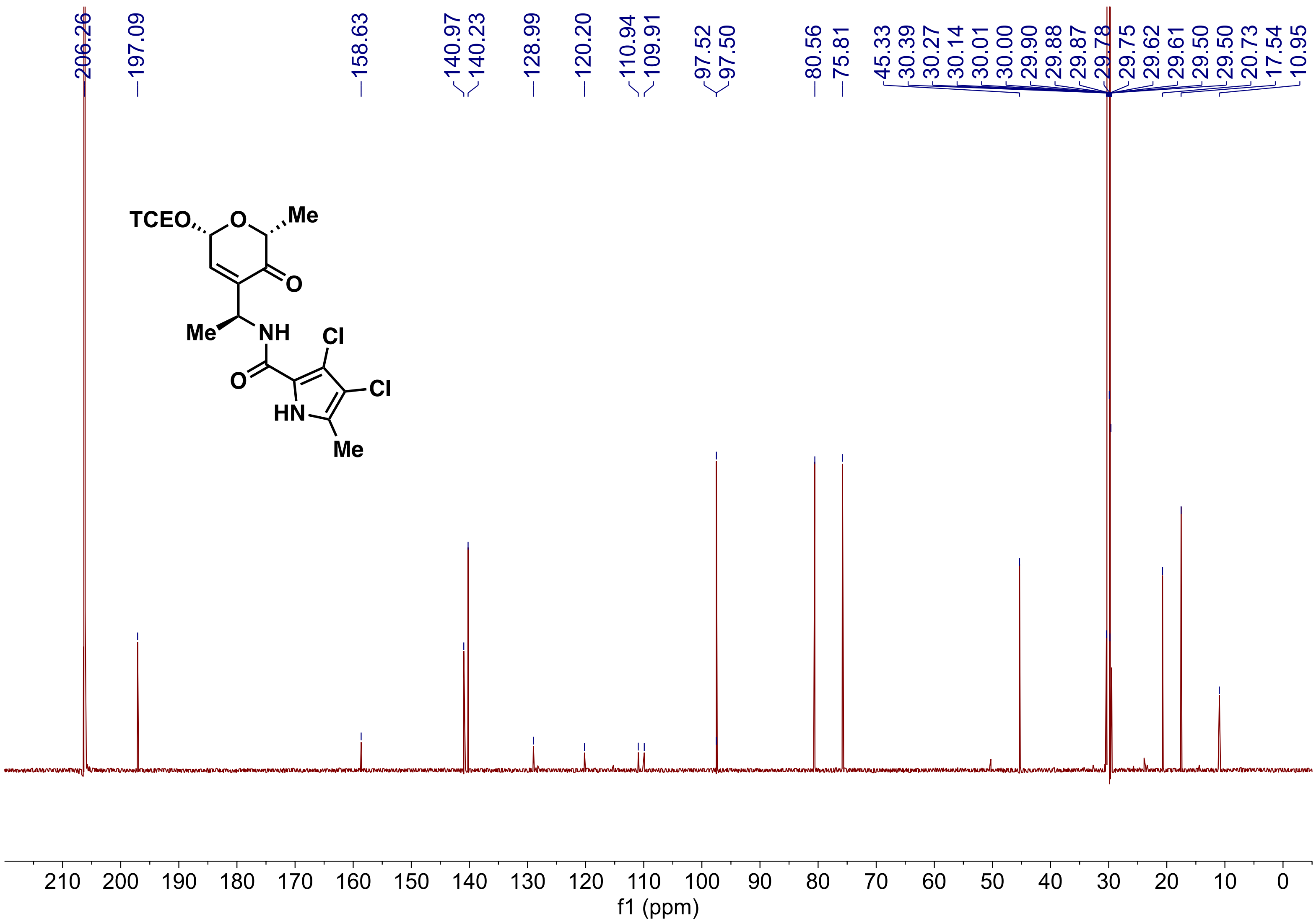

HSQC of **29B**

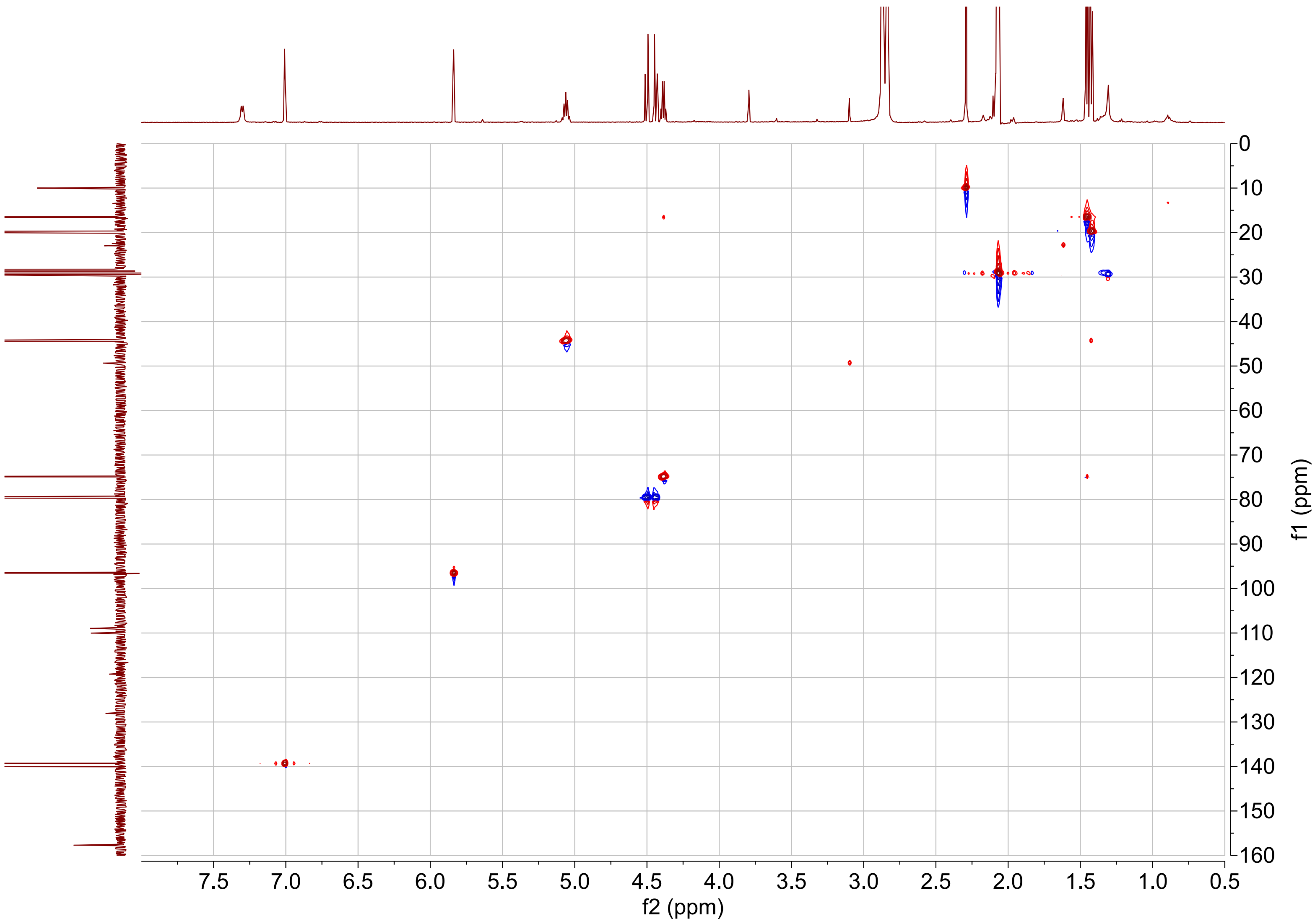

f2 (ppm)
f1 (ppm)
7.5 7.0 6.5 6.0 5.5 5.0 4.5 4.0 3.5 3.0 2.5 2.0 1.5 1.0 0.5
0 10 20 30 40 50 60 70 80 90 100 110 120 130 140 150 160

COSY of **29B**

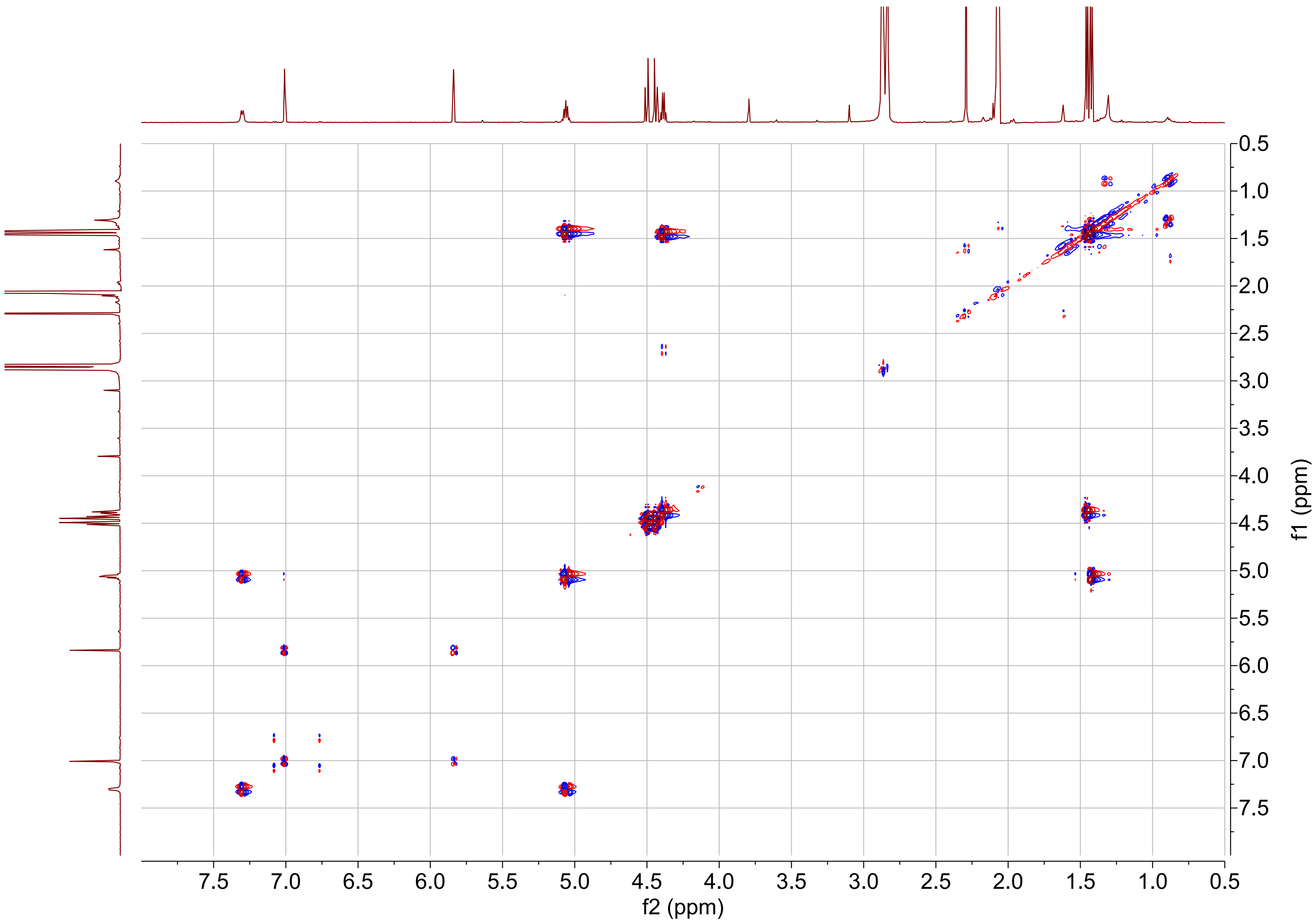

NOESY of **29B**

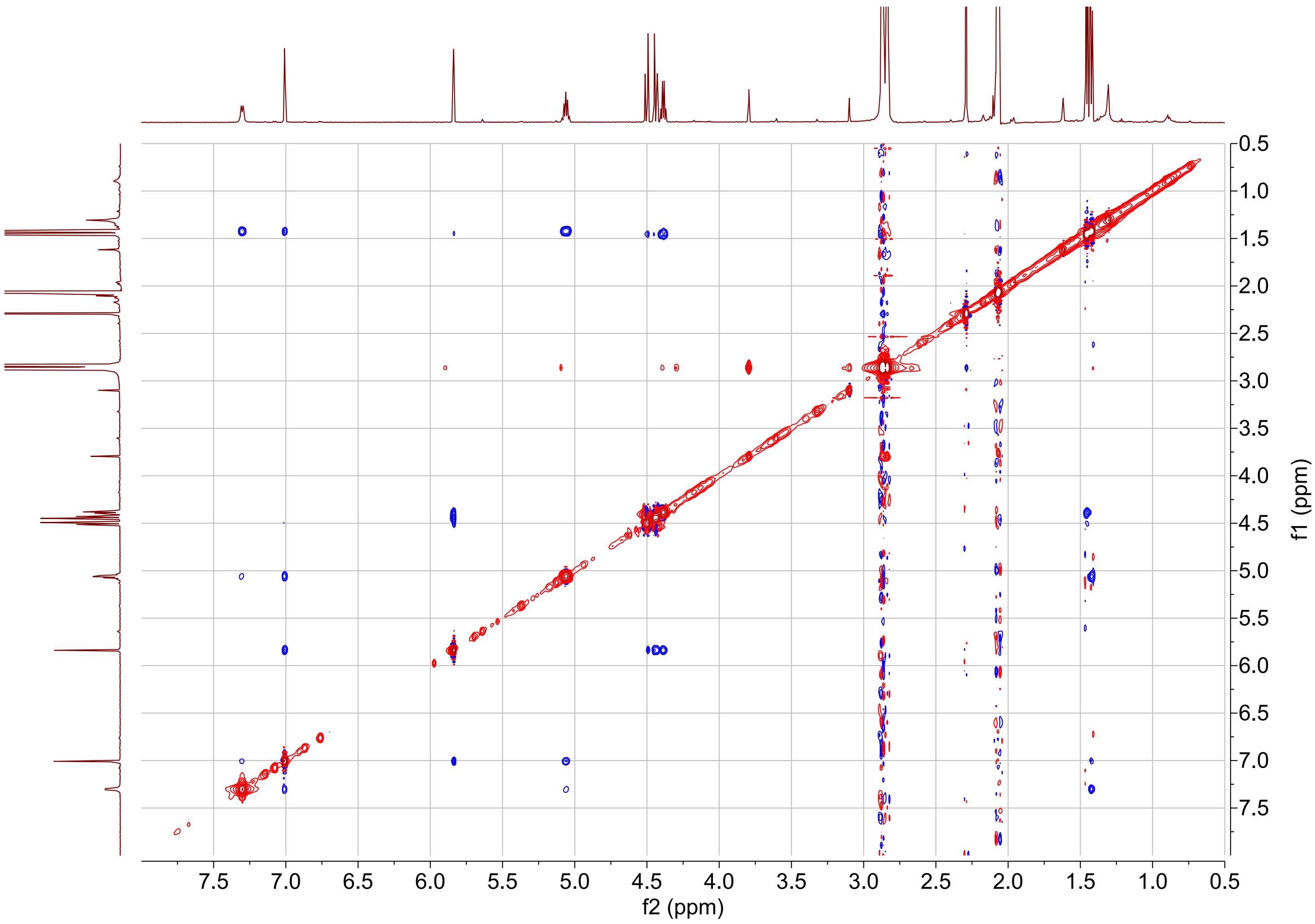

^{1}H NMR of **30**

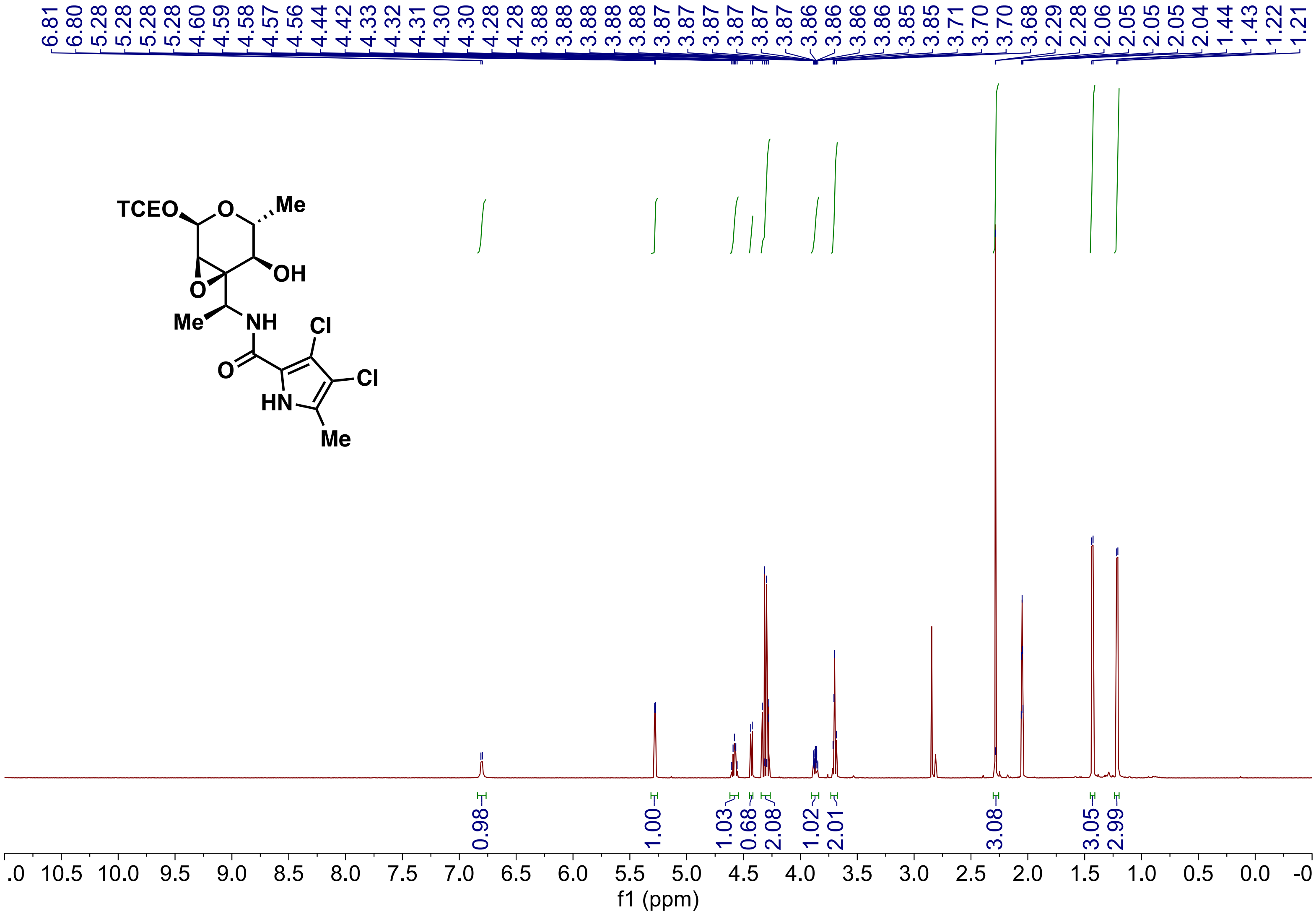
6.81 6.80 5.28 5.28 5.28 5.28 4.60 4.59 4.58 4.57 4.56 4.44 4.42 4.33 4.32 4.31 4.30 4.30 4.28 4.28 3.88 3.88 3.88 3.88 3.88 3.87 3.87 3.87 3.87 3.87 3.87 3.86 3.86 3.86 3.86 3.85 3.85 3.71 3.70 3.70 3.68 2.29 2.28 2.06 2.05 2.05 2.05 2.04 1.44 1.43 1.22 1.21
TCEO
O
Me
OH
O
Me
NH
Cl
Cl
O
HN
Me
0.98
1.00
1.03
0.68
2.08
1.02
2.01
3.08
3.05
2.99
.0 10.5 10.0 9.5 9.0 8.5 8.0 7.5 7.0 6.5 6.0 5.5 5.0 4.5 4.0 3.5 3.0 2.5 2.0 1.5 1.0 0.5 0.0 -0
f1 (ppm)

^{13}C NMR of **30**

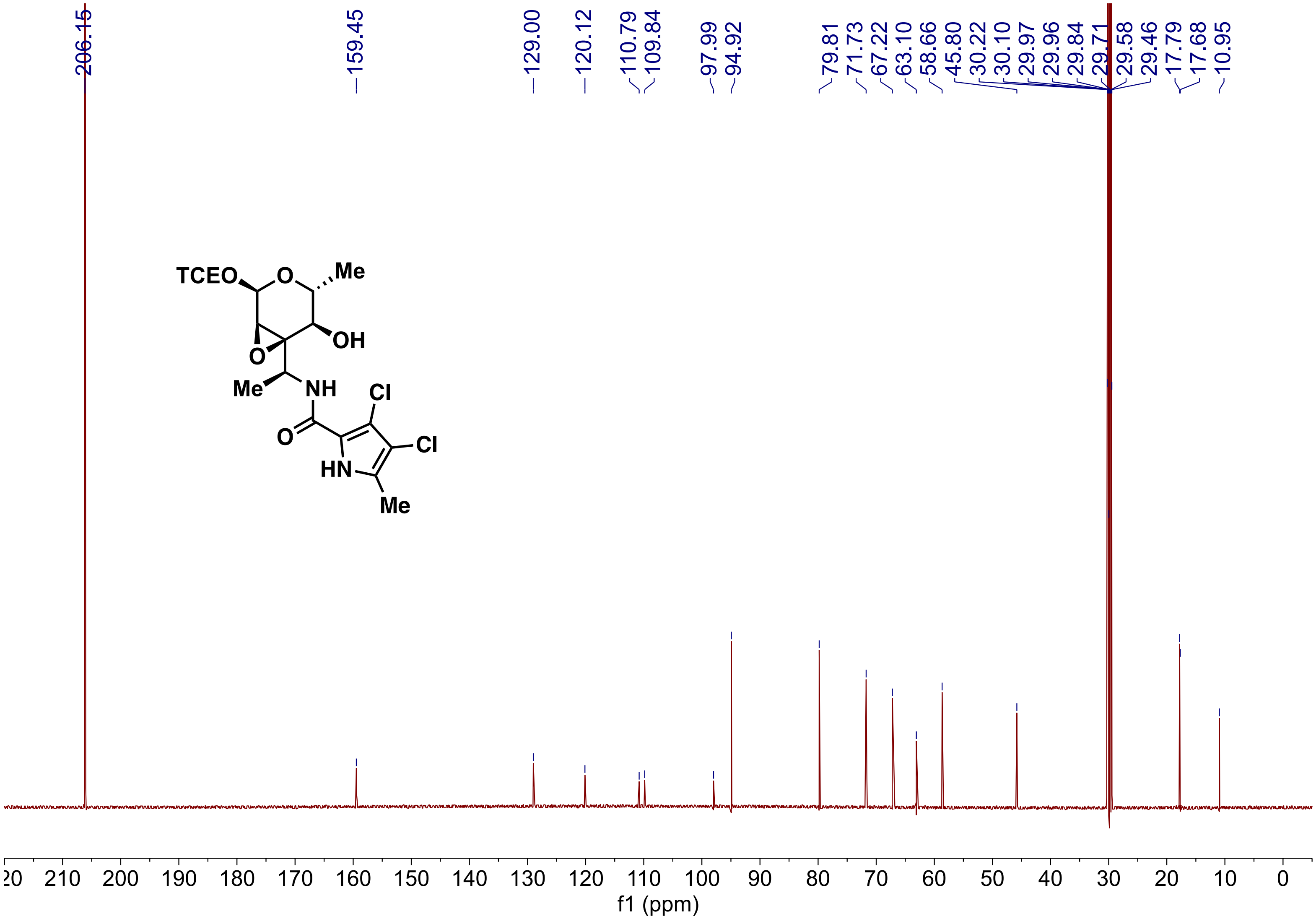
206.15
159.45
129.00
120.12
110.79
109.84
97.99
94.92
79.81
71.73
67.22
63.10
58.66
45.80
30.22
30.10
29.97
29.96
29.84
29.71
29.58
29.46
17.79
17.68
10.95
TCEO
O
Me
OH
O
Me
NH
Cl
Cl
O
HN
Me
20 210 200 190 180 170 160 150 140 130 120 110 100 90 80 70 60 50 40 30 20 10 0
f1 (ppm)

HSQC of **30**

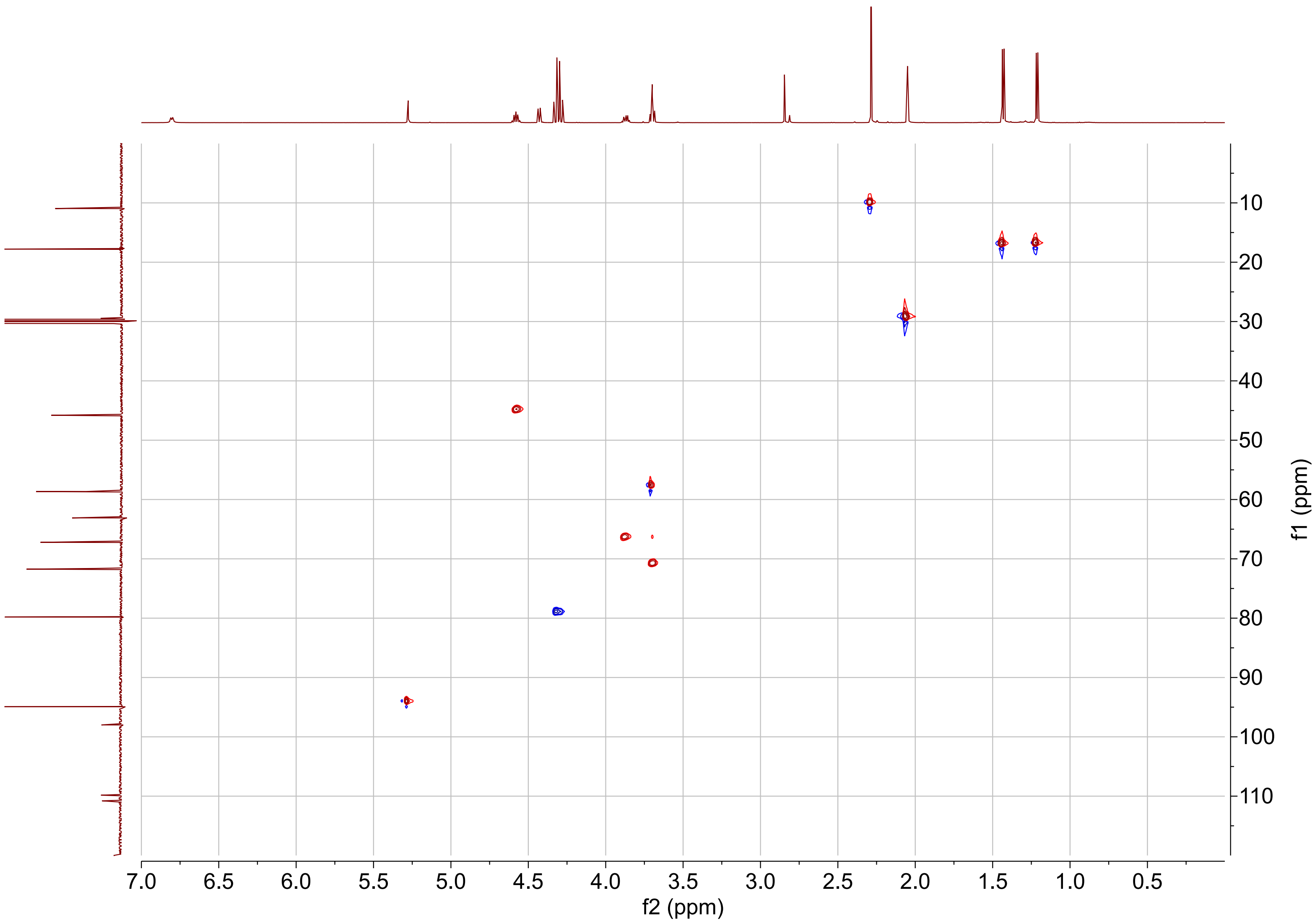

7.0
6.5
6.0
5.5
5.0
4.5
4.0
3.5
3.0
2.5
2.0
1.5
1.0
0.5
f2 (ppm)
10
20
30
40
50
60
70
80
90
100
110
f1 (ppm)

^{1}H NMR of **S9**

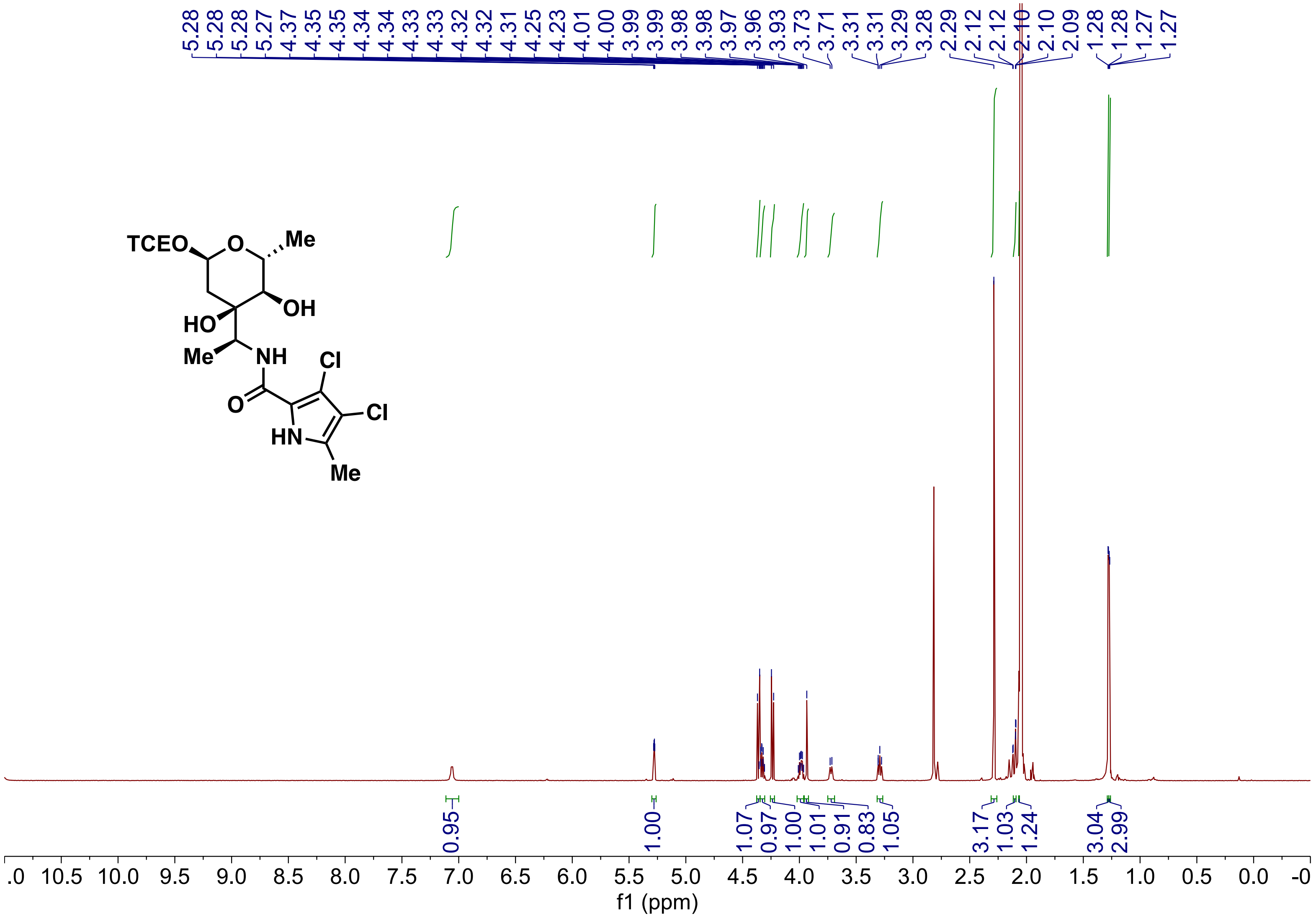
5.28 5.28 5.28 5.27 4.37 4.35 4.35 4.34 4.34 4.33 4.33 4.32 4.32 4.31 4.25 4.23 4.01 4.00 3.99 3.99 3.98 3.98 3.97 3.96 3.93 3.73 3.71 3.31 3.31 3.29 3.28 2.29 2.12 2.12 2.10 2.10 2.09 1.28 1.28 1.27 1.27
TCEO
O
Me
OH
HO
Me
NH
Cl
Cl
O
HN
Me
0.95
1.00
1.07 0.97 1.00 1.01 0.91 0.83 1.05
3.17 1.03 1.24
3.04 2.99
.0 10.5 10.0 9.5 9.0 8.5 8.0 7.5 7.0 6.5 6.0 5.5 5.0 4.5 4.0 3.5 3.0 2.5 2.0 1.5 1.0 0.5 0.0 -0
f1 (ppm)

^{13}C NMR of **S9**

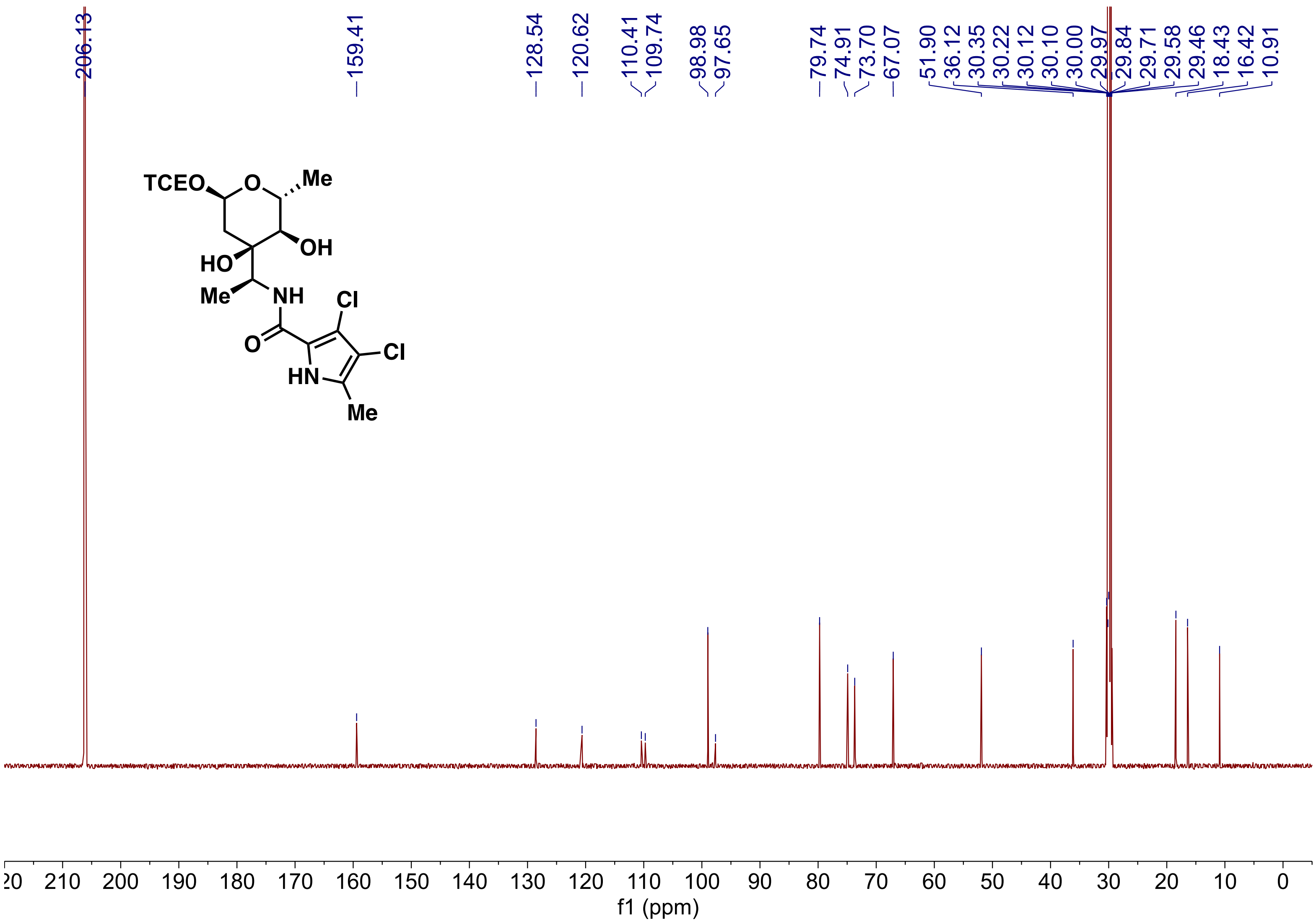
206.13
159.41
128.54
120.62
110.41
109.74
98.98
97.65
79.74
74.91
73.70
67.07
51.90
36.12
30.35
30.22
30.12
30.10
30.00
29.97
29.84
29.71
29.58
29.46
18.43
16.42
10.91
TCEO
O
Me
OH
HO
Me
NH
Cl
O
Cl
HN
Me
20 210 200 190 180 170 160 150 140 130 120 110 100 90 80 70 60 50 40 30 20 10 0
f1 (ppm)

HSQC of **S9**

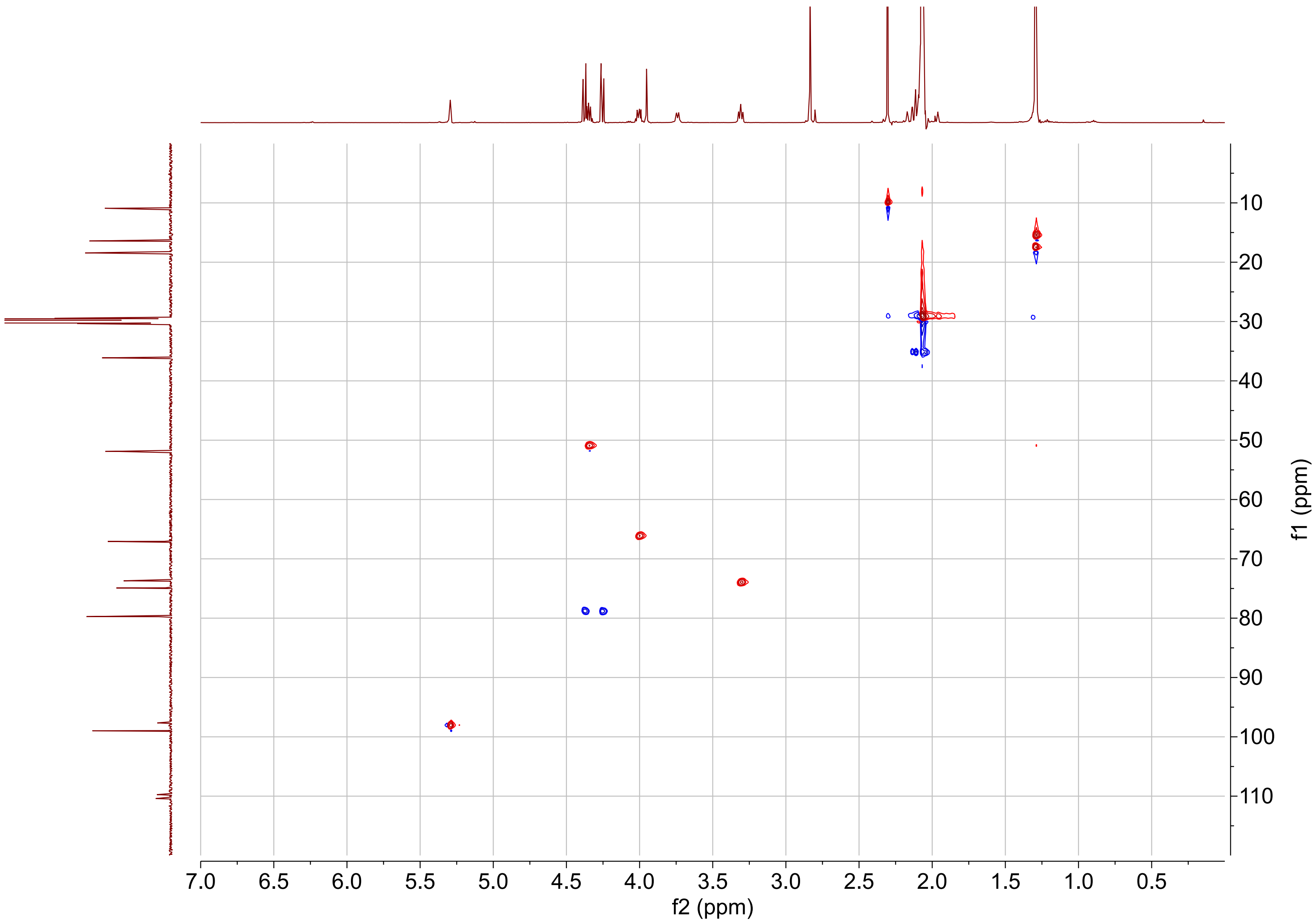

7.0
6.5
6.0
5.5
5.0
4.5
4.0
3.5
3.0
2.5
2.0
1.5
1.0
0.5
f2 (ppm)
10
20
30
40
50
60
70
80
90
100
110
f1 (ppm)

COSY of **S9**

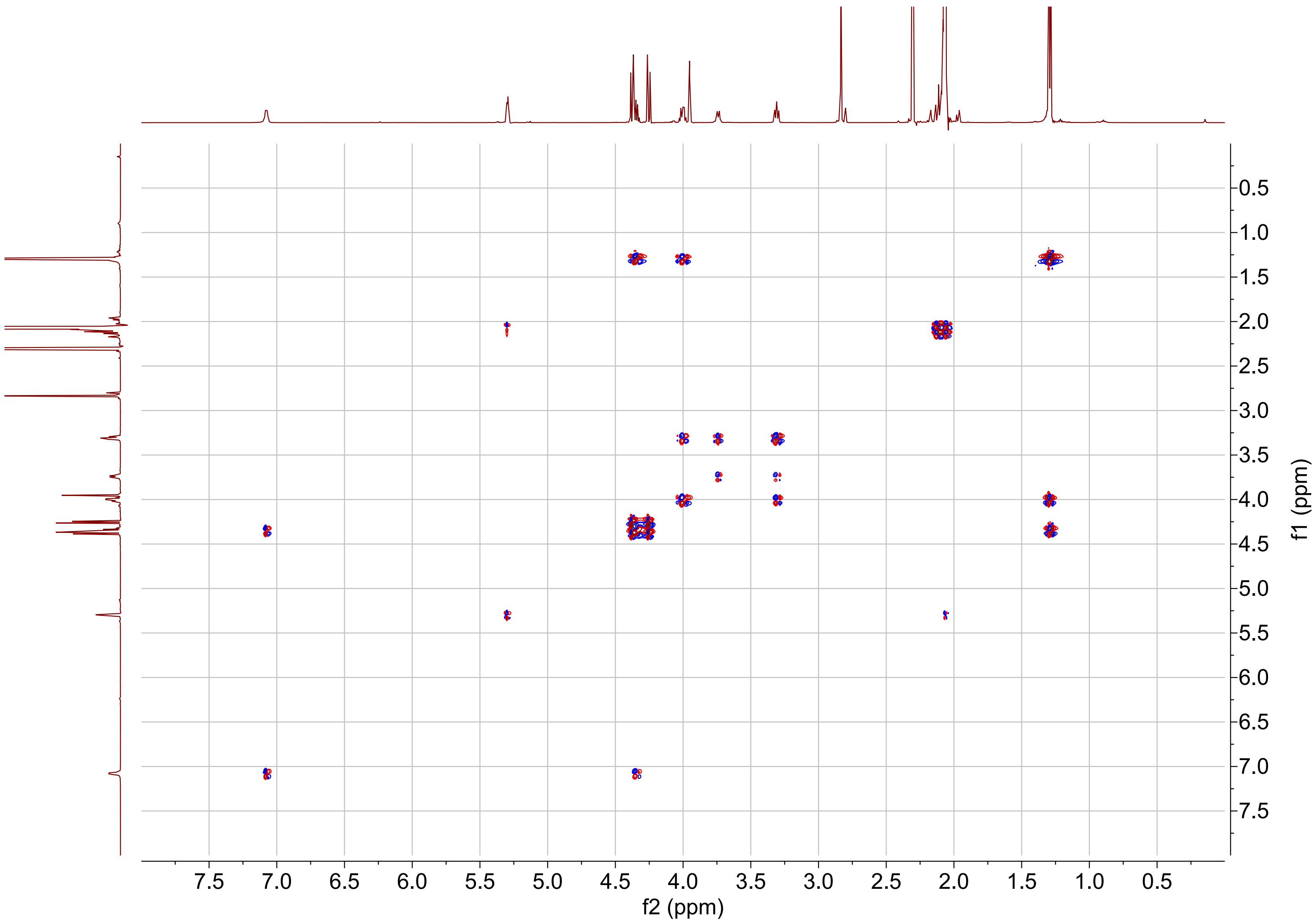

^{1}H NMR of **31**

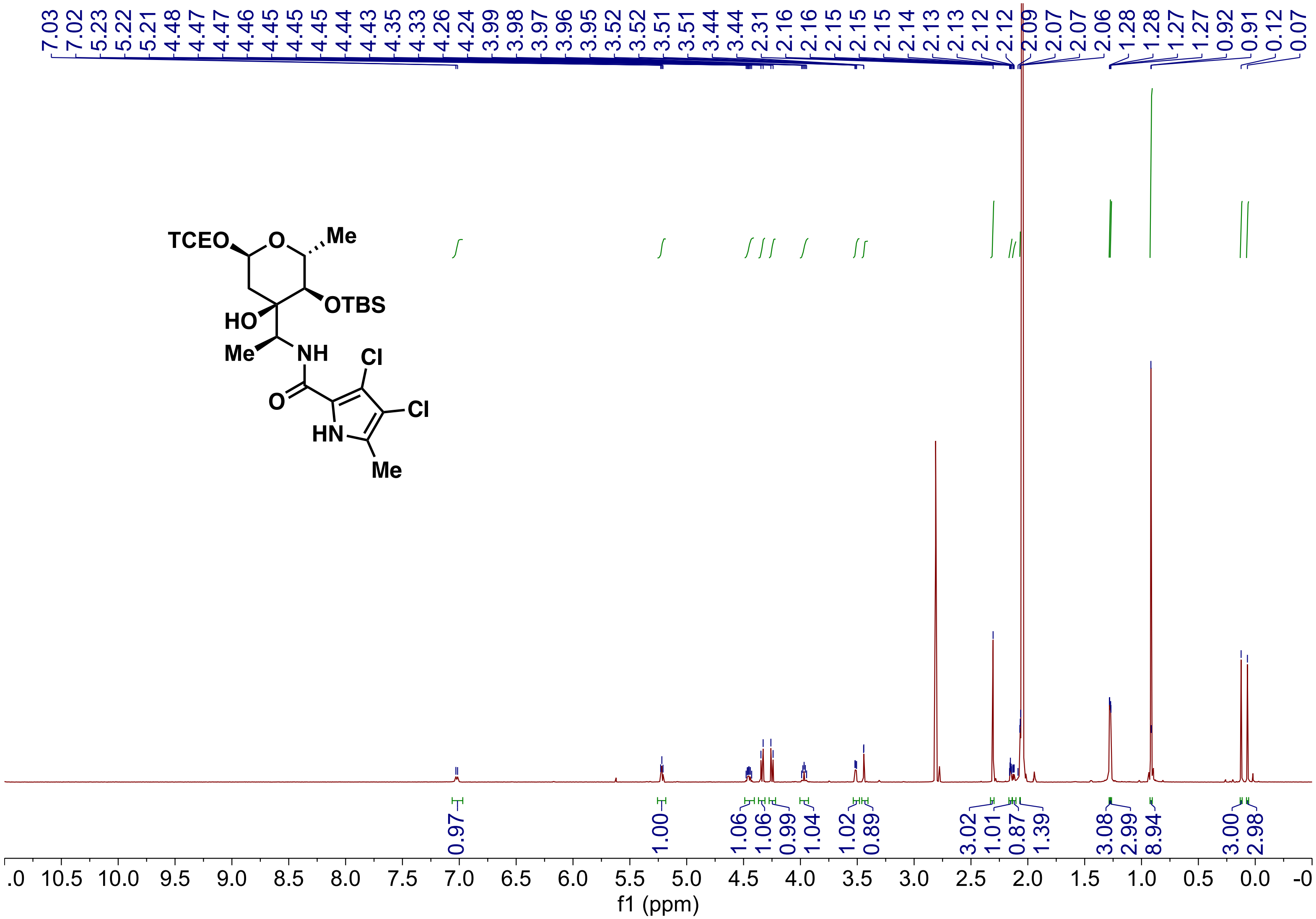
7.03
7.02
5.23
5.22
5.21
4.48
4.47
4.47
4.46
4.46
4.45
4.45
4.45
4.44
4.43
4.35
4.33
4.26
4.24
3.99
3.98
3.97
3.96
3.95
3.52
3.52
3.51
3.51
3.44
3.44
2.31
2.16
2.16
2.15
2.15
2.15
2.14
2.13
2.13
2.12
2.12
2.09
2.07
2.07
2.06
1.28
1.28
1.27
1.27
0.92
0.91
0.12
0.07
0.97
1.00
1.06
1.06
0.99
1.04
1.02
0.89
3.02
1.01
0.87
1.39
3.08
2.99
8.94
3.00
2.98
f1 (ppm)

^{13}C NMR of **31**

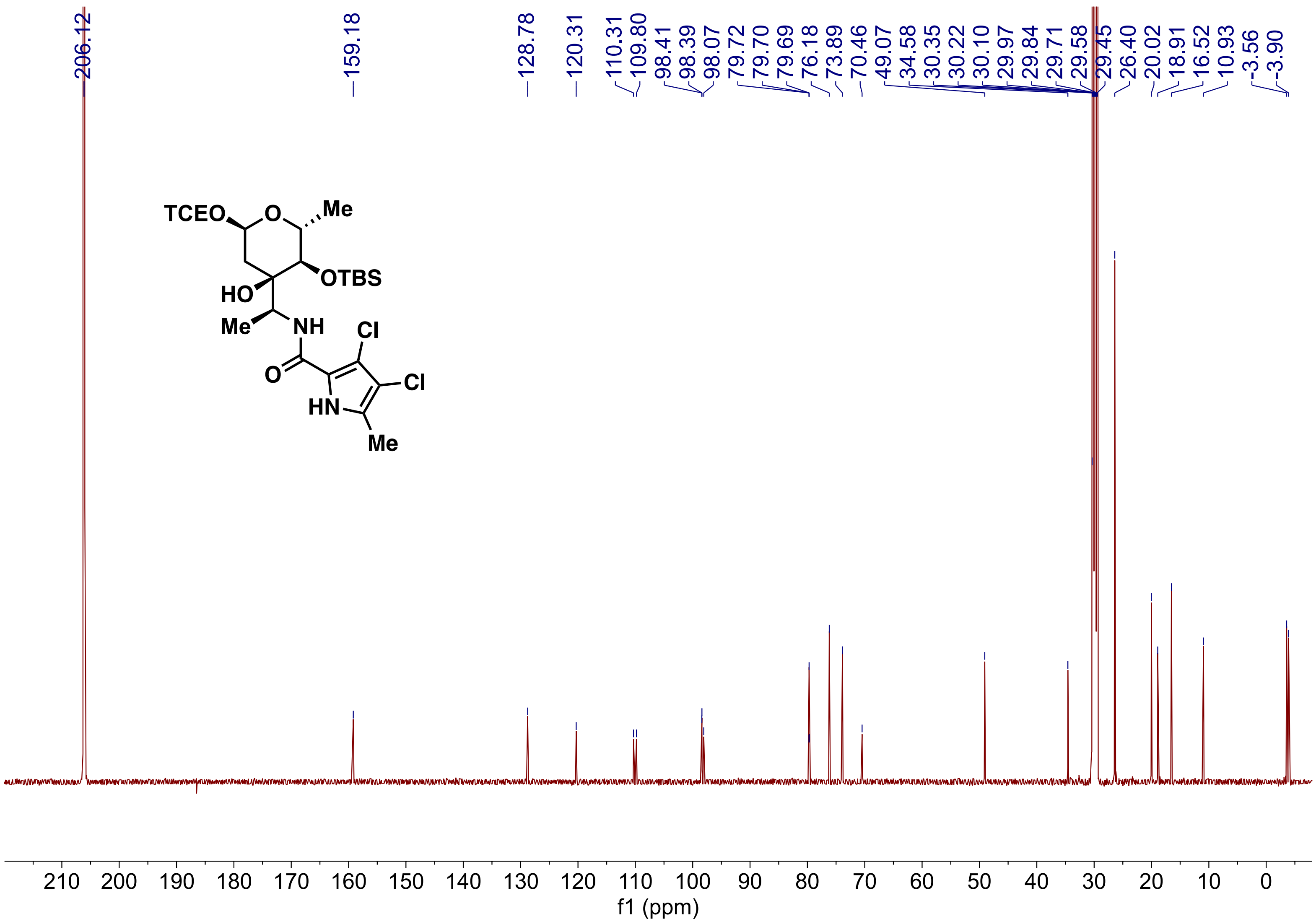
206.12
159.18
128.78
120.31
110.31
109.80
98.41
98.39
98.07
79.72
79.70
79.69
76.18
73.89
70.46
49.07
34.58
30.35
30.22
30.10
29.97
29.84
29.71
29.58
29.45
26.40
20.02
18.91
16.52
10.93
-3.56
-3.90
TCEO
O
Me
OTBS
HO
Me
NH
Cl
O
Cl
HN
Me
210 200 190 180 170 160 150 140 130 120 110 100 90 80 70 60 50 40 30 20 10 0
f1 (ppm)

HSQC of **31**

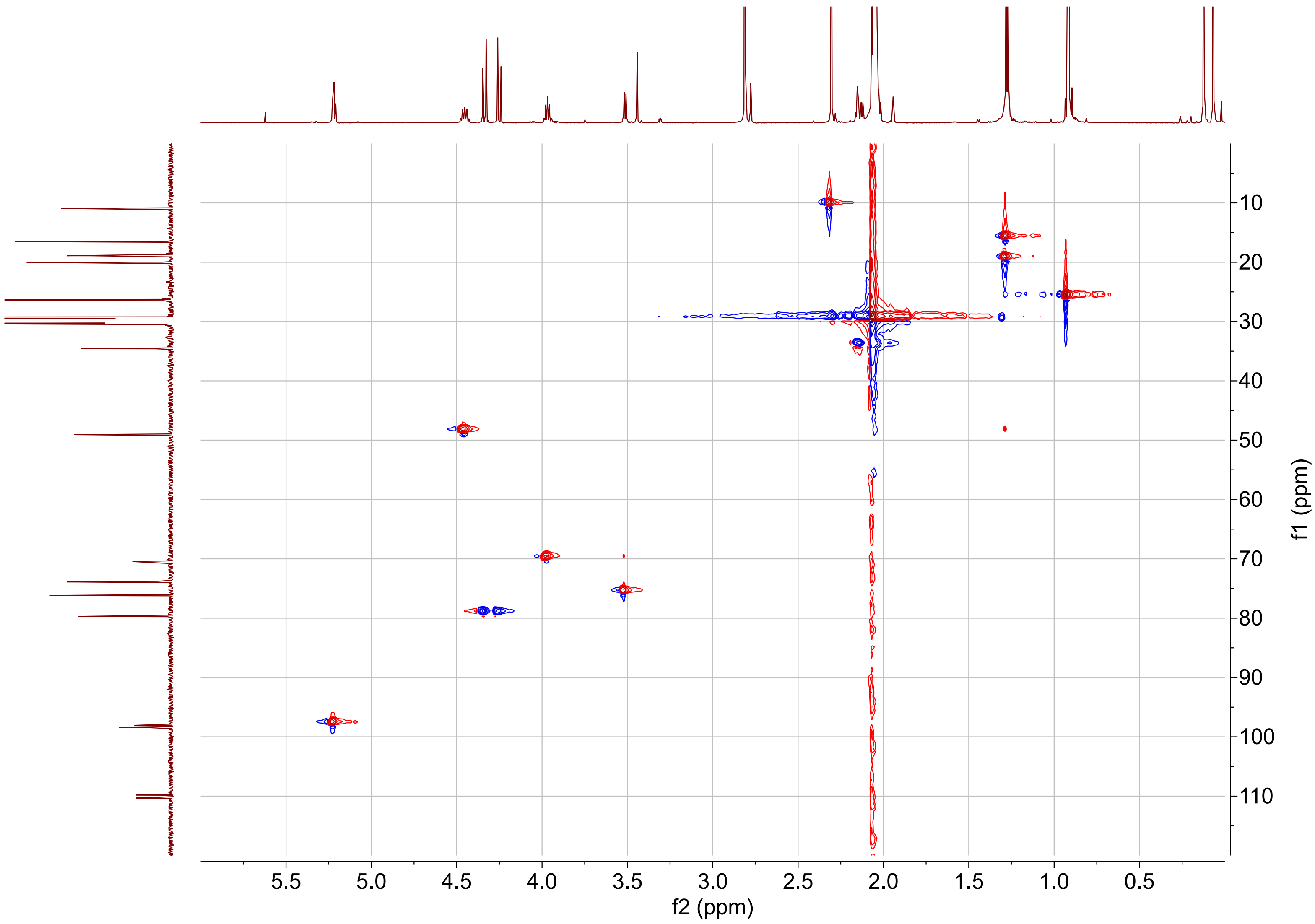

5.5
5.0
4.5
4.0
3.5
3.0
2.5
2.0
1.5
1.0
0.5
f2 (ppm)
10
20
30
40
50
60
70
80
90
100
110
f1 (ppm)

^{1}H NMR of **32**

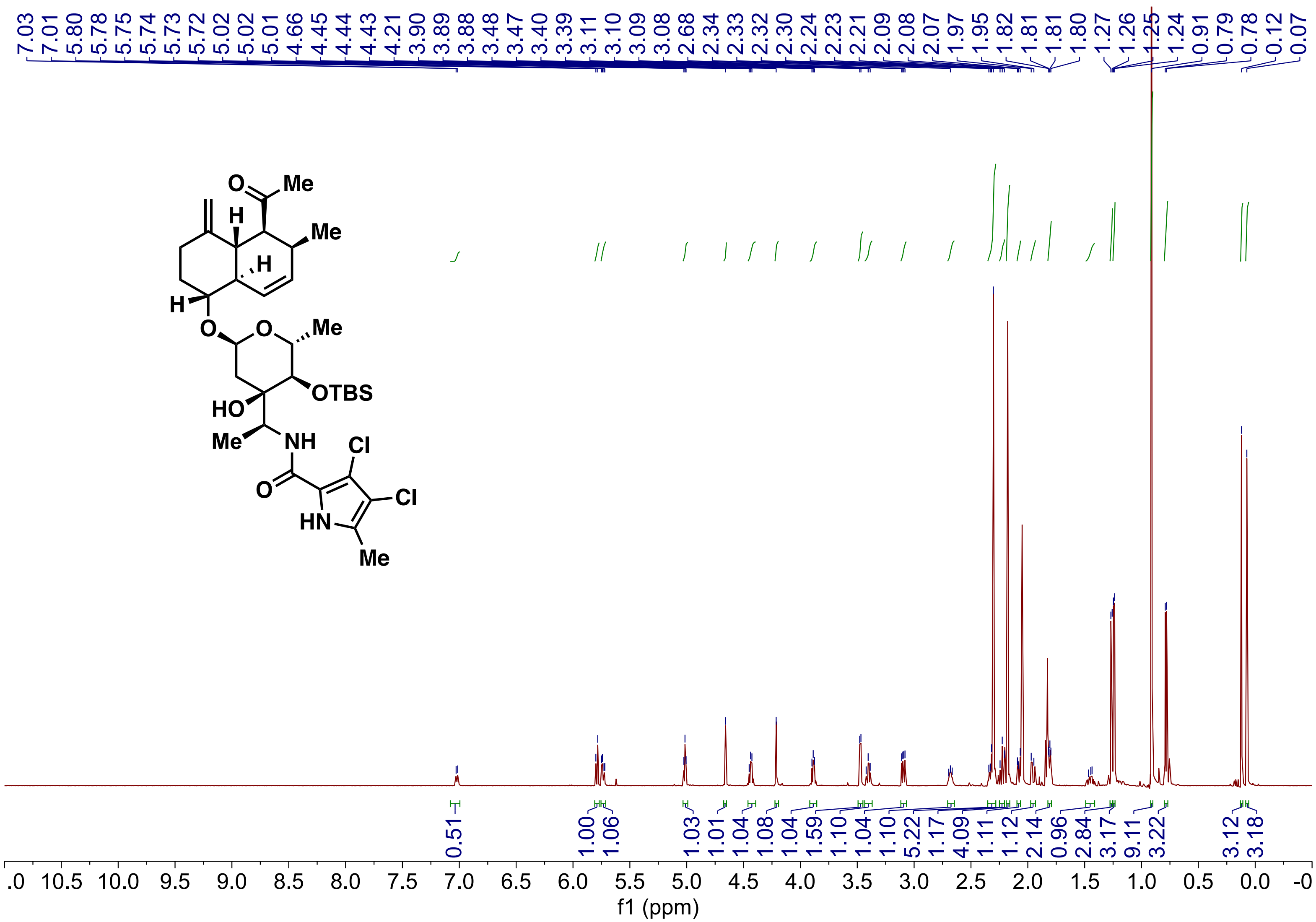

7.03
7.01
5.80
5.78
5.75
5.74
5.73
5.72
5.02
5.02
5.01
4.66
4.45
4.44
4.43
4.21
3.90
3.89
3.88
3.48
3.47
3.40
3.39
3.11
3.10
3.09
3.08
2.68
2.34
2.33
2.32
2.30
2.24
2.23
2.21
2.09
2.08
2.07
1.97
1.95
1.82
1.81
1.81
1.80
1.27
1.26
1.25
1.24
0.91
0.79
0.78
0.12
0.07
O
Me
H
Me
H
H
O
O
Me
OTBS
HO
Me
NH
Cl
O
Cl
HN
Me
0.51
1.00
1.06
1.03
1.01
1.04
1.08
1.04
1.59
1.10
1.04
1.10
5.22
1.17
4.09
1.11
1.12
2.14
0.96
2.84
3.17
9.11
3.22
3.12
3.18
f1 (ppm)

^{13}C NMR of **32**

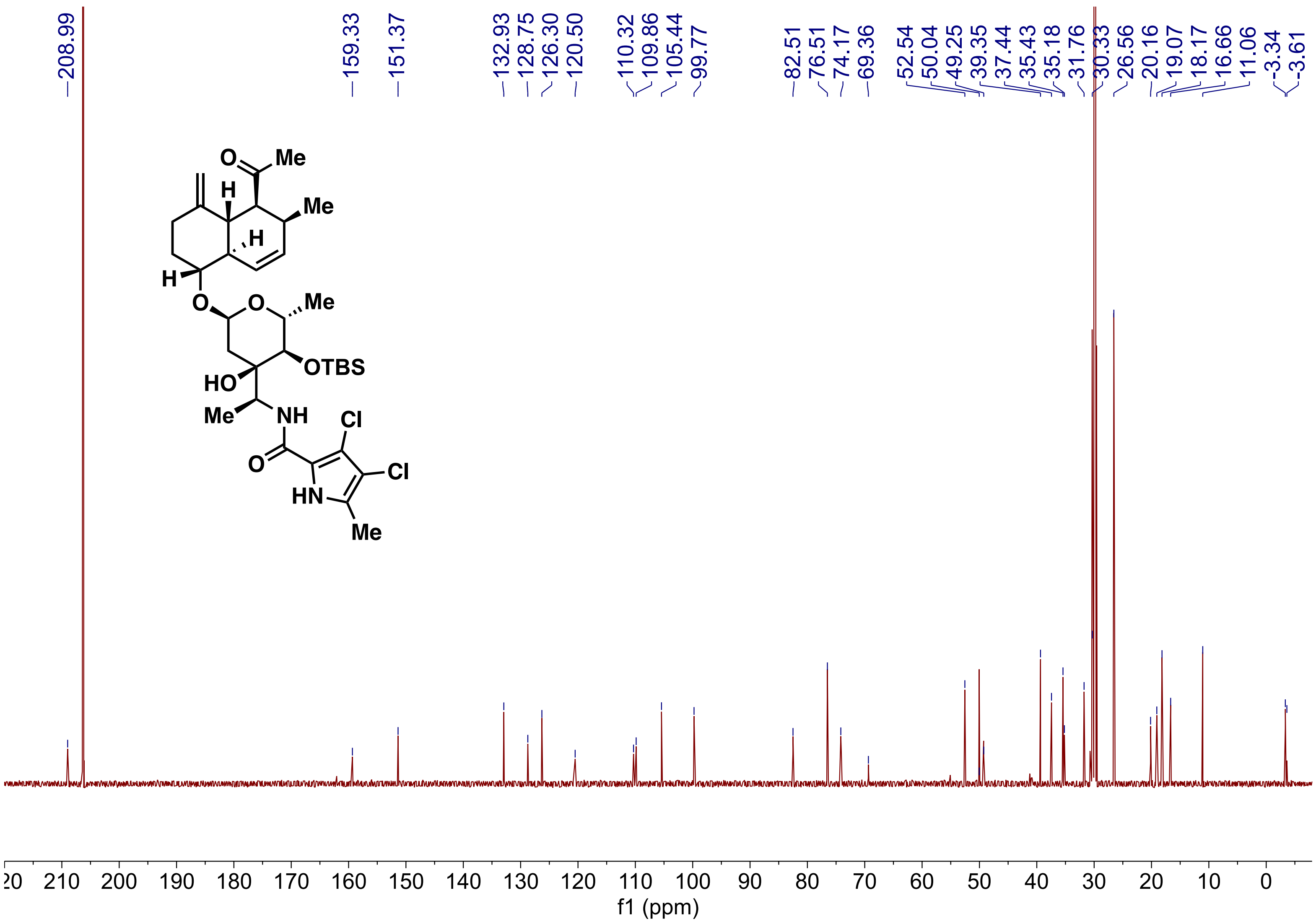
208.99
159.33
151.37
132.93
128.75
126.30
120.50
110.32
109.86
105.44
99.77
82.51
76.51
74.17
69.36
52.54
50.04
49.25
39.35
37.44
35.43
35.18
31.76
30.33
26.56
20.16
19.07
18.17
16.66
11.06
-3.34
-3.61
O
Me
H
Me
H
H
O
O
Me
OTBS
HO
Me
NH
Cl
O
Cl
HN
Me
20 210 200 190 180 170 160 150 140 130 120 110 100 90 80 70 60 50 40 30 20 10 0
f1 (ppm)

HSQC of **32**

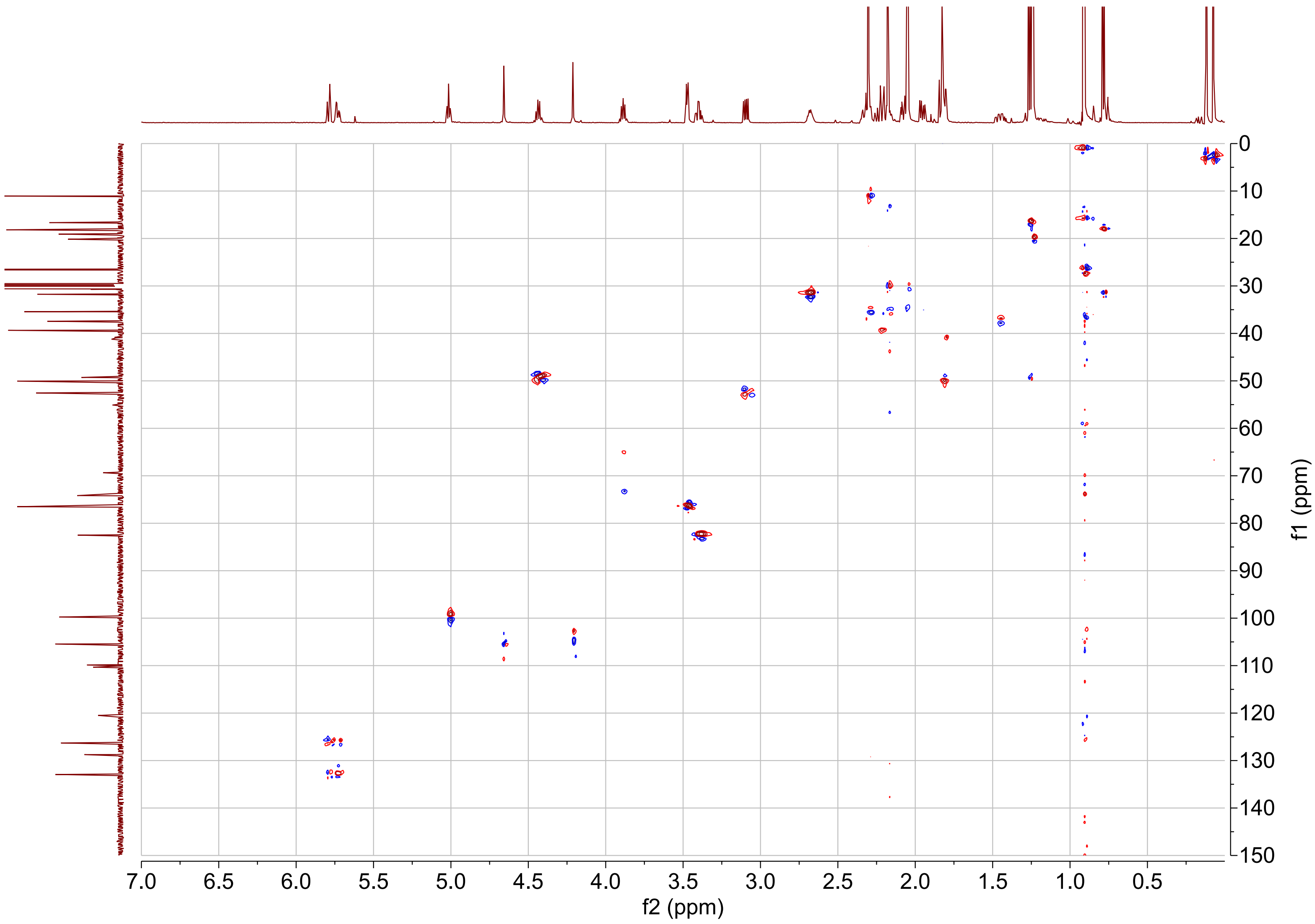

f2 (ppm)
f1 (ppm)
7.0
6.5
6.0
5.5
5.0
4.5
4.0
3.5
3.0
2.5
2.0
1.5
1.0
0.5
0
10
20
30
40
50
60
70
80
90
100
110
120
130
140
150

COSY of **32**

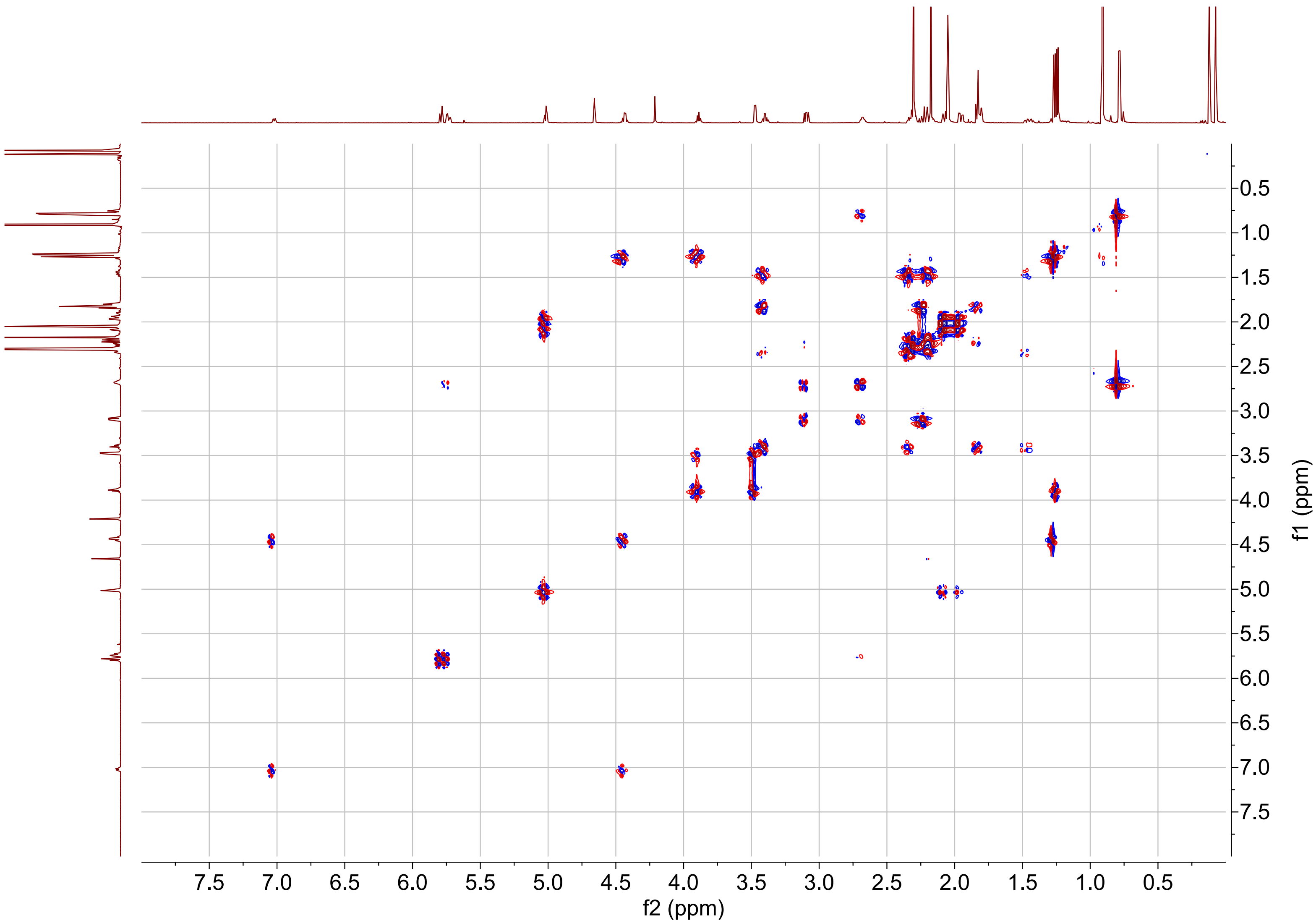

NOESY of **32**

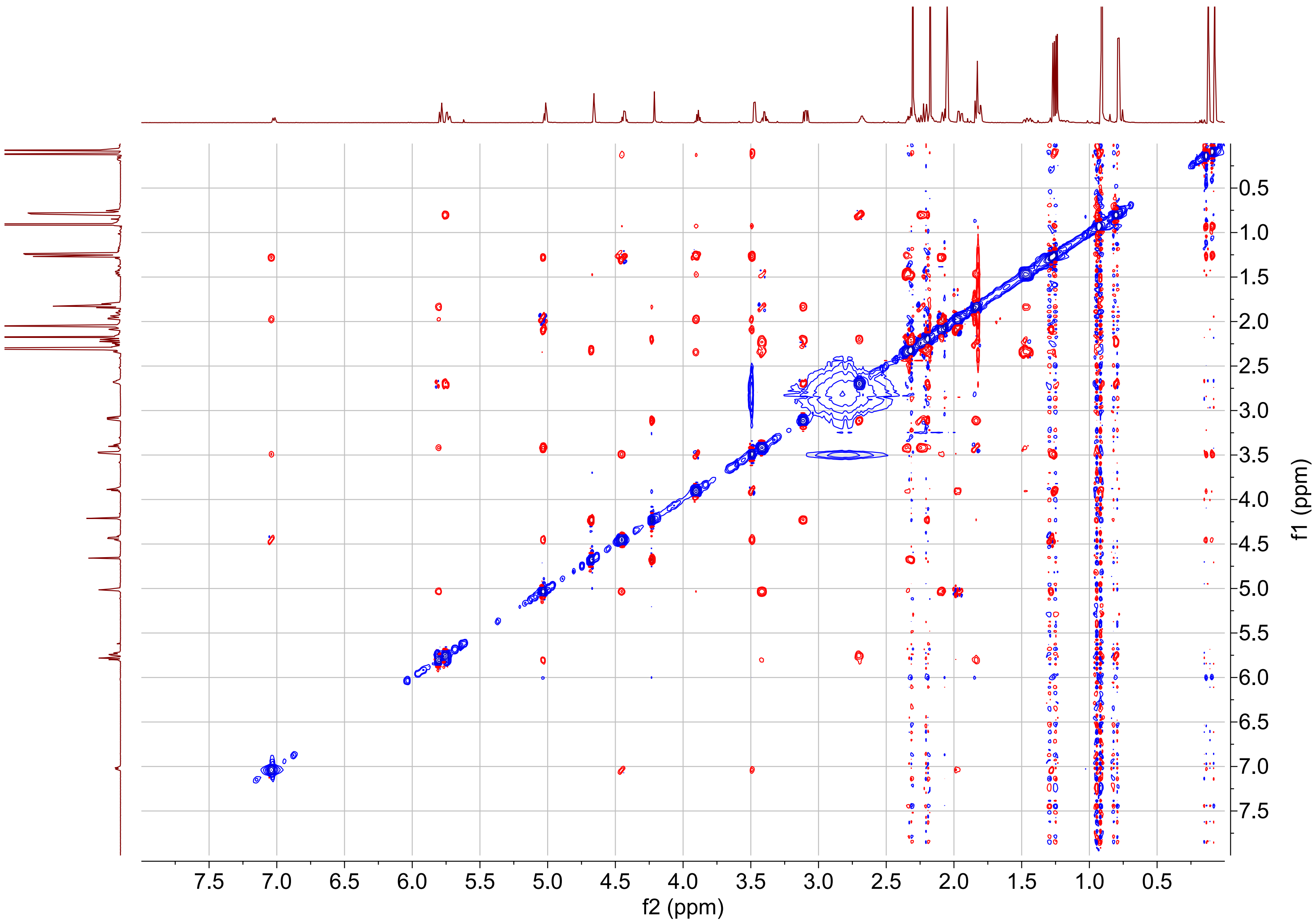

^{1}H NMR of **33**

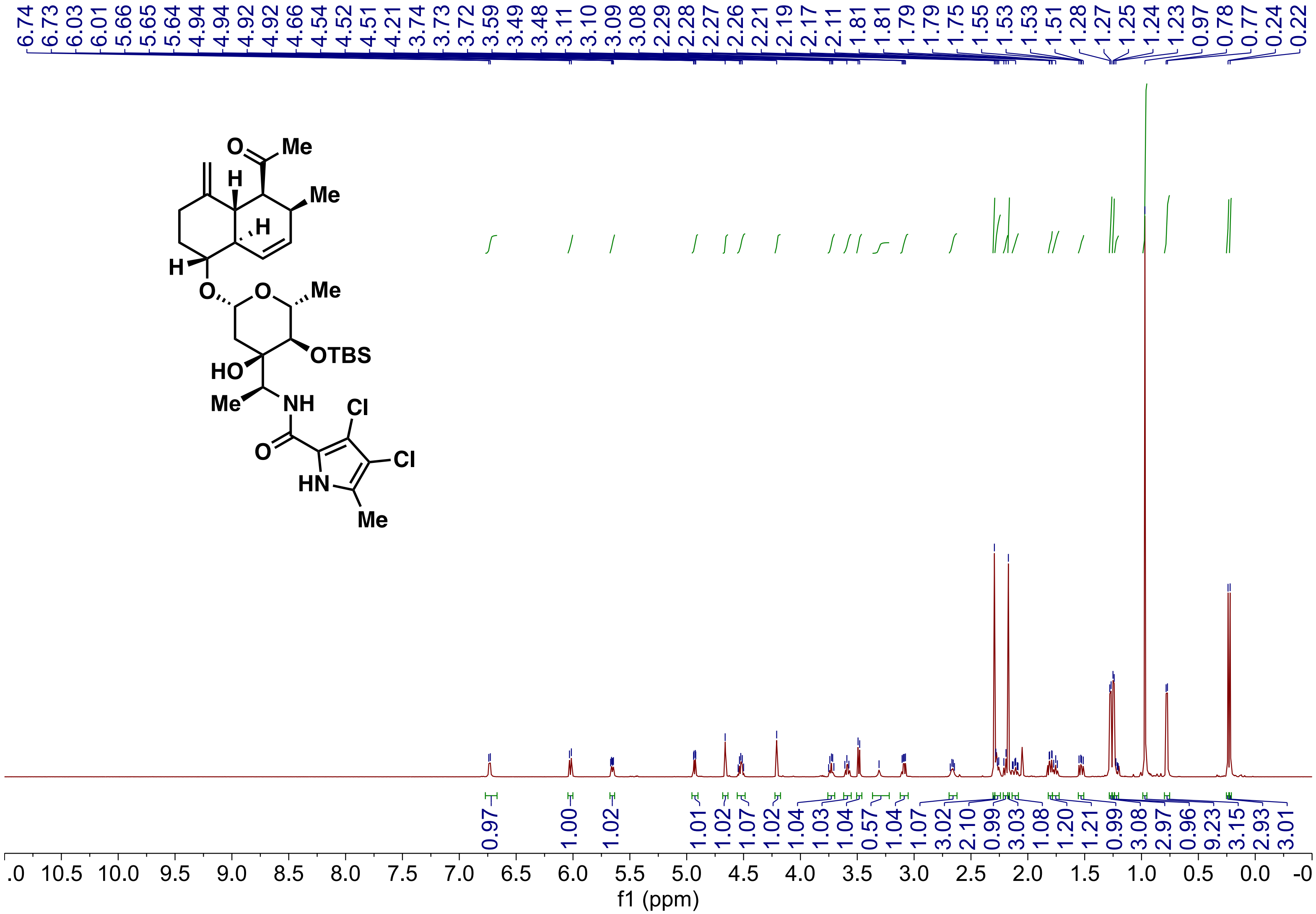

^{13}C NMR of **33**

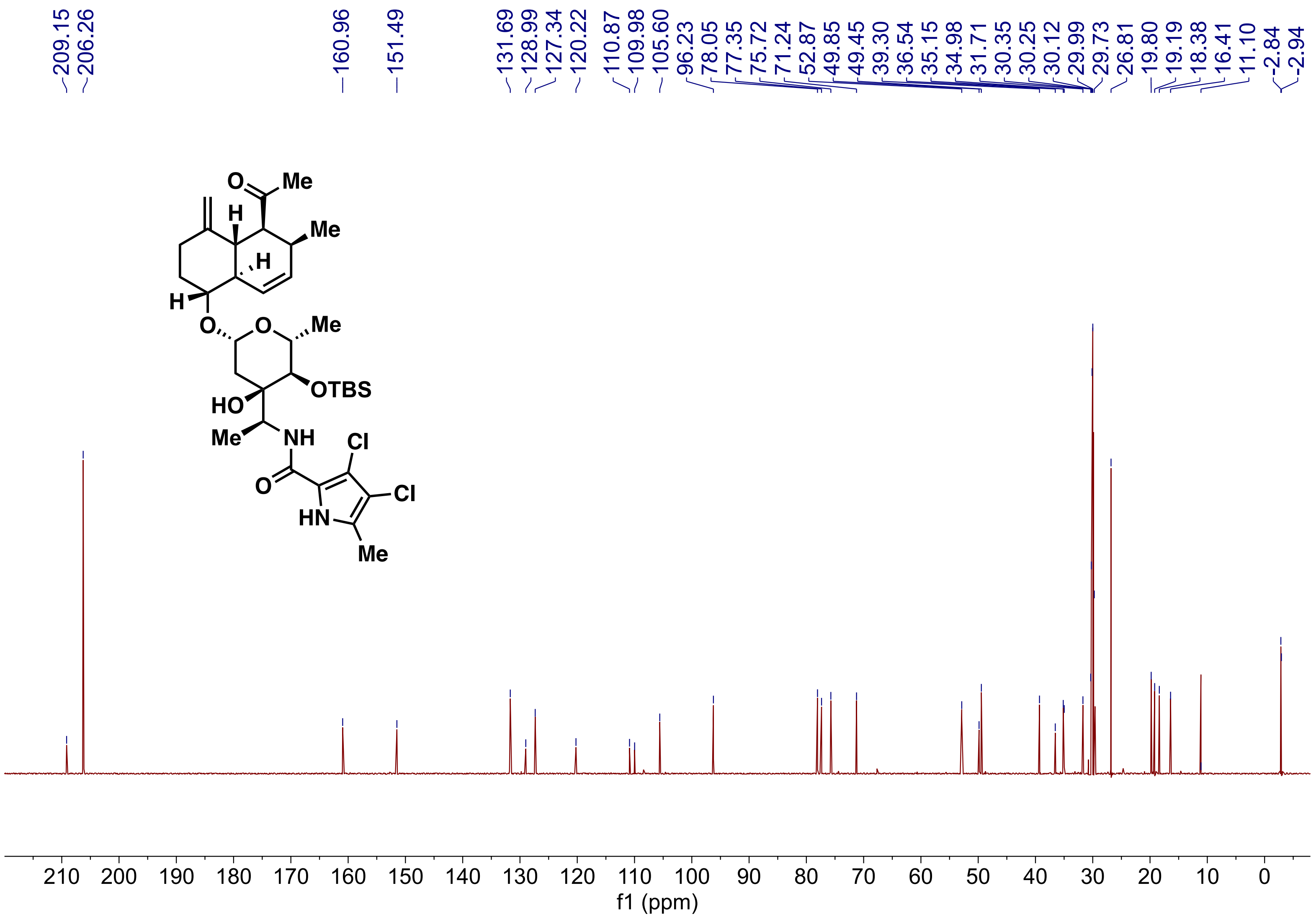
209.15
206.26
160.96
151.49
131.69
128.99
127.34
120.22
110.87
109.98
105.60
96.23
78.05
77.35
75.72
71.24
52.87
49.85
49.45
39.30
36.54
35.15
34.98
31.71
30.35
30.25
30.12
29.99
29.73
26.81
19.80
19.19
18.38
16.41
11.10
-2.84
-2.94
O
Me
H
Me
H
H
O
O
Me
OTBS
HO
Me
NH
Cl
O
Cl
HN
Me
210 200 190 180 170 160 150 140 130 120 110 100 90 80 70 60 50 40 30 20 10 0
f1 (ppm)

HSQC of **33**

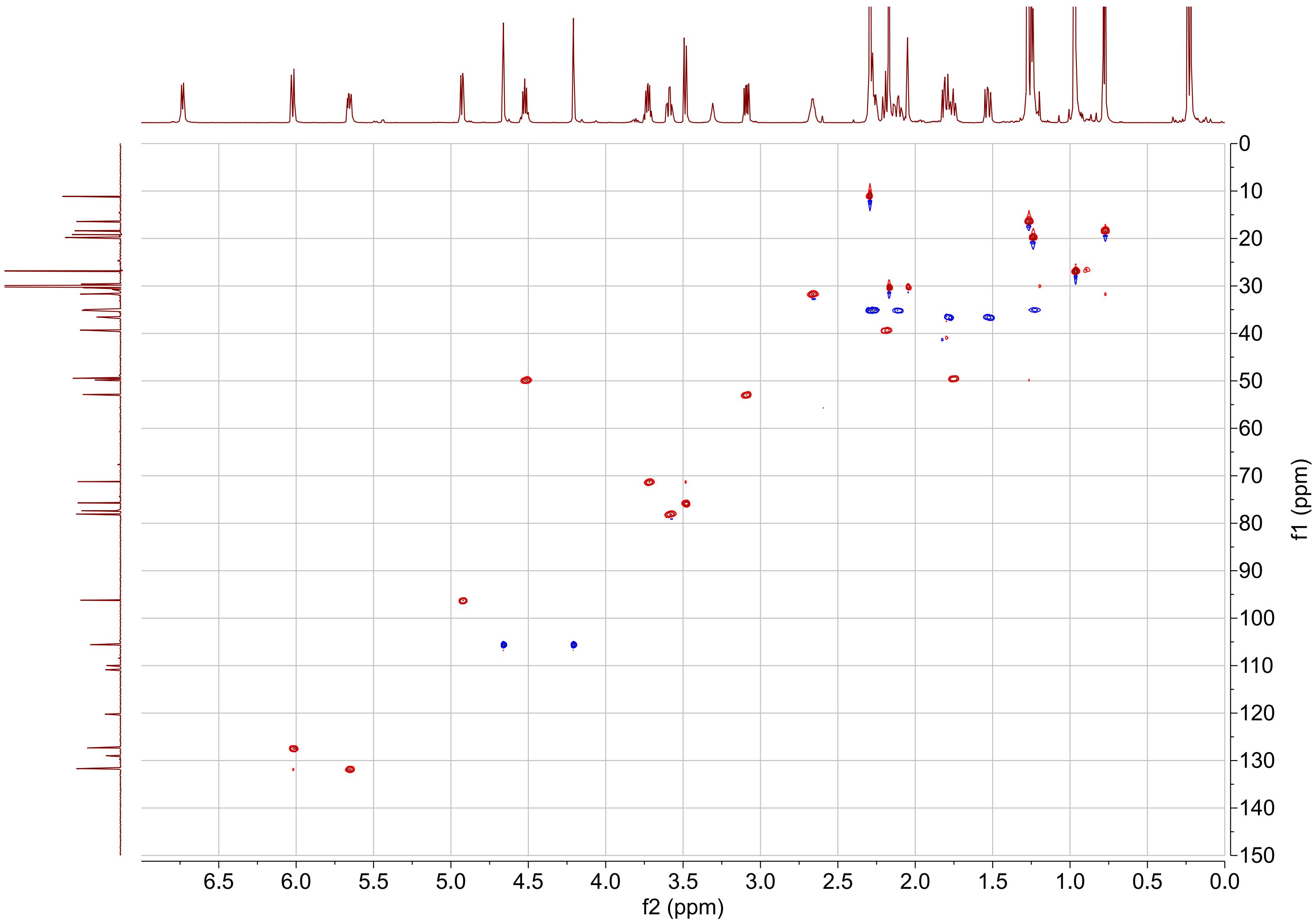

6.5
6.0
5.5
5.0
4.5
4.0
3.5
3.0
2.5
2.0
1.5
1.0
0.5
0.0
f2 (ppm)
0
10
20
30
40
50
60
70
80
90
100
110
120
130
140
150
f1 (ppm)

COSY of **33**

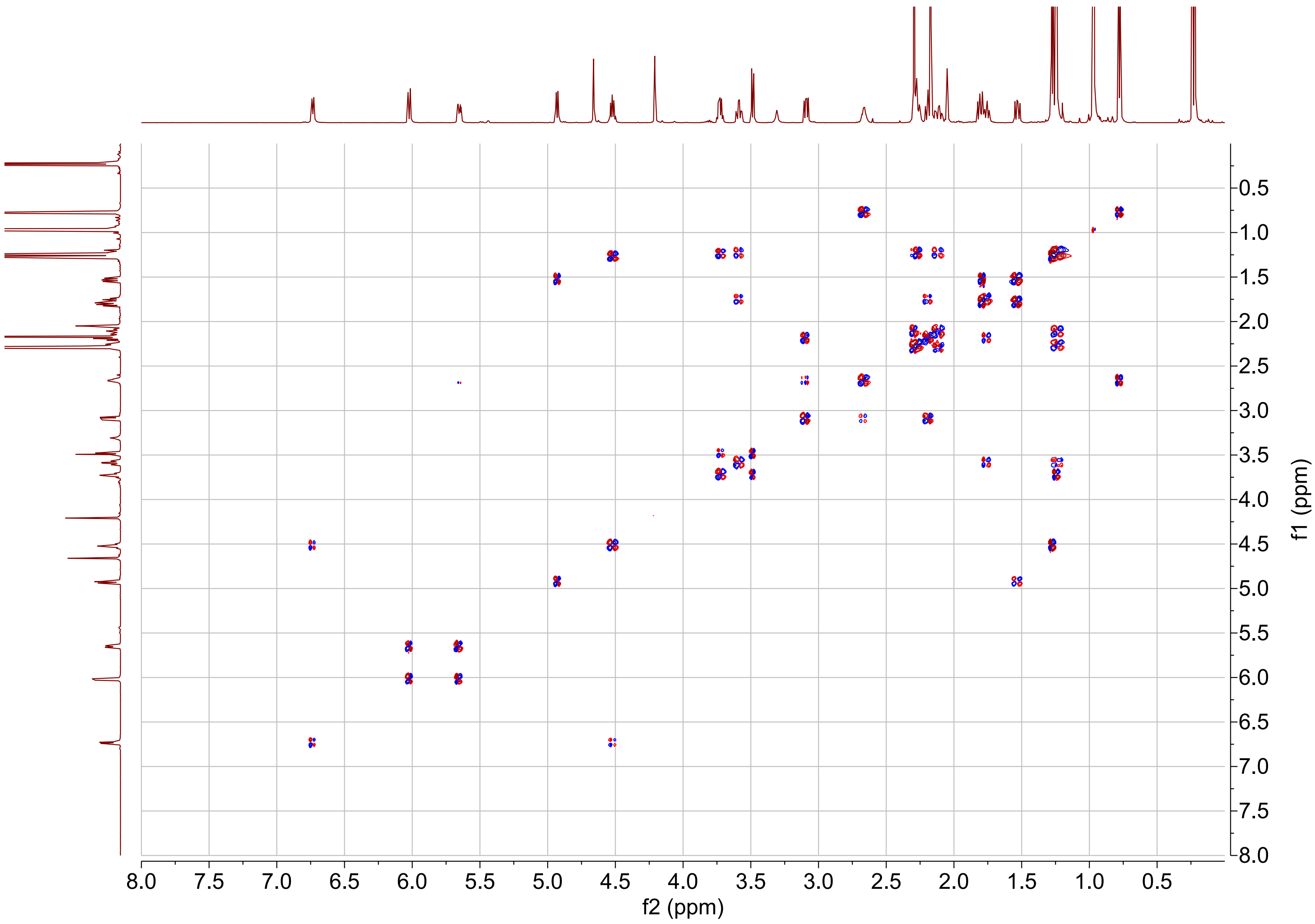

8.0 7.5 7.0 6.5 6.0 5.5 5.0 4.5 4.0 3.5 3.0 2.5 2.0 1.5 1.0 0.5
f2 (ppm)
0.5 1.0 1.5 2.0 2.5 3.0 3.5 4.0 4.5 5.0 5.5 6.0 6.5 7.0 7.5 8.0
f1 (ppm)

NOESY of **33**

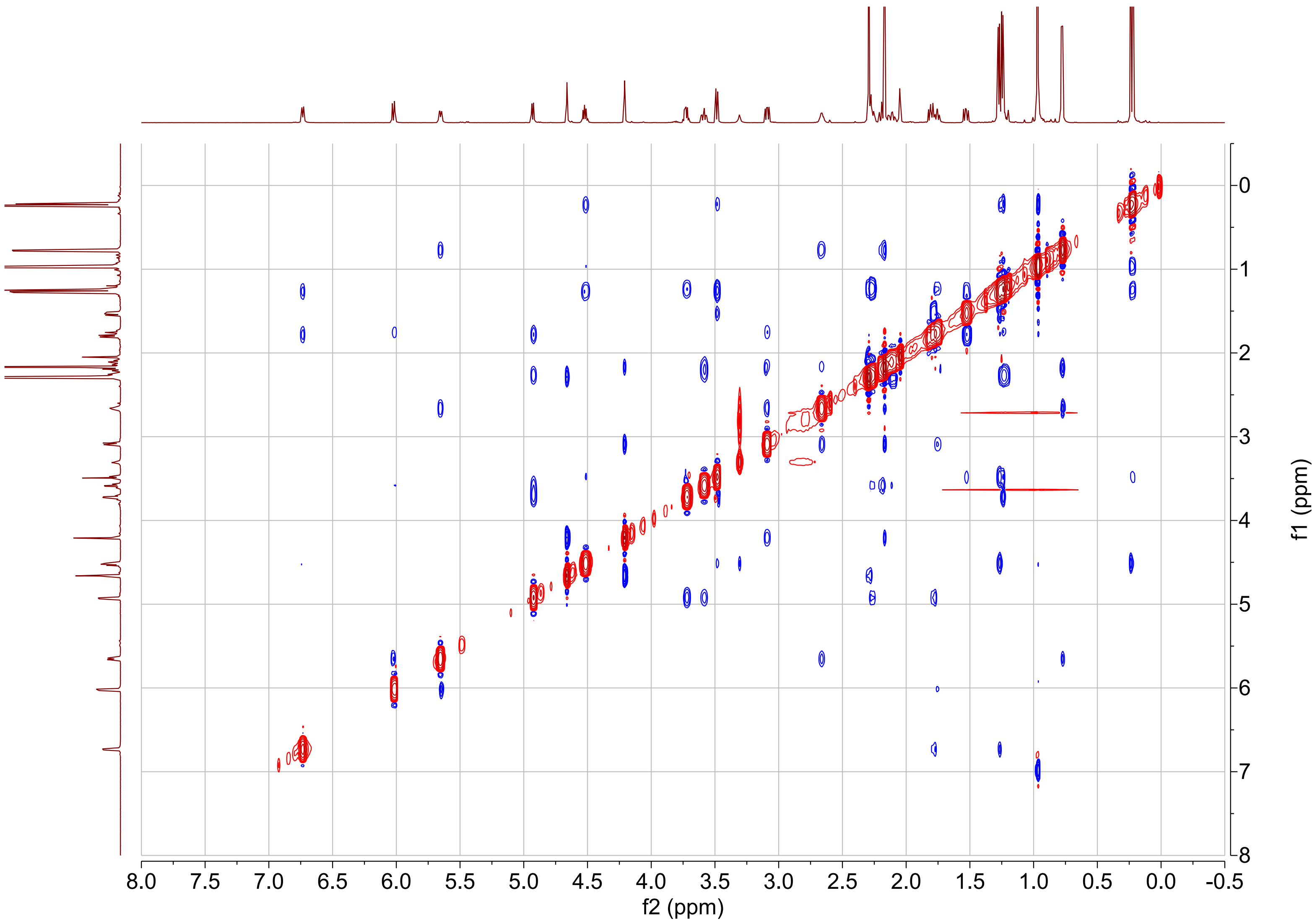

f2 (ppm)
f1 (ppm)
8.0
7.5
7.0
6.5
6.0
5.5
5.0
4.5
4.0
3.5
3.0
2.5
2.0
1.5
1.0
0.5
0.0
-0.5
0
1
2
3
4
5
6
7
8

^{1}H NMR of **34**

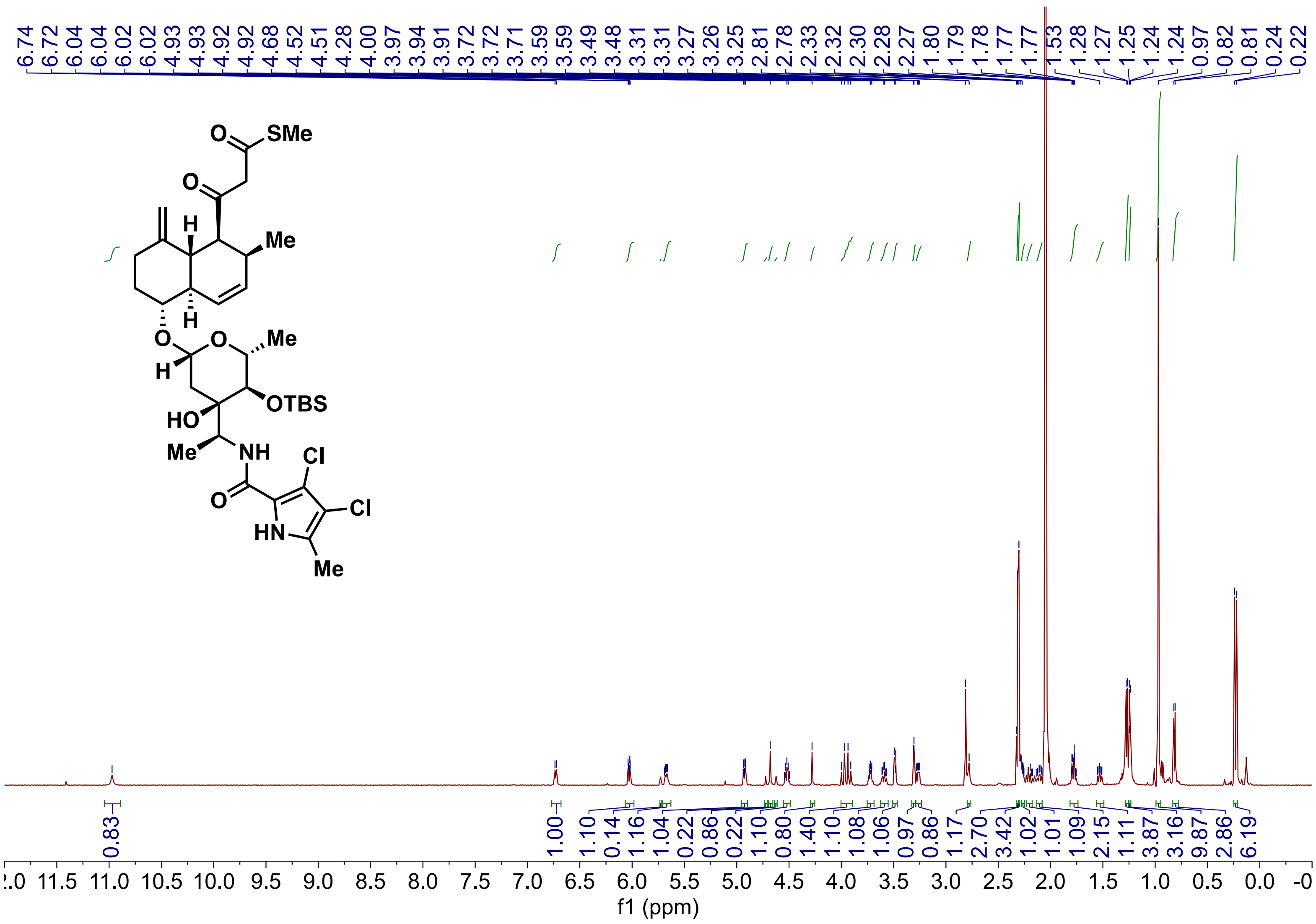

^{13}C NMR of **34**

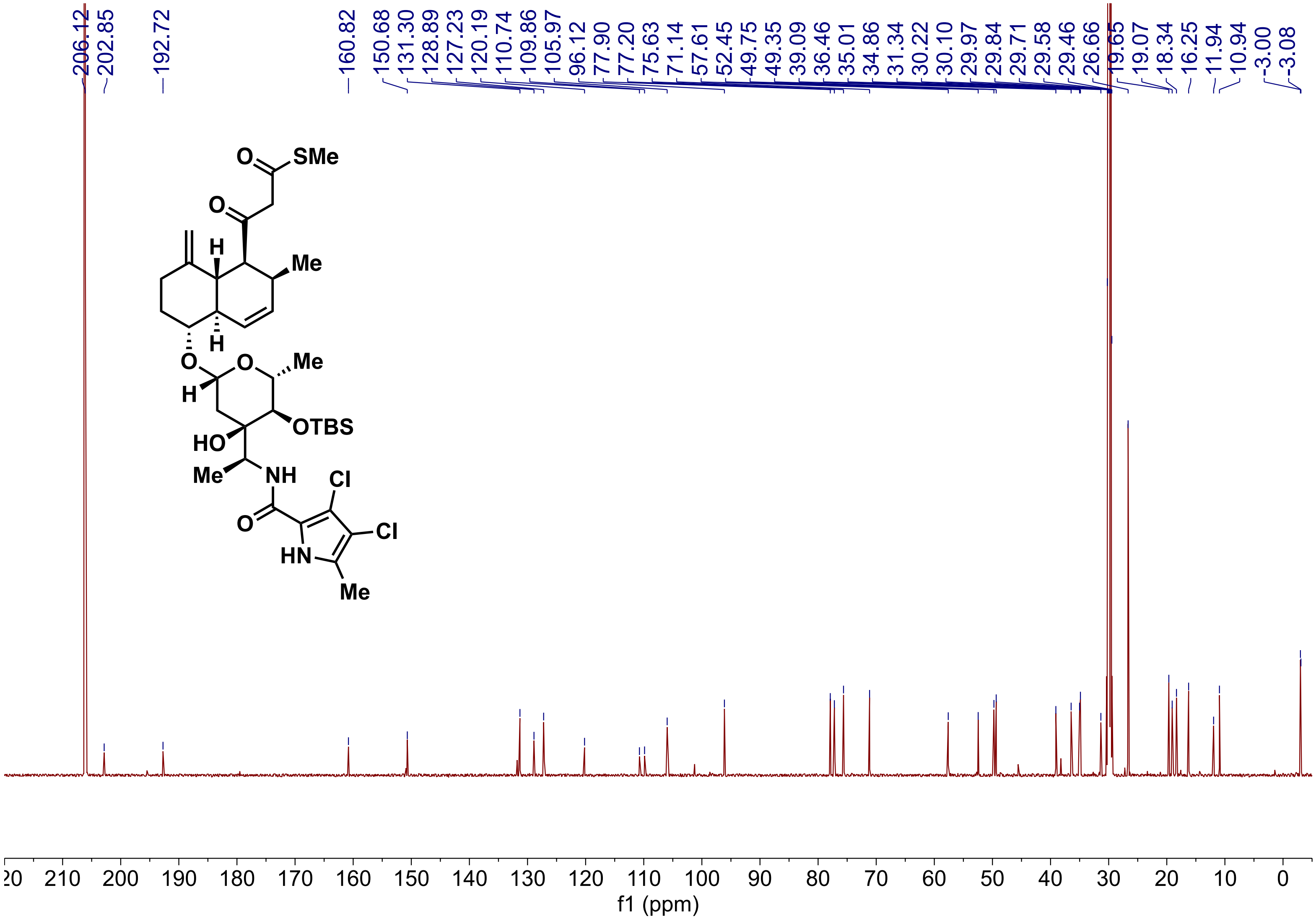

206.12
202.85
192.72
160.82
150.68
131.30
128.89
127.23
120.19
110.74
109.86
105.97
96.12
77.90
77.20
75.63
71.14
57.61
52.45
49.75
49.35
39.09
36.46
35.01
34.86
31.34
30.22
30.10
29.97
29.84
29.71
29.58
29.46
26.66
19.65
19.07
18.34
16.25
11.94
10.94
-3.00
-3.08
O
SMe
O
H
Me
H
O
O
Me
H
OTBS
HO
Me
NH
Cl
O
Cl
HN
Me
f1 (ppm)

^{1}H NMR of **35** (*epi*-KBD)

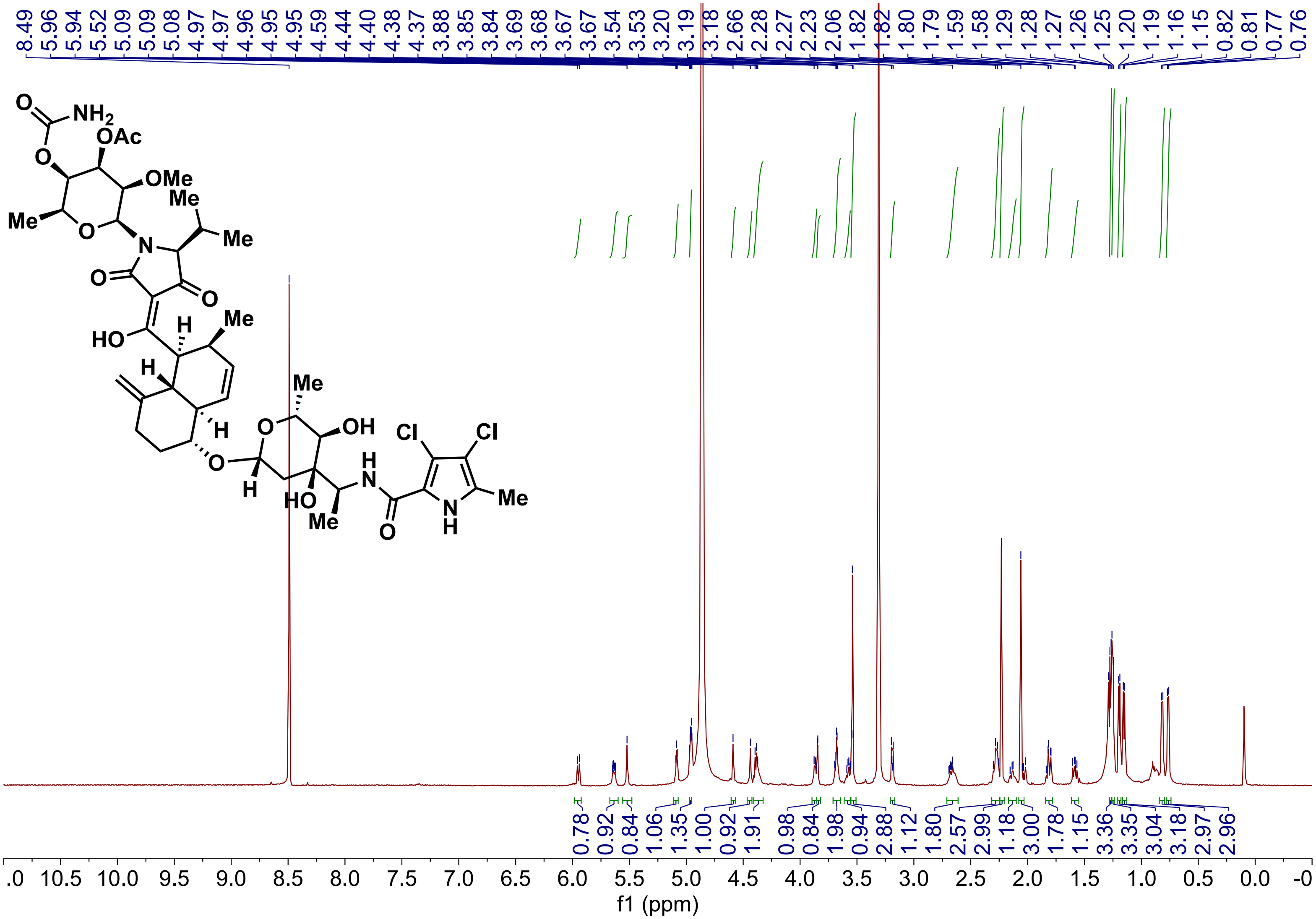

^{13}C NMR of **35** (*epi*-KBD)

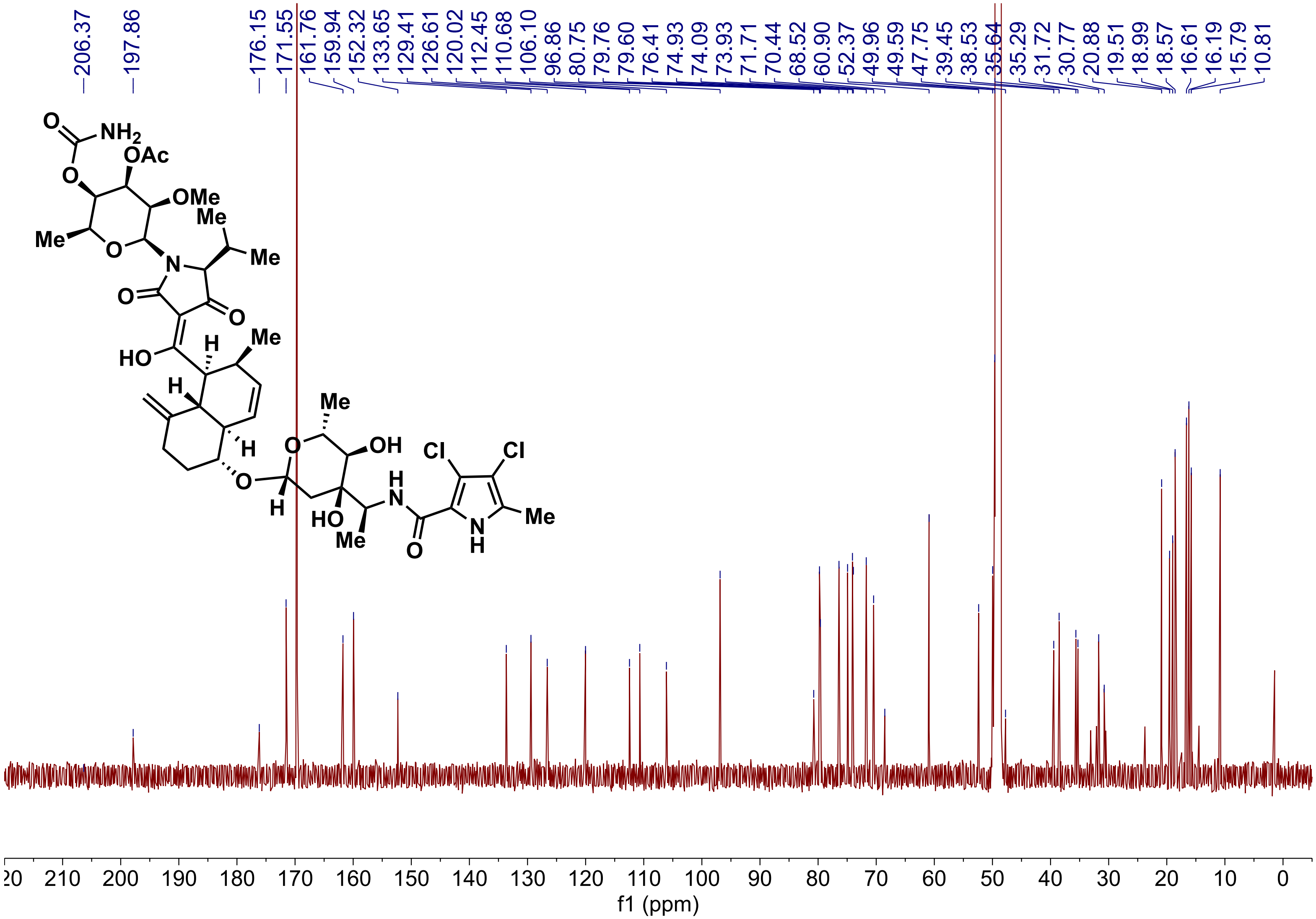

HSQC of **35** (*epi*-KBD)

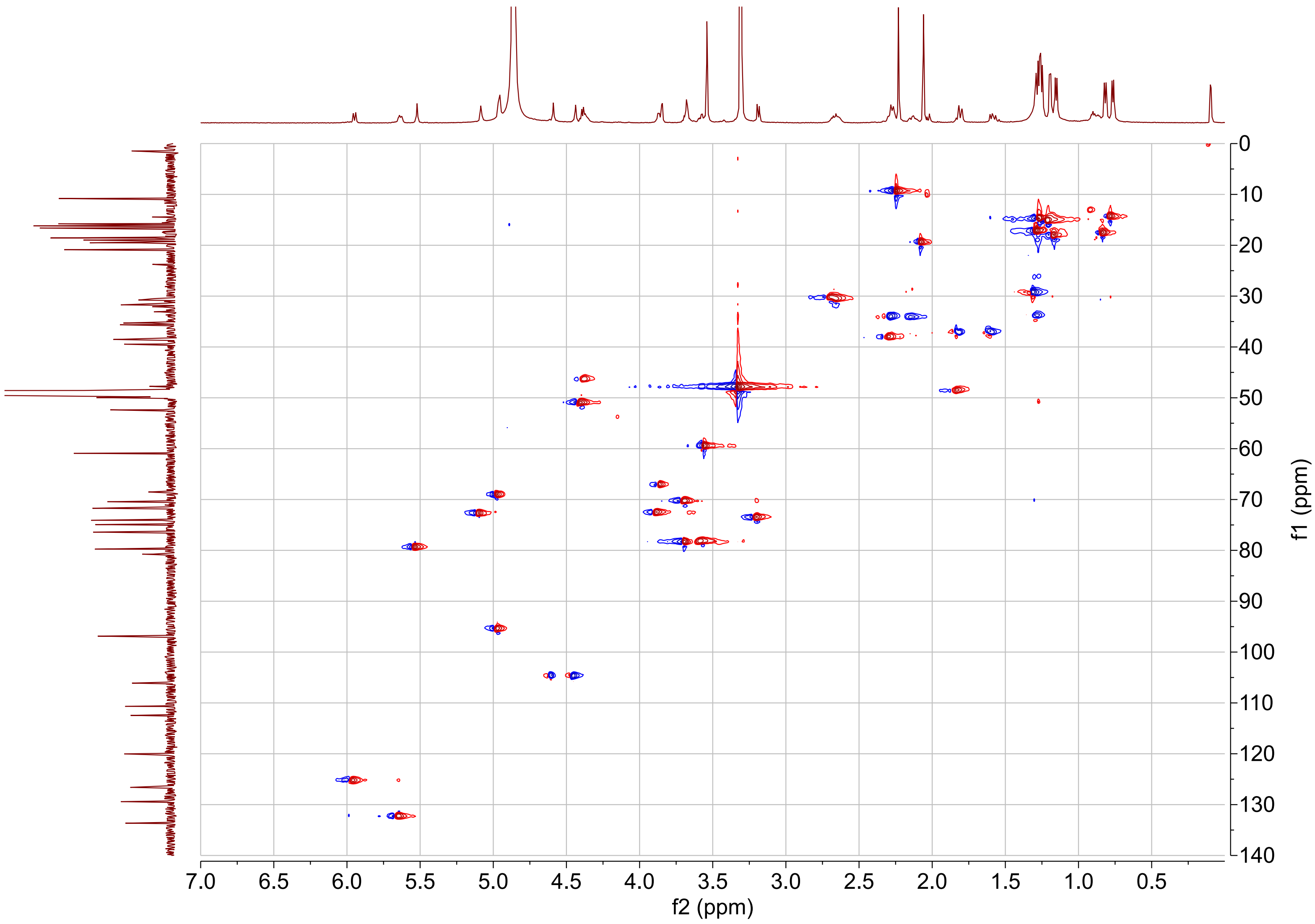

COSY of **35** (*epi*-KBD)

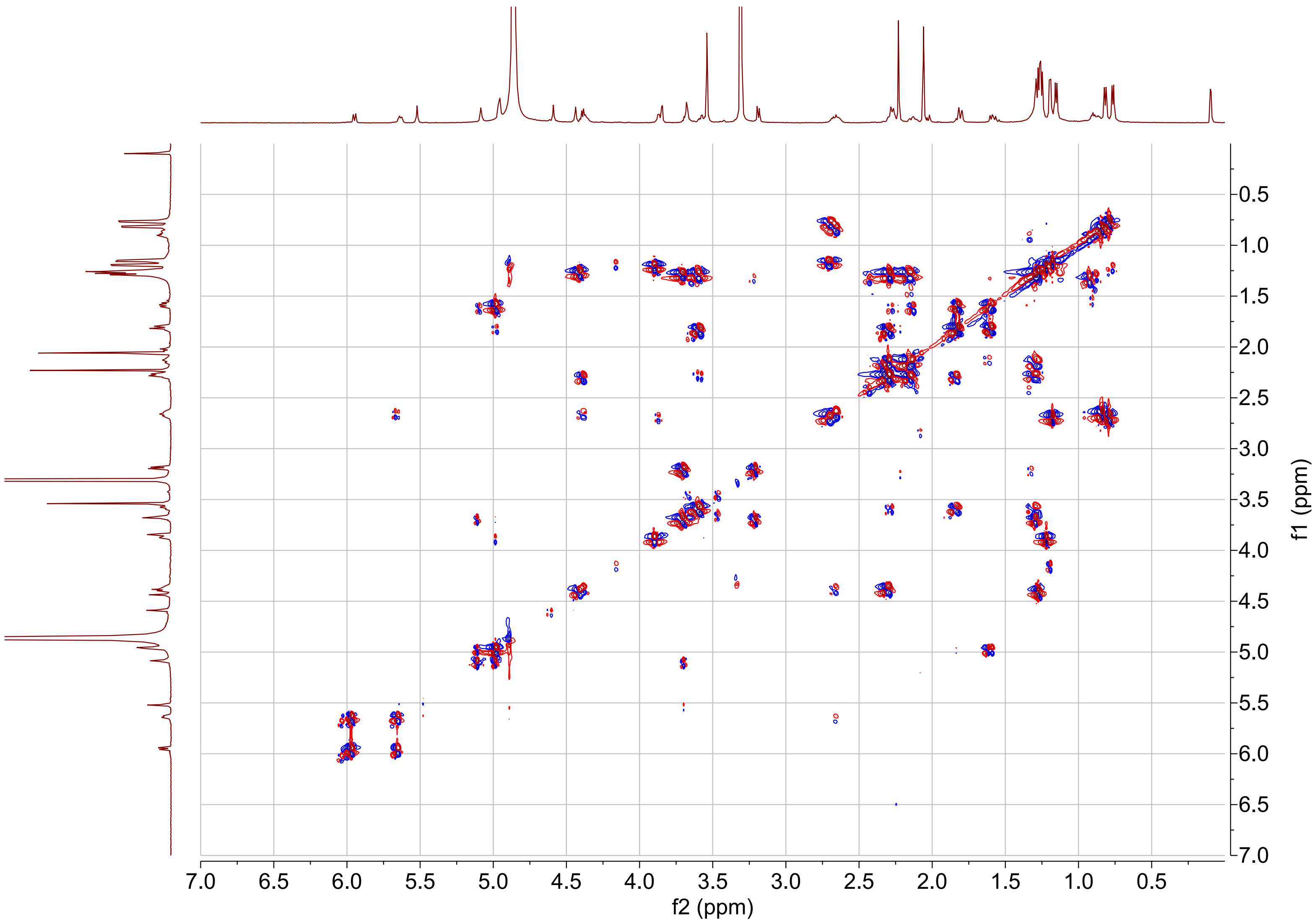

NOESY of **35** (*epi*-KBD)

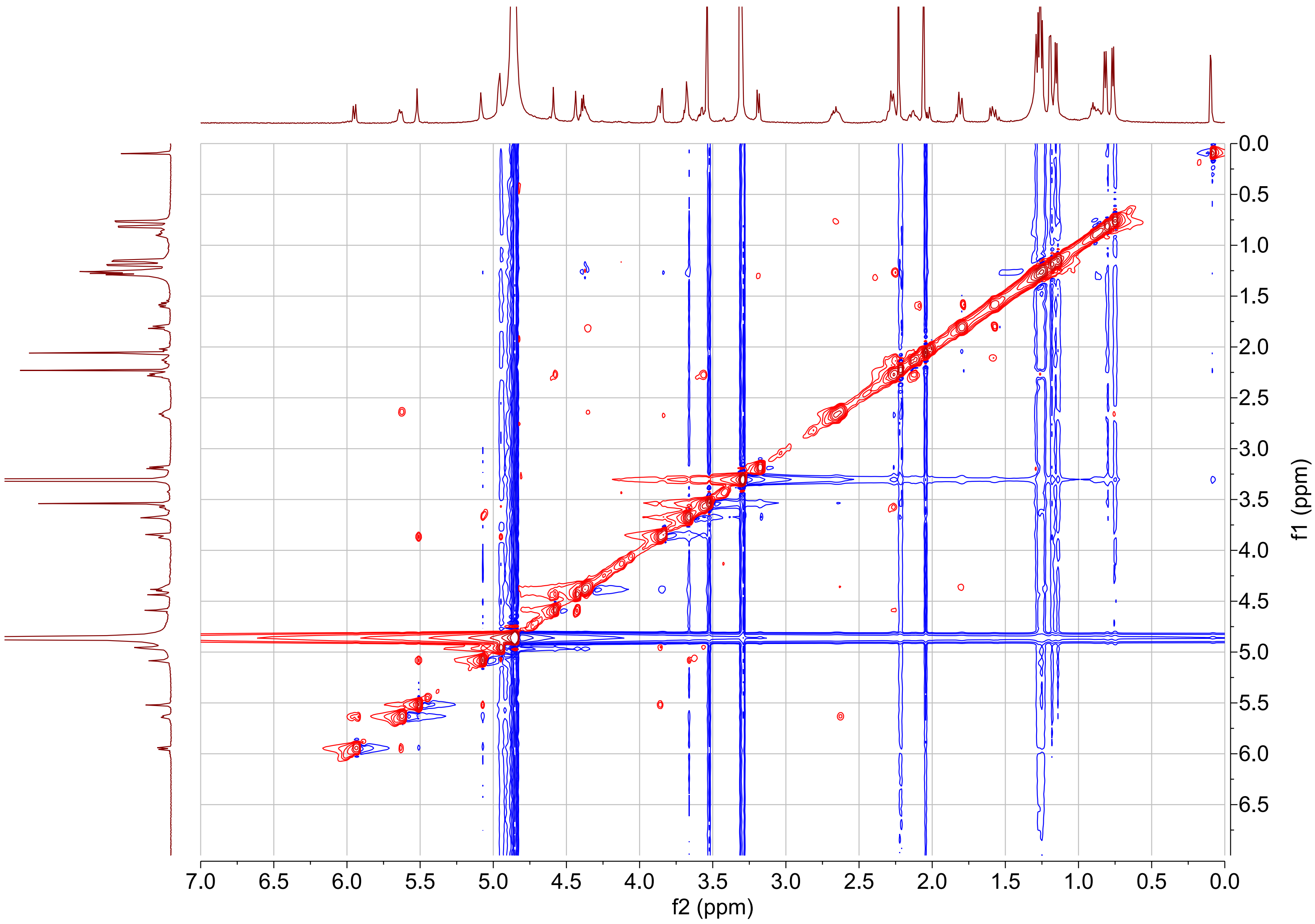

^{1}H NMR of **1b** (KBD/amy)

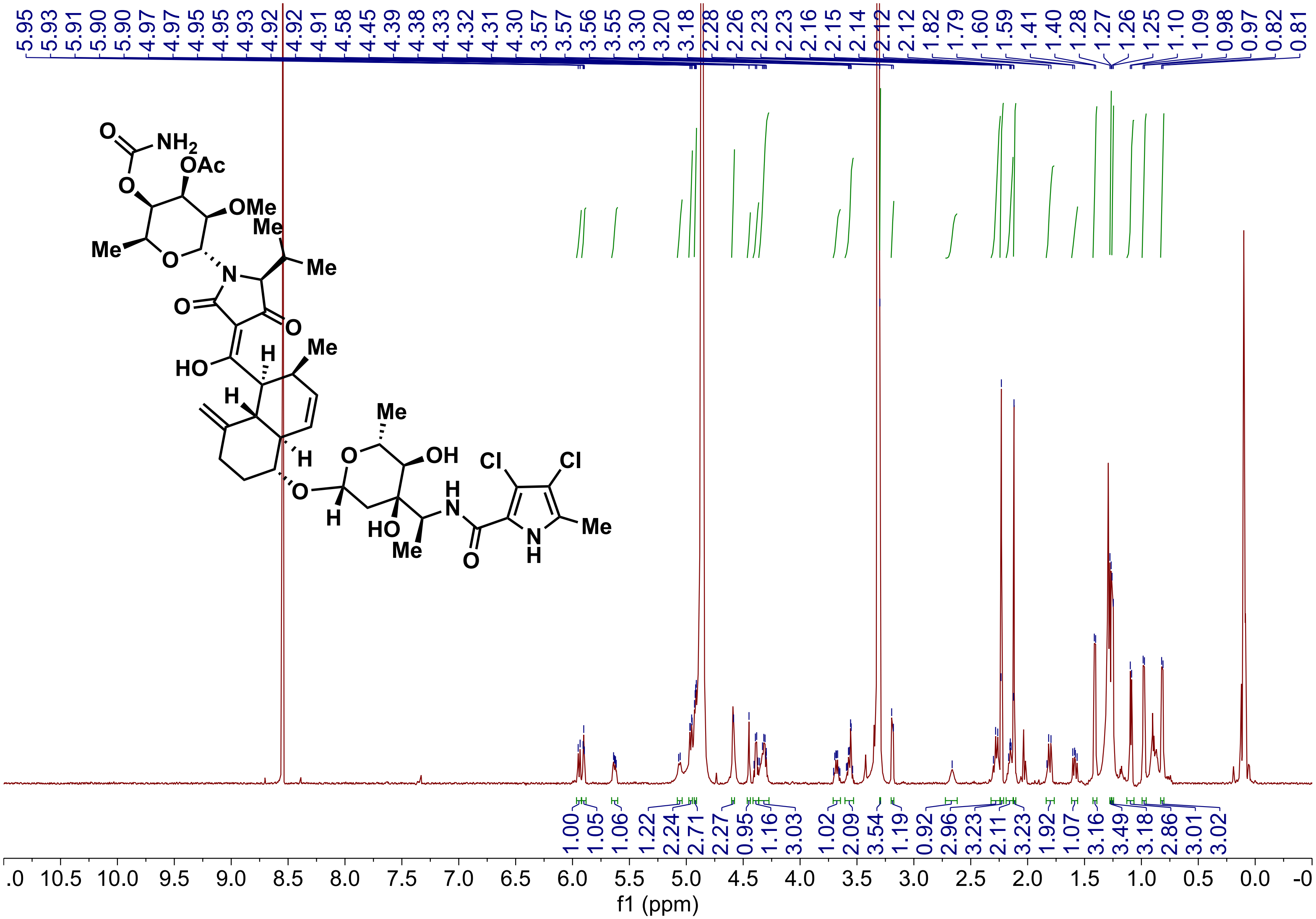

^{13}C NMR of **1b** (KBD/amy)

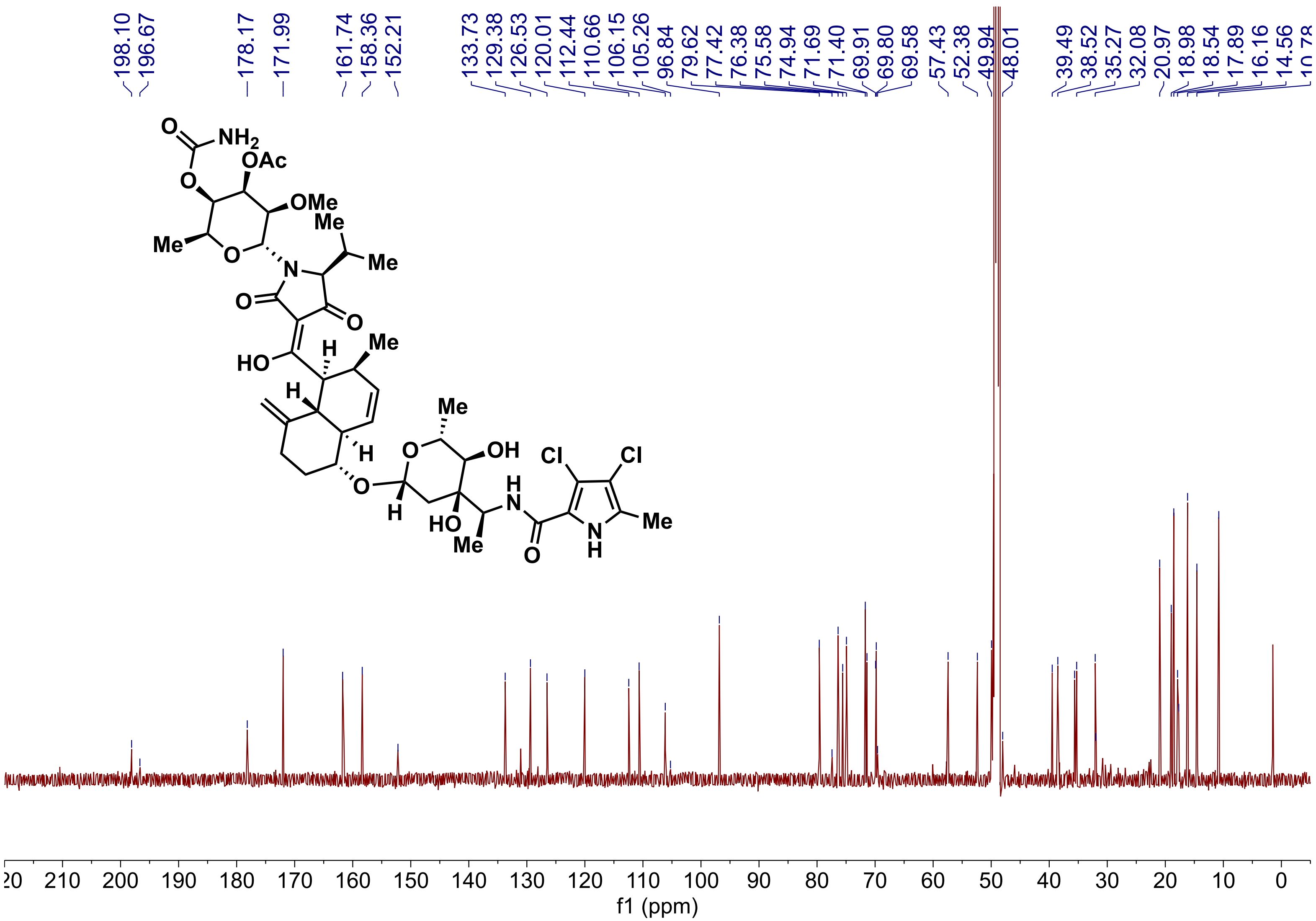

^{1}H NMR of **36**

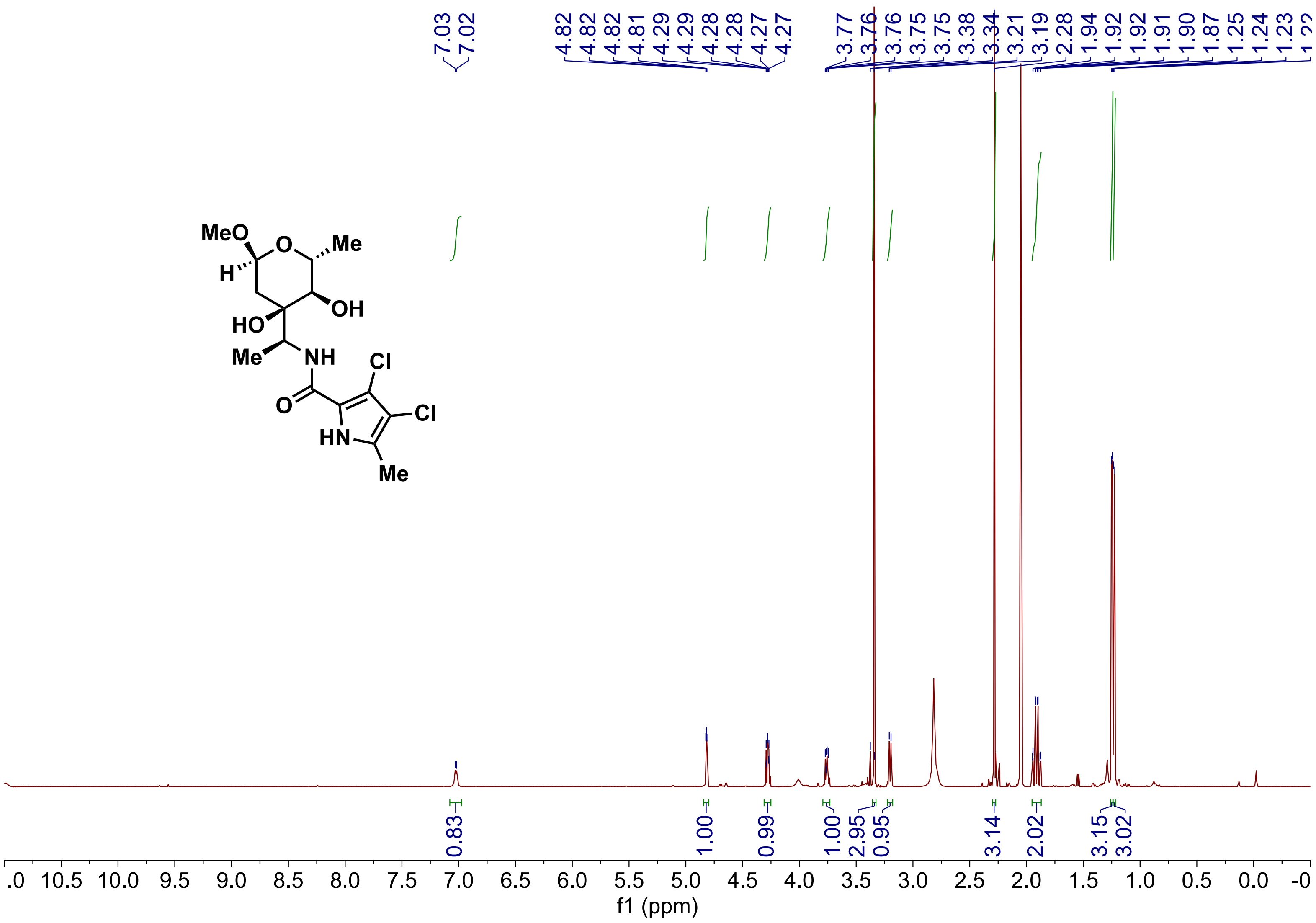

^{13}C NMR of **36**

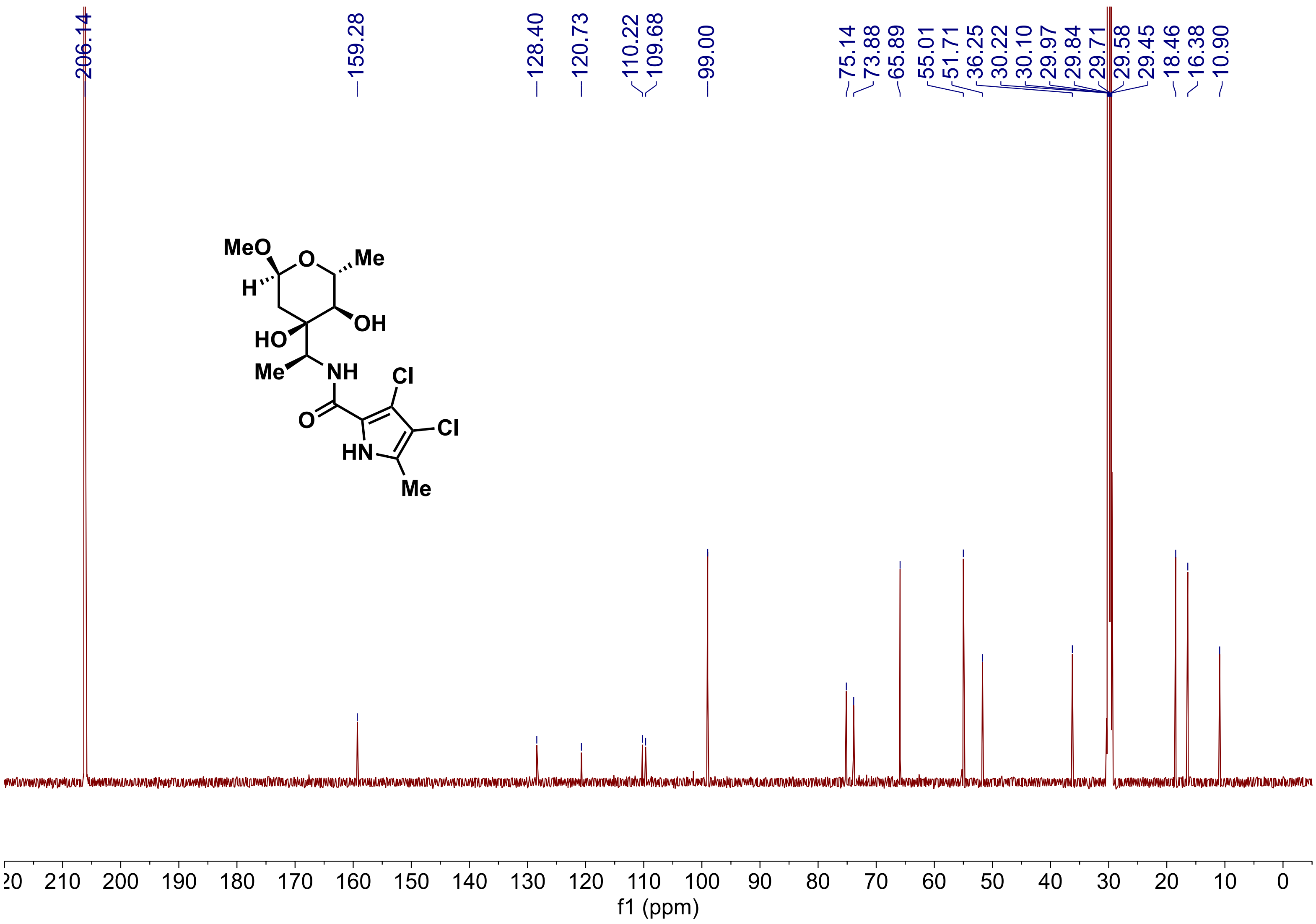
206.14
159.28
128.40
120.73
110.22
109.68
99.00
75.14
73.88
65.89
55.01
51.71
36.25
30.22
30.10
29.97
29.84
29.71
29.58
29.45
18.46
16.38
10.90
MeO
O
Me
H
OH
HO
Me
NH
Cl
Cl
O
HN
Me
20 210 200 190 180 170 160 150 140 130 120 110 100 90 80 70 60 50 40 30 20 10 0
f1 (ppm)

HSQC of **36**

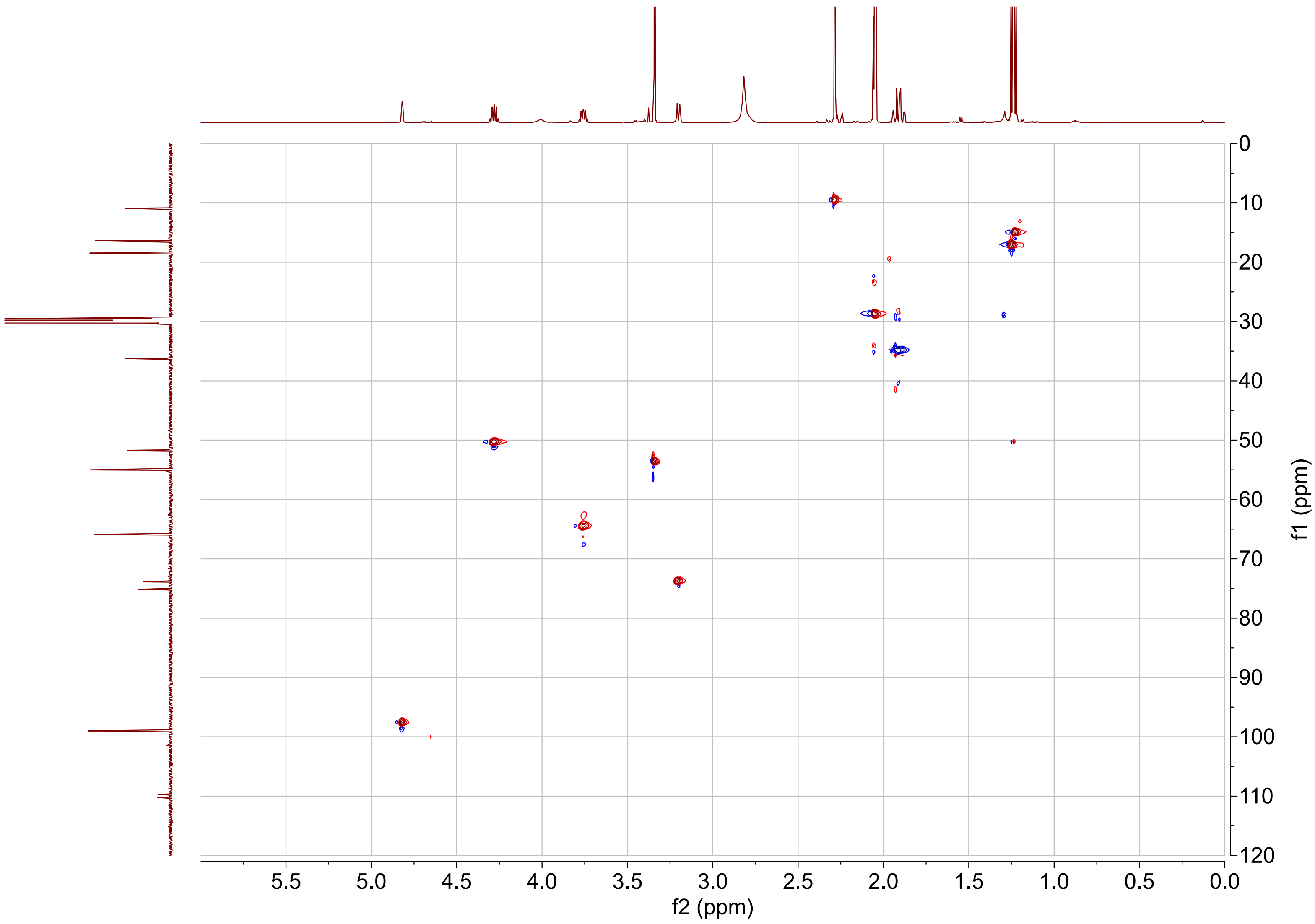

f2 (ppm)
f1 (ppm)
5.5
5.0
4.5
4.0
3.5
3.0
2.5
2.0
1.5
1.0
0.5
0.0
0
10
20
30
40
50
60
70
80
90
100
110
120

COSY of **36**

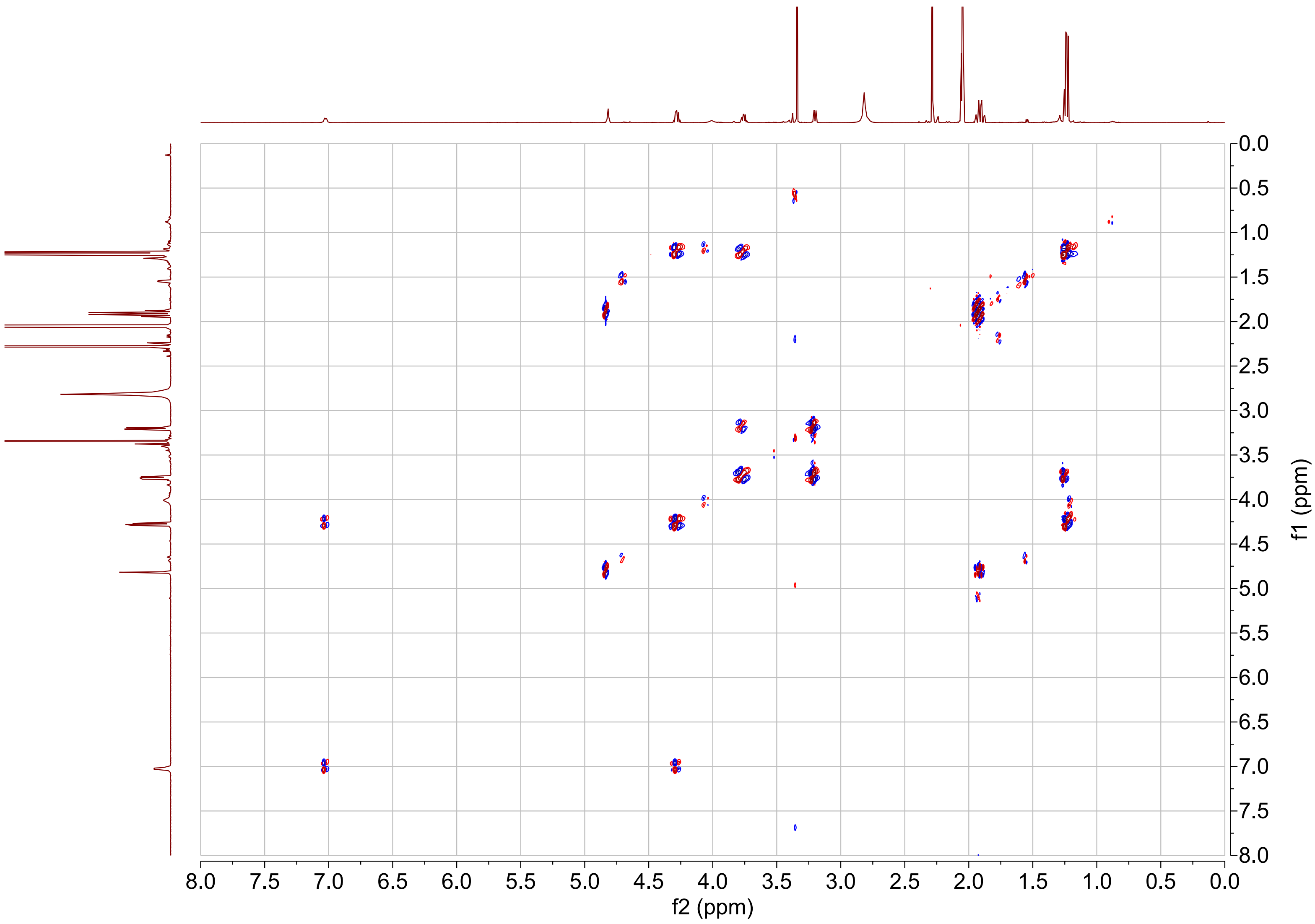

NOESY of **36**

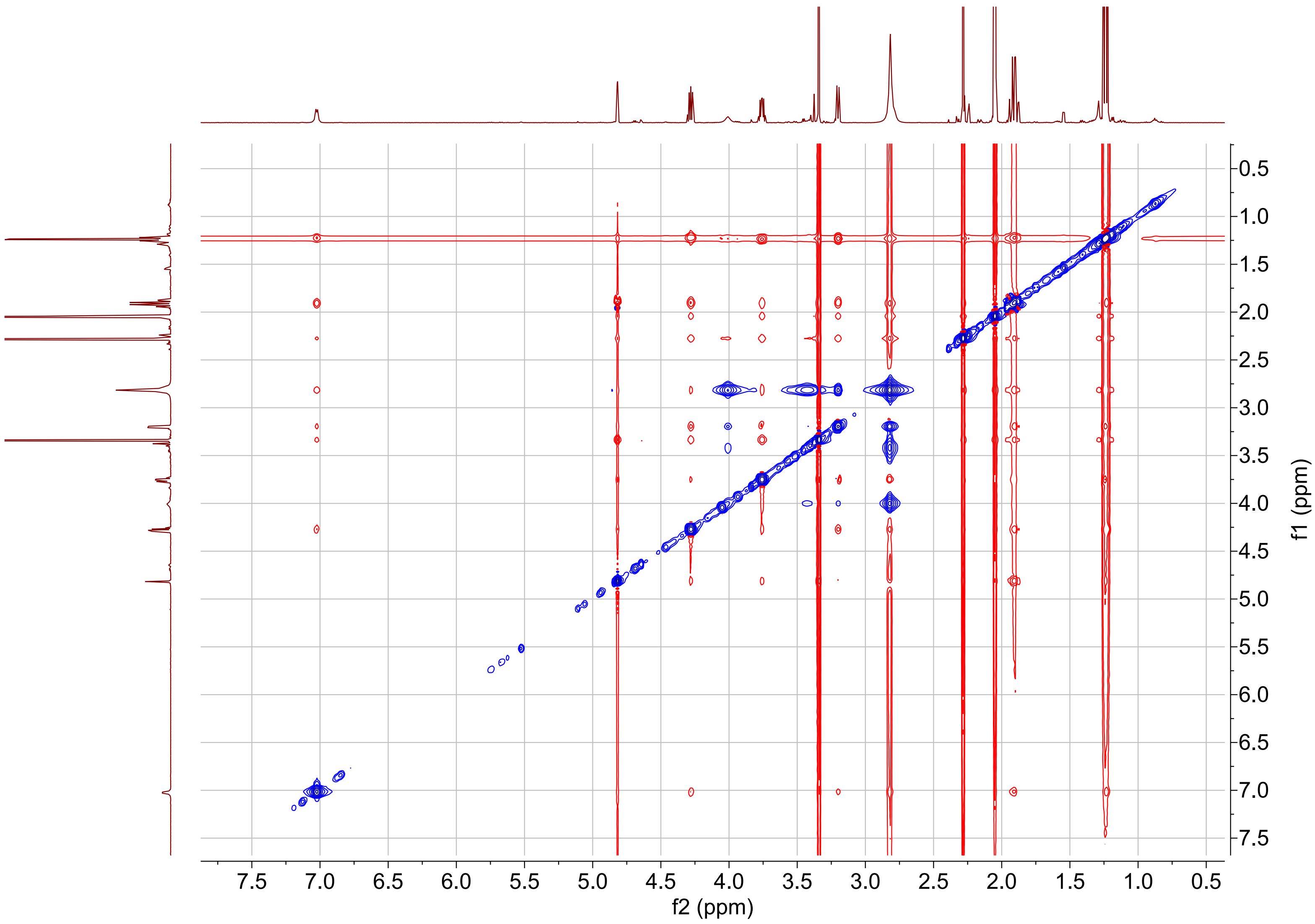

^{1}H NMR of **37**

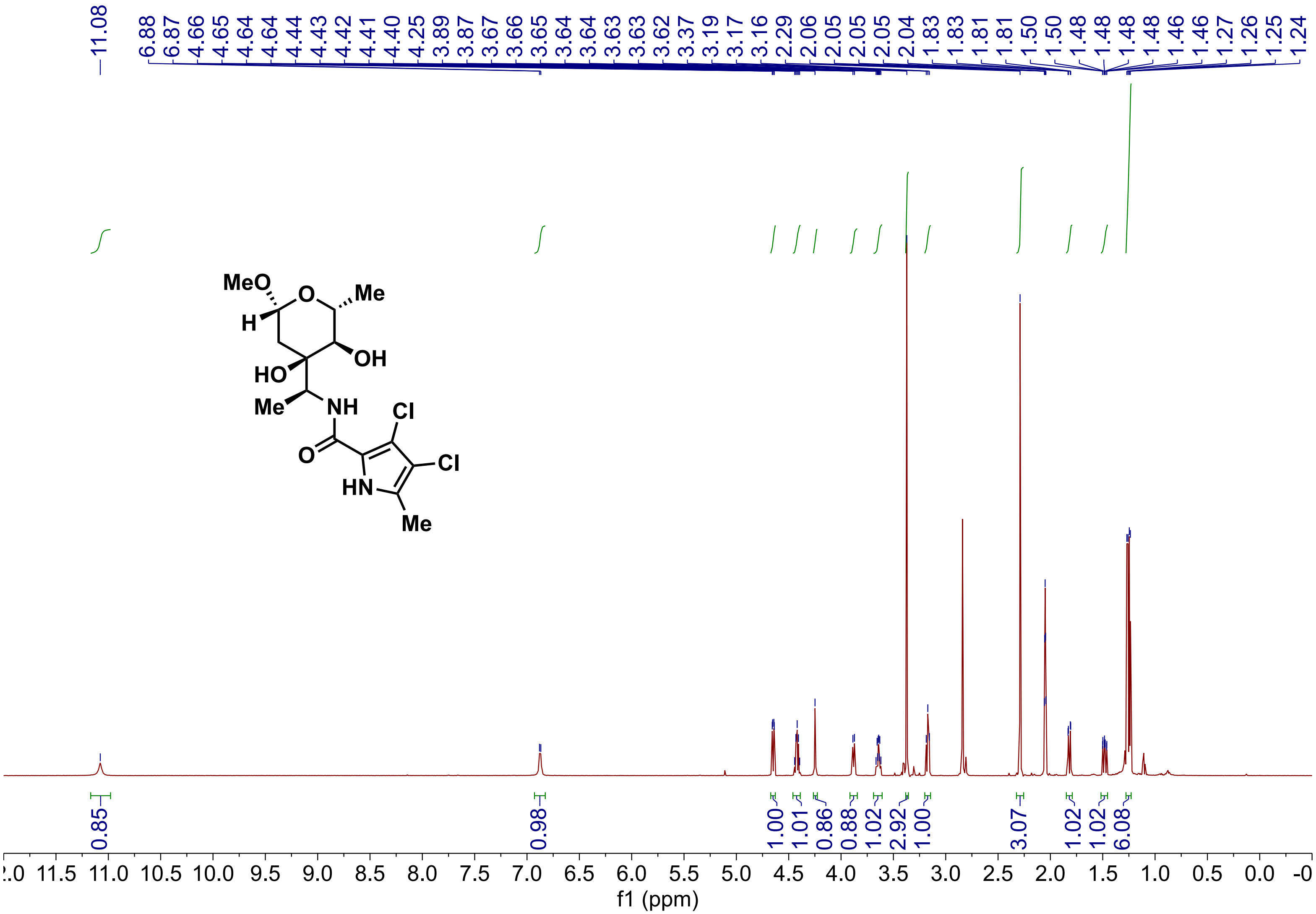

11.08
6.88
6.87
4.66
4.65
4.64
4.64
4.44
4.43
4.42
4.41
4.40
4.25
3.89
3.87
3.67
3.66
3.65
3.64
3.64
3.63
3.63
3.62
3.37
3.19
3.17
3.16
2.29
2.06
2.05
2.05
2.05
2.04
1.83
1.83
1.81
1.81
1.50
1.50
1.48
1.48
1.48
1.48
1.46
1.46
1.27
1.26
1.25
1.24
MeO
O
Me
H
OH
HO
Me
NH
Cl
Cl
O
HN
Me
0.85
0.98
1.00
1.01
0.86
0.88
1.02
2.92
1.00
3.07
1.02
1.02
6.08
f1 (ppm)

^{13}C NMR of **37**

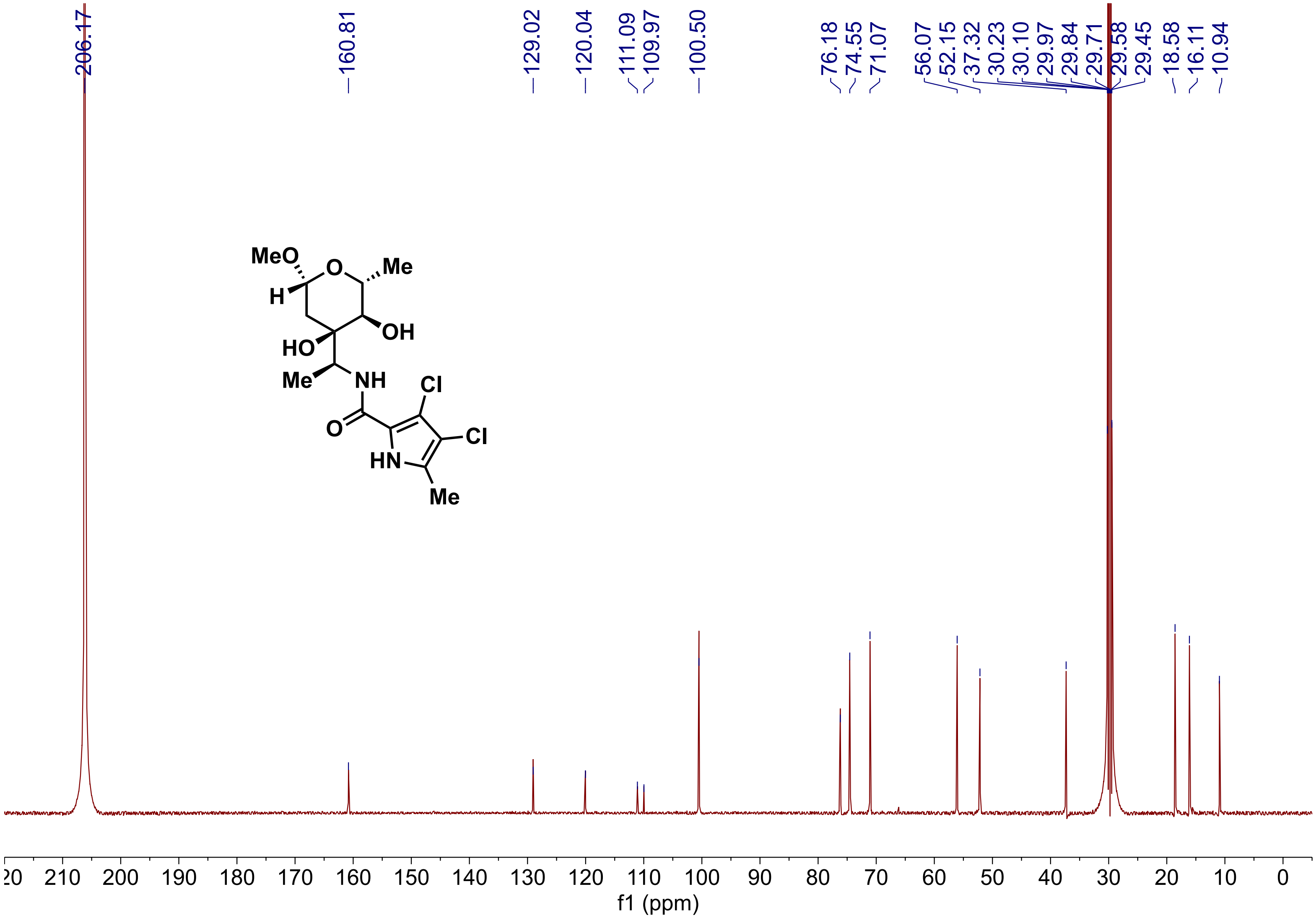
206.17
160.81
129.02
120.04
111.09
109.97
100.50
76.18
74.55
71.07
56.07
52.15
37.32
30.23
30.10
29.97
29.84
29.71
29.58
29.45
18.58
16.11
10.94
MeO
O
Me
H
OH
HO
Me
NH
Cl
Cl
O
HN
Me
220 210 200 190 180 170 160 150 140 130 120 110 100 90 80 70 60 50 40 30 20 10 0
f1 (ppm)

HSQC of **37**

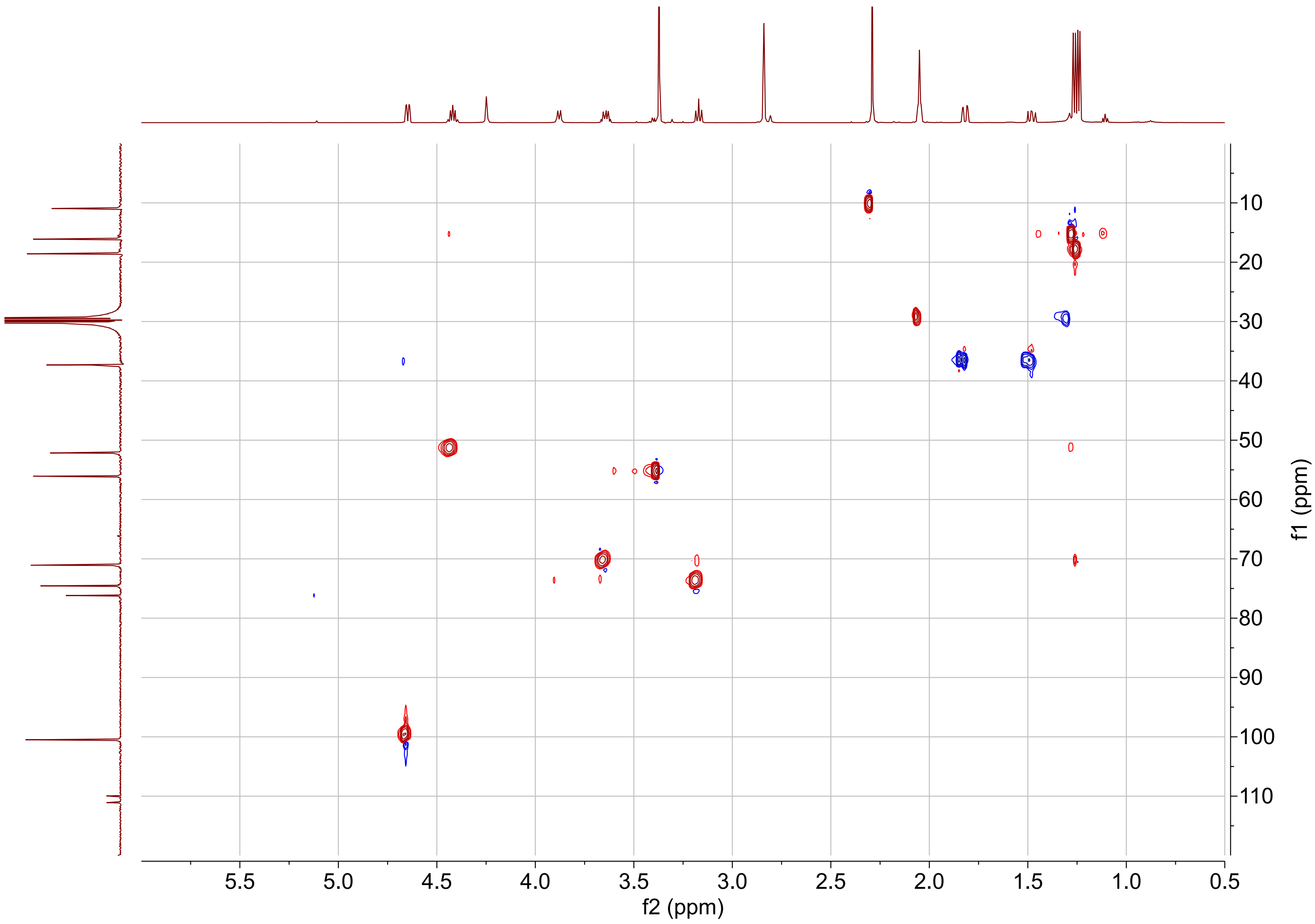

10
20
30
40
50
60
70
80
90
100
110
f1 (ppm)
5.5
5.0
4.5
4.0
3.5
3.0
2.5
2.0
1.5
1.0
0.5
f2 (ppm)

COSY of **37**

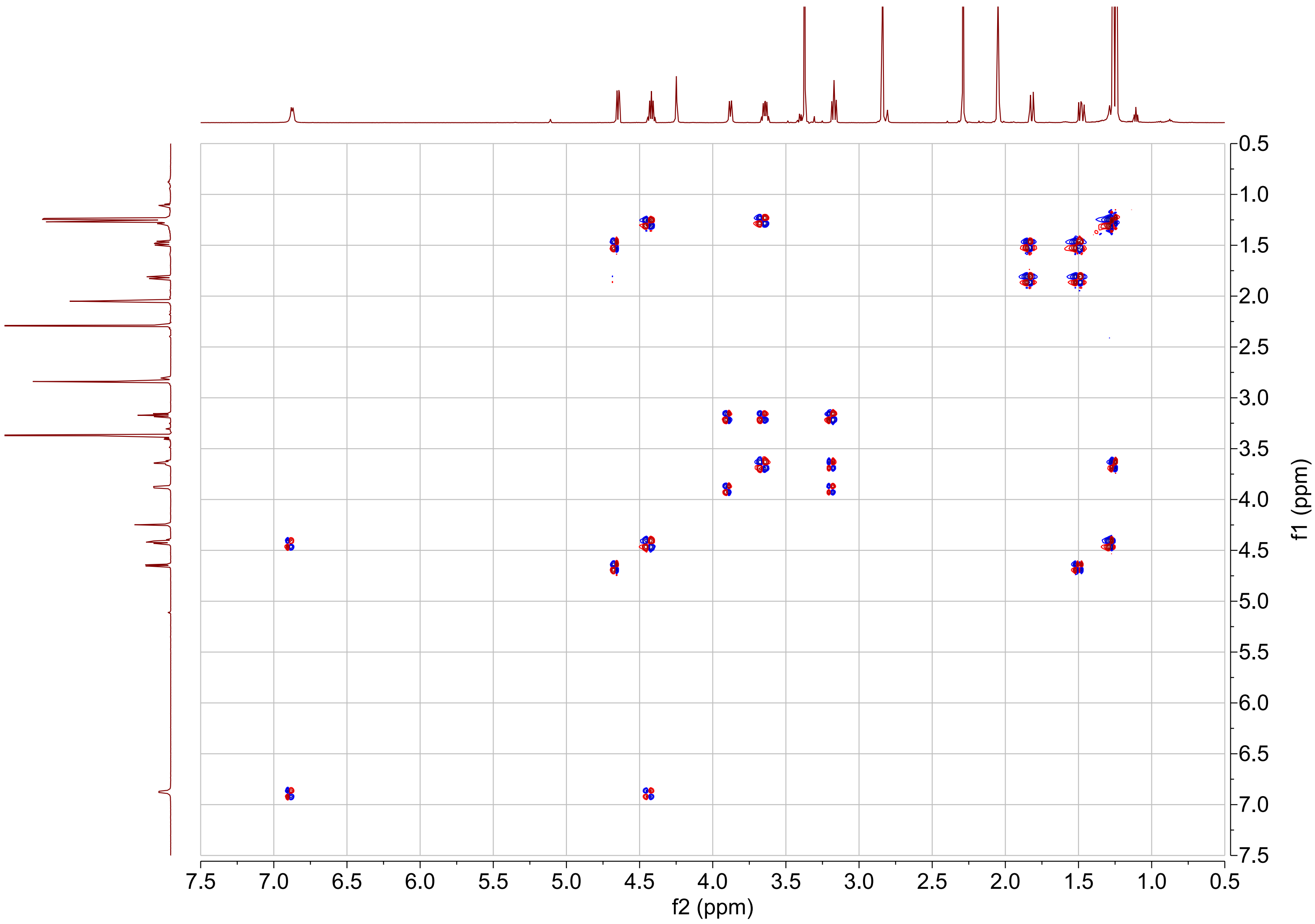

NOESY of **37**

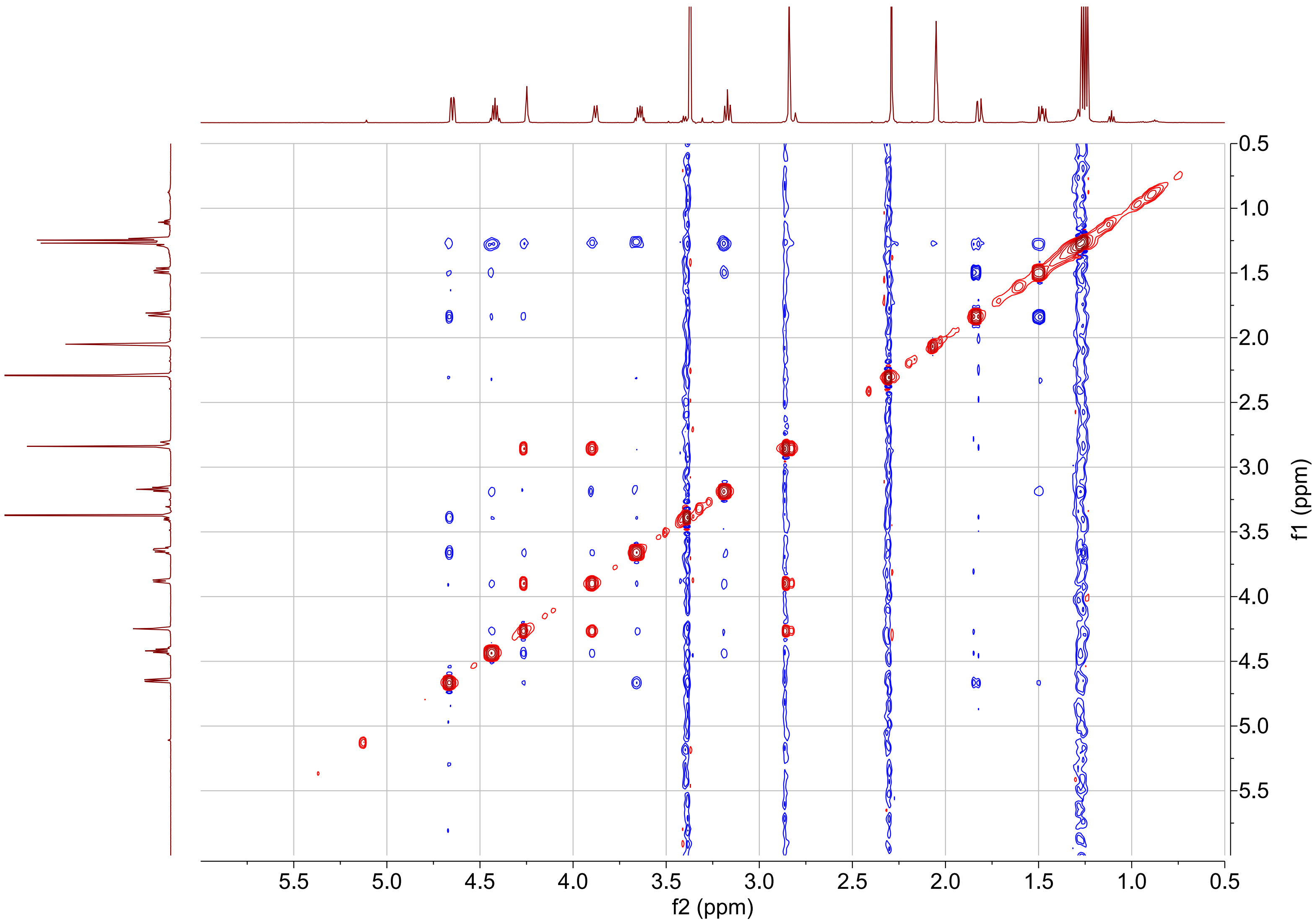

^{1}H NMR of **38**

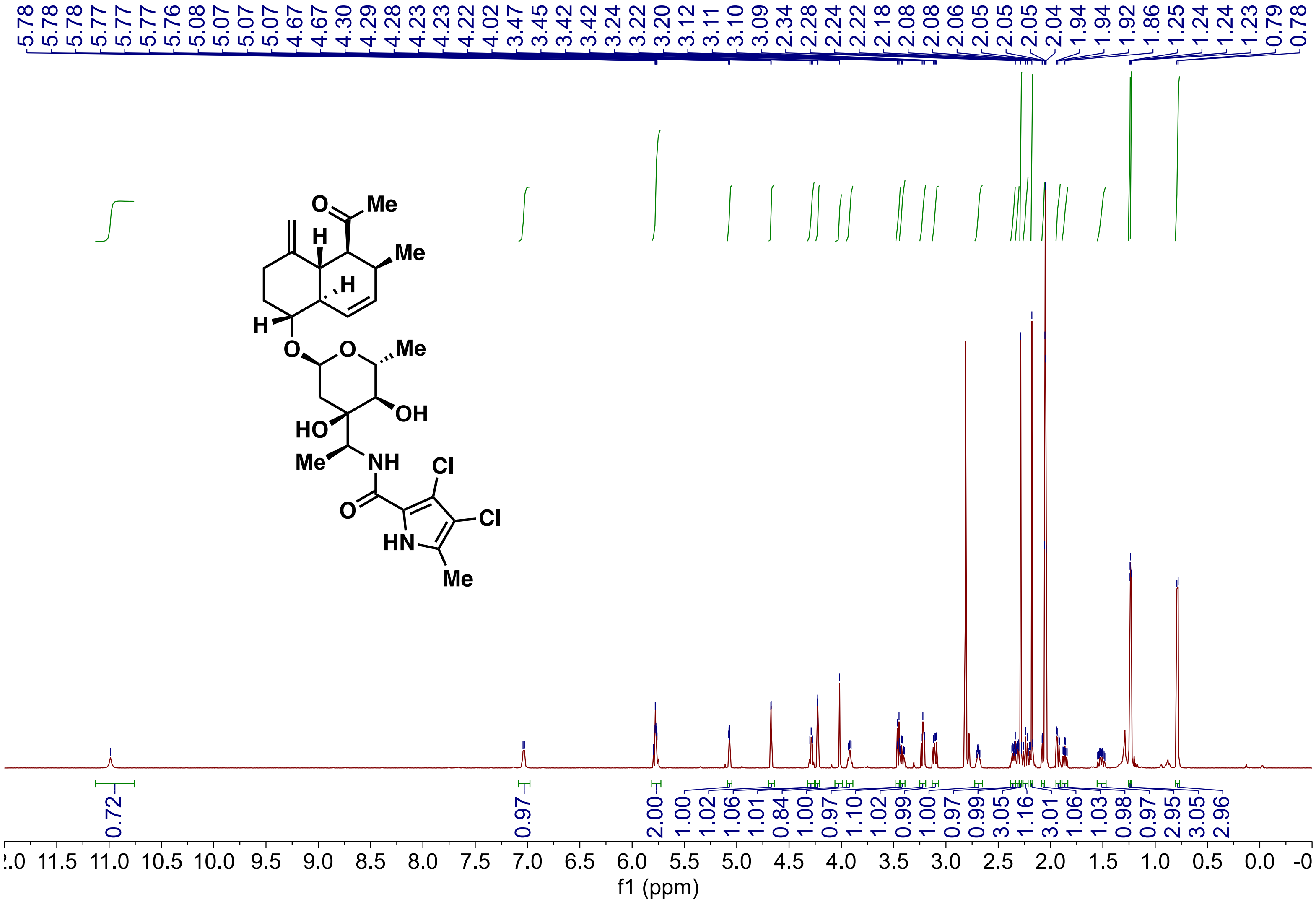
5.78 5.78 5.78 5.77 5.77 5.77 5.76 5.08 5.07 5.07 5.07 4.67 4.67 4.30 4.29 4.28 4.23 4.23 4.22 4.02 3.47 3.45 3.42 3.42 3.24 3.22 3.20 3.12 3.11 3.10 3.09 2.34 2.28 2.24 2.22 2.18 2.08 2.08 2.06 2.05 2.05 2.05 2.04 1.94 1.94 1.92 1.86 1.25 1.24 1.24 1.23 0.79 0.78
O
Me
H
Me
H
H
O
O
Me
OH
HO
Me
NH
Cl
Cl
O
HN
Me
0.72 0.97 2.00 1.00 1.02 1.06 1.01 0.84 1.00 0.97 1.10 1.02 0.99 1.00 0.97 0.99 3.05 1.16 3.01 1.06 1.03 0.98 0.97 2.95 3.05 2.96
11.5 11.0 10.5 10.0 9.5 9.0 8.5 8.0 7.5 7.0 6.5 6.0 5.5 5.0 4.5 4.0 3.5 3.0 2.5 2.0 1.5 1.0 0.5 0.0
f1 (ppm)

^{13}C NMR of **38**

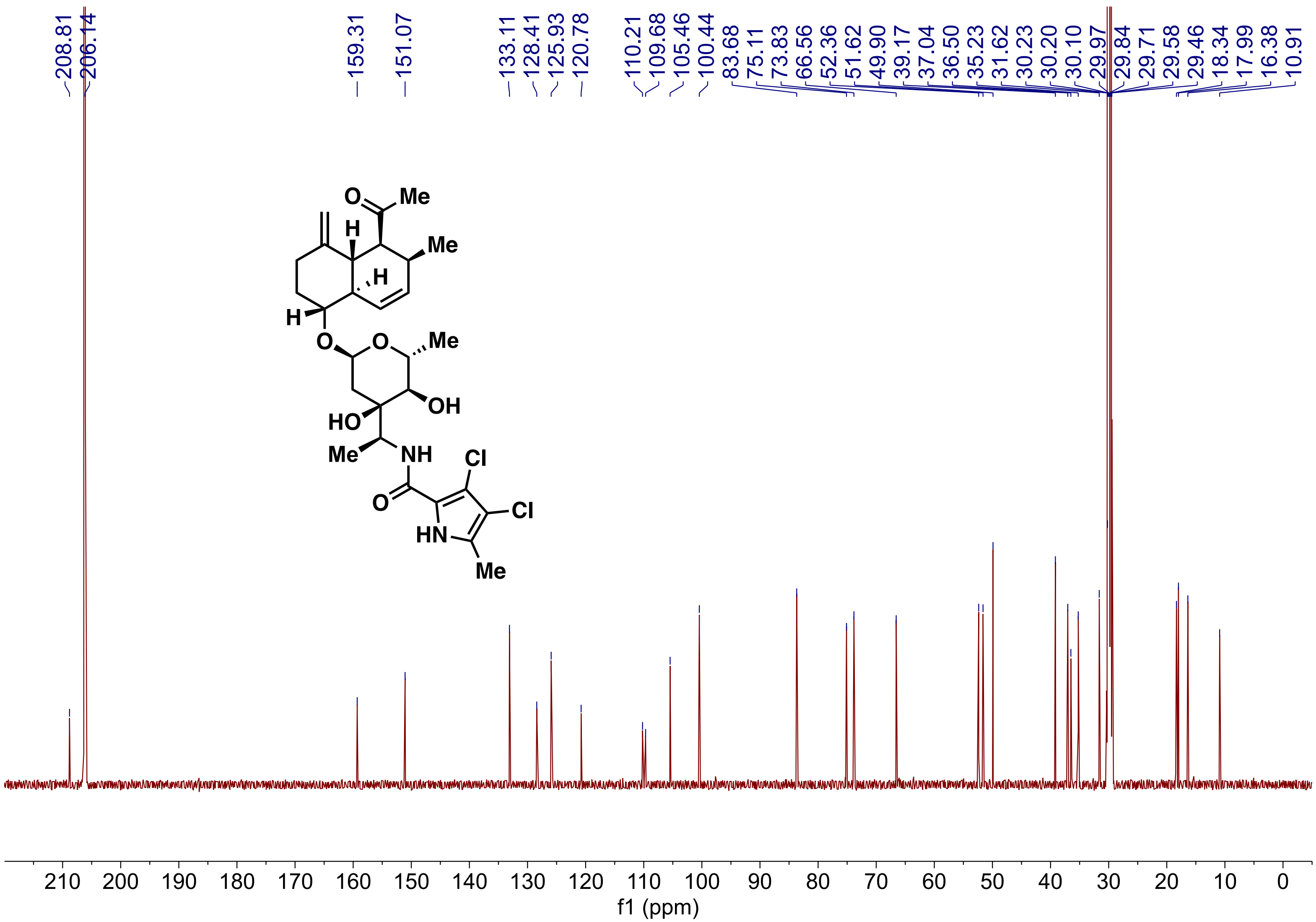

208.81
206.14
159.31
151.07
133.11
128.41
125.93
120.78
110.21
109.68
105.46
100.44
83.68
75.11
73.83
66.56
52.36
51.62
49.90
39.17
37.04
36.50
35.23
31.62
30.23
30.20
30.10
29.97
29.84
29.71
29.58
29.46
18.34
17.99
16.38
10.91
O
Me
H
Me
H
H
O
O
Me
HO
OH
Me
NH
Cl
O
Cl
HN
Me
210 200 190 180 170 160 150 140 130 120 110 100 90 80 70 60 50 40 30 20 10 0
f1 (ppm)

^{1}H NMR of **39**

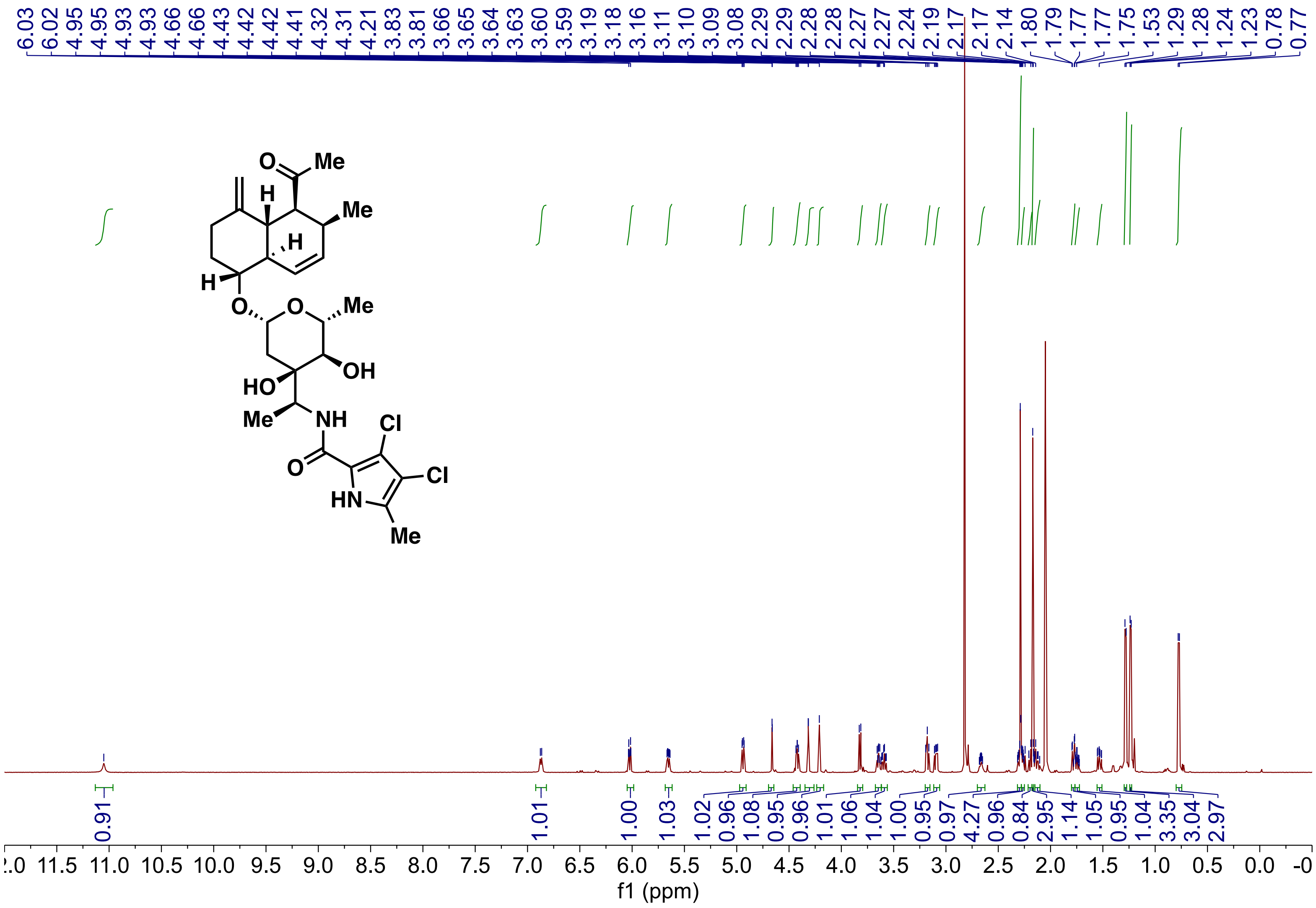

^{13}C NMR of **39**

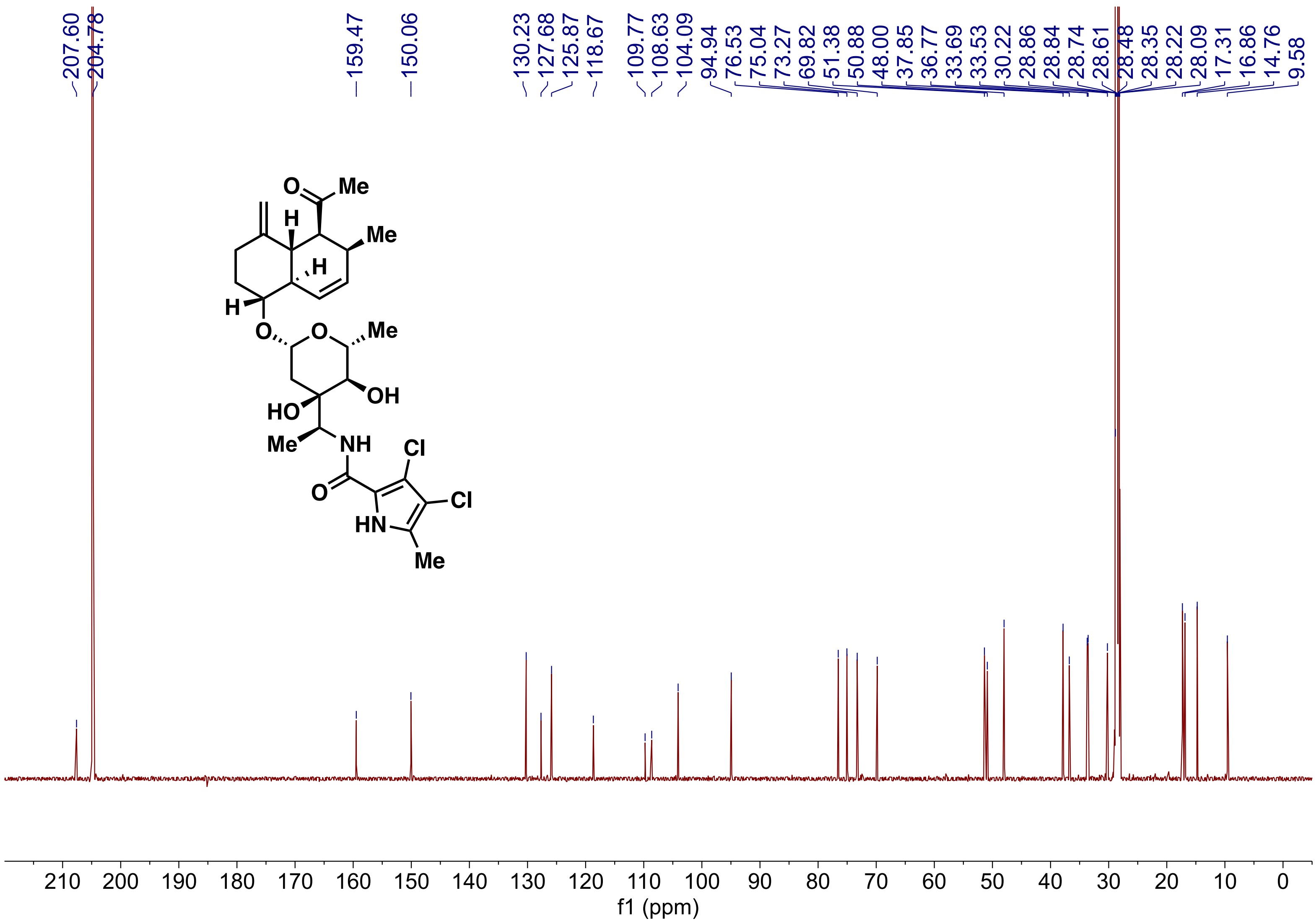

207.60
204.78
159.47
150.06
130.23
127.68
125.87
118.67
109.77
108.63
104.09
94.94
76.53
75.04
73.27
69.82
51.38
50.88
48.00
37.85
36.77
33.69
33.53
30.22
28.86
28.84
28.74
28.61
28.48
28.35
28.22
28.09
17.31
16.86
14.76
9.58
O
Me
H
Me
H
H
O
O
Me
OH
HO
Me
NH
Cl
Cl
O
HN
Me
210
200
190
180
170
160
150
140
130
120
110
100
90
80
70
60
50
40
30
20
10
0
f1 (ppm)

^{1}H NMR of **40**

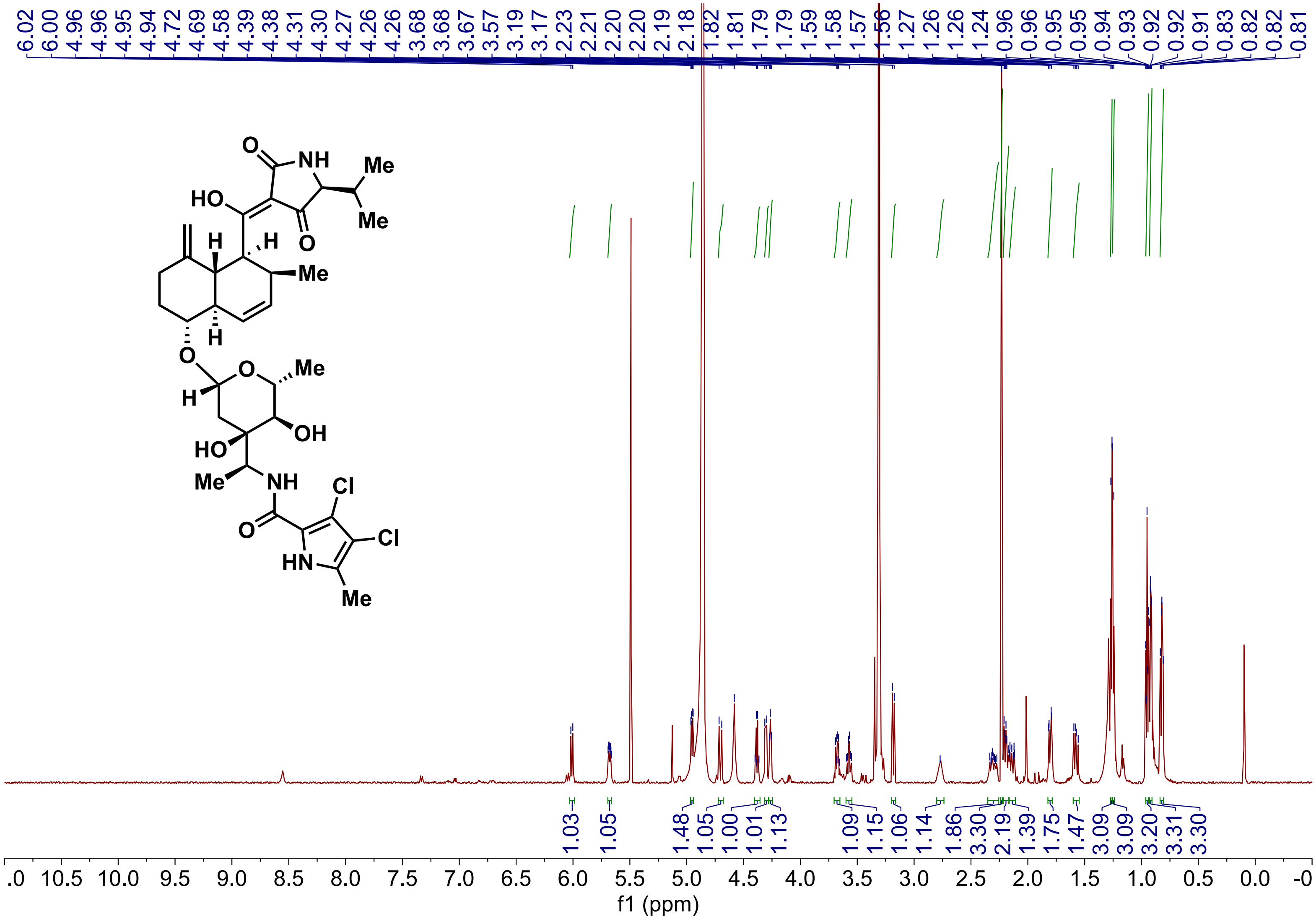

^{13}C NMR of **40**

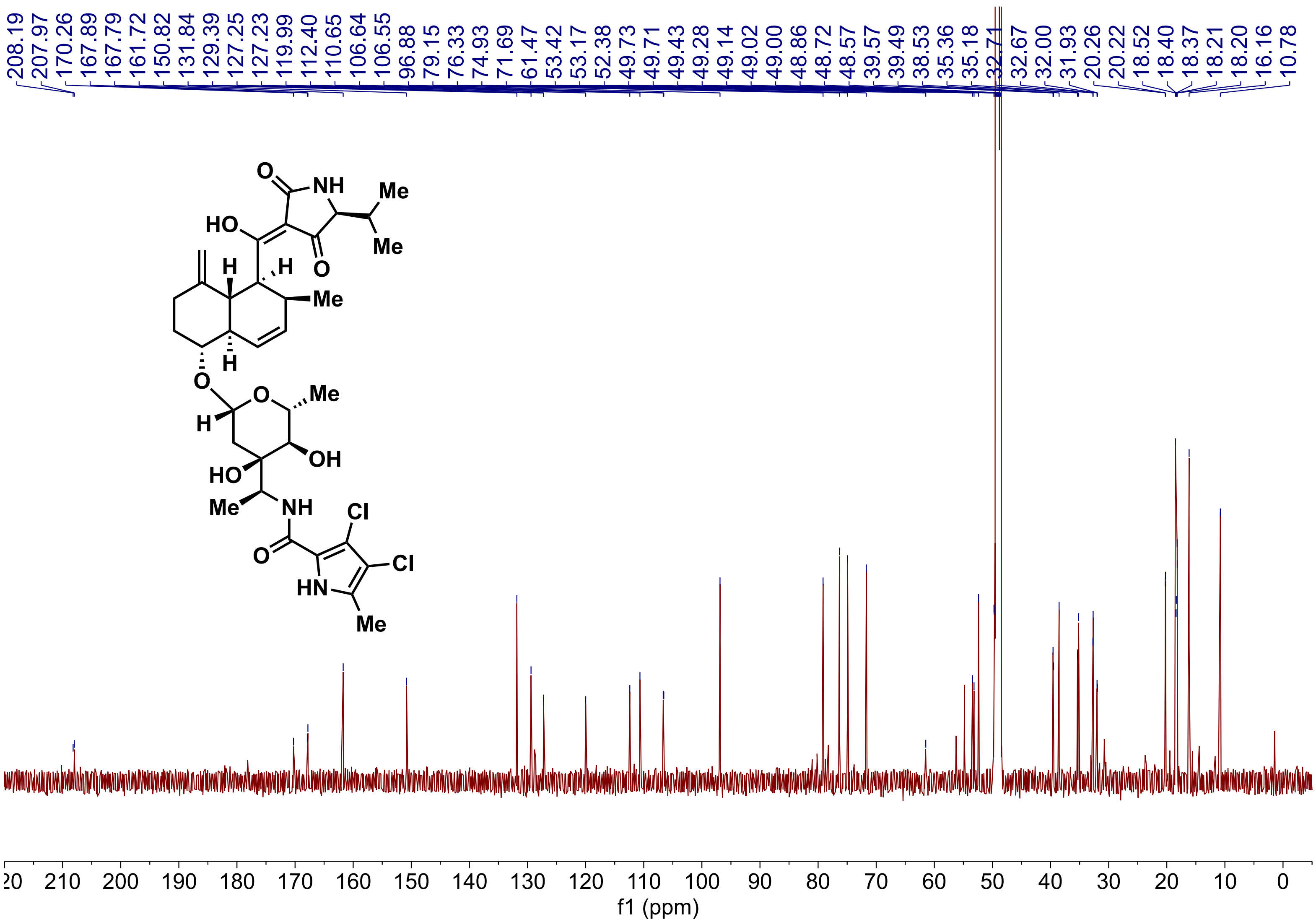

208.19
207.97
170.26
167.89
167.79
161.72
150.82
131.84
129.39
127.25
127.23
119.99
112.40
110.65
106.64
106.55
96.88
79.15
76.33
74.93
71.69
61.47
53.42
53.17
52.38
49.73
49.71
49.43
49.28
49.14
49.02
49.00
48.86
48.72
48.57
39.57
39.49
38.53
35.36
35.18
32.71
32.67
32.00
31.93
20.26
20.22
18.52
18.40
18.37
18.21
18.20
16.16
10.78
f1 (ppm)

HSQC of **40**

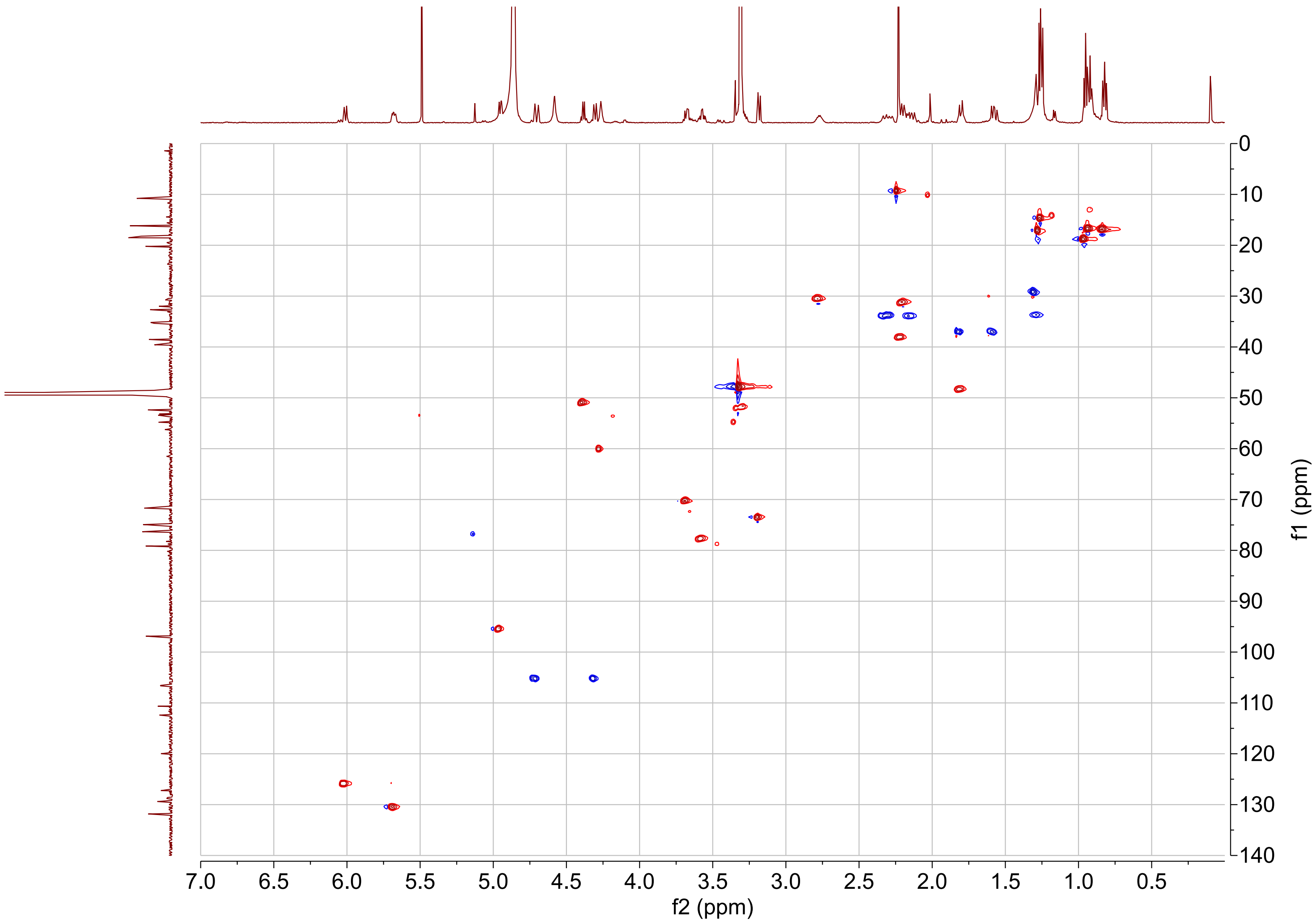

0
10
20
30
40
50
60
70
80
90
100
110
120
130
140
f1 (ppm)
7.0
6.5
6.0
5.5
5.0
4.5
4.0
3.5
3.0
2.5
2.0
1.5
1.0
0.5
f2 (ppm)

COSY of **40**

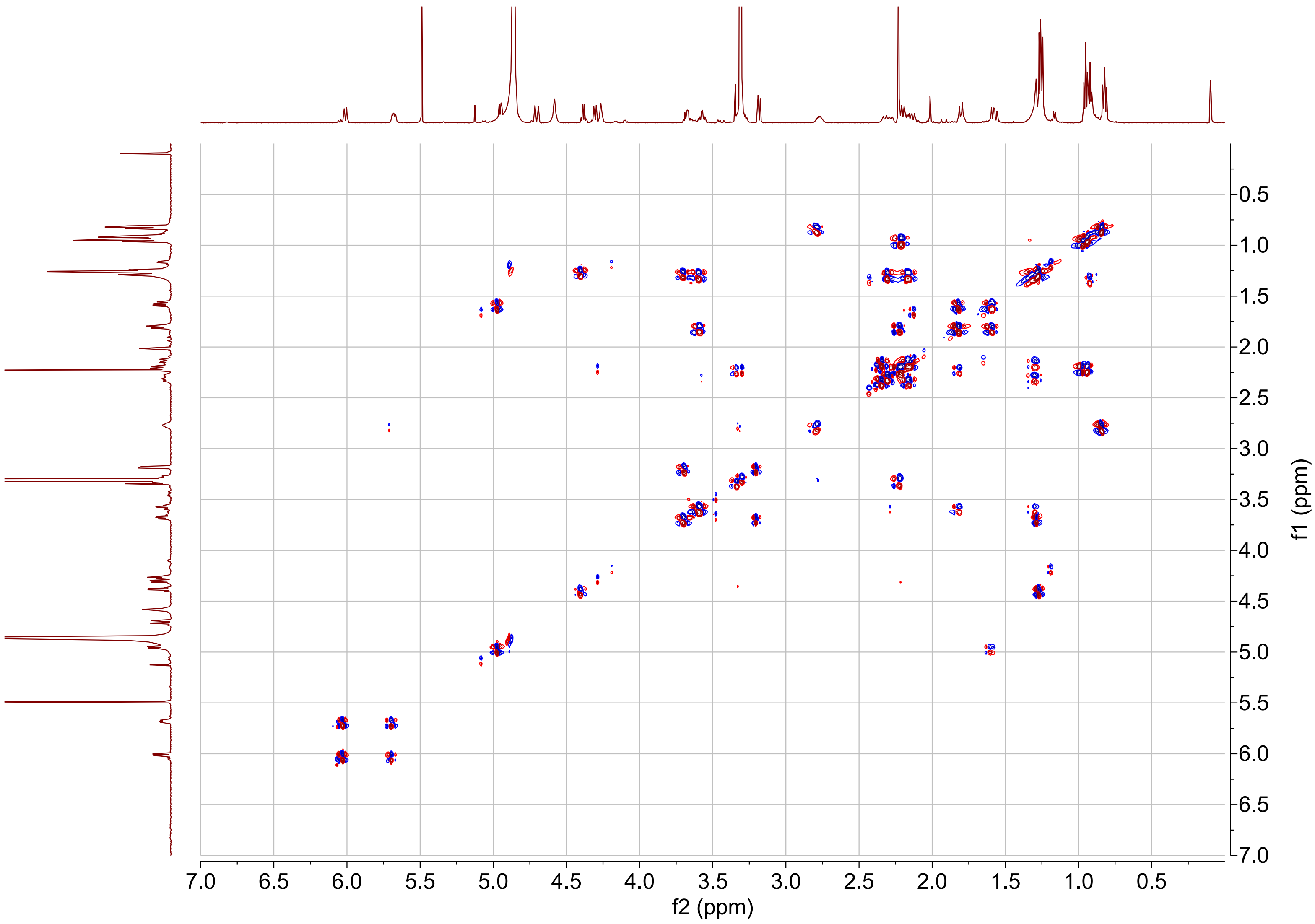

^{1}H NMR of **S10**

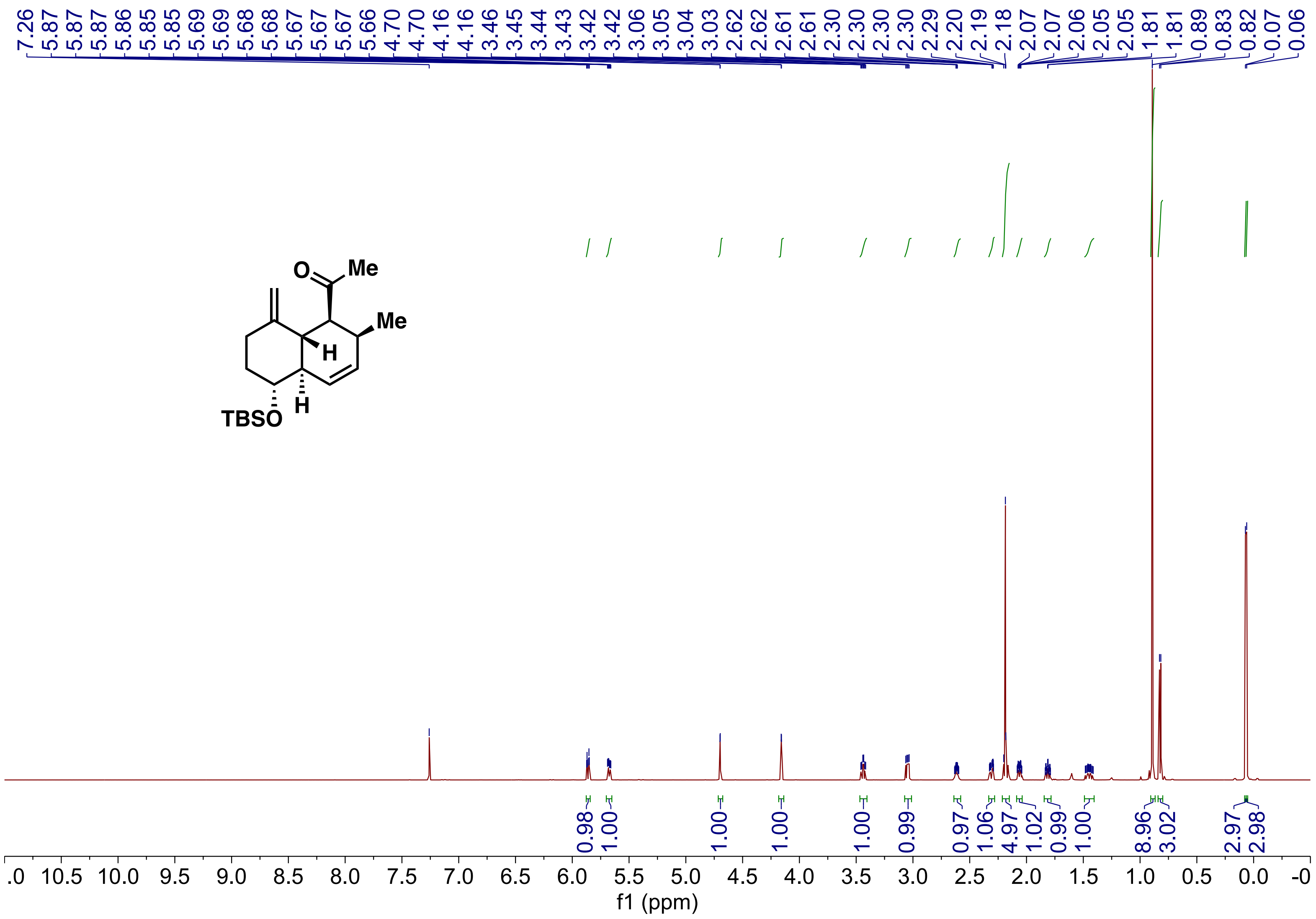
7.26
5.87
5.87
5.87
5.86
5.85
5.85
5.69
5.69
5.68
5.68
5.67
5.67
5.67
5.66
4.70
4.70
4.16
4.16
3.46
3.45
3.44
3.43
3.42
3.42
3.06
3.05
3.04
3.03
2.62
2.62
2.61
2.61
2.30
2.30
2.30
2.30
2.29
2.20
2.19
2.18
2.07
2.07
2.06
2.05
2.05
1.81
1.81
1.81
0.89
0.83
0.82
0.07
0.06
O
Me
Me
H
H
TBSO
0.98
1.00
1.00
1.00
1.00
0.99
0.97
1.06
4.97
1.02
0.99
1.00
8.96
3.02
2.97
2.98
f1 (ppm)

^{13}C NMR of **S10**

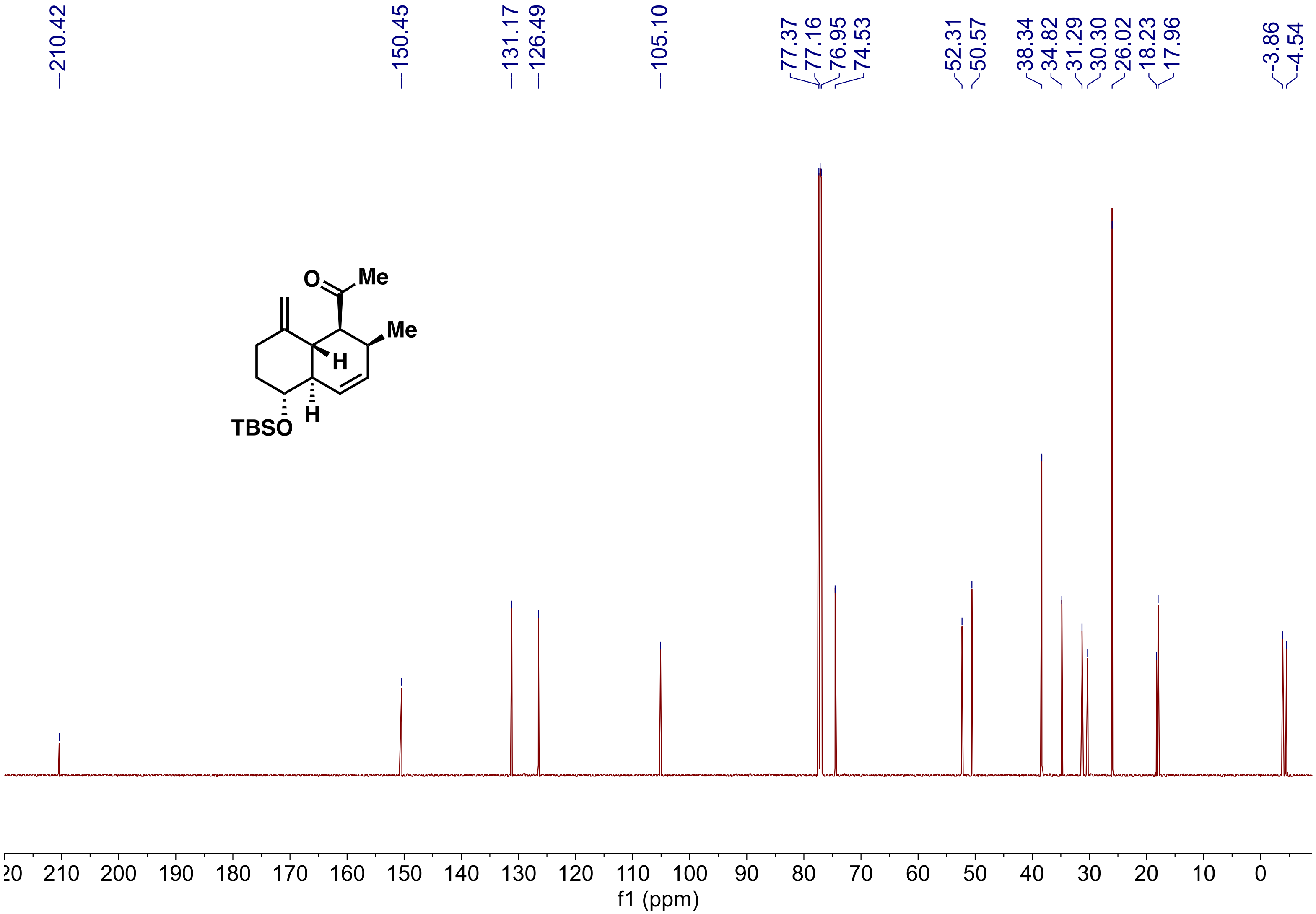

^{1}H NMR of **S11**

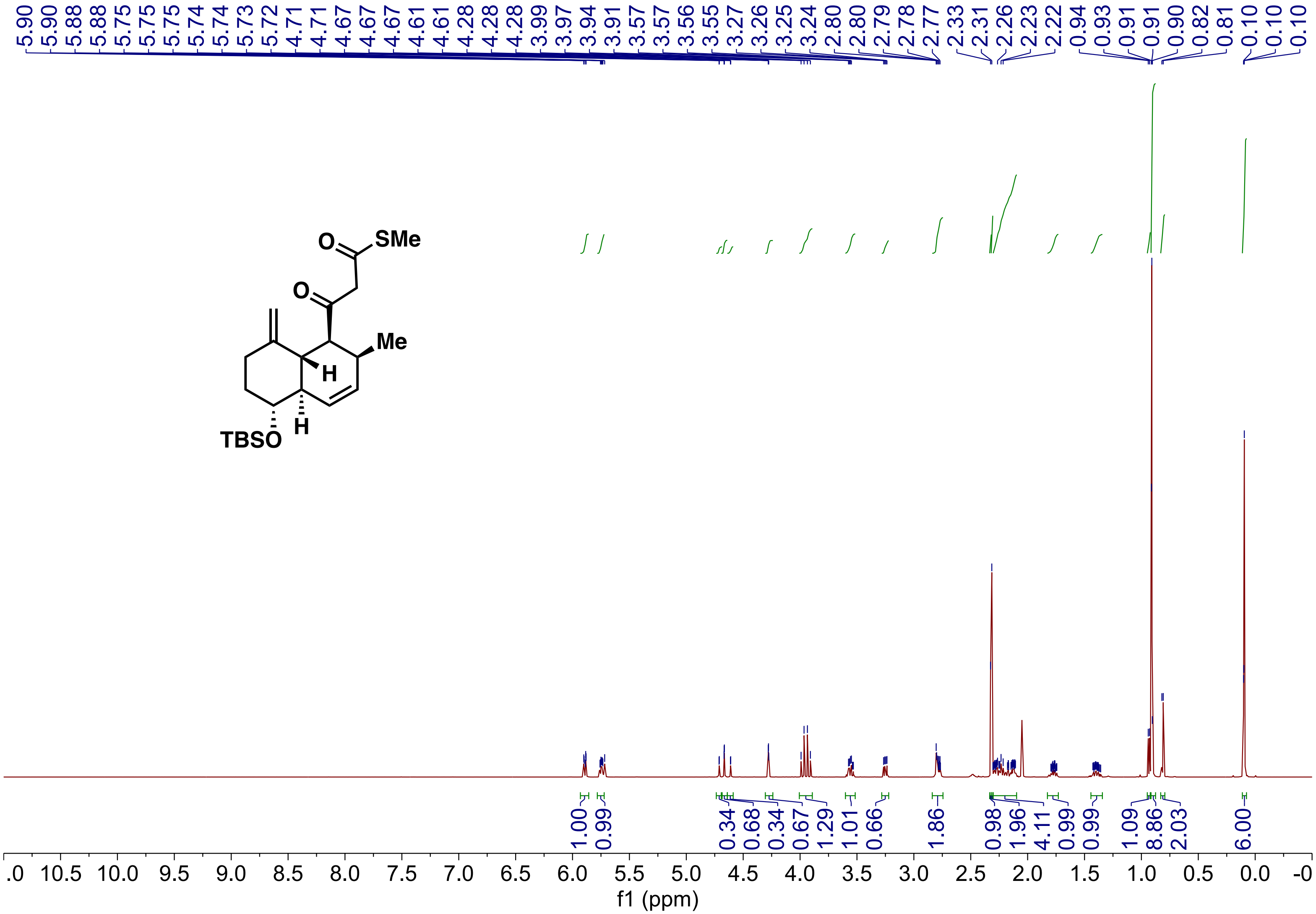

^{13}C NMR of **S11**

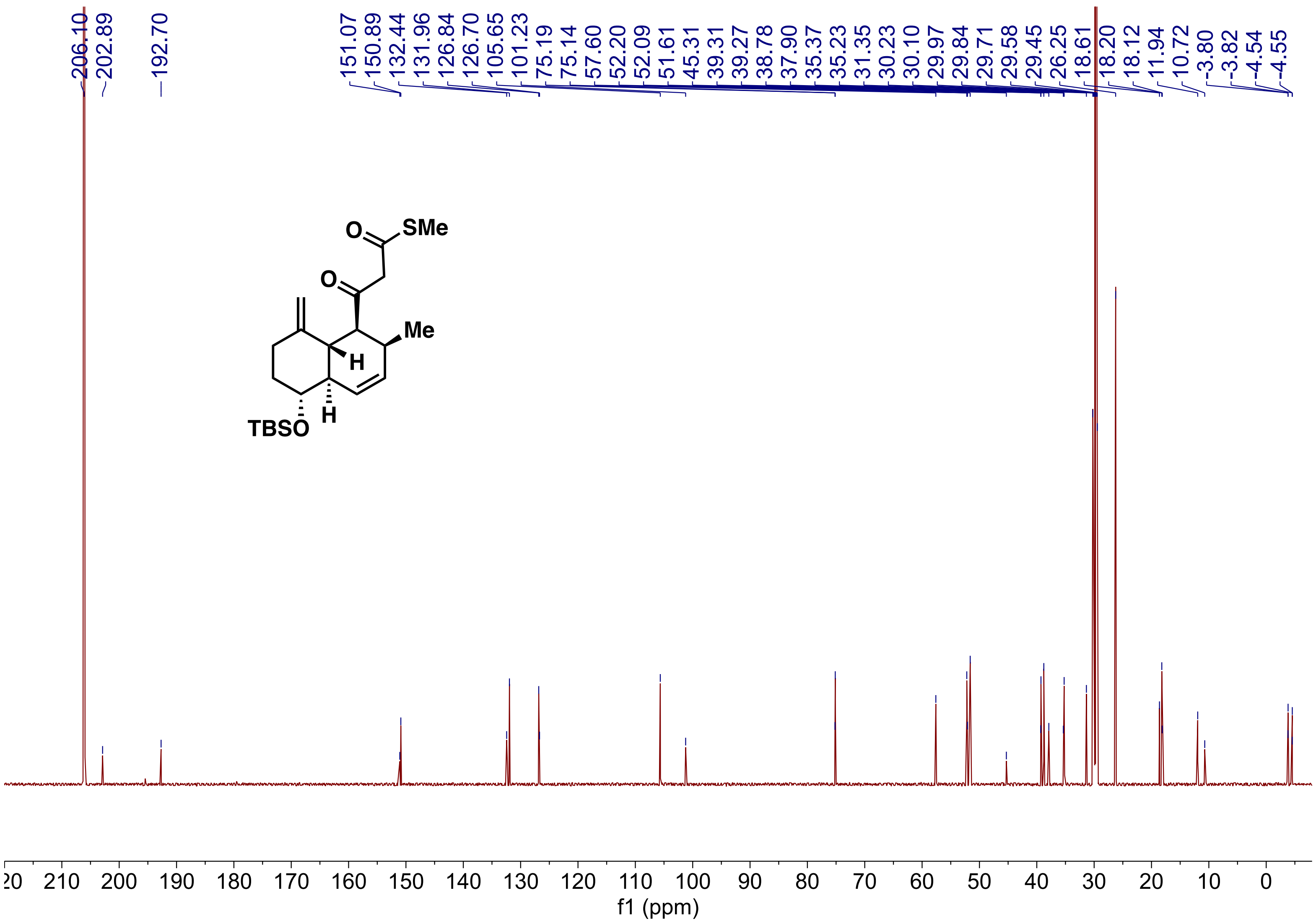

^{1}H NMR of **41**

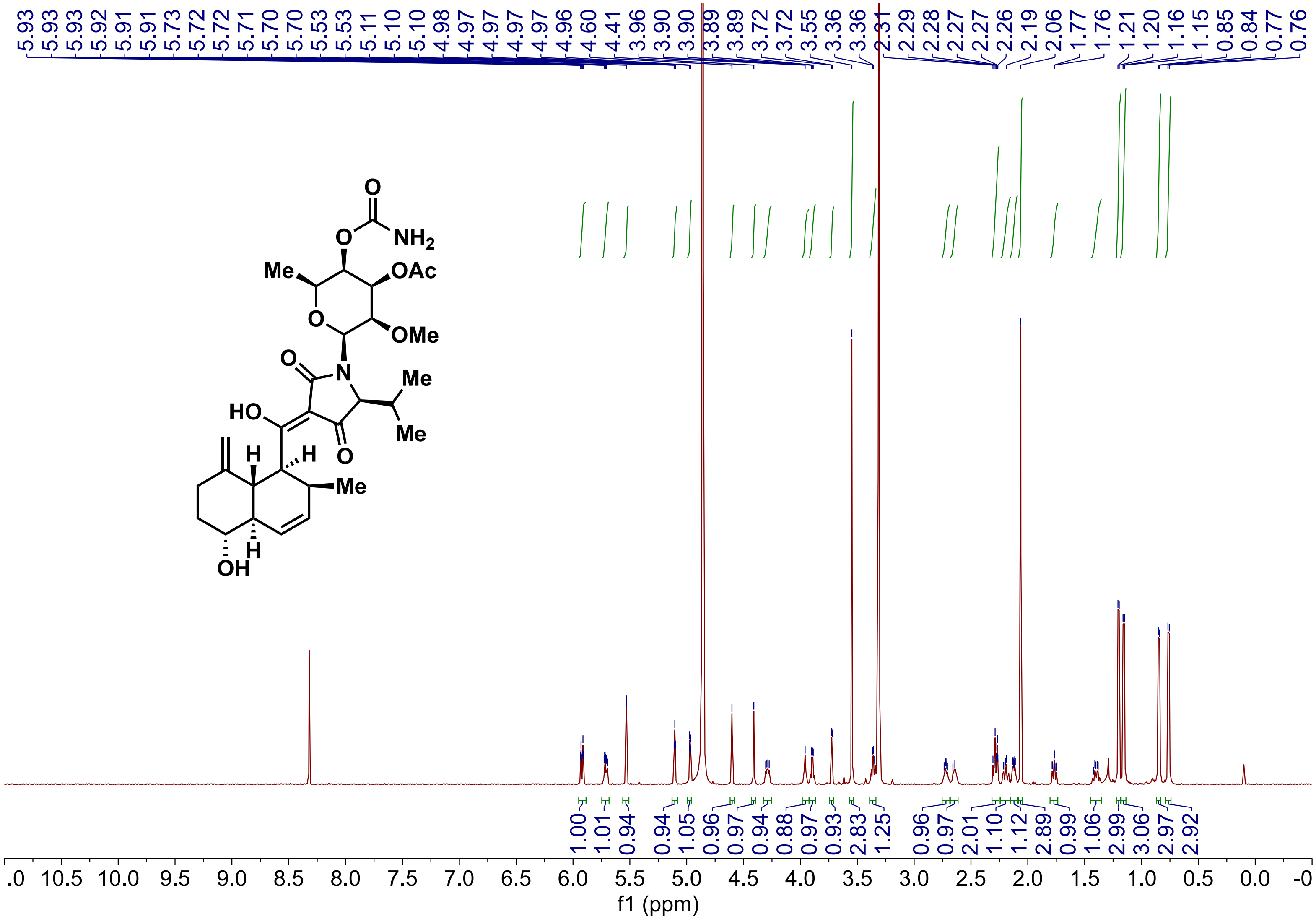

^{13}C NMR of **41**

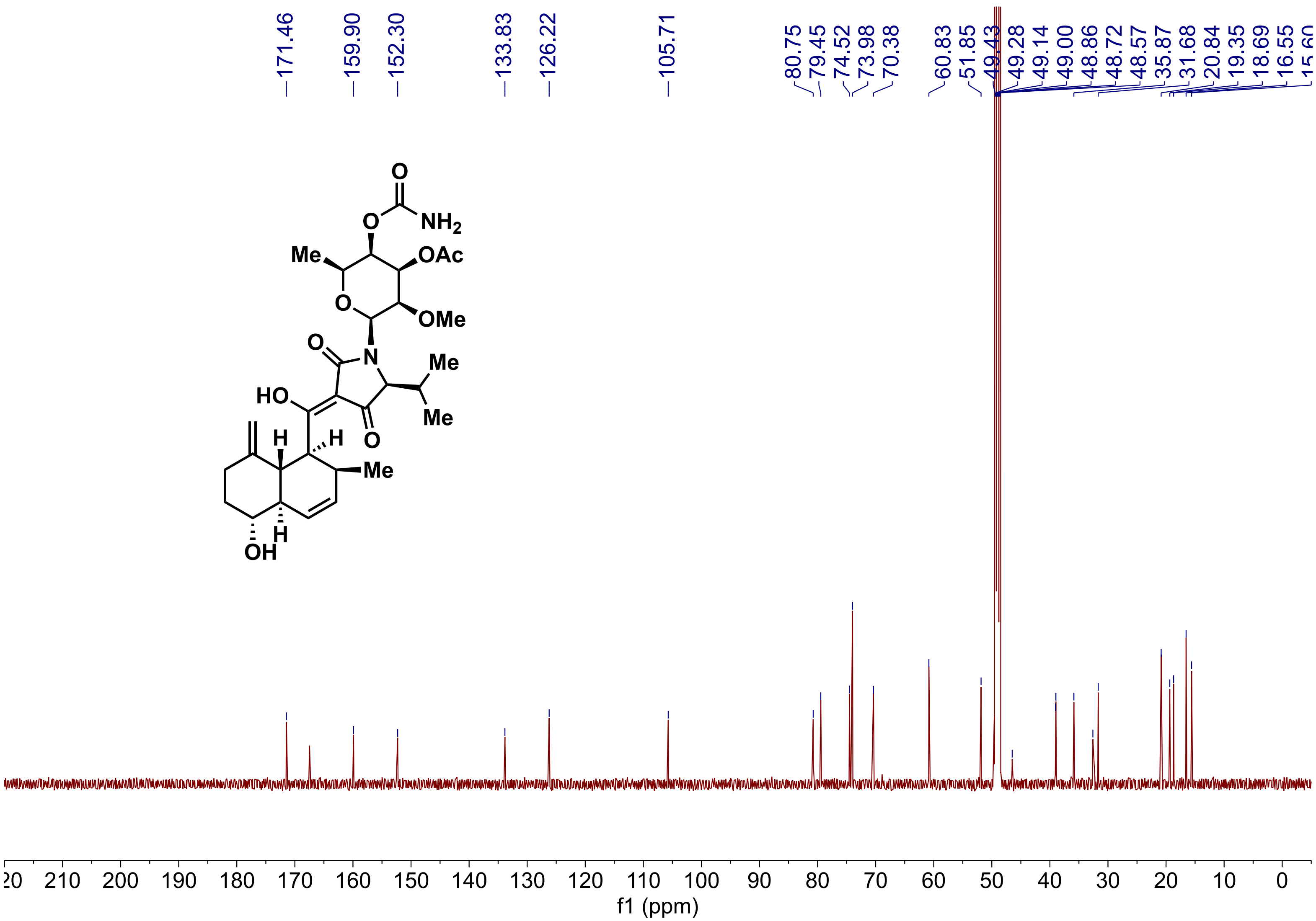
171.46
159.90
152.30
133.83
126.22
105.71
80.75
79.45
74.52
73.98
70.38
60.83
51.85
49.43
49.28
49.14
49.00
48.86
48.72
48.57
35.87
31.68
20.84
19.35
18.69
16.55
O
NH2
O
Me
OAc
O
OMe
O
N
Me
HO
Me
H
H
O
Me
H
OH
20
210
200
190
180
170
160
150
140
130
120
110
100
90
80
70
60
50
40
30
20
10
0
f1 (ppm)

HSQC of **41**

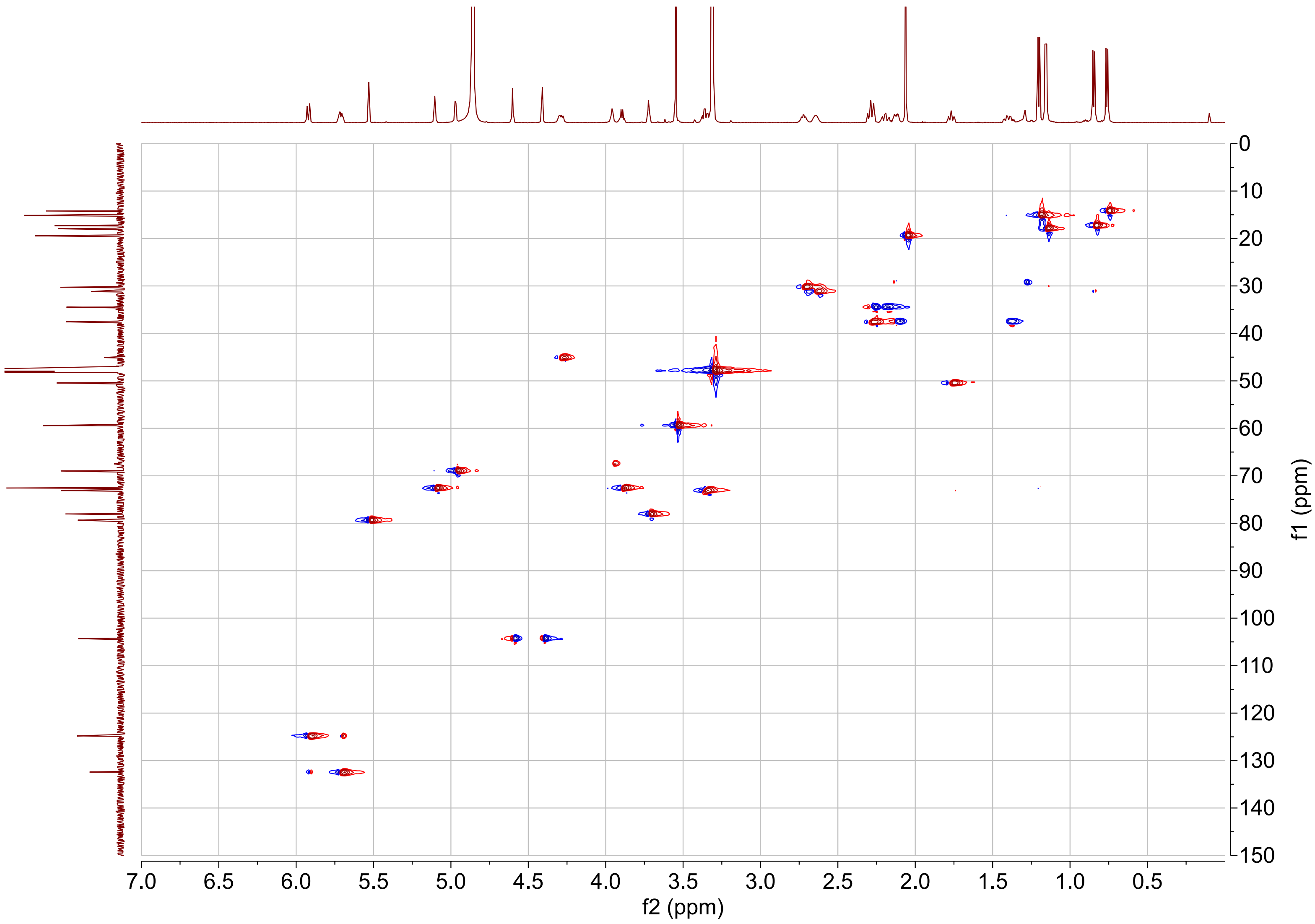

7.0
6.5
6.0
5.5
5.0
4.5
4.0
3.5
3.0
2.5
2.0
1.5
1.0
0.5
f2 (ppm)
0
10
20
30
40
50
60
70
80
90
100
110
120
130
140
150
f1 (ppm)

COSY of **41**

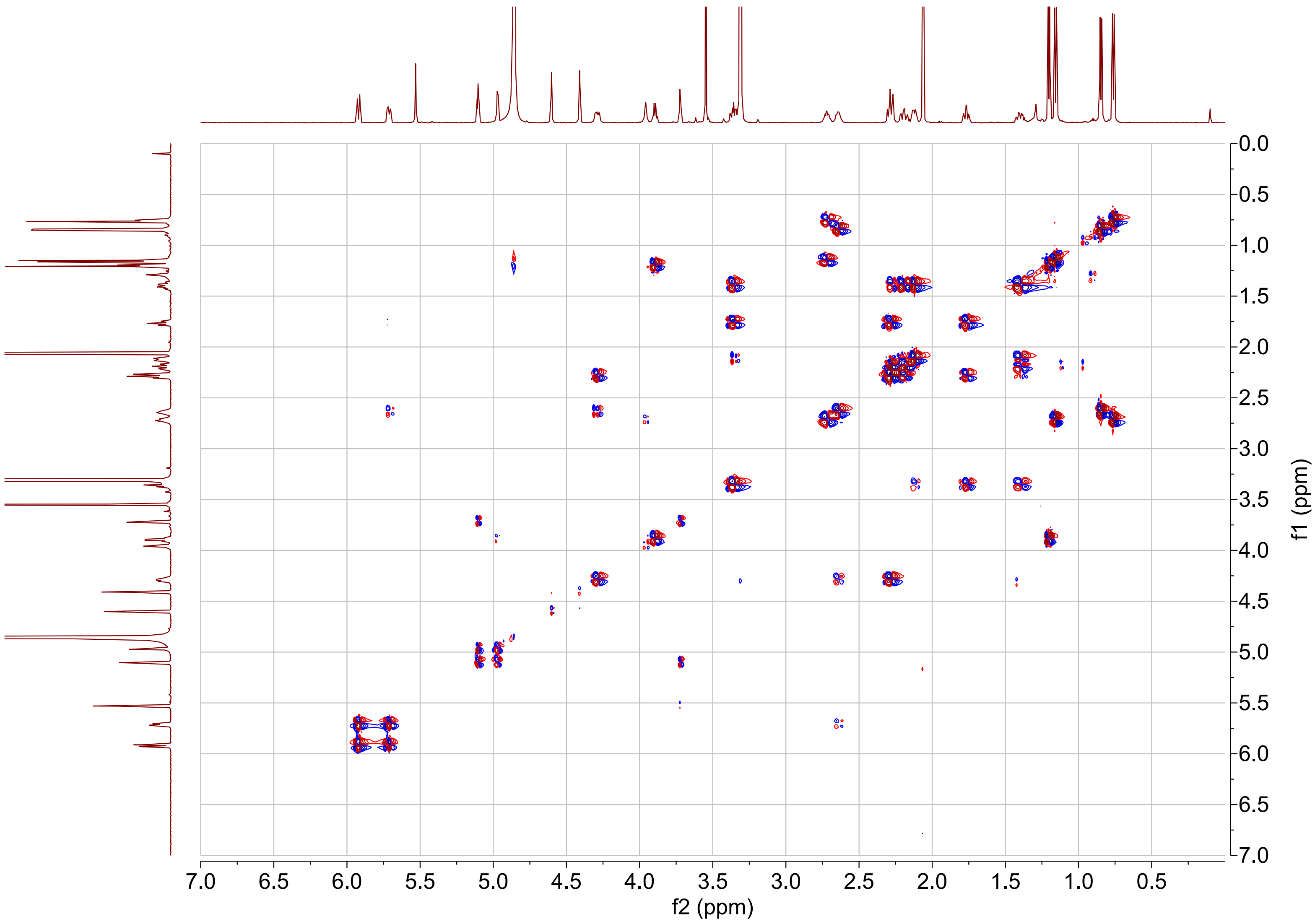

7.0
6.5
6.0
5.5
5.0
4.5
4.0
3.5
3.0
2.5
2.0
1.5
1.0
0.5
f2 (ppm)
0.0
0.5
1.0
1.5
2.0
2.5
3.0
3.5
4.0
4.5
5.0
5.5
6.0
6.5
7.0
f1 (ppm)

NOESY of **41**

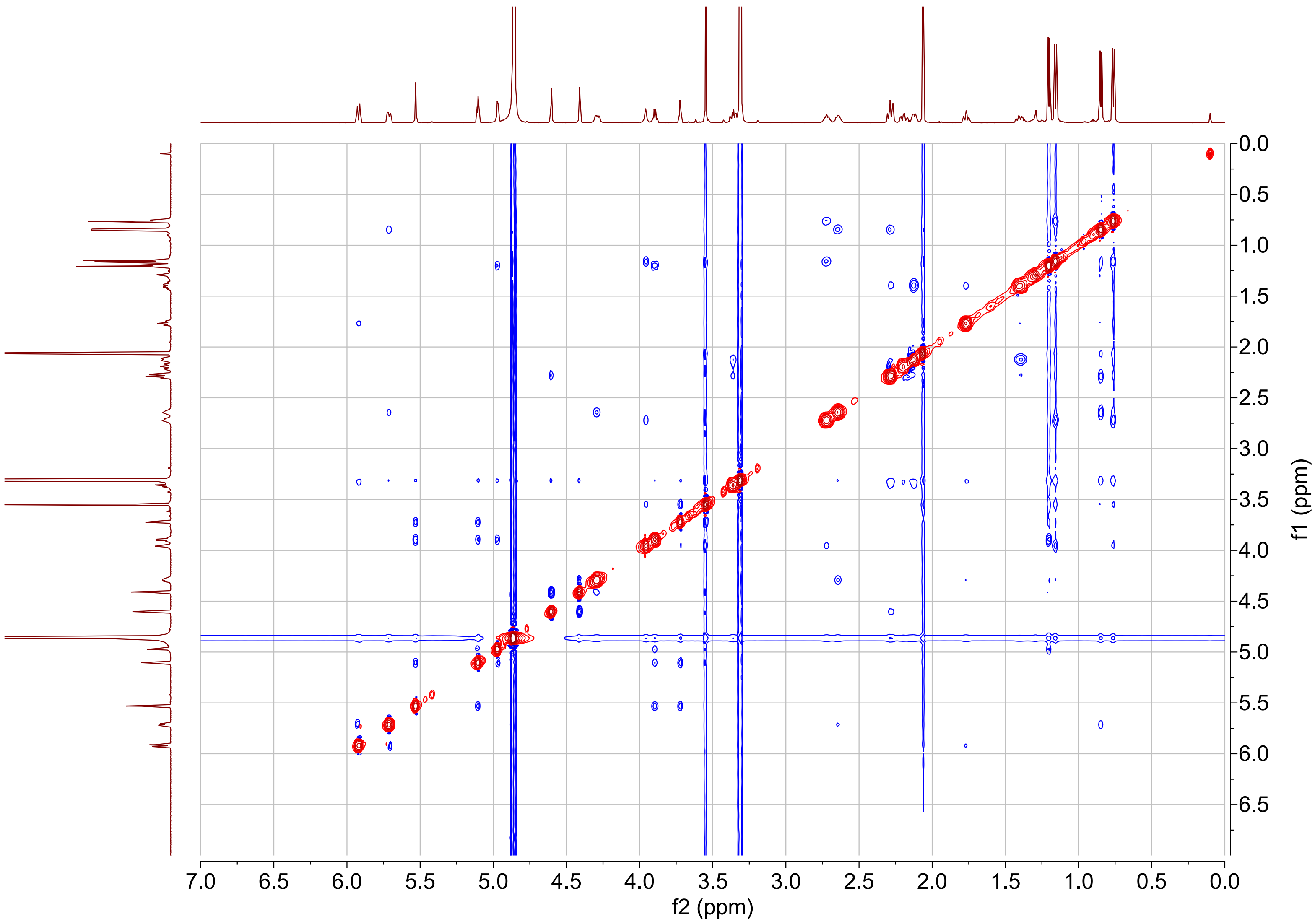

^{1}H NMR of **42**

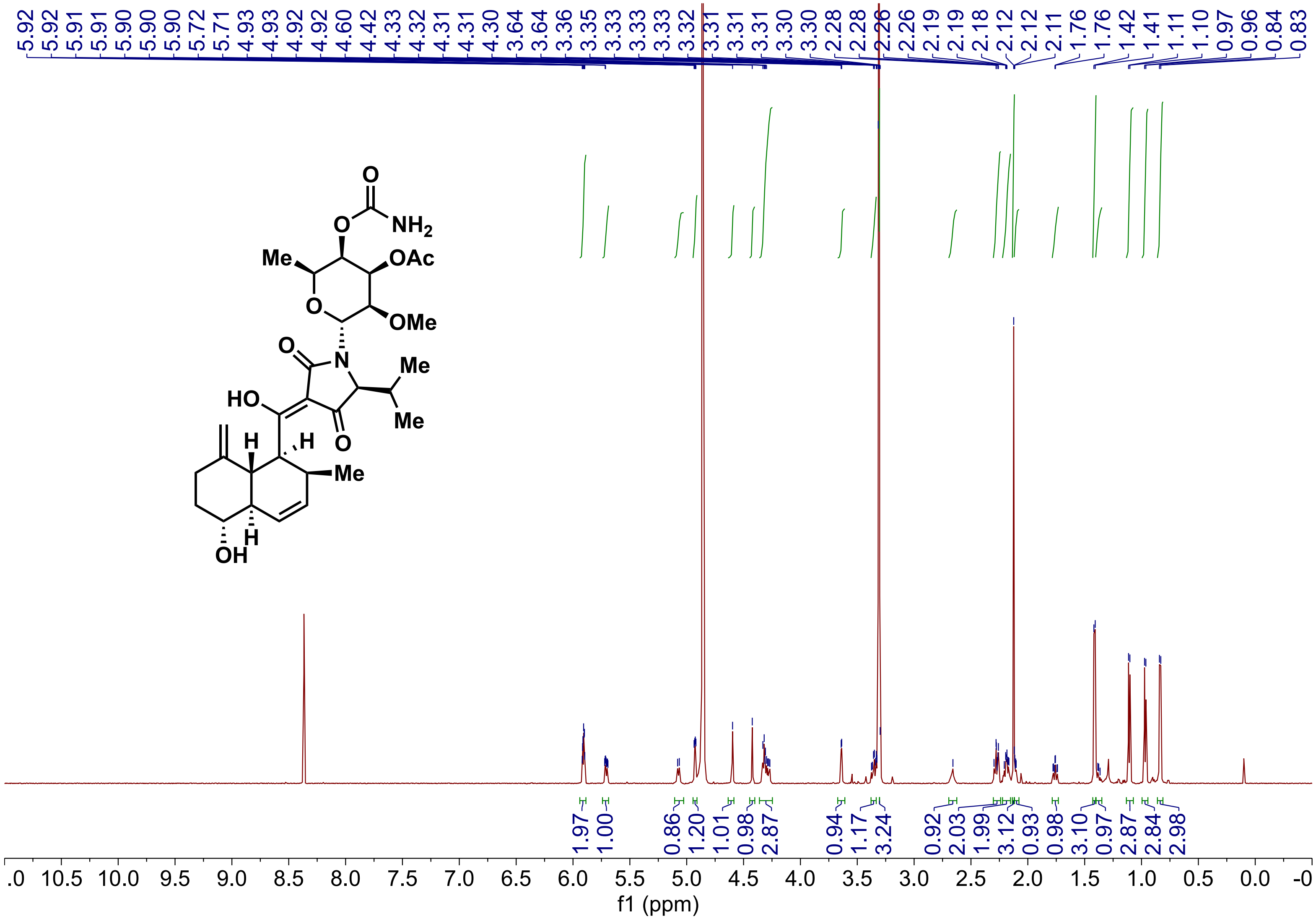

^{13}C NMR of **42**

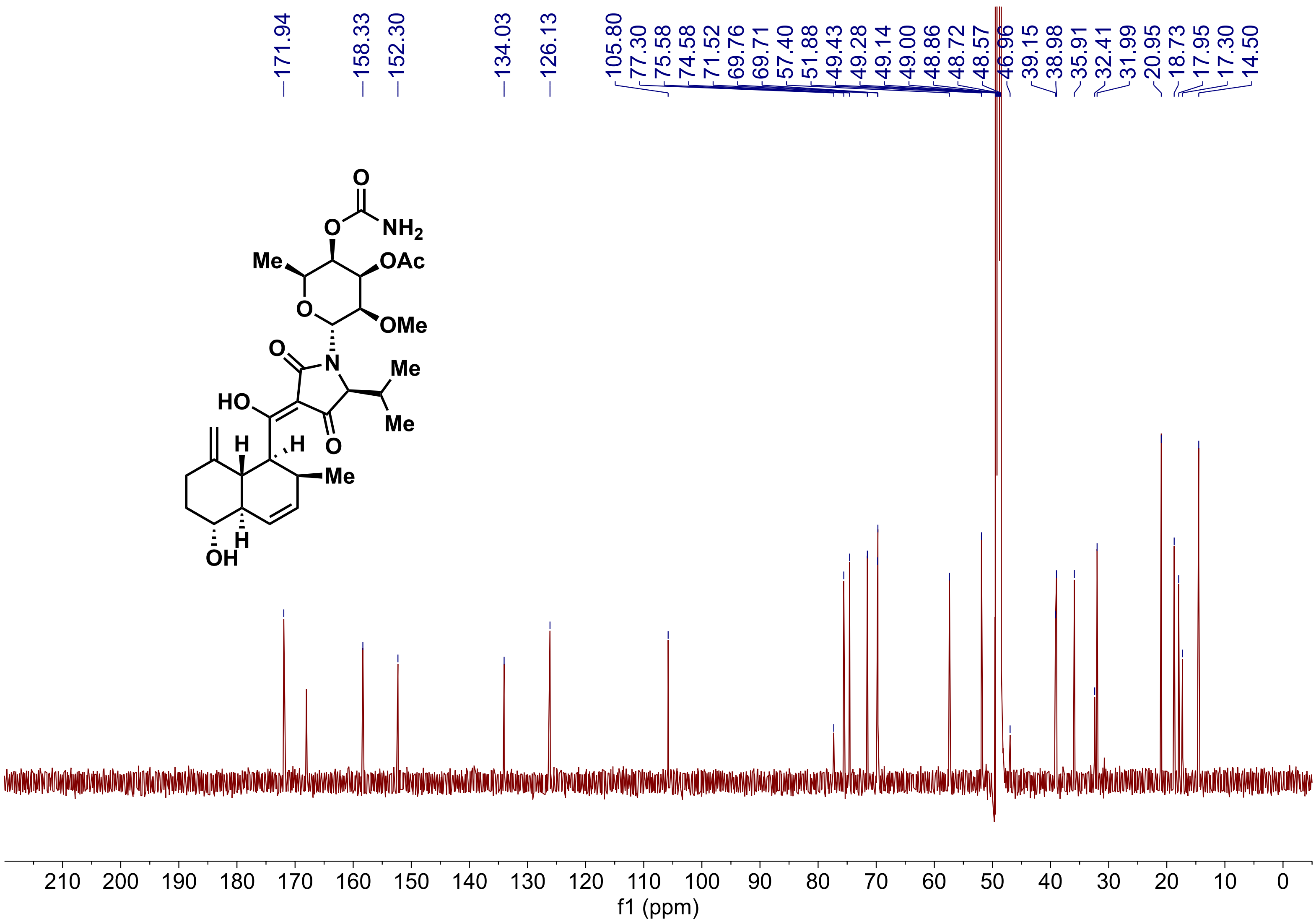

HSQC of **42**

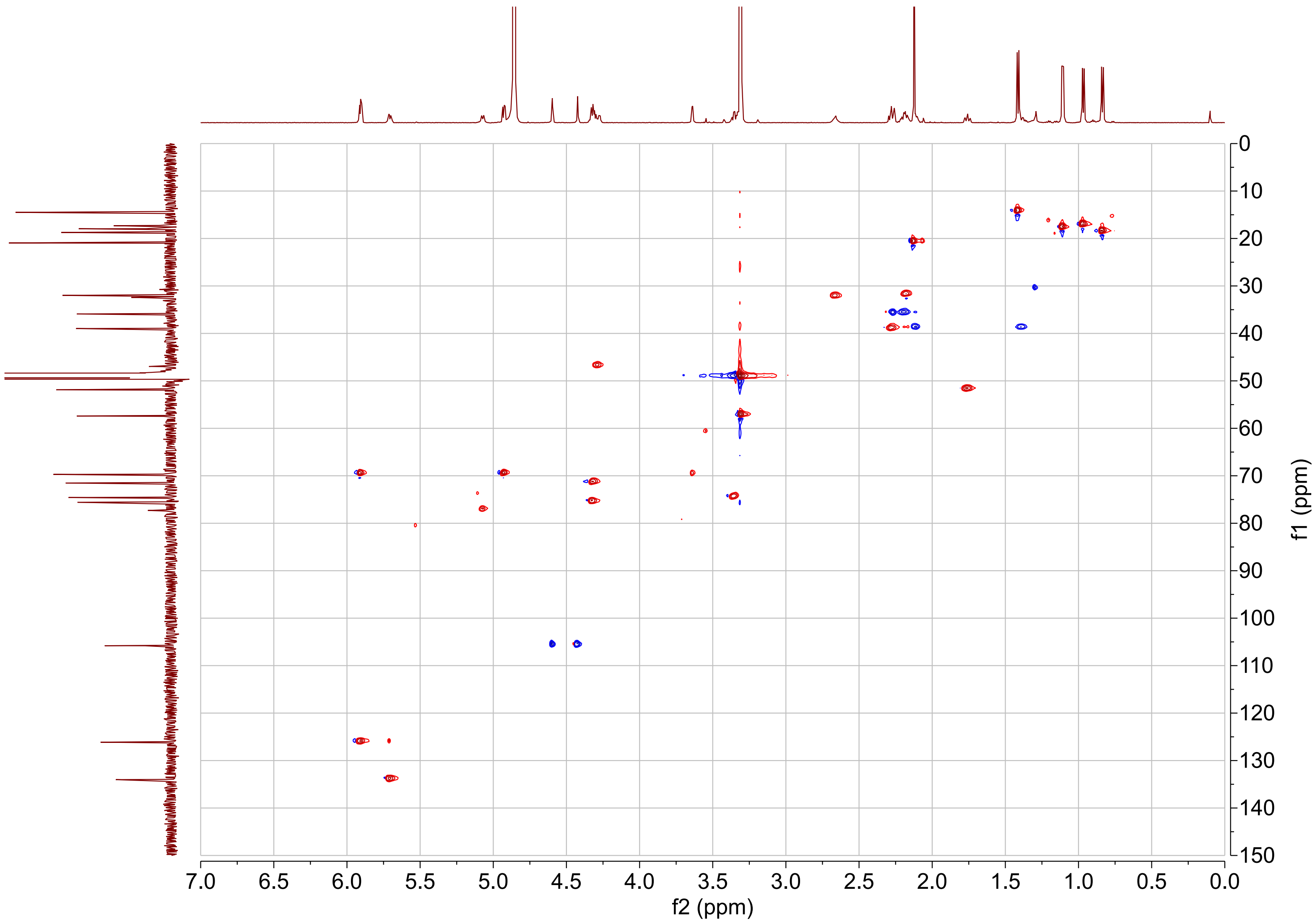
f2 (ppm)
f1 (ppm)
7.0
6.5
6.0
5.5
5.0
4.5
4.0
3.5
3.0
2.5
2.0
1.5
1.0
0.5
0.0
0
10
20
30
40
50
60
70
80
90
100
110
120
130
140
150

COSY of **42**

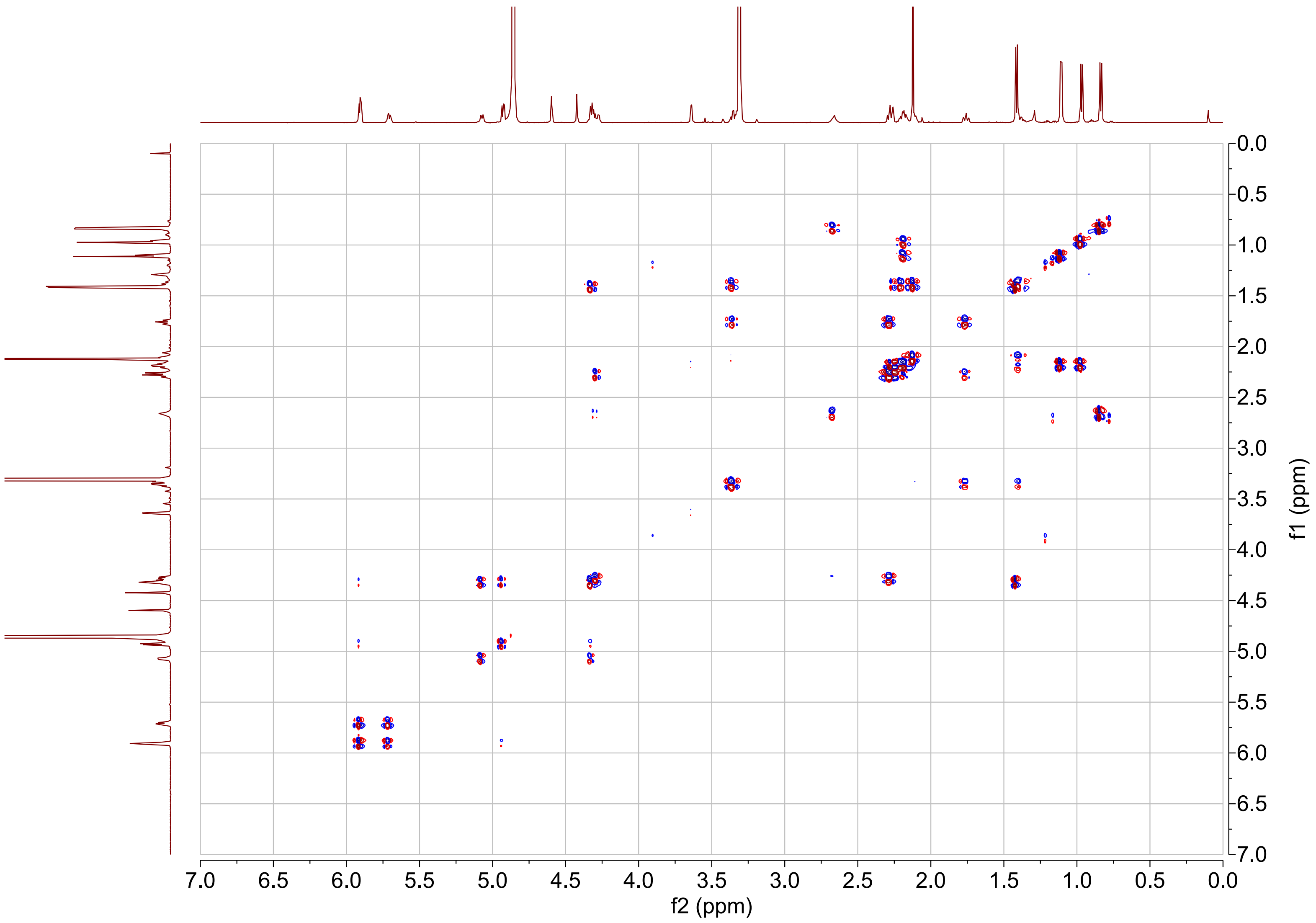
7.0
6.5
6.0
5.5
5.0
4.5
4.0
3.5
3.0
2.5
2.0
1.5
1.0
0.5
0.0
f2 (ppm)
0.0
0.5
1.0
1.5
2.0
2.5
3.0
3.5
4.0
4.5
5.0
5.5
6.0
6.5
7.0
f1 (ppm)

NOESY of **42**

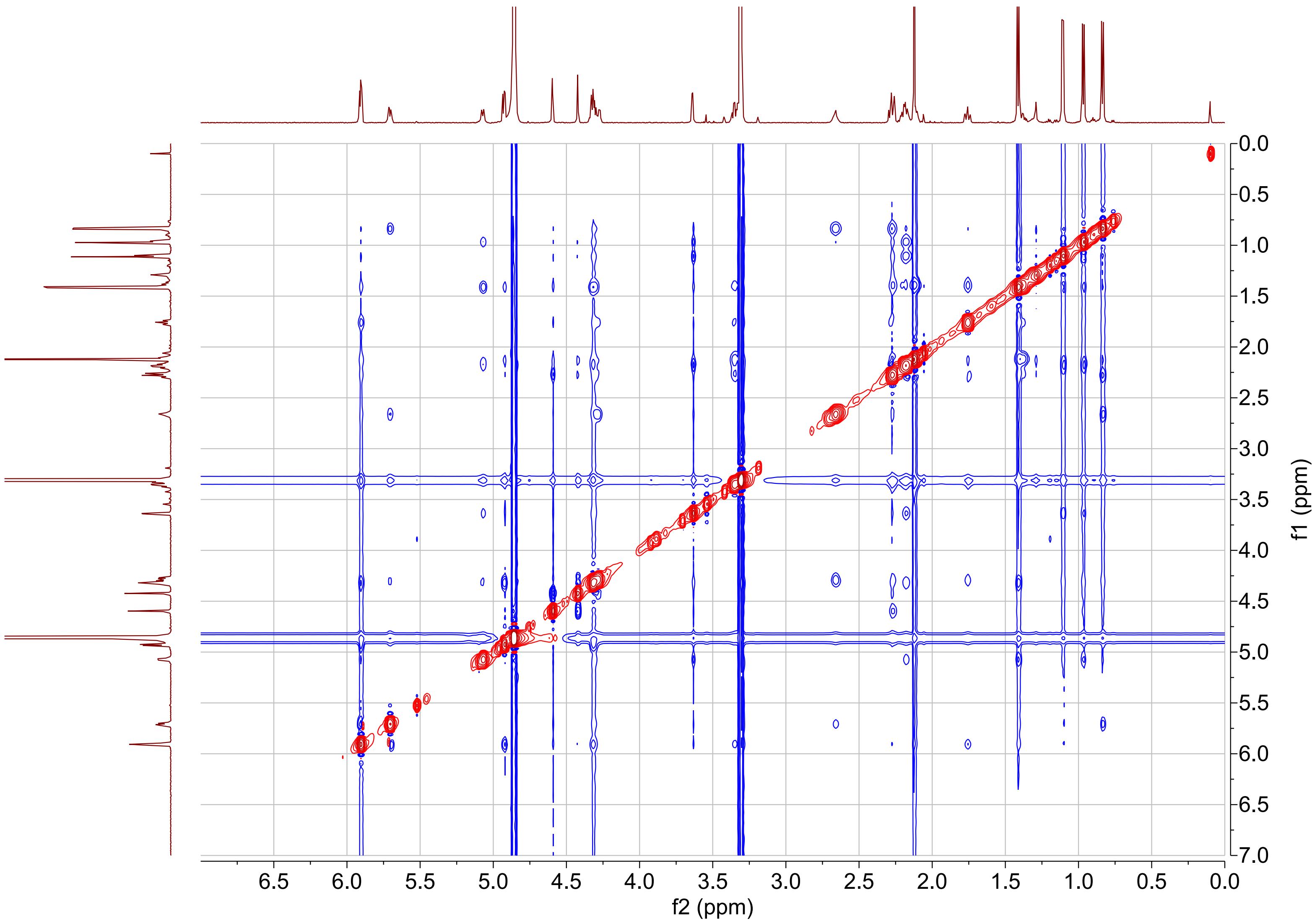

6.5
6.0
5.5
5.0
4.5
4.0
3.5
3.0
2.5
2.0
1.5
1.0
0.5
0.0
f2 (ppm)
0.0
0.5
1.0
1.5
2.0
2.5
3.0
3.5
4.0
4.5
5.0
5.5
6.0
6.5
7.0
f1 (ppm)
